# Performance of Chimeric *Trypanosoma cruzi* Antigens in Serological Screening for Chagas Disease in Blood Banks

**DOI:** 10.3389/fmed.2022.852864

**Published:** 2022-03-07

**Authors:** Emily Ferreira dos Santos, Ângelo Antônio Oliveira Silva, Natália Erdens Maron Freitas, Leonardo Maia Leony, Ramona Tavares Daltro, Carlos Antônio de Souza Teles Santos, Maria da Conceição Chagas de Almeida, Fernando Luiz Vieira de Araújo, Paola Alejandra Fiorani Celedon, Marco Aurélio Krieger, Nilson Ivo Tonin Zanchin, Mitermayer Galvão dos Reis, Fred Luciano Neves Santos

**Affiliations:** ^1^Advanced Health Public Laboratory, Gonçalo Moniz Institute, Oswaldo Cruz Foundation - Bahia (FIOCRUZ-BA), Salvador, Brazil; ^2^Center for Integration of Data and Health Knowledge (CIDACS), Gonçalo Moniz Institute, Oswaldo Cruz Foundation - Bahia (FIOCRUZ-BA), Salvador, Brazil; ^3^Molecular Epidemiology and Biostatistics Laboratory, Gonçalo Moniz Institute, Oswaldo Cruz Foundation - Bahia (FIOCRUZ-BA), Salvador, Brazil; ^4^Hematology and Hemotherapy Foundation of the State of Bahia (HEMOBA), Salvador, Brazil; ^5^Laboratory of Molecular Biology of Trypanosomatids, Carlos Chagas Institute, Oswaldo Cruz Foundation - Paraná (FIOCRUZ-PR), Curitiba, Brazil; ^6^Laboratory for Applied Science and Technology in Health, Carlos Chagas Institute, Oswaldo Cruz Foundation - Paraná (FIOCRUZ-PR), Curitiba, Brazil; ^7^Integrated Translational Program in Chagas Disease From Fiocruz (Fio-Chagas), Oswaldo Cruz Foundation – Rio de Janeiro (FIOCRUZ-RJ), Rio de Janeiro, Brazil; ^8^Structural Biology and Protein Engineering, Carlos Chagas Institute, Oswaldo Cruz Foundation - Paraná (FIOCRUZ-PR), Curitiba, Brazil; ^9^Pathology and Molecular Biology Laboratory, Gonçalo Moniz Institute, Oswaldo Cruz Foundation - Bahia (FIOCRUZ-BA), Salvador, Brazil; ^10^Faculty of Medicine of Bahia, Federal University of Bahia, Salvador, Brazil; ^11^Department of Epidemiology of Microbial Diseases, Yale School of Public Health, Yale University, New Haven, CT, United States

**Keywords:** Chagas disease, blood bank, recombinant antigens, serological screening, diagnostic performance

## Abstract

Chagas disease (CD) is among the top 10 causes of inability to blood donation. Blood donation centers screen for anti-*Trypanosoma cruzi* antibodies using highly sensitive immunoenzymatic (ELISA) or chemiluminescent methods, which can lead to false positive results. Since positive samples cannot be used, to avoid the loss of valuable blood donations, it is necessary to improve specificity without reducing the sensitivity of the tests used for blood screening. For this purpose, our group has developed four chimeric proteins (IBMP-8.1, IBMP-8.2, IBMP-8.3, and IBMP-8.4) that have been evaluated in phase I and II studies with high performance and low cross-reactivity rates. The study included a panel of 5,014 serum samples collected from volunteer blood donors at the Hematology and Hemotherapy Foundation of the State of Bahia (Brazil). They were subjected to the detection of anti-*T. cruzi* antibodies, using all four IBMP antigens individually and latent class analysis (LCA) as a reference test, since there is no gold standard test for this purpose. Considering the sample size analyzed, LCA classified 4,993 (99.6%) samples as *T. cruzi*-negative and 21 (0.42%) as *T. cruzi*-positive. Sensitivity values ranged from 85.71% for IBMP-8.1 and 90.48% for IBMP-8.2–95.24% for IBMP-8.3 and 100% for IBMP-8.4, while specificity ranged from 99.98% for IBMP-8.3 and IBMP-8.4–100% for IBMP-8.1 and IBMP-8.2. Accuracy values ranged from 99.4 to 99.98%. The pretest probability for the molecules was 0.42, whereas the positive posttest probability ranged from 95.24 to 99.95% and the negative posttest probability ranged from 0.00001 to 0.0006% for all antigens. The higher odds ratio diagnosis was found for IBMP-8.4, which has been shown to be a safe single antigen for serological screening of CD in blood samples. The use of chimeric IBMP antigens is an alternative to reduce the number of bags discarded due to false-positive results. These molecules have high diagnostic performance and were shown to be suitable for use in screening CD in blood banks, isolated (IBMP-8.4) or in combination; and their use in blood banks could significantly reduce unnecessary disposal of blood bags or the risk of *T. cruzi* transmission.

## Introduction

Human Chagas disease (CD) or American trypanosomiasis is a life-threatening, neglected tropical parasitic disease caused by the hemoflagellate protozoan *Trypanosoma cruzi*. According to recent estimates, approximately 6 million people in 21 Latin American countries are affected by CD and 7,500 CD-associated deaths are reported annually (1). *T. cruzi* is usually transmitted through contact with feces/urine from infected bloodsucking triatomines that harbor the parasite in their intestines. Due to constant presence of the vector, 65 million people in these regions are at risk of infection (1). In addition, other routes of transmission such as blood transfusion, organ donation, consumption of contaminated food or beverages, and mother-to-child transmission represent increasingly important alternative routes of infection (2, 3). Since the late 1990s, demographic shifts and migration flows have fueled the spread of *T. cruzi*-infected individuals worldwide, particularly in non-endemic countries in North America, Europe, and Oceania (4–6). Due to the lack of universal donor screening to exclude CD in blood banks, transmission through contaminated blood transfusions accounts for nearly 20% of new cases annually worldwide (7).

Laboratory diagnosis of CD depends on the stage of the disease. In the acute phase, which lasts about 2 months and is usually asymptomatic, the parasites are easily detected in the blood of infected individuals by direct parasitological tests, molecular biology methods, xenodiagnosis, or blood cultures (8). The chronic phase begins 8–10 weeks after the acute phase and may last for several years or even the entire life of the host. Due to intermittent or low parasitemia with high anti-*T. cruzi* antibody levels, CD diagnosis in the chronic phase requires the use of antigen-antibody detection techniques using *in vitro* diagnostic (IVD) techniques. These include indirect immunofluorescence (IIF), indirect hemagglutination (IHA), rapid diagnostic tests (RTDs), enzyme-linked immunosorbent assays (ELISA), and chemiluminescence-based immunoassays (CLIA) (8–11). Since there is no precise standard assay for serologic diagnosis of chronic *T. cruzi* infection, WHO and PAHO recommend the simultaneous use of two serologic tests based on different methods (e.g., RTD and ELISA or IHA and IIF) and/or antigens (e.g., recombinant antigens and whole parasite lysate) to improve diagnosis consistency (12, 13). Therefore, test algorithms vary by location (endemic or non-endemic areas) and application (screening of blood/organ donors or diagnosis) (14–17).

In blood banks, serologic screening for anti-*T. cruzi* antibodies should be performed using a high-sensitivity IVD (18, 19), which can be achieved by using purified, recombinant, or synthetic peptides as antigens mainly in ELISA or CLIA diagnostic platforms. Commercial tests for screening CD should be able to identify *T. cruzi* antibodies regardless of genetic variability, endemicity, and cross-reactivity with other infectious and parasitic diseases. The major challenge for blood banks in serological screening CD is to reduce both the number of blood bags that are incorrectly discarded due to false-positive results and the costs associated to assays used in the screening.

The Brazilian Health Regulatory Agency (ANVISA) reported serological inability for donation in 0.34% of all collections performed in Brazil due to non-negative results for CD in 2013, 0.16% in 2014, 0.21% in 2015, 0.16% in 2016, 0.26% in 2017, 0.17% in 2018 (20), and 0.15% in 2019 (21). Due to this high number of non-negative (and discarded) blood bags, the serological tests used for screening in blood banks must have high accuracy and low cross-reactivity. The Brazilian Ministry of Health has adopted only one test with high sensitivity (22), e.g., ELISA or chemiluminescence, because it is a high-throughput automated method that can analyze a large number of samples daily. On the other hand, high analytical sensitivity leads to a greater number of false-positive results, resulting in emotional distress to donors and improper disposal of blood bags (23). In addition, the high degree of genetic polymorphism of the parasite may have a direct impact on the performance of the test depending on the geographic region where the screening tests are performed (24).

To overcome these obstacles, assays with higher specificity and sensitivity are required. This can be achieved by using chimeric recombinant proteins as antigenic matrices for immunoassays, consisting of conserved and repeating regions of multiple *T. cruzi* proteins in a single molecule (25–27). This strategy allows maintaining high performance rates even when the assay is used in geographic regions where different genetic strains of the parasite circulate (28–30). To this end, four chimeric recombinant proteins (IBMP-8.1, IBMP-8.2, IBMP-8.3, and IBMP-8.4) were genetically engineered and tested in phase I (25) and II (30) studies using ELISA, liquid microarray (31), immunochromatographic (11), and impedimetric immunosensor (32) assays. These studies were performed with panels of previously characterized samples from different endemic settings in several Latin American countries and in their immigrants living in Barcelona/Spain. High accuracy and low cross-reactivity rates have been observed in several infectious and parasitic diseases, including leishmaniasis (30, 33). In addition, all antigens have been shown to maintain their functional and structural stability under adverse conditions (34), making them robust and reliable candidates for future *in vitro* diagnostic assays that can be used for various models of point-of-care devices, including advanced biosensors. The performance of these antigens was evaluated using latent class analysis (LCA), a statistical tool used to evaluate new assays in the absence of a gold standard (35). Because the diagnostic potential of IBMP antigens has been extensively evaluated, the objective of this study was to evaluate the use of IBMP-8.1, IBMP-8.2, IBMP-8.3, and IBMP-8.4 chimeric *T. cruzi* antigens for serologic screening for Chagas disease in blood banks using a reference array of chimeric antigens as the gold standard.

## Materials and Methods

### Synthesis of Recombinant Chimeric Antigens

Synthetic genes encoding *T. cruzi*-chimeric antigens were obtained from a commercial supplier (GenScript, Piscataway, NJ, USA), subcloned into the pET28a vector, and expressed in *Escherichia coli* BL21-Star DE3 (Thermo Fisher Scientific). Cells were grown in Lysogenic broth supplemented with 0.5 mM isopropyl-β-D-1-thiogalactopyranoside (IPTG). *E. coli* lysates were prepared and His-labeled chimeric antigens were purified by affinity and ion exchange chromatography and then quantified using a fluorimetric assay (Qubit 2.0, Invitrogen Technologies, Carlsbad-CA, USA). Expression and purity of recombinant antigens were verified by SDS-PAGE (36). The plasmid constructs were described previously in Santos et al. (25). The antigenic composition of all four chimeric proteins is described in Table 1.

**Table 1:** Constitution of the IBMP chimeric recombinant antigens.

**Chimeric antigen**	**Sequence name**	**Amino acid range**	**Gene bank sequence ID**
IBMP-8.1	Trans-sialidase	747–774	XP_820062.1
	60S ribosomal protein L19	218–238	XP_820995.1
	Trans-sialidase	1435–1449	XP_813586.1
	Surface antigen 2 (CA-2)	276–297	XP_813516.1
IBMP-8.2	Antigen, partial	13–73	ACM47959.1
	Surface antigen 2 (CA-2)	166–220	XP_818927.1
	Calpain cysteine peptidase	31-97	XP_804989.1
IBMP-8.3	Trans-sialidase	710–754	XP_813237.1
	Flagellar repetitive antigen protein	15–56	AAA30177.1
	60S ribosomal protein L19	236–284	XP_808122.1
	Surface antigen 2 (CA-2)	279–315	XP_813516.1
IBMP-8.4	Shed-acute-phase-antigen	681–704	CAA40511.1
	Kinetoplastid membrane protein KMP-11	76–92	XP_810488.1
	Trans-sialidase	1436–1449	XP_813586.1
	Flagellar repetitive antigen protein	20–47	AAA30177.1
	Trans-sialidase	740–759	XP_820062.1
	Surface antigen 2 (CA-2)	276–298	XP_813516.1
	Flagellar repetitive antigen protein	1–68	AAA30197.1
	60S ribosomal protein L19	218–238	XP_820995.1
	Microtubule-associated protein	421–458	XP_809567.1

### Sample Collection

Samples were collected from volunteer blood donors at Hematology and Hemotherapy Foundation of the State of Bahia (HEMOBA Foundation) between December 2018 and August 2019 and stored in aliquots at −20°C. Because this is a prospective study (phase III), the results of screening tests performed by the HEMOBA Foundation for Chagas disease, syphilis, HIV-1/2, HTLV-1/2, hepatitis B (HBV), and hepatitis C (HCV), as well as the age, sex, and place of residence of blood donors, were kept confidential until the completion of the present study. The sample size was calculated with an expected sensitivity and specificity of 99%, an absolute error of 2%, a confidence interval of 95%, and a prevalence of chronic Chagas disease of 2% in the Brazilian population (37). Based on these parameters, the formula of Buderer (38) was used in the web version of the calculator (https://wnarifin.github.io/ssc/sssnsp.html) to estimate the minimum number of serum samples required to perform this study as 4,754. A total of 5,014 previously collected anonymized human serum samples were used to evaluate the individual performance of IBMP chimeras for *T. cruzi* by ELISA, using latent class analysis (LCA) as the reference test, as previously determined (33, 35, 39).

### Immunoassays (IBMP-ELISA)

Anti-*T. cruzi* serology was performed by ELISA as described previously (30). Assays were performed on transparent 96-well flat-bottom microplates (UV-Star® Microplate, Greiner Bio-One, Kremsmünster, Austria) coated with one of the chimeric IBMP antigens at concentrations of 12.5 ng (IBMP-8.2) or 25 ng (IBMP-8.1, IBMP-8.3, and IBMP-8.4) per well in coating buffer (0.05 M carbonate bicarbonate, pH 9.6). Coating and blocking were performed simultaneously with a synthetic buffer (WellChampion; Kem-En-Tec Diagnostics A/S, Taastrup, Denmark) according to the manufacturer's instructions. Serum samples were added to the coated wells diluted 1:100 in 0.05 M phosphate-buffered saline (PBS; pH 7.4), and the microtiter plates were incubated at 37 °C for 60 min. Thereafter, all wells were washed with PBS-0.05% Tween-20 (PBS-T; pH 7.4) to remove non-adsorbed material and incubated again at 37 °C for 30 min with 100 μl of HRP-conjugated goat anti-human IgG (Bio-Manguinhos, FIOCRUZ, Rio de Janeiro, Brazil) diluted 1:40,000 in PBS. After another wash cycle, 100 μl of TBM substrate (Kem-En-Tec Diagnostics A/S, Taastrup, Denmark) was added to the wells to detect the formation of immune complexes. Incubation was then performed for 10 min at room temperature in the dark. The colorimetric reactions were stopped by adding 50 μl of 0.3 M H_2_SO_4_ to each well. Optical density was determined in a SPECTRAmax 340PC microplate reader with a 450 nm filter (Molecular Devices, San Jose-CA, USA), and background values were subtracted from the measurement experiments.

### Latent Class Analysis as a Reference Test

**Figure 1:**
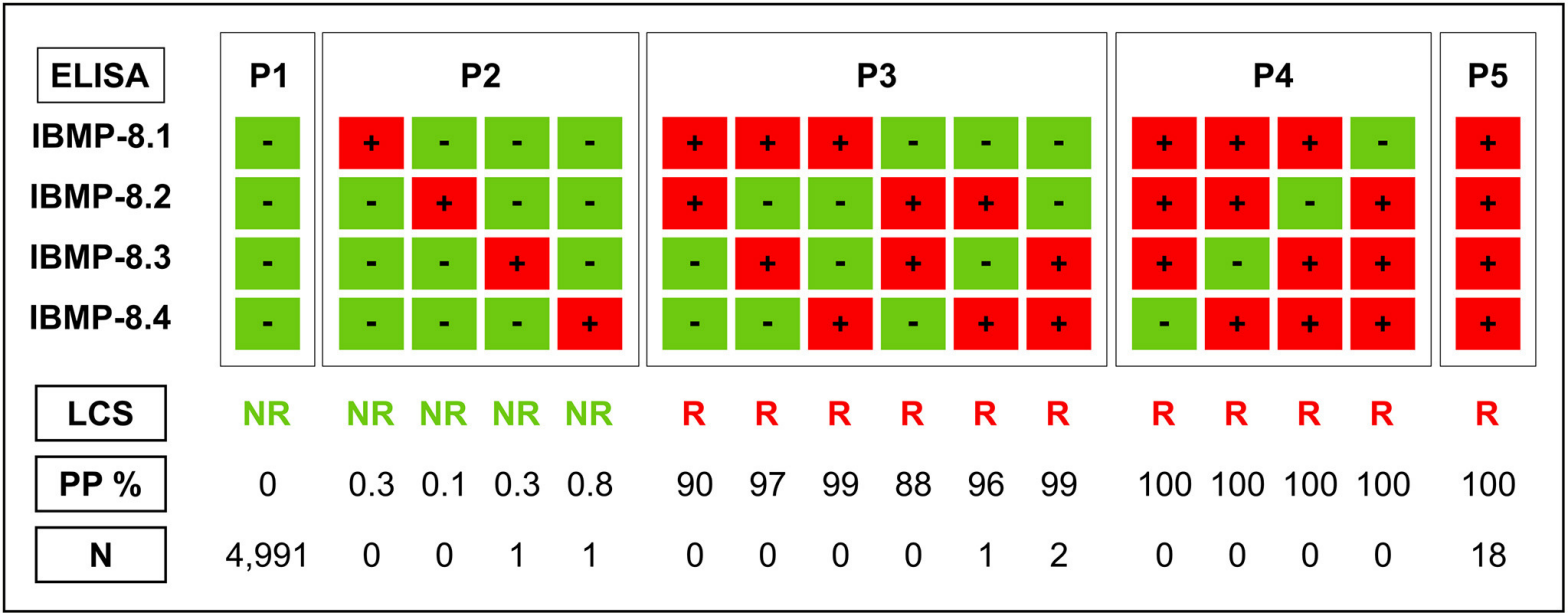
Response patterns of chimeric antigens in latent class analysis (LCA) used in anti-*T. cruzi* ELISA tests in HEMOBA Foundation blood donor volunteers between December 2018 and August 2019. LCS, latent class status; NR, non-reactive; PP, a posteriori probability; R, reactive; P1, P2, P3, P4, and P5, reaction response; N, number of samples.

Latent class analysis (LCA) was used for serological classification of *T. cruzi* as reactive or non-reactive for specific antibodies. This statistical model had been previously described and validated by our group in other studies (33, 35, 39). LCA is a multivariate statistical approach based on categorical indicators or latent variables. First, four indicators representing IBMP-8.1, IBMP-8.2, IBMP-8.3, and IBMP-8.4 were defined to characterize the latent variable that can correctly diagnose *T. cruzi* infection. Thus, the latent class response patterns defined a given sample as *T. cruzi* reactive if it showed positive results in at least two different chimera-based assays (*a posteriori* probability ranged from 87.9 to 100%). Conversely, a sample was considered non-reactive for *T. cruzi* if all four chimeric antigens gave a non-reactive result or if only one of the antigens was positive (*a posteriori* probability ranged from 0 to 0.8%) (Figure 1). A total of 16 response patterns were identified, which were divided into five categories (P1 to P5).

### Statistical Analysis

**Figure 2:**
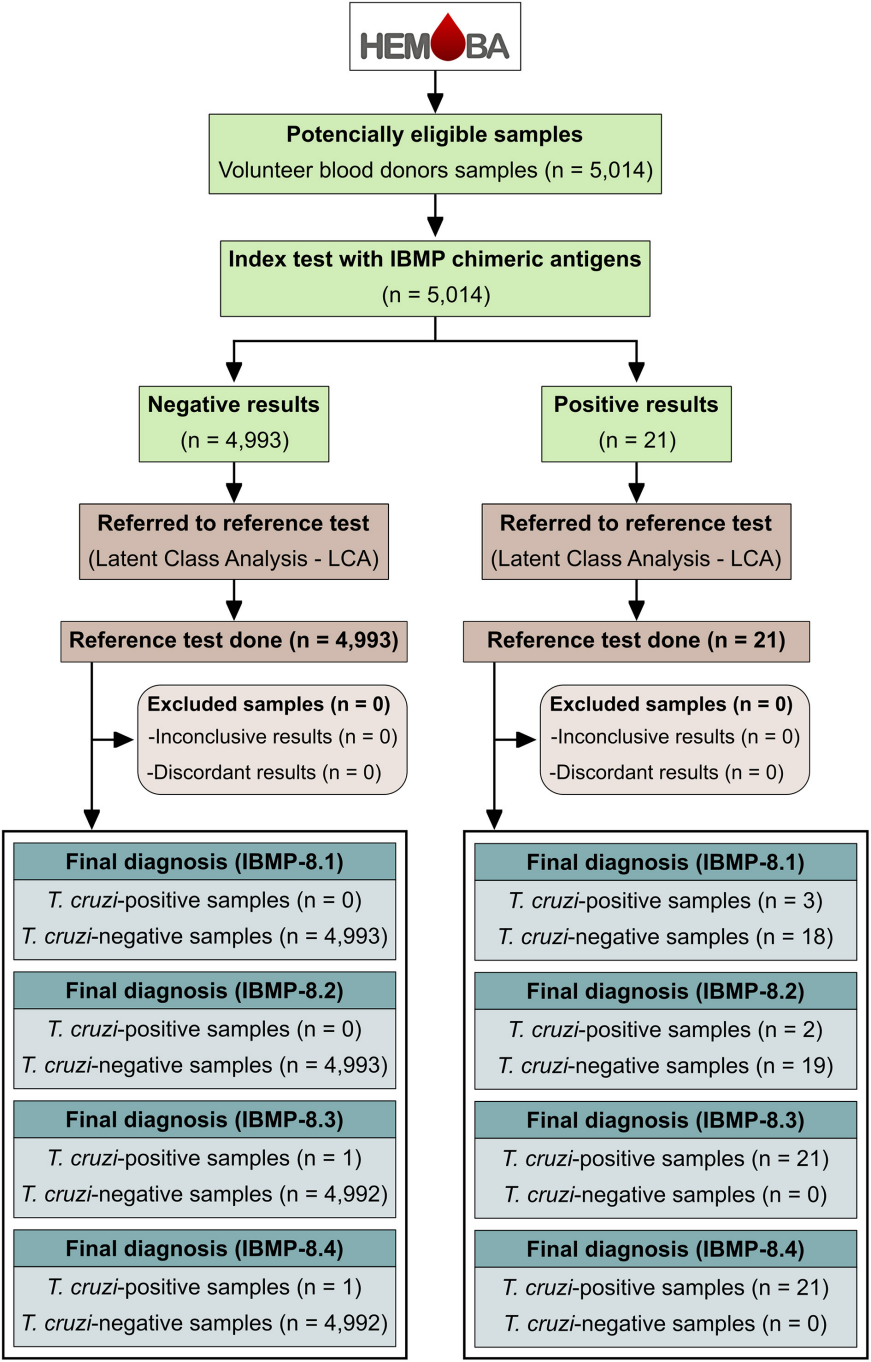
Flowchart depicting study design in accordance with the Standards for Reporting of Diagnostic Accuracy Studies (STARD) guidelines (46).

Data were analyzed with Scatterplot software (Prism, version 8; GraphPad, San Diego-CA, USA). Descriptive statistics are presented as geometric means ± standard deviations. The Shapiro-Wilk test followed by Student's *t*-test was used to test normality of the data sets. Wilcoxon's signed-rank test was used when the assumed homogeneity could not be confirmed. A significance level of 5% was assumed for all statistical tests (*p*-value < 0.05). Threshold (cut-off) analysis was used to determine the optimal optical density value (OD) to discriminate between *T. cruzi*-negative and positive blood bags. The threshold was determined by calculating the area under the ROC curve (AUC). The AUC values were also used to assess the global accuracy for each antigen, which could be classified as low (0.51–0.61), moderate (0.62–0.81), elevated (0.82–0.99), or outstanding (1.0) (40). The performance of ELISA-IBMP was calculated using a dichotomous approach (2 × 2 contingency table), and the performance characteristics of each IBMP protein were compared in terms of sensitivity, specificity, accuracy, likelihood ratio (LR), diagnostic odds ratio (DOR), predictive values, and post-test probabilities (41, 42). To better assess the diagnostic performance of the four IBMP chimeras, multiple testing (serial and parallel approaches) was applied to individual test characteristics. Multiple tests can be ordered simultaneously (parallel tests), in which case a positive result in any of the tests is evidence of disease, or they can be ordered sequentially (serial tests), as new tests are requested depending on the result of the previous test. In this case, all results must be positive to establish a diagnosis of disease. (43). Confidence intervals (CI) with a 95% confidence level (95% CI) were used, and the absence of overlapping 95% CI bars was used to derive statistical significance (44). The results were expressed as an index representing the ratio between the OD of the samples and the OD of the cut-off. This index is called the reactivity index (RI) and all results >1.00 were considered positive. Samples were considered inconclusive (or in the gray zone) if the RI values fell in the indeterminate zone, which was assumed to be RI values of 1.0 ± 10%. Statistical analysis of RIs was performed based on the absence of overlapping 95% CI. The strength of agreement between the results of the screening tests IBMP-ELISA and the result of LCA was assessed with the Cohen's kappa coefficient (κ) (45) interpreted as follows: poor (κ = 0), slight (0 < κ ≤ 0.20), fair (0.21 < κ ≤ 0.40), moderate (0.41 < κ ≤ 0.60), substantial (0.61 < κ ≤ 0.80), and almost perfect (0.81 < κ ≤ 1.0) agreement. The study workflow (Figure 2) was established according to STARD guidelines (46).

## Results

### Diagnostic Performance

A total of 5,014 previously collected, anonymized human serum samples were included in the study. The mean age of the population studied was 40.4 years [interquartile range (IQR): 28.3–58.2 years] and the female-to-male ratio was 0.75/1. At least one blood donation was performed in each microregion of Bahia. This represents 232 of 417 (55.6%) municipalities and a total population of 11,448,009 inhabitants (~77% of Bahia's population). Seventy-nine samples were from blood donors from the Federal District (*n* = 2) and other Brazilian states: Rio Grande do Norte (*n* = 1), Pernambuco (*n* = 58), Alagoas (*n* = 1), Minas Gerais (*n* = 5), Goiás (*n* = 2), Espírito Santo (*n* = 2), Rio de Janeiro (*n* = 1), São Paulo (*n* = 4), Paraná (*n* = 1), and Rio Grande do Sul (*n* = 2). Information on the geographic origin of the blood donors was missing in 26 samples.

All human sera were employed to evaluate the individual performance of IBMP chimeras for *T. cruzi* by ELISA using latent class analysis (LCA) as the reference test. LCA classified 4,993 samples (99.58%) as *T. cruzi*-negative, of which 4,991 and two samples were categorized as P1 (negative result for all four IBMP proteins) and P2 (negative result for three IBMP proteins), respectively. The remaining 21 samples (0.42%) were categorized as *T. cruzi*-positive: 18 were categorized as P5 (positive for all four IBMP proteins) and three as P3 (positive result for 2 IBMP proteins). Sociodemographic variables for all 21 *T. cruzi*-positive samples and reactivity indices for all four chimeric IBMP antigens are summarized in Table 2. For these samples, the mean age of *T. cruzi*-positive blood donors was 41.0 (IQR: 30.0–50.5 years) and the female-to-male ratio was 0.62/1.

**Table 2:** Data stratified by sociodemographic variables and reactivity indices for chimeric IBMP antigens from all 21 blood donors classified as *T. cruzi*-positive by latent class analysis.

**Sample ID**	**Sex**	**Age**	**Microregion**	**RI 8.1**	**RI 8.2**	**RI 8.3**	**RI 8.4**	**LCA**
3028	Male	52	Irecê	0.61	0.58	2.12	1.42	Pos
3295	Female	23	Jequié	1.46	1.38	1.22	1.93	Pos
4097	Male	27	Vitória da Conquista	0.32	0.29	1.99	2.60	Pos
4160	Male	49	Barreiras	1.74	1.84	1.33	1.44	Pos
4465	Female	40	Irecê	2.34	2.75	2.68	2.86	Pos
5231	Male	47	Barreiras	1.56	2.82	2.69	2.38	Pos
5617	Female	28	Salvador	1.59	2.78	2.21	2.20	Pos
5618	Male	39	Barreiras	0.44	1.14	0.79	1.15	Pos
5900	Male	29	Salvador	2.22	2.32	2.41	1.91	Pos
5901	Male	50	Sto Antônio de Jesus	2.55	2.65	2.93	2.46	Pos
5917	Male	41	Cotegipe	1.97	2.36	2.10	1.61	Pos
5918	Female	43	Feira de Santana	1.88	1.91	1.59	1.98	Pos
5936	Female	41	Salvador	1.69	1.29	1.14	1.81	Pos
6797	Female	30	Sto Antônio de Jesus	2.67	2.87	2.58	2.20	Pos
6802	Male	37	Cotegipe	1.54	1.41	2.00	1.31	Pos
6827	Male	41	Salvador	2.40	2.21	2.52	2.31	Pos
6840	Male	51	Salvador	3.35	3.71	2.80	2.68	Pos
6856	Male	51	Barreiras	2.73	2.48	2.02	2.33	Pos
6920	Female	63	Feira de Santana	2.89	1.93	3.24	2.67	Pos
7013	Male	30	Salvador	1.92	1.83	1.70	1.95	Pos
7087	Female	59	Brumado	2.28	2.21	1.58	2.07	Pos

*RI, Reactivity Index; Sto Antônio de Jesus, Santo Antônio de Jesus; Cut-off = 1.0*.

**Figure 3:**
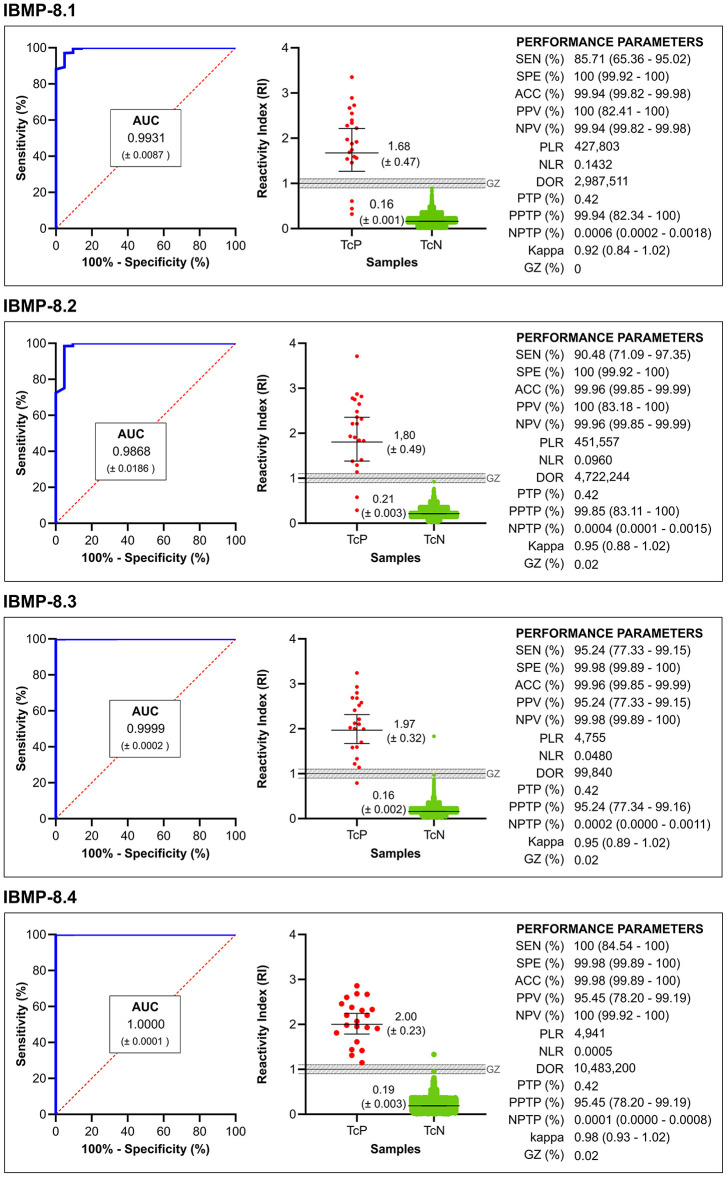
Graphical analysis of areas under the curve (AUC) ROC (left). Reactivity index (middle) obtained with serum samples from *Trypanosoma cruzi*-positive (TcP) and *Trypanosoma cruzi*-negative (TcN) samples. The cut-off value is the reactivity index = 1.0 and the shaded area represents the gray zone (RI = 1.0 ± 0.10). The horizontal lines and numbers for each group of results represent the geometric means (± 95% CI). Performance parameters (right) obtained for all chimeric IBMP proteins. SEN, sensitivity; SPE, specificity; ACC, accuracy; PPV, positive predictive value; NPV, negative predictive value; PLR, positive likelihood ratio; NLR, negative likelihood ratio; DOR, diagnostic odds ratio; PTP, pre-test probability; PPTP, positive post-test probability; NPTP, negative post-test probability; *Kappa*, cohen's *Kappa* coefficient; GR, gray zone.

Following the serological definition of 5,014 samples as *T. cruzi*-positive or *T. cruzi*-negative by LCA, the performance parameters of chimeric IBMP proteins were determined (Figure 3; individual data points are available in the Supplementary Table 1). Area under the curve (AUC) values were extremely high for all chimeric proteins, ranging from 98.68 (IBMP-8.2) to 100% (IBMP-8.4), demonstrating excellent overall diagnostic accuracy for all chimeric proteins. Considering a 95% confidence interval, all IBMP antigens showed similar performance. For *T. cruzi*-positive samples, IBMP-8.4 provided the highest RI (reactivity index) values, while IBMP-8.1 had the lowest RI distribution. No significant differences were observed between the RIs of all four IBMP proteins. *T. cruzi*-negative samples yielded low mean RI values among all four chimeric antigens tested (<0.22). Global RI analysis showed a significant difference between *T. cruzi*-positive and negative samples for all four proteins (*p* < 0.0001).

The diagnostic efficiency of antigens can also be assessed by the number of samples that fall into the gray zone. Considering a gray zone set as a cut-off value ± 10% (RI values of 1.0 ± 0.10), only one sample (0.02%) was found in the gray zone for the proteins IBMP-8.2 (*T. cruzi*-negative sample; sample ID 5245; RI 0.92), IBMP-8.3 (*T. cruzi*-negative sample; sample ID 7017; RI 0.97), and IBMP-8.4 (*T. cruzi*-negative sample; sample ID 6834; RI 0.92). No result was found in the gray zone when both *T. cruzi*-positive and *T. cruzi*-negative samples were tested with IBMP-8.1 antigen.

The IBMP-8.4 antigen yielded a sensitivity of 100%, followed by IBMP-8.3 (95.2%), IBMP-8.2 (90.5%), and IBMP-8.1 (87.7%). The differences in sensitivity were not statistically significant for the values obtained for all four proteins. The highest value for specificity was obtained for IBMP-8.1 and IBMP-8.2 proteins (100%), while IBMP-8.3 and IBMP-8.4 had values of 99.98%, with no differences between them. Regarding diagnostic accuracy, all chimeric proteins yielded values ≥ 99.94% (Figure 3). DOR Scores, based on positive and negative likelihood ratios, were 10,483,200 for IBMP-8.4, 4,722,244 for IBMP-8.2, 427,803 for IBMP-8.1, and 99,840 for IBMP-8.3. Qualitative assessment of the results showed near-perfect agreement between all chimeric IBMP proteins using the Cohens' kappa method (κ ≥ 0.92), with particular emphasis on IBMP-8.4 (κ = 0.98).

### Predictive Values

Because this was a phase 3 study, it was possible for the first time to determine the positive and negative predictive values of the IBMP proteins. The highest positive predictive value was obtained with IBMP-8.1 and IBMP-8.2 proteins (100%), followed by IBMP-8.4 (95.5%) and IBMP-8.3 (95.2%). All chimeric proteins yielded a negative predictive value >99.9%. Considering the 95% CI overlap, no statistical differences were observed in the positive and negative predictive values among the IBMP proteins. The pretest probability refers to the prevalence of the disease in the analyzed sample. It was estimated to be 0.42% of the samples regardless of the IBMP protein tested. IBMP-8.1 and IBMP-8.2 yielded the highest values for positive post-test probability: 99.94 and 99.85%, respectively. IBMP-8.3 provided the lowest value for positive post-test probability (95.24%), followed by IBMP-8.4 (99.45%). As for the negative post-test probability, all proteins yielded values ≤ 0.0006. At a confidence interval of 95%, all IBMP antigens showed similar positive and negative post-test probabilities. IBMP-8.4 offered the best performance among the chimeric recombinant proteins studied, as shown by the analysis of ROC and, most importantly, by the exceptionally high diagnostic odds ratio of this protein (DOR = 10,483,200; Figure 3).

### Diagnostic Performance of IBMP Pairs

In addition to individual performance, the performance of pairs of all four chimeric IBMP proteins was also estimated in serial and parallel approaches (Table 3). In the serial scheme, sensitivity ranged from 77.6 to 95.2%, whereas minimum specificity and negative predictive values reached 99.9%. Positive predictive values ranged from 90.9 to 100%. Conversely, sensitivity values ranged from 98.3 to 100% with a parallel scheme. Interestingly, no false-negative result was obtained when the positive samples were tested with IBMP-8.1/IBMP-8.4, IBMP-8.2/IBMP-8.3, IBMP-8.2/IBMP-8.4, and IBMP-8.3/IBMP-8.4 pairs. Regardless of the IBMP pairs analyzed, no false-positive result was obtained using a parallel approach. Positive and negative predictive values were 100% for all IBMP pairs, except for the positive predictive value when IBMP-8.3/IBMP-8.4 was analyzed (99.8%).

**Table 3:** Analysis of the diagnostic performance of the pair of chimeric IBMP proteins using serial and parallel approaches.

**Pair of tests**	**SEN**	**SPE**	**PPV**	**NPV**
**Series**	**% (95% CI)**	**% (95% CI)**	**% (95% CI)**	**% (95% CI)**
IBMP-8.1/IBMP-8.2	77.6 (46.5–92.5)	100 (99.8–100)	100 (68.6–100)	99.9 (99.7–100)
IBMP-8.1/IBMP-8.3	81.6 (50.6–94.2)	100 (99.8–100)	95.2 (63.7–99.2)	99.9 (99.7–100)
IBMP-8.1/IBMP-8.4	85.7 (55.3–95.0)	99.9 (99.8–100)	95.5 (64.4–99.2)	99.9 (99.7–100)
IBMP-8.2/IBMP-8.3	86.2 (55.0–96.5)	99.9 (99.8–100)	95.2 (64.3–99.2)	99.9 (99.7–100)
IBMP-8.2/IBMP-8.4	90.5 (60.1–97.4)	99.9 (99.8–100)	95.5 (65.1–99.2)	100 (99.9–100)
IBMP-8.3/IBMP-8.4	95.2 (65.4–99.2)	99.9 (99. 8–100)	90.9 (60.5–98.6)	99.9 (99.8–100)
**Parallel**	**% (95% CI)**	**% (95% CI)**	**% (95% CI)**	**% (95% CI)**
IBMP-8.1/IBMP-8.2	98.6 (90.0–99.9)	100 (99.9–100)	100 (97.0–100)	100 (99.9–100)
IBMP-8.1/IBMP-8.3	99.3 (92.2–99.9)	100 (99.9–100)	100 (96.0–100)	100 (99.9–100)
IBMP-8.1/IBMP-8.4	100 (94.7–100)	100 (99.9–100)	100 (96.2–100)	100 (99.9–100)
IBMP-8.2/IBMP-8.3	100 (93.5–100)	100 (99.9–100)	100 (96.2–100)	100 (99.9–100)
IBMP-8.2/IBMP-8.4	100 (95.5–100)	100 (99.9–100)	100 (96.3–100)	100 (99.9–100)
IBMP-8.3/IBMP-8.4	100 (96.5–100)	100 (99.9–100)	99.8 (95.1–99.9)	100 (99.9–100)

*CI, confidence interval; SEN, sensitivity; SPE, specificity; PPV, positive predictive value; NPV, negative predictive value*.

### Cross Reaction Analysis

**Figure 4:**
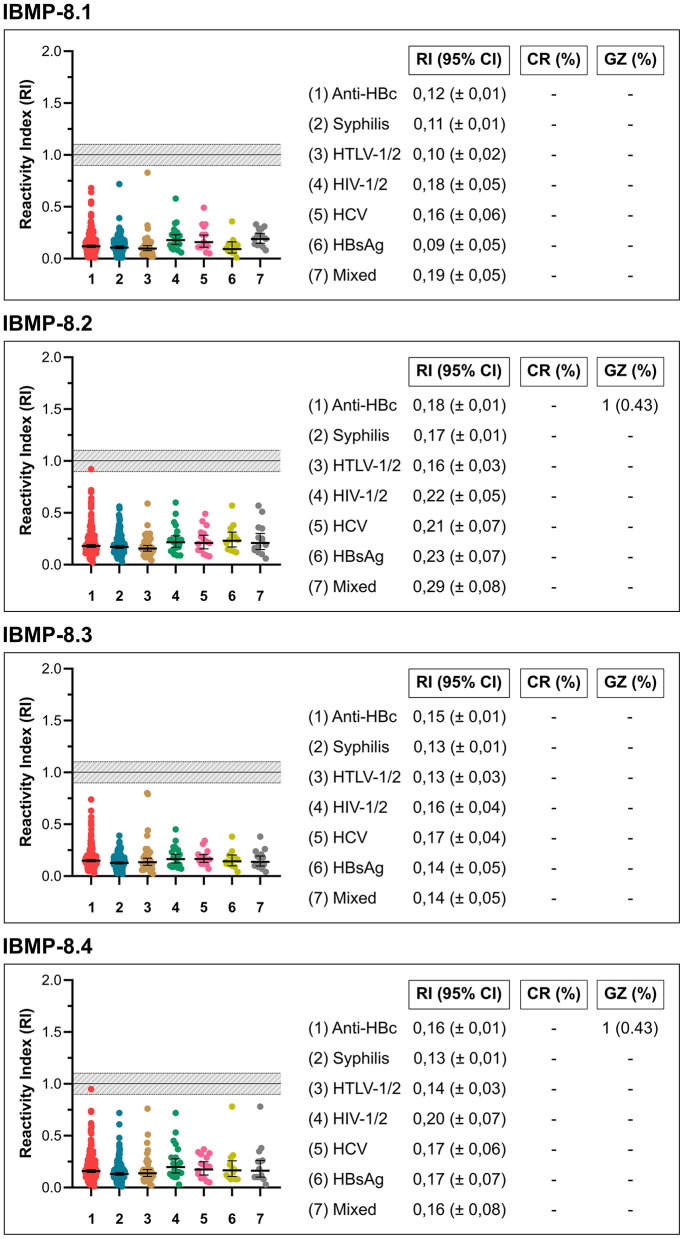
Graphical analysis of cross-reactivity with IBMP antigens using non-negative samples screened by HEMOBA Foundation. 95% CI, 95% confidence interval; IR, reactivity index.

According to the serologic screening performed by the HEMOBA Foundation with the 4,993 *T. cruzi*-negative sera in the present study, 233 samples tested positive for anti-HBc, 150 for syphilis, 37 for HTLV-1/2, 20 for HIV-1/2, 15 for HCV, and 12 for HBsAg. Mixed infections were detected in 14 sera: anti-HBc + syphilis (*n* = 4), anti-HBc + HBsAg (*n* = 2), anti-HBc + HBV (*n* = 2), HBsAg + HBV (*n* = 1), HTLV-1/2 + syphilis (*n* = 1), anti-HBc + HTLV-1/2 (*n* = 1), HIV-1/2 + syphilis (*n* = 1), HCV + syphilis (*n* = 1), and HTLV-1/2 + syphilis (*n* = 1). All these positive samples were used to evaluate the potential cross-reactivity (RI ≥ 1.0) of the IBMP chimeric proteins. As shown in Figure 4 (individual data points are available in the Supplementary Table 2), no cross-reactivity was found. In addition, only one sample that was anti-HBc positive (0.43%) was found to be inconclusive with the chimeric proteins IBMP-8.2 (sample ID 5245; RI 0.92) and IBMP-8.4 (sample ID 6834; RI 0.95). Among the 21 *T. cruzi*-positive samples, two were coinfected with HTLV-1/2, two with syphilis, one with both HCV and syphilis, and one was also positive for anti-HBc.

### IBMP Antigens for Blood Screening

**Figure 5:**
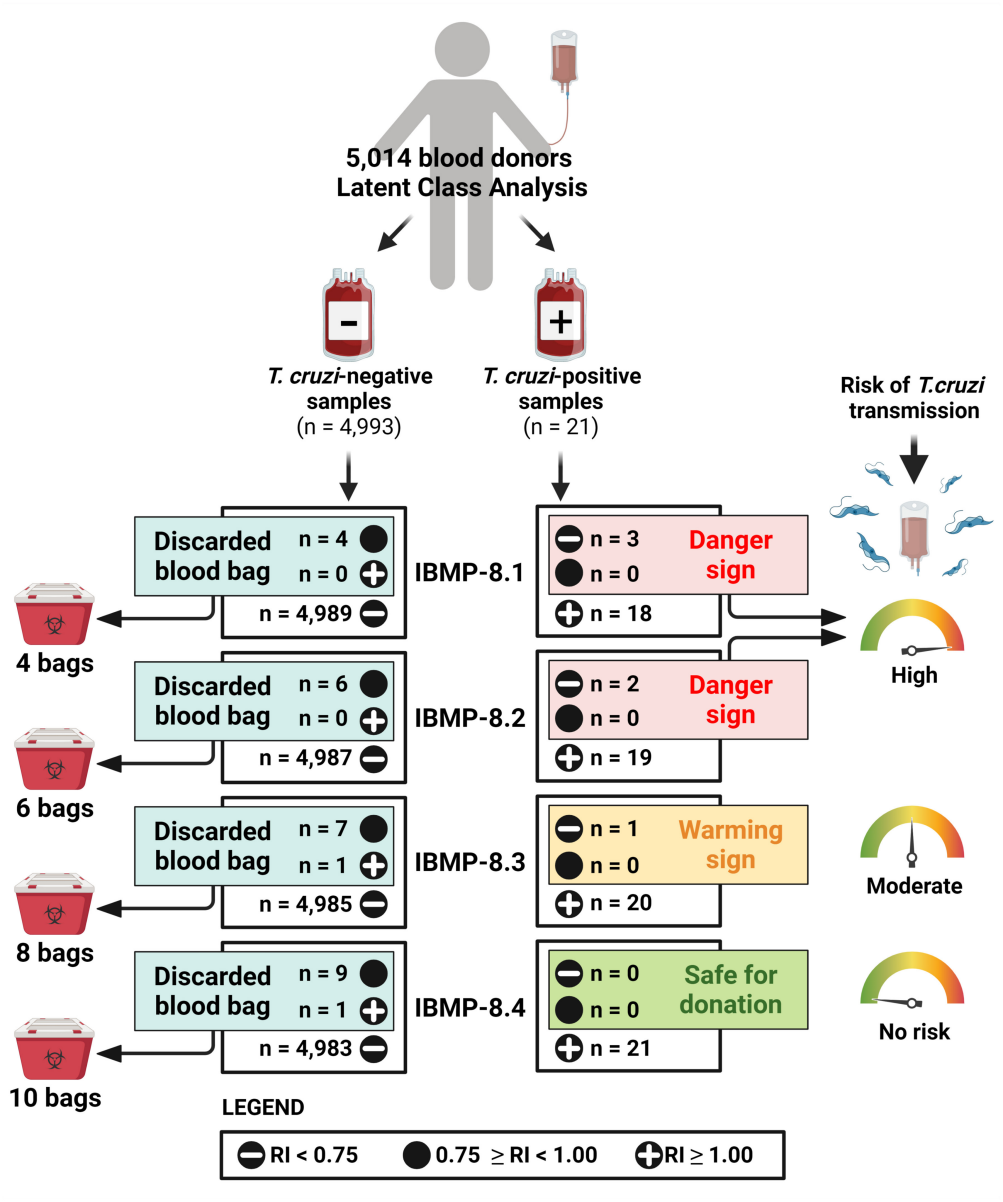
Analysis of individual use of chimeric IBMP proteins for CD serological. RI, reactivity index.

Individual use of chimeric IBMP proteins for CD serological screening was also analyzed (Figure 5). For this purpose, the criteria used by the HEMOBA Foundation were considered: (1) *T. cruzi*-positive samples: RI ≥ 1.00; (2) *T. cruzi*-negative samples: RI < 0.75; and (3) *T. cruzi*-inconclusive samples: 0.75 ≥ RI <1.00. Both *T. cruzi*-positive and *T. cruzi*-inconclusive samples are considered unsuitable for blood donation; therefore, the blood bags are discarded. Of the 4,993 *T. cruzi*-negative samples, IBMP-8.1, IBMP-8.2, IBMP-8.3, and IBMP-8.4 proteins classified four, six, seven, and nine blood bags, respectively, as *T. cruzi*-inconclusive samples, while IBMP-8.3 and IBMP-8.4 classified one sample each as *T. cruzi*-positive. Accordingly, screening with IBMP-8.4 would discard a total of ten *T. cruzi*-negative blood bags, followed by eight bags screened with IBMP-8.3, six bags screened with IBMP-8.2, and four bags screened with IBMP-8.1 protein. Conversely, all 21 *T. cruzi*-positive blood bags were correctly identified as positive when tested with IBMP-8.4 protein. One positive sample yielded an inconclusive result when tested with IBMP-8.3, which triggered a warning signal with a clear risk of *T. cruzi* transfusion transmission. A danger signal was triggered when IBMP-8.1 and IBMP-8.2 proteins gave a negative result in three and two bags, respectively, indicating a high risk of *T. cruzi* transmission.

## Discussion

In the present study, we evaluated the performance of the chimeric antigens for serological screening of CD and their potential use in blood banks. All four IBMP antigens showed high diagnostic capacity based on the AUC values found, which ranged from 98.68 to 100%, suggesting high discriminatory ability. These results are consistent with the phase I (11, 25, 32) and phase II (28–31) studies. Comparing the AUC values found here with those reported in the literature for other antigens, IBMP antigens showed higher values than those reported for mixtures of different synthetic epitopes (47), multiepitope antigens (48) and assays such as the Abbott Chagas Elisa (49). Conversely, some multiepitope antigens, such as CP1, CP3, and CP1 + CP3, showed similar AUC values to IBMP antigens (50).

The antigen IBMP-8.4 had the highest sensitivity value in the present study as well as in the previously performed studies, regardless of the population studied and the methodology used. This is due to the nature of the molecule, as it comprises a larger repertoire of epitopes compared to the others, making it responsive to a greater diversity of anti-*T. cruzi* antibodies (25, 30, 34, 35). In contrast to the IBMP-8.4 protein, identification of anti-*T. cruzi* was less efficient in blood donors with IBMP-8.1, probably due to its limited repertoire of antigens. This result contrasts with the results of other studies that have used this molecule as an antigenic matrix. In the sensitivity analysis, 21 samples were predicted by LCA to be positive. This small number of positive samples has a strong influence on the determination of sensitivity, since each false-negative result corresponds to a 4.76% reduction in the sensitivity value. The opposite is true for specificity, where a single false-positive sample would reduce the value by only 0.02%. Considering that this is a phase III study (blind study), it was not possible to control the sample size of each group (positive and negative). Nevertheless, the accuracy values for all antigens were ≥99.4%, thanks to the large number of negative samples and the high specificity of all antigens (99.98 to 100%).

Among the 21 *T. cruzi*-samples specimens positive at LCA classification, six were positive for coinfection with other diseases: HTLV, syphilis, HBV, and HCV. HTLV, HCV, and syphilis have similar characteristics: they are sexually transmitted, endemic, and considered a public health problem in Brazil. Their specific mode of transmission facilitates coinfection with other diseases (51–53). On the other hand, the prevalence rate for HBV in Brazil is low, most likely due to vaccination, which has been available for more than 20 years (54, 55), although there are still unvaccinated individuals at risk. HBV is transmitted through blood (parenterally and vertically), sexually, and by sharing contaminated objects (55), so it can be said that coinfections between HTLV, HCV, syphilis, and HBV are common because of their routes of transmission. In our study, there was no cross-reactivity to any of the four IBMP antigens. Only two samples were classified in the inconclusive zone (RI ± 10%) when analyzed with IBMP-8.2 and IBMP-8.4 antigens. These results confirm previous studies performed with the molecules for various infectious and parasitic diseases, such as dengue, HBV, HCV, HIV, HTLV, leishmaniasis, schistosomiasis, filariasis, leptospirosis, measles, rubella, and syphilis (30, 33).

Recommendations for serologic screening in blood banks vary according to the CD endemic area. In endemic countries, screening should be performed with a high-sensitivity IVD (18, 19), whereas in non-endemic countries, screening should take into account that (1) all donors with a history of CD should be permanently deferred; (2) if screening tests for CD are not available, all donors with a recognized risk for CD should be identified and permanently deferred; and (3) if screening tests for CD are available, all donors with an identified risk for CD should initially be deferred for 6 months after their last return from an endemic area. Their subsequent donations should then be screened for signs of infection using a highly sensitive IVD (18). In recent years, purified, recombinant, or synthetic peptide antigens have been used as solid phase in IVD for detection of anti-*T. cruzi* antibodies with acceptable sensitivity values for safe serological CD screening in blood banks.

After analyzing the performance parameters of the four all IBMP antigens, an analysis of the individual use of each molecule in serological screening for CD. Normally, according to safety criteria, blood banks set the cutoff point at 20 to 25% of the manufacturer's specified value to reduce the possibility of transmission of bloodborne pathogens as much as possible; HEMOBA Foundation lowers the cutoff value to 25%. Considering these cut-off values, four, six, seven, and nine bags were rejected as false positive for IBMP-8.1, IBMP-8.2, IBMP-8.3, and IBMP-8.4 antigens, respectively. The antigens IBMP-8.1 and IBMP-8.2 detected as false-negative four and two bags, respectively, indicating that their use alone in serological screening of CD in blood banks is not recommended because of the possibility of transmission during transfusion. However, the combined use of these molecules is safe because the false-negative or exclusion zone results are not the same when the four assays are compensated. Despite the greater number of bags discarded when the IBMP-8.4 molecule was used, this was the safest molecule to be used alone in serological screening in blood banks.

The disposal of 29 negative bags harms public health, not only because of the resources invested in collection, donor pickup, and serologic screening, but also because of the indirect costs of reducing the supply of blood available for transfusion. Overall, the prevalence of CD in Brazil is estimated at ~2.16% (4.6 million people) (37), which is considered low. Therefore, a test with a specificity of <98.5% would lead to more false-positive than true-positive results (56). This gap could be easily closed by using the four antigens, especially with IBMP-8.1 and IBMP-8.2, since they have a specificity of 100%. The combined use of molecules, e.g., IBMP-8.4 in the first stage of diagnosis, would eliminate all false-negative results, then IBMP-8.1 or IBMP-8.2 can be used to exclude false-positive results. In this way, we would have a more effective and safer diagnosis, which would result in a lower number of blood bag disposals and reduce the cost of monitoring blood quality for transfusions.

In summary, this was a phase III study evaluating the four chimeric recombinant antigens IBMP-8.1, IBMP-8.2, IBMP-8.3, and IBMP-8.4 for serological diagnosis of chronic Chagas disease. The molecules exhibited high diagnostic performance and were shown to be suitable for screening CD in blood banks, isolated (IBMP-8.4) or in combination. Their use in blood banks could significantly reduce unnecessary disposal of blood bags or the risk of *T. cruzi* transmission.

## Data Availability Statement

The original contributions presented in the study are included in the article/Supplementary Material, further inquiries can be directed to the corresponding author.

## Ethics Statement

The studies involving human participants were reviewed and approved by Institutional Review Board (IRB) for Human Research at the Gonçalo Moniz Institute, Oswaldo Cruz Foundation, Salvador, Bahia (BA), Brazil (CAAE 67809417.0.0000.0040). Written informed consent for participation was not required for this study in accordance with the national legislation and the institutional requirements.

## Author Contributions

FS, MR, PC, MK, and NZ designed the experimental procedure. ES, ÂS, NF, LL, and RD performed the data acquisition, analysis, and interpretation. CS and MA determined the sample size. PC expressed and purified the chimeric recombinant antigens. ES performed the ELISA experiments. ES and FS wrote the paper. ÂS, NF, LL, and RD helped write the article. FS prepared the figures and supervised the work. FS, FA, MR, MK, and NZ provided laboratory space. FS, MR, MK, and NZ obtained funding for this study. All authors contributed substantially to the work described in this article, read, and agreed to the published version of the manuscript.

## Funding

This research was supported by the Coordination of Superior Level Staff Improvement-Brazil (CAPES; Finance Code 001), the Research Support Foundation of the State of Bahia (FAPESB); and Inova Fiocruz/VPPCB (Grant Number VPPCB-008-FIO-18-2-20). MK, MR, and FS are CNPq research fellows (Grant Numbers 590032/2011-9, 307319/2016-4, and 309263/2020-4, respectively). Funders had no influence on the study design, data collection and analysis, decision to publish, or preparation of the manuscript.

## Conflict of Interest

The authors declare that the research was conducted in the absence of any commercial or financial relationships that could be construed as a potential conflict of interest.

## Publisher's Note

All claims expressed in this article are solely those of the authors and do not necessarily represent those of their affiliated organizations, or those of the publisher, the editors and the reviewers. Any product that may be evaluated in this article, or claim that may be made by its manufacturer, is not guaranteed or endorsed by the publisher.
