# Supplementary material for: Performance of Chimeric Trypanosoma cruzi Antigens in Serological Screening for Chagas Disease in Blood Banks
Source: Front Med (Lausanne). 2022 Mar 7;9:852864. doi: 10.3389/fmed.2022.852864 (PMC8940225; doi:10.3389/fmed.2022.852864)
Supplement: Supplementary file 1 [file Table_1.pdf]

Supplementary Material

Table S1. Reactivity Index for diagnostic performance assessment.

Samples	REACTIVITY INDEX			
	IBMP-8.1	IBMP-8.2	IBMP-8.3	IBMP-8.4
T. cruzi-positive_1	0.61	0.58	2.12	1.42
T. cruzi-positive_2	1.46	1.38	1.22	1.93
T. cruzi-positive_3	0.32	0.29	1.99	2.60
T. cruzi-positive_4	1.74	1.84	1.33	1.44
T. cruzi-positive_5	2.34	2.75	2.68	2.86
T. cruzi-positive_6	1.56	2.82	2.69	2.38
T. cruzi-positive_7	1.59	2.78	2.21	2.20
T. cruzi-positive_8	0.44	1.14	0.79	1.15
T. cruzi-positive_9	2.22	2.32	2.41	1.91
T. cruzi-positive_10	2.55	2.65	2.93	2.46
T. cruzi-positive_11	1.97	2.36	2.10	1.61
T. cruzi-positive_12	1.88	1.91	1.59	1.98
T. cruzi-positive_13	1.69	1.29	1.14	1.81
T. cruzi-positive_14	2.67	2.87	2.58	2.20
T. cruzi-positive_15	1.54	1.41	2.00	1.31
T. cruzi-positive_16	2.40	2.21	2.52	2.31
T. cruzi-positive_17	3.35	3.71	2.80	2.68
T. cruzi-positive_18	2.73	2.48	2.02	2.33
T. cruzi-positive_19	2.89	1.93	3.24	2.67
T. cruzi-positive_20	1.92	1.83	1.70	1.95
T. cruzi-positive_21	2.28	2.21	1.58	2.07
T. cruzi-negative_1	0.18	0.28	0.18	0.12
T. cruzi-negative_2	0.16	0.21	0.21	0.16
T. cruzi-negative_3	0.15	0.19	0.16	0.14
T. cruzi-negative_4	0.19	0.24	0.27	0.21
T. cruzi-negative_5	0.27	0.25	0.36	0.34
T. cruzi-negative_6	0.30	0.31	0.24	0.19
T. cruzi-negative_7	0.22	0.26	0.22	0.08
T. cruzi-negative_8	0.23	0.26	0.24	0.16
T. cruzi-negative_9	0.13	0.16	0.34	0.08
T. cruzi-negative_10	0.20	0.18	0.15	0.11
T. cruzi-negative_11	0.24	0.26	0.21	0.27
T. cruzi-negative_12	0.19	0.16	0.16	0.13

*T. cruz*i-negative_13	0.23	0.34	0.28	0.20
*T. cruz*i-negative_14	0.32	0.35	0.25	0.22
*T. cruz*i-negative_15	0.29	0.33	0.16	0.15
*T. cruz*i-negative_16	0.42	0.23	0.20	0.31
*T. cruz*i-negative_17	0.31	0.38	0.33	0.18
*T. cruz*i-negative_18	0.30	0.26	0.17	0.34
*T. cruz*i-negative_19	0.16	0.17	0.16	0.24
*T. cruz*i-negative_20	0.26	0.23	0.21	0.25
*T. cruz*i-negative_21	0.18	0.24	0.22	0.13
*T. cruz*i-negative_22	0.38	0.68	0.33	0.23
*T. cruz*i-negative_23	0.36	0.37	0.21	0.30
*T. cruz*i-negative_24	0.37	0.31	0.21	0.13
*T. cruz*i-negative_25	0.17	0.19	0.15	0.13
*T. cruz*i-negative_26	0.31	0.38	0.27	0.22
*T. cruz*i-negative_27	0.56	0.28	0.06	0.28
*T. cruz*i-negative_28	0.25	0.43	0.31	0.23
*T. cruz*i-negative_29	0.27	0.71	0.25	0.20
*T. cruz*i-negative_30	0.15	0.17	0.11	0.11
*T. cruz*i-negative_31	0.32	0.38	0.21	0.22
*T. cruz*i-negative_32	0.19	0.19	0.34	0.44
*T. cruz*i-negative_33	0.38	0.27	0.27	0.17
*T. cruz*i-negative_34	0.30	0.27	0.17	0.29
*T. cruz*i-negative_35	0.34	0.54	0.06	0.24
*T. cruz*i-negative_36	0.18	0.19	0.22	0.40
*T. cruz*i-negative_37	0.15	0.16	0.11	0.10
*T. cruz*i-negative_38	0.32	0.46	0.27	0.24
*T. cruz*i-negative_39	0.32	0.40	0.17	0.21
*T. cruz*i-negative_40	0.30	0.42	0.23	0.21
*T. cruz*i-negative_41	0.50	0.66	0.48	0.57
*T. cruz*i-negative_42	0.45	0.36	0.23	0.25
*T. cruz*i-negative_43	0.53	0.52	0.05	0.30
*T. cruz*i-negative_44	0.22	0.33	0.20	0.17
*T. cruz*i-negative_45	0.30	0.37	0.22	0.20
*T. cruz*i-negative_46	0.36	0.37	0.24	0.19
*T. cruz*i-negative_47	0.36	0.37	0.23	0.14
*T. cruz*i-negative_48	0.31	0.35	0.30	0.34
*T. cruz*i-negative_49	0.35	0.29	0.27	0.19
*T. cruz*i-negative_50	0.37	0.33	0.19	0.34
*T. cruz*i-negative_51	0.32	0.39	0.18	0.19
*T. cruz*i-negative_52	0.14	0.17	0.07	0.12
*T. cruz*i-negative_53	0.19	0.23	0.08	0.10
*T. cruz*i-negative_54	0.19	0.24	0.15	0.12

*T. cruz*i-negative_55	0.24	0.23	0.18	0.08
*T. cruz*i-negative_56	0.16	0.27	0.13	0.10
*T. cruz*i-negative_57	0.15	0.25	0.08	0.14
*T. cruz*i-negative_58	0.27	0.23	0.10	0.13
*T. cruz*i-negative_59	0.16	0.17	0.09	0.10
*T. cruz*i-negative_60	0.29	0.23	0.08	0.09
*T. cruz*i-negative_61	0.22	0.16	0.13	0.17
*T. cruz*i-negative_62	0.22	0.22	0.12	0.10
*T. cruz*i-negative_63	0.23	0.33	0.26	0.23
*T. cruz*i-negative_64	0.17	0.25	0.22	0.11
*T. cruz*i-negative_65	0.11	0.16	0.19	0.07
*T. cruz*i-negative_66	0.38	0.22	0.08	0.29
*T. cruz*i-negative_67	0.24	0.25	0.09	0.13
*T. cruz*i-negative_68	0.28	0.26	0.15	0.12
*T. cruz*i-negative_69	0.33	0.18	0.23	0.24
*T. cruz*i-negative_70	0.16	0.15	0.11	0.10
*T. cruz*i-negative_71	0.19	0.19	0.06	0.08
*T. cruz*i-negative_72	0.03	0.13	0.11	0.07
*T. cruz*i-negative_73	0.14	0.25	0.13	0.11
*T. cruz*i-negative_74	0.28	0.25	0.20	0.22
*T. cruz*i-negative_75	0.13	0.16	0.08	0.08
*T. cruz*i-negative_76	0.20	0.35	0.09	0.12
*T. cruz*i-negative_77	0.13	0.19	0.05	0.13
*T. cruz*i-negative_78	0.14	0.29	0.07	0.08
*T. cruz*i-negative_79	0.20	0.22	0.13	0.79
*T. cruz*i-negative_80	0.12	0.21	0.08	0.08
*T. cruz*i-negative_81	0.11	0.24	0.05	0.08
*T. cruz*i-negative_82	0.14	0.15	0.09	0.09
*T. cruz*i-negative_83	0.18	0.16	0.09	0.15
*T. cruz*i-negative_84	0.10	0.16	0.05	0.07
*T. cruz*i-negative_85	0.31	0.10	0.16	0.21
*T. cruz*i-negative_86	0.01	0.12	0.22	0.26
*T. cruz*i-negative_87	0.20	0.19	0.09	0.12
*T. cruz*i-negative_88	0.01	0.23	0.11	0.18
*T. cruz*i-negative_89	0.19	0.25	0.11	0.12
*T. cruz*i-negative_90	0.14	0.43	0.06	0.10
*T. cruz*i-negative_91	0.11	0.15	0.02	0.06
*T. cruz*i-negative_92	0.09	0.22	0.05	0.08
*T. cruz*i-negative_93	0.13	0.22	0.03	0.01
*T. cruz*i-negative_94	0.01	0.14	0.06	0.10
*T. cruz*i-negative_95	0.14	0.25	0.10	0.15
*T. cruz*i-negative_96	0.29	0.21	0.09	0.09
*T. cruz*i-negative_97	0.15	0.35	0.11	0.14

*T. cruz*i-negative_98	0.12	0.16	0.05	0.10
*T. cruz*i-negative_99	0.20	0.29	0.07	0.09
*T. cruz*i-negative_100	0.04	0.18	0.05	0.06
*T. cruz*i-negative_101	0.05	0.16	0.04	0.07
*T. cruz*i-negative_102	0.17	0.26	0.07	0.11
*T. cruz*i-negative_103	0.09	0.19	0.16	0.20
*T. cruz*i-negative_104	0.21	0.26	0.37	0.17
*T. cruz*i-negative_105	0.11	0.15	0.16	0.30
*T. cruz*i-negative_106	0.22	0.30	0.16	0.19
*T. cruz*i-negative_107	0.22	0.31	0.17	0.15
*T. cruz*i-negative_108	0.16	0.21	0.13	0.15
*T. cruz*i-negative_109	0.28	0.26	0.21	0.14
*T. cruz*i-negative_110	0.18	0.30	0.15	0.11
*T. cruz*i-negative_111	0.36	0.21	0.23	0.26
*T. cruz*i-negative_112	0.40	0.28	0.31	0.23
*T. cruz*i-negative_113	0.25	0.27	0.18	0.15
*T. cruz*i-negative_114	0.32	0.34	0.23	0.22
*T. cruz*i-negative_115	0.26	0.29	0.28	0.19
*T. cruz*i-negative_116	0.20	0.15	0.17	0.13
*T. cruz*i-negative_117	0.31	0.26	0.20	0.15
*T. cruz*i-negative_118	0.25	0.29	0.26	0.12
*T. cruz*i-negative_119	0.30	0.25	0.25	0.13
*T. cruz*i-negative_120	0.25	0.14	0.19	0.13
*T. cruz*i-negative_121	0.22	0.33	0.15	0.15
*T. cruz*i-negative_122	0.26	0.40	0.23	0.22
*T. cruz*i-negative_123	0.21	0.09	0.26	0.15
*T. cruz*i-negative_124	0.18	0.34	0.32	0.11
*T. cruz*i-negative_125	0.33	0.19	0.24	0.15
*T. cruz*i-negative_126	0.31	0.27	0.23	0.15
*T. cruz*i-negative_127	0.36	0.16	0.36	0.12
*T. cruz*i-negative_128	0.75	0.20	0.41	0.23
*T. cruz*i-negative_129	0.57	0.18	0.33	0.23
*T. cruz*i-negative_130	0.39	0.24	0.39	0.26
*T. cruz*i-negative_131	0.31	0.14	0.36	0.17
*T. cruz*i-negative_132	0.29	0.17	0.46	0.15
*T. cruz*i-negative_133	0.37	0.20	0.24	0.13
*T. cruz*i-negative_134	0.28	0.19	0.20	0.10
*T. cruz*i-negative_135	0.20	0.23	0.17	0.18
*T. cruz*i-negative_136	0.18	0.17	0.16	0.14
*T. cruz*i-negative_137	0.20	0.19	0.14	0.13
*T. cruz*i-negative_138	0.15	0.19	0.12	0.14
*T. cruz*i-negative_139	0.19	0.16	0.10	0.11

*T. cruz*i-negative_140	0.12	0.19	0.11	0.10
*T. cruz*i-negative_141	0.26	0.46	0.20	0.20
*T. cruz*i-negative_142	0.18	0.24	0.15	0.20
*T. cruz*i-negative_143	0.19	0.26	0.15	0.14
*T. cruz*i-negative_144	0.22	0.29	0.15	0.17
*T. cruz*i-negative_145	0.20	0.24	0.12	0.11
*T. cruz*i-negative_146	0.14	0.17	0.11	0.11
*T. cruz*i-negative_147	0.16	0.18	0.12	0.15
*T. cruz*i-negative_148	0.17	0.21	0.15	0.14
*T. cruz*i-negative_149	0.09	0.16	0.08	0.14
*T. cruz*i-negative_150	0.22	0.25	0.14	0.17
*T. cruz*i-negative_151	0.17	0.24	0.11	0.12
*T. cruz*i-negative_152	0.17	0.22	0.14	0.17
*T. cruz*i-negative_153	0.20	0.26	0.19	0.17
*T. cruz*i-negative_154	0.21	0.21	0.16	0.17
*T. cruz*i-negative_155	0.24	0.29	0.19	0.18
*T. cruz*i-negative_156	0.10	0.15	0.09	0.10
*T. cruz*i-negative_157	0.27	0.32	0.17	0.19
*T. cruz*i-negative_158	0.18	0.22	0.11	0.13
*T. cruz*i-negative_159	0.13	0.15	0.09	0.09
*T. cruz*i-negative_160	0.17	0.19	0.10	0.18
*T. cruz*i-negative_161	0.24	0.16	0.09	0.21
*T. cruz*i-negative_162	0.11	0.12	0.05	0.08
*T. cruz*i-negative_163	0.16	0.12	0.10	0.12
*T. cruz*i-negative_164	0.17	0.23	0.14	0.12
*T. cruz*i-negative_165	0.20	0.28	0.14	0.17
*T. cruz*i-negative_166	0.17	0.16	0.08	0.10
*T. cruz*i-negative_167	0.21	0.19	0.14	0.17
*T. cruz*i-negative_168	0.23	0.20	0.08	0.18
*T. cruz*i-negative_169	0.25	0.25	0.19	0.23
*T. cruz*i-negative_170	0.16	0.16	0.13	0.14
*T. cruz*i-negative_171	0.26	0.29	0.21	0.18
*T. cruz*i-negative_172	0.24	0.28	0.23	0.23
*T. cruz*i-negative_173	0.21	0.32	0.15	0.22
*T. cruz*i-negative_174	0.15	0.15	0.10	0.10
*T. cruz*i-negative_175	0.15	0.17	0.09	0.13
*T. cruz*i-negative_176	0.25	0.31	0.21	0.15
*T. cruz*i-negative_177	0.20	0.19	0.12	0.12
*T. cruz*i-negative_178	0.18	0.21	0.15	0.15
*T. cruz*i-negative_179	0.20	0.26	0.14	0.14
*T. cruz*i-negative_180	0.10	0.17	0.11	0.10
*T. cruz*i-negative_181	0.19	0.22	0.13	0.23
*T. cruz*i-negative_182	0.15	0.16	0.14	0.14

T. cruzi-negative_183	0.25	0.25	0.19	0.20
T. cruzi-negative_184	0.23	0.20	0.16	0.15
T. cruzi-negative_185	0.19	0.15	0.15	0.16
T. cruzi-negative_186	0.25	0.22	0.18	0.19
T. cruzi-negative_187	0.14	0.13	0.09	0.13
T. cruzi-negative_188	0.16	0.22	0.16	0.14
T. cruzi-negative_189	0.20	0.27	0.20	0.16
T. cruzi-negative_190	0.20	0.18	0.15	0.13
T. cruzi-negative_191	0.22	0.27	0.20	0.19
T. cruzi-negative_192	0.20	0.29	0.29	0.17
T. cruzi-negative_193	0.25	0.22	0.17	0.18
T. cruzi-negative_194	0.15	0.19	0.12	0.14
T. cruzi-negative_195	0.21	0.20	0.13	0.15
T. cruzi-negative_196	0.21	0.22	0.18	0.19
T. cruzi-negative_197	0.40	0.36	0.32	0.34
T. cruzi-negative_198	0.20	0.19	0.15	0.17
T. cruzi-negative_199	0.16	0.26	0.15	0.14
T. cruzi-negative_200	0.22	0.27	0.17	0.19
T. cruzi-negative_201	0.15	0.19	0.12	0.14
T. cruzi-negative_202	0.16	0.22	0.13	0.15
T. cruzi-negative_203	0.32	0.35	0.26	0.21
T. cruzi-negative_204	0.20	0.17	0.16	0.23
T. cruzi-negative_205	0.26	0.21	0.18	0.19
T. cruzi-negative_206	0.18	0.19	0.16	0.15
T. cruzi-negative_207	0.41	0.34	0.35	0.34
T. cruzi-negative_208	0.37	0.32	0.22	0.23
T. cruzi-negative_209	0.33	0.33	0.23	0.28
T. cruzi-negative_210	0.37	0.32	0.29	0.25
T. cruzi-negative_211	0.24	0.21	0.15	0.18
T. cruzi-negative_212	0.23	0.24	0.17	0.17
T. cruzi-negative_213	0.20	0.17	0.15	0.16
T. cruzi-negative_214	0.32	0.27	0.24	0.25
T. cruzi-negative_215	0.24	0.22	0.17	0.18
T. cruzi-negative_216	0.35	0.39	0.21	0.31
T. cruzi-negative_217	0.27	0.26	0.20	0.20
T. cruzi-negative_218	0.67	0.35	0.18	0.25
T. cruzi-negative_219	0.21	0.23	0.15	0.16
T. cruzi-negative_220	0.15	0.21	0.16	0.15
T. cruzi-negative_221	0.16	0.19	0.17	0.16
T. cruzi-negative_222	0.18	0.24	0.21	0.18
T. cruzi-negative_223	0.17	0.16	0.16	0.14
T. cruzi-negative_224	0.39	0.42	0.39	0.28

*T. cruz*i-negative_225	0.32	0.40	0.32	0.26
*T. cruz*i-negative_226	0.36	0.40	0.36	0.33
*T. cruz*i-negative_227	0.25	0.26	0.21	0.19
*T. cruz*i-negative_228	0.21	0.25	0.23	0.19
*T. cruz*i-negative_229	0.16	0.19	0.19	0.15
*T. cruz*i-negative_230	0.16	0.21	0.18	0.18
*T. cruz*i-negative_231	0.25	0.22	0.24	0.20
*T. cruz*i-negative_232	0.19	0.18	0.17	0.14
*T. cruz*i-negative_233	0.17	0.26	0.25	0.20
*T. cruz*i-negative_234	0.14	0.20	0.15	0.15
*T. cruz*i-negative_235	0.14	0.20	0.15	0.14
*T. cruz*i-negative_236	0.19	0.22	0.20	0.20
*T. cruz*i-negative_237	0.13	0.21	0.16	0.15
*T. cruz*i-negative_238	0.10	0.13	0.14	0.12
*T. cruz*i-negative_239	0.15	0.19	0.14	0.13
*T. cruz*i-negative_240	0.17	0.17	0.11	0.14
*T. cruz*i-negative_241	0.14	0.26	0.27	0.16
*T. cruz*i-negative_242	0.10	0.17	0.15	0.12
*T. cruz*i-negative_243	0.17	0.22	0.25	0.17
*T. cruz*i-negative_244	0.11	0.17	0.12	0.10
*T. cruz*i-negative_245	0.15	0.21	0.15	0.14
*T. cruz*i-negative_246	0.14	0.16	0.14	0.14
*T. cruz*i-negative_247	0.13	0.16	0.11	0.12
*T. cruz*i-negative_248	0.13	0.23	0.12	0.11
*T. cruz*i-negative_249	0.11	0.23	0.20	0.14
*T. cruz*i-negative_250	0.07	0.13	0.12	0.08
*T. cruz*i-negative_251	0.20	0.33	0.29	0.25
*T. cruz*i-negative_252	0.13	0.21	0.19	0.12
*T. cruz*i-negative_253	0.15	0.18	0.16	0.14
*T. cruz*i-negative_254	0.13	0.14	0.11	0.15
*T. cruz*i-negative_255	0.20	0.23	0.17	0.21
*T. cruz*i-negative_256	0.14	0.13	0.08	0.10
*T. cruz*i-negative_257	0.21	0.31	0.27	0.18
*T. cruz*i-negative_258	0.24	0.28	0.27	0.18
*T. cruz*i-negative_259	0.22	0.26	0.20	0.14
*T. cruz*i-negative_260	0.26	0.31	0.24	0.20
*T. cruz*i-negative_261	0.20	0.22	0.24	0.14
*T. cruz*i-negative_262	0.21	0.25	0.21	0.17
*T. cruz*i-negative_263	0.18	0.17	0.17	0.12
*T. cruz*i-negative_264	0.16	0.16	0.15	0.11
*T. cruz*i-negative_265	0.13	0.16	0.15	0.14
*T. cruz*i-negative_266	0.16	0.24	0.20	0.14
*T. cruz*i-negative_267	0.09	0.14	0.11	0.08

*T. cruz*i-negative_268	0.12	0.17	0.14	0.10
*T. cruz*i-negative_269	0.16	0.18	0.16	0.13
*T. cruz*i-negative_270	0.21	0.24	0.28	0.21
*T. cruz*i-negative_271	0.19	0.18	0.18	0.16
*T. cruz*i-negative_272	0.16	0.20	0.12	0.12
*T. cruz*i-negative_273	0.15	0.31	0.23	0.15
*T. cruz*i-negative_274	0.15	0.25	0.20	0.15
*T. cruz*i-negative_275	0.20	0.26	0.22	0.18
*T. cruz*i-negative_276	0.25	0.26	0.24	0.28
*T. cruz*i-negative_277	0.19	0.26	0.22	0.19
*T. cruz*i-negative_278	0.20	0.21	0.20	0.14
*T. cruz*i-negative_279	0.23	0.27	0.20	0.16
*T. cruz*i-negative_280	0.33	0.33	0.18	0.19
*T. cruz*i-negative_281	0.13	0.21	0.16	0.11
*T. cruz*i-negative_282	0.11	0.15	0.13	0.08
*T. cruz*i-negative_283	0.15	0.22	0.21	0.15
*T. cruz*i-negative_284	0.22	0.41	0.21	0.18
*T. cruz*i-negative_285	0.21	0.24	0.23	0.18
*T. cruz*i-negative_286	0.10	0.13	0.15	0.08
*T. cruz*i-negative_287	0.15	0.16	0.15	0.10
*T. cruz*i-negative_288	0.19	0.40	0.17	0.13
*T. cruz*i-negative_289	0.24	0.32	0.32	0.20
*T. cruz*i-negative_290	0.14	0.22	0.16	0.18
*T. cruz*i-negative_291	0.18	0.25	0.16	0.15
*T. cruz*i-negative_292	0.20	0.21	0.20	0.14
*T. cruz*i-negative_293	0.25	0.24	0.19	0.18
*T. cruz*i-negative_294	0.12	0.16	0.13	0.12
*T. cruz*i-negative_295	0.14	0.11	0.11	0.22
*T. cruz*i-negative_296	0.16	0.19	0.14	0.10
*T. cruz*i-negative_297	0.13	0.27	0.10	0.10
*T. cruz*i-negative_298	0.33	0.41	0.26	0.25
*T. cruz*i-negative_299	0.27	0.25	0.18	0.18
*T. cruz*i-negative_300	0.18	0.20	0.15	0.44
*T. cruz*i-negative_301	0.28	0.24	0.19	0.19
*T. cruz*i-negative_302	0.13	0.13	0.13	0.12
*T. cruz*i-negative_303	0.19	0.23	0.16	0.17
*T. cruz*i-negative_304	0.21	0.24	0.15	0.14
*T. cruz*i-negative_305	0.25	0.23	0.27	0.18
*T. cruz*i-negative_306	0.28	0.27	0.26	0.24
*T. cruz*i-negative_307	0.24	0.25	0.20	0.21
*T. cruz*i-negative_308	0.18	0.23	0.16	0.12
*T. cruz*i-negative_309	0.15	0.17	0.11	0.09

*T. cruz*i-negative_310	0.25	0.23	0.22	0.22
*T. cruz*i-negative_311	0.17	0.16	0.14	0.12
*T. cruz*i-negative_312	0.23	0.22	0.42	0.25
*T. cruz*i-negative_313	0.28	0.33	0.29	0.26
*T. cruz*i-negative_314	0.24	0.25	0.20	0.21
*T. cruz*i-negative_315	0.17	0.16	0.13	0.18
*T. cruz*i-negative_316	0.21	0.22	0.17	0.18
*T. cruz*i-negative_317	0.23	0.25	0.17	0.19
*T. cruz*i-negative_318	0.26	0.26	0.27	0.23
*T. cruz*i-negative_319	0.17	0.18	0.13	0.15
*T. cruz*i-negative_320	0.13	0.15	0.13	0.15
*T. cruz*i-negative_321	0.19	0.19	0.16	0.18
*T. cruz*i-negative_322	0.13	0.13	0.12	0.14
*T. cruz*i-negative_323	0.25	0.25	0.19	0.19
*T. cruz*i-negative_324	0.22	0.25	0.19	0.18
*T. cruz*i-negative_325	0.19	0.16	0.13	0.13
*T. cruz*i-negative_326	0.28	0.35	0.26	0.27
*T. cruz*i-negative_327	0.17	0.19	0.14	0.16
*T. cruz*i-negative_328	0.28	0.29	0.25	0.24
*T. cruz*i-negative_329	0.19	0.22	0.16	0.15
*T. cruz*i-negative_330	0.20	0.21	0.17	0.16
*T. cruz*i-negative_331	0.20	0.24	0.15	0.15
*T. cruz*i-negative_332	0.19	0.15	0.14	0.14
*T. cruz*i-negative_333	0.15	0.16	0.11	0.13
*T. cruz*i-negative_334	0.14	0.16	0.12	0.12
*T. cruz*i-negative_335	0.12	0.10	0.09	0.11
*T. cruz*i-negative_336	0.11	0.12	0.09	0.11
*T. cruz*i-negative_337	0.19	0.19	0.19	0.23
*T. cruz*i-negative_338	0.18	0.23	0.14	0.16
*T. cruz*i-negative_339	0.25	0.25	0.59	0.73
*T. cruz*i-negative_340	0.36	0.43	0.34	0.47
*T. cruz*i-negative_341	0.16	0.15	0.13	0.12
*T. cruz*i-negative_342	0.34	0.22	0.17	0.19
*T. cruz*i-negative_343	0.22	0.22	0.19	0.20
*T. cruz*i-negative_344	0.15	0.14	0.12	0.15
*T. cruz*i-negative_345	0.21	0.25	0.23	0.23
*T. cruz*i-negative_346	0.23	0.21	0.21	0.18
*T. cruz*i-negative_347	0.39	0.35	0.31	0.32
*T. cruz*i-negative_348	0.18	0.16	0.17	0.16
*T. cruz*i-negative_349	0.24	0.29	0.20	0.24
*T. cruz*i-negative_350	0.19	0.18	0.13	0.13
*T. cruz*i-negative_351	0.17	0.16	0.16	0.16
*T. cruz*i-negative_352	0.28	0.26	0.23	0.23

*T. cruz*i-negative_353	0.16	0.19	0.23	0.15
*T. cruz*i-negative_354	0.25	0.26	0.22	0.27
*T. cruz*i-negative_355	0.15	0.15	0.31	0.14
*T. cruz*i-negative_356	0.24	0.30	0.18	0.20
*T. cruz*i-negative_357	0.26	0.31	0.23	0.23
*T. cruz*i-negative_358	0.19	0.21	0.16	0.17
*T. cruz*i-negative_359	0.26	0.26	0.19	0.22
*T. cruz*i-negative_360	0.29	0.30	0.22	0.23
*T. cruz*i-negative_361	0.22	0.21	0.17	0.18
*T. cruz*i-negative_362	0.27	0.28	0.20	0.26
*T. cruz*i-negative_363	0.32	0.27	0.28	0.22
*T. cruz*i-negative_364	0.31	0.34	0.25	0.23
*T. cruz*i-negative_365	0.26	0.29	0.20	0.23
*T. cruz*i-negative_366	0.16	0.15	0.11	0.12
*T. cruz*i-negative_367	0.26	0.26	0.23	0.25
*T. cruz*i-negative_368	0.26	0.29	0.21	0.20
*T. cruz*i-negative_369	0.22	0.25	0.21	0.22
*T. cruz*i-negative_370	0.12	0.14	0.16	0.12
*T. cruz*i-negative_371	0.28	0.33	0.22	0.23
*T. cruz*i-negative_372	0.36	0.40	0.29	0.41
*T. cruz*i-negative_373	0.09	0.09	0.08	0.07
*T. cruz*i-negative_374	0.31	0.34	0.24	0.22
*T. cruz*i-negative_375	0.17	0.19	0.15	0.14
*T. cruz*i-negative_376	0.23	0.27	0.18	0.22
*T. cruz*i-negative_377	0.21	0.16	0.19	0.15
*T. cruz*i-negative_378	0.18	0.20	0.17	0.15
*T. cruz*i-negative_379	0.18	0.18	0.15	0.15
*T. cruz*i-negative_380	0.26	0.24	0.18	0.22
*T. cruz*i-negative_381	0.15	0.14	0.12	0.10
*T. cruz*i-negative_382	0.21	0.17	0.15	0.10
*T. cruz*i-negative_383	0.26	0.20	0.20	0.18
*T. cruz*i-negative_384	0.17	0.19	0.13	0.19
*T. cruz*i-negative_385	0.15	0.17	0.10	0.10
*T. cruz*i-negative_386	0.27	0.25	0.13	0.17
*T. cruz*i-negative_387	0.22	0.16	0.10	0.11
*T. cruz*i-negative_388	0.30	0.31	0.22	0.25
*T. cruz*i-negative_389	0.18	0.19	0.14	0.14
*T. cruz*i-negative_390	0.11	0.15	0.06	0.08
*T. cruz*i-negative_391	0.21	0.30	0.18	0.15
*T. cruz*i-negative_392	0.19	0.23	0.17	0.16
*T. cruz*i-negative_393	0.30	0.29	0.17	0.23
*T. cruz*i-negative_394	0.21	0.18	0.13	0.15

T. cruzi-negative_395	0.25	0.26	0.19	0.18
T. cruzi-negative_396	0.36	0.20	0.17	0.28
T. cruzi-negative_397	0.21	0.26	0.17	0.20
T. cruzi-negative_398	0.31	0.47	0.21	0.24
T. cruzi-negative_399	0.17	0.24	0.17	0.13
T. cruzi-negative_400	0.19	0.26	0.13	0.17
T. cruzi-negative_401	0.25	0.36	0.22	0.23
T. cruzi-negative_402	0.15	0.24	0.15	0.14
T. cruzi-negative_403	0.20	0.18	0.13	0.14
T. cruzi-negative_404	0.16	0.18	0.18	0.16
T. cruzi-negative_405	0.11	0.15	0.11	0.12
T. cruzi-negative_406	0.27	0.37	0.13	0.17
T. cruzi-negative_407	0.27	0.31	0.18	0.27
T. cruzi-negative_408	0.19	0.21	0.15	0.12
T. cruzi-negative_409	0.22	0.23	0.14	0.16
T. cruzi-negative_410	0.25	0.25	0.15	0.19
T. cruzi-negative_411	0.23	0.31	0.21	0.19
T. cruzi-negative_412	0.13	0.18	0.14	0.13
T. cruzi-negative_413	0.25	0.32	0.27	0.24
T. cruzi-negative_414	0.10	0.14	0.06	0.06
T. cruzi-negative_415	0.19	0.27	0.11	0.15
T. cruzi-negative_416	0.13	0.16	0.12	0.09
T. cruzi-negative_417	0.21	0.31	0.22	0.18
T. cruzi-negative_418	0.27	0.29	0.17	0.21
T. cruzi-negative_419	0.21	0.37	0.18	0.17
T. cruzi-negative_420	0.14	0.22	0.15	0.11
T. cruzi-negative_421	0.17	0.30	0.19	0.18
T. cruzi-negative_422	0.20	0.25	0.17	0.12
T. cruzi-negative_423	0.22	0.38	0.20	0.19
T. cruzi-negative_424	0.14	0.17	0.08	0.11
T. cruzi-negative_425	0.26	0.30	0.15	0.16
T. cruzi-negative_426	0.15	0.18	0.10	0.09
T. cruzi-negative_427	0.17	0.22	0.17	0.12
T. cruzi-negative_428	0.22	0.35	0.29	0.22
T. cruzi-negative_429	0.12	0.17	0.09	0.10
T. cruzi-negative_430	0.26	0.31	0.13	0.13
T. cruzi-negative_431	0.18	0.26	0.13	0.11
T. cruzi-negative_432	0.19	0.30	0.17	0.15
T. cruzi-negative_433	0.28	0.26	0.17	0.15
T. cruzi-negative_434	0.12	0.19	0.13	0.10
T. cruzi-negative_435	0.13	0.21	0.18	0.11
T. cruzi-negative_436	0.22	0.25	0.20	0.18
T. cruzi-negative_437	0.17	0.31	0.19	0.19

*T. cruz*i-negative_438	0.11	0.19	0.08	0.05
*T. cruz*i-negative_439	0.31	0.41	0.24	0.24
*T. cruz*i-negative_440	0.23	0.35	0.18	0.19
*T. cruz*i-negative_441	0.22	0.31	0.18	0.16
*T. cruz*i-negative_442	0.21	0.31	0.19	0.17
*T. cruz*i-negative_443	0.22	0.31	0.28	0.21
*T. cruz*i-negative_444	0.14	0.21	0.18	0.13
*T. cruz*i-negative_445	0.22	0.31	0.22	0.26
*T. cruz*i-negative_446	0.19	0.31	0.18	0.14
*T. cruz*i-negative_447	0.09	0.16	0.14	0.10
*T. cruz*i-negative_448	0.15	0.24	0.14	0.10
*T. cruz*i-negative_449	0.16	0.24	0.18	0.15
*T. cruz*i-negative_450	0.14	0.24	0.16	0.14
*T. cruz*i-negative_451	0.10	0.17	0.13	0.11
*T. cruz*i-negative_452	0.18	0.27	0.16	0.19
*T. cruz*i-negative_453	0.24	0.35	0.26	0.31
*T. cruz*i-negative_454	0.17	0.35	0.17	0.12
*T. cruz*i-negative_455	0.21	0.35	0.21	0.17
*T. cruz*i-negative_456	0.19	0.30	0.18	0.13
*T. cruz*i-negative_457	0.32	0.28	0.17	0.21
*T. cruz*i-negative_458	0.09	0.18	0.10	0.08
*T. cruz*i-negative_459	0.16	0.24	0.16	0.15
*T. cruz*i-negative_460	0.14	0.24	0.17	0.16
*T. cruz*i-negative_461	0.18	0.32	0.21	0.23
*T. cruz*i-negative_462	0.24	0.40	0.21	0.14
*T. cruz*i-negative_463	0.23	0.35	0.19	0.15
*T. cruz*i-negative_464	0.16	0.33	0.16	0.14
*T. cruz*i-negative_465	0.22	0.34	0.18	0.17
*T. cruz*i-negative_466	0.20	0.31	0.19	0.15
*T. cruz*i-negative_467	0.27	0.45	0.26	0.26
*T. cruz*i-negative_468	0.15	0.29	0.14	0.15
*T. cruz*i-negative_469	0.26	0.28	0.25	0.33
*T. cruz*i-negative_470	0.38	0.43	0.30	0.57
*T. cruz*i-negative_471	0.25	0.23	0.23	0.35
*T. cruz*i-negative_472	0.31	0.33	0.23	0.41
*T. cruz*i-negative_473	0.35	0.41	0.31	0.68
*T. cruz*i-negative_474	0.25	0.18	0.17	0.32
*T. cruz*i-negative_475	0.40	0.33	0.27	0.45
*T. cruz*i-negative_476	0.23	0.19	0.13	0.25
*T. cruz*i-negative_477	0.18	0.21	0.16	0.46
*T. cruz*i-negative_478	0.23	0.21	0.18	0.27
*T. cruz*i-negative_479	0.18	0.18	0.16	0.26

T. cruzi-negative_480	0.14	0.17	0.12	0.21
T. cruzi-negative_481	0.23	0.13	0.14	0.25
T. cruzi-negative_482	0.28	0.33	0.29	0.51
T. cruzi-negative_483	0.24	0.18	0.14	0.24
T. cruzi-negative_484	0.27	0.26	0.16	0.31
T. cruzi-negative_485	0.15	0.19	0.12	0.17
T. cruzi-negative_486	0.30	0.36	0.30	0.23
T. cruzi-negative_487	0.21	0.23	0.23	0.28
T. cruzi-negative_488	0.22	0.22	0.18	0.30
T. cruzi-negative_489	0.14	0.15	0.13	0.20
T. cruzi-negative_490	0.24	0.22	0.15	0.23
T. cruzi-negative_491	0.22	0.20	0.14	0.23
T. cruzi-negative_492	0.27	0.26	0.17	0.29
T. cruzi-negative_493	0.22	0.28	0.18	0.32
T. cruzi-negative_494	0.17	0.18	0.14	0.18
T. cruzi-negative_495	0.19	0.21	0.20	0.24
T. cruzi-negative_496	0.35	0.37	0.25	0.41
T. cruzi-negative_497	0.17	0.15	0.13	0.20
T. cruzi-negative_498	0.30	0.36	0.25	0.46
T. cruzi-negative_499	0.20	0.25	0.14	0.20
T. cruzi-negative_500	0.29	0.26	0.19	0.33
T. cruzi-negative_501	0.16	0.14	0.11	0.16
T. cruzi-negative_502	0.12	0.17	0.10	0.13
T. cruzi-negative_503	0.18	0.24	0.18	0.29
T. cruzi-negative_504	0.18	0.28	0.13	0.35
T. cruzi-negative_505	0.10	0.16	0.14	0.27
T. cruzi-negative_506	0.14	0.15	0.12	0.15
T. cruzi-negative_507	0.25	0.31	0.22	0.30
T. cruzi-negative_508	0.15	0.22	0.19	0.21
T. cruzi-negative_509	0.18	0.22	0.12	0.20
T. cruzi-negative_510	0.19	0.31	0.16	0.27
T. cruzi-negative_511	0.11	0.19	0.12	0.22
T. cruzi-negative_512	0.14	0.19	0.13	0.16
T. cruzi-negative_513	0.14	0.20	0.15	0.25
T. cruzi-negative_514	0.20	0.24	0.14	0.19
T. cruzi-negative_515	0.30	0.31	0.21	0.35
T. cruzi-negative_516	0.16	0.26	0.16	0.28
T. cruzi-negative_517	0.23	0.34	0.20	0.26
T. cruzi-negative_518	0.19	0.23	0.17	0.19
T. cruzi-negative_519	0.13	0.17	0.16	0.28
T. cruzi-negative_520	0.14	0.17	0.13	0.26
T. cruzi-negative_521	0.19	0.24	0.19	0.31
T. cruzi-negative_522	0.24	0.27	0.15	0.23

*T. cruz*i-negative_523	0.20	0.19	0.23	0.20
*T. cruz*i-negative_524	0.20	0.20	0.11	0.18
*T. cruz*i-negative_525	0.25	0.34	0.20	0.30
*T. cruz*i-negative_526	0.33	0.28	0.28	0.38
*T. cruz*i-negative_527	0.28	0.30	0.21	0.38
*T. cruz*i-negative_528	0.17	0.19	0.12	0.32
*T. cruz*i-negative_529	0.26	0.24	0.15	0.32
*T. cruz*i-negative_530	0.32	0.36	0.22	0.31
*T. cruz*i-negative_531	0.30	0.21	0.23	0.21
*T. cruz*i-negative_532	0.28	0.25	0.15	0.23
*T. cruz*i-negative_533	0.25	0.27	0.14	0.22
*T. cruz*i-negative_534	0.22	0.35	0.16	0.22
*T. cruz*i-negative_535	0.27	0.35	0.17	0.36
*T. cruz*i-negative_536	0.23	0.24	0.11	0.29
*T. cruz*i-negative_537	0.19	0.22	0.10	0.22
*T. cruz*i-negative_538	0.38	0.45	0.27	0.39
*T. cruz*i-negative_539	0.24	0.22	0.08	0.17
*T. cruz*i-negative_540	0.36	0.35	0.21	0.38
*T. cruz*i-negative_541	0.22	0.22	0.09	0.23
*T. cruz*i-negative_542	0.23	0.33	0.14	0.27
*T. cruz*i-negative_543	0.17	0.20	0.09	0.28
*T. cruz*i-negative_544	0.21	0.27	0.12	0.24
*T. cruz*i-negative_545	0.31	0.35	0.20	0.36
*T. cruz*i-negative_546	0.21	0.17	0.10	0.13
*T. cruz*i-negative_547	0.37	0.33	0.30	0.59
*T. cruz*i-negative_548	0.24	0.25	0.10	0.21
*T. cruz*i-negative_549	0.18	0.16	0.11	0.17
*T. cruz*i-negative_550	0.14	0.15	0.10	0.13
*T. cruz*i-negative_551	0.26	0.24	0.21	0.31
*T. cruz*i-negative_552	0.47	0.69	0.20	0.53
*T. cruz*i-negative_553	0.35	0.54	0.24	0.40
*T. cruz*i-negative_554	0.27	0.45	0.20	0.24
*T. cruz*i-negative_555	0.26	0.45	0.19	0.23
*T. cruz*i-negative_556	0.18	0.43	0.20	0.23
*T. cruz*i-negative_557	0.24	0.40	0.20	0.32
*T. cruz*i-negative_558	0.28	0.35	0.18	0.28
*T. cruz*i-negative_559	0.20	0.33	0.12	0.14
*T. cruz*i-negative_560	0.25	0.32	0.19	0.22
*T. cruz*i-negative_561	0.21	0.32	0.17	0.28
*T. cruz*i-negative_562	0.54	0.31	0.33	0.31
*T. cruz*i-negative_563	0.13	0.31	0.29	0.15
*T. cruz*i-negative_564	0.18	0.30	0.15	0.18

T. cruzi-negative_565	0.31	0.30	0.26	0.34
T. cruzi-negative_566	0.25	0.30	0.19	0.31
T. cruzi-negative_567	0.26	0.30	0.21	0.28
T. cruzi-negative_568	0.16	0.30	0.12	0.34
T. cruzi-negative_569	0.39	0.30	0.22	0.69
T. cruzi-negative_570	0.27	0.29	0.19	0.20
T. cruzi-negative_571	0.21	0.29	0.18	0.30
T. cruzi-negative_572	0.13	0.29	0.16	0.16
T. cruzi-negative_573	0.18	0.29	0.18	0.27
T. cruzi-negative_574	0.49	0.29	0.34	0.66
T. cruzi-negative_575	0.11	0.29	0.11	0.13
T. cruzi-negative_576	0.15	0.28	0.12	0.16
T. cruzi-negative_577	0.22	0.28	0.18	0.22
T. cruzi-negative_578	0.17	0.28	0.13	0.19
T. cruzi-negative_579	0.27	0.28	0.22	0.34
T. cruzi-negative_580	0.25	0.27	0.24	0.33
T. cruzi-negative_581	0.13	0.27	0.14	0.19
T. cruzi-negative_582	0.26	0.27	0.22	0.30
T. cruzi-negative_583	0.23	0.26	0.24	0.22
T. cruzi-negative_584	0.24	0.25	0.17	0.19
T. cruzi-negative_585	0.17	0.25	0.13	0.19
T. cruzi-negative_586	0.08	0.25	0.09	0.12
T. cruzi-negative_587	0.17	0.25	0.15	0.24
T. cruzi-negative_588	0.11	0.24	0.12	0.16
T. cruzi-negative_589	0.37	0.24	0.35	0.73
T. cruzi-negative_590	0.17	0.24	0.11	0.21
T. cruzi-negative_591	0.14	0.24	0.11	0.20
T. cruzi-negative_592	0.18	0.24	0.14	0.19
T. cruzi-negative_593	0.30	0.24	0.21	0.32
T. cruzi-negative_594	0.33	0.24	0.28	0.52
T. cruzi-negative_595	0.14	0.23	0.12	0.17
T. cruzi-negative_596	0.28	0.23	0.28	0.35
T. cruzi-negative_597	0.21	0.23	0.18	0.26
T. cruzi-negative_598	0.30	0.23	0.24	0.36
T. cruzi-negative_599	0.25	0.22	0.19	0.21
T. cruzi-negative_600	0.14	0.22	0.16	0.16
T. cruzi-negative_601	0.20	0.22	0.16	0.20
T. cruzi-negative_602	0.15	0.22	0.14	0.21
T. cruzi-negative_603	0.08	0.22	0.16	0.17
T. cruzi-negative_604	0.14	0.21	0.14	0.14
T. cruzi-negative_605	0.16	0.21	0.14	0.23
T. cruzi-negative_606	0.15	0.20	0.15	0.19
T. cruzi-negative_607	0.23	0.20	0.19	0.17

*T. cruz*i-negative_608	0.18	0.20	0.23	0.33
*T. cruz*i-negative_609	0.14	0.20	0.15	0.29
*T. cruz*i-negative_610	0.31	0.19	0.16	0.17
*T. cruz*i-negative_611	0.19	0.19	0.14	0.21
*T. cruz*i-negative_612	0.17	0.19	0.15	0.19
*T. cruz*i-negative_613	0.08	0.19	0.08	0.10
*T. cruz*i-negative_614	0.16	0.19	0.18	0.20
*T. cruz*i-negative_615	0.12	0.18	0.11	0.11
*T. cruz*i-negative_616	0.28	0.18	0.37	0.75
*T. cruz*i-negative_617	0.19	0.18	0.12	0.20
*T. cruz*i-negative_618	0.09	0.17	0.10	0.12
*T. cruz*i-negative_619	0.21	0.17	0.18	0.19
*T. cruz*i-negative_620	0.33	0.17	0.22	0.24
*T. cruz*i-negative_621	0.12	0.17	0.11	0.14
*T. cruz*i-negative_622	0.21	0.16	0.19	0.51
*T. cruz*i-negative_623	0.22	0.16	0.23	0.29
*T. cruz*i-negative_624	0.23	0.16	0.22	0.26
*T. cruz*i-negative_625	0.19	0.16	0.15	0.24
*T. cruz*i-negative_626	0.14	0.16	0.20	0.28
*T. cruz*i-negative_627	0.20	0.15	0.16	0.25
*T. cruz*i-negative_628	0.21	0.15	0.22	0.31
*T. cruz*i-negative_629	0.11	0.14	0.14	0.16
*T. cruz*i-negative_630	0.30	0.14	0.22	0.39
*T. cruz*i-negative_631	0.49	0.13	0.46	0.40
*T. cruz*i-negative_632	0.14	0.13	0.15	0.27
*T. cruz*i-negative_633	0.13	0.13	0.11	0.19
*T. cruz*i-negative_634	0.13	0.12	0.16	0.17
*T. cruz*i-negative_635	0.11	0.12	0.14	0.21
*T. cruz*i-negative_636	0.12	0.11	0.13	0.16
*T. cruz*i-negative_637	0.50	0.61	0.21	0.19
*T. cruz*i-negative_638	0.33	0.40	0.24	0.17
*T. cruz*i-negative_639	0.28	0.33	0.22	0.27
*T. cruz*i-negative_640	0.19	0.24	0.24	0.17
*T. cruz*i-negative_641	0.22	0.26	0.31	0.16
*T. cruz*i-negative_642	0.30	0.32	0.37	0.13
*T. cruz*i-negative_643	0.33	0.43	0.31	0.16
*T. cruz*i-negative_644	0.45	0.37	0.42	0.71
*T. cruz*i-negative_645	0.28	0.46	0.30	0.18
*T. cruz*i-negative_646	0.22	0.19	0.19	0.11
*T. cruz*i-negative_647	0.29	0.27	0.24	0.33
*T. cruz*i-negative_648	0.54	0.48	0.48	0.34
*T. cruz*i-negative_649	0.19	0.28	0.29	0.25

T. cruzi-negative_650	0.31	0.36	0.36	0.11
T. cruzi-negative_651	0.40	0.45	0.44	0.50
T. cruzi-negative_652	0.39	0.36	0.29	0.23
T. cruzi-negative_653	0.41	0.31	0.31	0.39
T. cruzi-negative_654	0.32	0.20	0.18	0.12
T. cruzi-negative_655	0.52	0.28	0.48	0.49
T. cruzi-negative_656	0.33	0.25	0.21	0.21
T. cruzi-negative_657	0.31	0.23	0.22	0.22
T. cruzi-negative_658	0.27	0.28	0.20	0.13
T. cruzi-negative_659	0.37	0.37	0.48	0.16
T. cruzi-negative_660	0.52	0.37	0.26	0.24
T. cruzi-negative_661	0.37	0.25	0.18	0.14
T. cruzi-negative_662	0.42	0.27	0.34	0.20
T. cruzi-negative_663	0.38	0.29	0.25	0.26
T. cruzi-negative_664	0.25	0.18	0.16	0.10
T. cruzi-negative_665	0.56	0.31	0.35	0.35
T. cruzi-negative_666	0.38	0.34	0.22	0.16
T. cruzi-negative_667	0.34	0.25	0.17	0.16
T. cruzi-negative_668	0.33	0.24	0.14	0.13
T. cruzi-negative_669	0.25	0.30	0.13	0.16
T. cruzi-negative_670	0.34	0.25	0.59	0.16
T. cruzi-negative_671	0.45	0.33	0.28	0.21
T. cruzi-negative_672	0.31	0.21	0.19	0.13
T. cruzi-negative_673	0.57	0.42	0.27	0.33
T. cruzi-negative_674	0.31	0.33	0.23	0.16
T. cruzi-negative_675	0.34	0.26	0.18	0.16
T. cruzi-negative_676	0.39	0.29	0.27	0.25
T. cruzi-negative_677	0.31	0.29	0.21	0.17
T. cruzi-negative_678	0.30	0.23	0.17	0.17
T. cruzi-negative_679	0.49	0.29	0.28	0.26
T. cruzi-negative_680	0.30	0.26	0.26	0.19
T. cruzi-negative_681	0.28	0.24	0.21	0.18
T. cruzi-negative_682	0.37	0.28	0.22	0.14
T. cruzi-negative_683	0.31	0.29	0.21	0.14
T. cruzi-negative_684	0.56	0.39	0.29	0.41
T. cruzi-negative_685	0.48	0.33	0.21	0.22
T. cruzi-negative_686	0.33	0.25	0.32	0.20
T. cruzi-negative_687	0.33	0.23	0.19	0.18
T. cruzi-negative_688	0.42	0.28	0.23	0.27
T. cruzi-negative_689	0.42	0.64	0.42	0.50
T. cruzi-negative_690	0.24	0.17	0.15	0.10
T. cruzi-negative_691	0.44	0.24	0.23	0.18
T. cruzi-negative_692	0.55	0.30	0.25	0.22

*T. cruz*i-negative_693	0.33	0.20	0.18	0.15
*T. cruz*i-negative_694	0.29	0.20	0.22	0.16
*T. cruz*i-negative_695	0.49	0.33	0.28	0.28
*T. cruz*i-negative_696	0.43	0.33	0.28	0.26
*T. cruz*i-negative_697	0.26	0.19	0.19	0.19
*T. cruz*i-negative_698	0.33	0.22	0.26	0.24
*T. cruz*i-negative_699	0.42	0.30	0.28	0.27
*T. cruz*i-negative_700	0.26	0.32	0.70	0.25
*T. cruz*i-negative_701	0.32	0.20	0.17	0.21
*T. cruz*i-negative_702	0.21	0.18	0.15	0.15
*T. cruz*i-negative_703	0.61	0.37	0.37	0.34
*T. cruz*i-negative_704	0.31	0.22	0.20	0.25
*T. cruz*i-negative_705	0.26	0.11	0.12	0.11
*T. cruz*i-negative_706	0.29	0.19	0.19	0.15
*T. cruz*i-negative_707	0.36	0.24	0.20	0.22
*T. cruz*i-negative_708	0.65	0.40	0.33	0.34
*T. cruz*i-negative_709	0.25	0.17	0.16	0.28
*T. cruz*i-negative_710	0.41	0.34	0.27	0.32
*T. cruz*i-negative_711	0.43	0.31	0.56	0.35
*T. cruz*i-negative_712	0.33	0.31	0.26	0.36
*T. cruz*i-negative_713	0.34	0.22	0.24	0.24
*T. cruz*i-negative_714	0.41	0.30	0.28	0.27
*T. cruz*i-negative_715	0.55	0.38	0.35	0.36
*T. cruz*i-negative_716	0.60	0.44	0.32	0.31
*T. cruz*i-negative_717	0.42	0.32	0.27	0.31
*T. cruz*i-negative_718	0.42	0.25	0.23	0.27
*T. cruz*i-negative_719	0.40	0.25	0.25	0.26
*T. cruz*i-negative_720	0.38	0.26	0.24	0.33
*T. cruz*i-negative_721	0.25	0.32	0.12	0.26
*T. cruz*i-negative_722	0.28	0.30	0.14	0.21
*T. cruz*i-negative_723	0.26	0.31	0.13	0.23
*T. cruz*i-negative_724	0.23	0.28	0.11	0.23
*T. cruz*i-negative_725	0.40	0.47	0.34	0.41
*T. cruz*i-negative_726	0.38	0.36	0.25	0.33
*T. cruz*i-negative_727	0.26	0.32	0.17	0.27
*T. cruz*i-negative_728	0.30	0.27	0.15	0.18
*T. cruz*i-negative_729	0.20	0.26	0.15	0.24
*T. cruz*i-negative_730	0.30	0.31	0.16	0.24
*T. cruz*i-negative_731	0.23	0.33	0.14	0.19
*T. cruz*i-negative_732	0.25	0.30	0.15	0.29
*T. cruz*i-negative_733	0.18	0.21	0.08	0.19
*T. cruz*i-negative_734	0.20	0.20	0.07	0.17

T. cruzi-negative_735	0.49	0.60	0.28	0.33
T. cruzi-negative_736	0.24	0.25	0.11	0.16
T. cruzi-negative_737	0.48	0.55	0.38	0.45
T. cruzi-negative_738	0.25	0.24	0.08	0.15
T. cruzi-negative_739	0.15	0.21	0.05	0.15
T. cruzi-negative_740	0.25	0.32	0.09	0.24
T. cruzi-negative_741	0.39	0.42	0.33	0.36
T. cruzi-negative_742	0.40	0.51	0.34	0.44
T. cruzi-negative_743	0.30	0.36	0.20	0.24
T. cruzi-negative_744	0.33	0.41	0.26	0.27
T. cruzi-negative_745	0.34	0.38	0.21	0.24
T. cruzi-negative_746	0.32	0.38	0.27	0.25
T. cruzi-negative_747	0.30	0.40	0.28	0.22
T. cruzi-negative_748	0.36	0.39	0.25	0.30
T. cruzi-negative_749	0.32	0.48	0.38	0.29
T. cruzi-negative_750	0.43	0.38	0.26	0.34
T. cruzi-negative_751	0.33	0.34	0.14	0.19
T. cruzi-negative_752	0.32	0.30	0.30	0.19
T. cruzi-negative_753	0.31	0.39	0.27	0.27
T. cruzi-negative_754	0.29	0.24	0.24	0.15
T. cruzi-negative_755	0.30	0.32	0.26	0.23
T. cruzi-negative_756	0.40	0.31	0.27	0.24
T. cruzi-negative_757	0.30	0.38	0.32	0.30
T. cruzi-negative_758	0.18	0.17	0.07	0.12
T. cruzi-negative_759	0.30	0.27	0.15	0.14
T. cruzi-negative_760	0.21	0.23	0.15	0.13
T. cruzi-negative_761	0.24	0.30	0.15	0.21
T. cruzi-negative_762	0.38	0.33	0.24	0.21
T. cruzi-negative_763	0.37	0.40	0.34	0.29
T. cruzi-negative_764	0.30	0.34	0.25	0.29
T. cruzi-negative_765	0.20	0.27	0.16	0.18
T. cruzi-negative_766	0.29	0.28	0.23	0.18
T. cruzi-negative_767	0.27	0.41	0.20	0.21
T. cruzi-negative_768	0.47	0.35	0.29	0.17
T. cruzi-negative_769	0.38	0.49	0.29	0.34
T. cruzi-negative_770	0.46	0.43	0.44	0.31
T. cruzi-negative_771	0.32	0.44	0.33	0.22
T. cruzi-negative_772	0.31	0.29	0.20	0.24
T. cruzi-negative_773	0.33	0.40	0.29	0.28
T. cruzi-negative_774	0.28	0.33	0.18	0.17
T. cruzi-negative_775	0.41	0.62	0.31	0.22
T. cruzi-negative_776	0.29	0.25	0.21	0.13
T. cruzi-negative_777	0.23	0.49	0.24	0.15

*T. cruz*i-negative_778	0.17	0.19	0.09	0.09
*T. cruz*i-negative_779	0.19	0.27	0.18	0.11
*T. cruz*i-negative_780	0.32	0.35	0.30	0.21
*T. cruz*i-negative_781	0.27	0.33	0.30	0.19
*T. cruz*i-negative_782	0.27	0.28	0.15	0.10
*T. cruz*i-negative_783	0.34	0.41	0.20	0.14
*T. cruz*i-negative_784	0.29	0.36	0.24	0.19
*T. cruz*i-negative_785	0.27	0.40	0.11	0.10
*T. cruz*i-negative_786	0.29	0.32	0.24	0.14
*T. cruz*i-negative_787	0.31	0.55	0.34	0.18
*T. cruz*i-negative_788	0.22	0.27	0.17	0.12
*T. cruz*i-negative_789	0.40	0.43	0.32	0.20
*T. cruz*i-negative_790	0.33	0.36	0.31	0.12
*T. cruz*i-negative_791	0.28	0.38	0.19	0.11
*T. cruz*i-negative_792	0.28	0.35	0.27	0.12
*T. cruz*i-negative_793	0.35	0.50	0.34	0.12
*T. cruz*i-negative_794	0.18	0.20	0.09	0.05
*T. cruz*i-negative_795	0.27	0.35	0.15	0.11
*T. cruz*i-negative_796	0.27	0.26	0.14	0.12
*T. cruz*i-negative_797	0.29	0.30	0.21	0.12
*T. cruz*i-negative_798	0.43	0.45	0.23	0.15
*T. cruz*i-negative_799	0.33	0.39	0.17	0.11
*T. cruz*i-negative_800	0.19	0.18	0.09	0.03
*T. cruz*i-negative_801	0.50	0.64	0.39	0.15
*T. cruz*i-negative_802	0.35	0.32	0.21	0.12
*T. cruz*i-negative_803	0.23	0.34	0.13	0.09
*T. cruz*i-negative_804	0.33	0.35	0.23	0.15
*T. cruz*i-negative_805	0.25	0.22	0.28	0.21
*T. cruz*i-negative_806	0.29	0.32	0.35	0.35
*T. cruz*i-negative_807	0.26	0.24	0.17	0.16
*T. cruz*i-negative_808	0.37	0.25	0.31	0.31
*T. cruz*i-negative_809	0.27	0.28	0.24	0.20
*T. cruz*i-negative_810	0.29	0.30	0.42	0.29
*T. cruz*i-negative_811	0.21	0.26	0.28	0.23
*T. cruz*i-negative_812	0.19	0.25	0.14	0.12
*T. cruz*i-negative_813	0.43	0.40	0.19	0.17
*T. cruz*i-negative_814	0.15	0.29	0.18	0.16
*T. cruz*i-negative_815	0.30	0.21	0.19	0.15
*T. cruz*i-negative_816	0.33	0.39	0.15	0.13
*T. cruz*i-negative_817	0.39	0.32	0.27	0.29
*T. cruz*i-negative_818	0.25	0.28	0.17	0.15
*T. cruz*i-negative_819	0.31	0.30	0.20	0.21

T. cruzi-negative_820	0.25	0.32	0.17	0.17
T. cruzi-negative_821	0.28	0.25	0.21	0.17
T. cruzi-negative_822	0.59	0.47	0.17	0.14
T. cruzi-negative_823	0.48	0.38	0.12	0.17
T. cruzi-negative_824	0.27	0.32	0.19	0.20
T. cruzi-negative_825	0.43	0.38	0.17	0.26
T. cruzi-negative_826	0.17	0.20	0.25	0.12
T. cruzi-negative_827	0.29	0.40	0.16	0.12
T. cruzi-negative_828	0.19	0.22	0.30	0.20
T. cruzi-negative_829	0.21	0.27	0.31	0.30
T. cruzi-negative_830	0.25	0.21	0.16	0.15
T. cruzi-negative_831	0.36	0.32	0.22	0.29
T. cruzi-negative_832	0.25	0.12	0.23	0.31
T. cruzi-negative_833	0.24	0.31	0.28	0.40
T. cruzi-negative_834	0.25	0.38	0.17	0.15
T. cruzi-negative_835	0.30	0.39	0.24	0.16
T. cruzi-negative_836	0.16	0.52	0.22	0.16
T. cruzi-negative_837	0.23	0.26	0.17	0.15
T. cruzi-negative_838	0.24	0.30	0.17	0.17
T. cruzi-negative_839	0.33	0.32	0.12	0.16
T. cruzi-negative_840	0.59	0.52	0.14	0.20
T. cruzi-negative_841	0.41	0.46	0.16	0.23
T. cruzi-negative_842	0.39	0.44	0.31	0.23
T. cruzi-negative_843	0.36	0.39	0.31	0.26
T. cruzi-negative_844	0.27	0.33	0.28	0.20
T. cruzi-negative_845	0.43	0.39	0.13	0.12
T. cruzi-negative_846	0.25	0.27	0.20	0.19
T. cruzi-negative_847	0.23	0.28	0.21	0.26
T. cruzi-negative_848	0.34	0.35	0.18	0.22
T. cruzi-negative_849	0.28	0.30	0.17	0.23
T. cruzi-negative_850	0.27	0.29	0.41	0.37
T. cruzi-negative_851	0.33	0.36	0.16	0.16
T. cruzi-negative_852	0.25	0.24	0.21	0.20
T. cruzi-negative_853	0.37	0.50	0.13	0.14
T. cruzi-negative_854	0.29	0.28	0.24	0.24
T. cruzi-negative_855	0.52	0.39	0.20	0.28
T. cruzi-negative_856	0.46	0.45	0.18	0.24
T. cruzi-negative_857	0.49	0.50	0.17	0.20
T. cruzi-negative_858	0.31	0.29	0.21	0.23
T. cruzi-negative_859	0.23	0.28	0.17	0.20
T. cruzi-negative_860	0.22	0.25	0.13	0.12
T. cruzi-negative_861	0.22	0.21	0.24	0.23
T. cruzi-negative_862	0.31	0.36	0.19	0.19

*T. cruz*i-negative_863	0.56	0.43	0.21	0.20
*T. cruz*i-negative_864	0.39	0.37	0.49	0.27
*T. cruz*i-negative_865	0.35	0.41	0.26	0.31
*T. cruz*i-negative_866	0.25	0.24	0.23	0.19
*T. cruz*i-negative_867	0.28	0.34	0.20	0.28
*T. cruz*i-negative_868	0.37	0.27	0.27	0.28
*T. cruz*i-negative_869	0.58	0.50	0.25	0.26
*T. cruz*i-negative_870	0.63	0.49	0.12	0.13
*T. cruz*i-negative_871	0.62	0.47	0.34	0.26
*T. cruz*i-negative_872	0.40	0.30	0.11	0.11
*T. cruz*i-negative_873	0.44	0.32	0.18	0.21
*T. cruz*i-negative_874	0.43	0.27	0.19	0.17
*T. cruz*i-negative_875	0.41	0.21	0.14	0.18
*T. cruz*i-negative_876	0.48	0.34	0.27	0.28
*T. cruz*i-negative_877	0.38	0.30	0.16	0.17
*T. cruz*i-negative_878	0.48	0.33	0.18	0.19
*T. cruz*i-negative_879	0.34	0.21	0.22	0.24
*T. cruz*i-negative_880	0.35	0.21	0.20	0.20
*T. cruz*i-negative_881	0.49	0.32	0.18	0.20
*T. cruz*i-negative_882	0.57	0.27	0.30	0.22
*T. cruz*i-negative_883	0.60	0.17	0.18	0.21
*T. cruz*i-negative_884	0.29	0.21	0.26	0.25
*T. cruz*i-negative_885	0.54	0.25	0.25	0.23
*T. cruz*i-negative_886	0.36	0.15	0.20	0.16
*T. cruz*i-negative_887	0.55	0.26	0.16	0.17
*T. cruz*i-negative_888	0.30	0.17	0.15	0.17
*T. cruz*i-negative_889	0.16	0.32	0.12	0.20
*T. cruz*i-negative_890	0.19	0.46	0.12	0.26
*T. cruz*i-negative_891	0.09	0.20	0.11	0.19
*T. cruz*i-negative_892	0.33	0.46	0.13	0.17
*T. cruz*i-negative_893	0.10	0.37	0.11	0.22
*T. cruz*i-negative_894	0.24	0.32	0.22	0.23
*T. cruz*i-negative_895	0.21	0.38	0.15	0.25
*T. cruz*i-negative_896	0.05	0.19	0.12	0.26
*T. cruz*i-negative_897	0.14	0.28	0.16	0.28
*T. cruz*i-negative_898	0.15	0.32	0.16	0.18
*T. cruz*i-negative_899	0.11	0.21	0.10	0.21
*T. cruz*i-negative_900	0.21	0.23	0.18	0.27
*T. cruz*i-negative_901	0.18	0.52	0.05	0.27
*T. cruz*i-negative_902	0.10	0.30	0.20	0.28
*T. cruz*i-negative_903	0.15	0.34	0.20	0.29
*T. cruz*i-negative_904	0.11	0.29	0.20	0.30

T. cruzi-negative_905	0.25	0.34	0.14	0.27
T. cruzi-negative_906	0.11	0.28	0.21	0.39
T. cruzi-negative_907	0.08	0.20	0.26	0.34
T. cruzi-negative_908	0.12	0.36	0.12	0.21
T. cruzi-negative_909	0.24	0.38	0.20	0.35
T. cruzi-negative_910	0.13	0.40	0.12	0.20
T. cruzi-negative_911	0.10	0.26	0.19	0.29
T. cruzi-negative_912	0.21	0.44	0.11	0.18
T. cruzi-negative_913	0.37	0.53	0.12	0.21
T. cruzi-negative_914	0.07	0.29	0.10	0.20
T. cruzi-negative_915	0.23	0.37	0.13	0.30
T. cruzi-negative_916	0.23	0.34	0.05	0.16
T. cruzi-negative_917	0.39	0.57	0.10	0.23
T. cruzi-negative_918	0.09	0.31	0.16	0.31
T. cruzi-negative_919	0.11	0.36	0.18	0.29
T. cruzi-negative_920	0.13	0.45	0.25	0.40
T. cruzi-negative_921	0.10	0.29	0.07	0.19
T. cruzi-negative_922	0.07	0.28	0.07	0.22
T. cruzi-negative_923	0.01	0.19	0.10	0.23
T. cruzi-negative_924	0.04	0.26	0.15	0.32
T. cruzi-negative_925	0.33	0.32	0.18	0.36
T. cruzi-negative_926	0.12	0.48	0.26	0.34
T. cruzi-negative_927	0.19	0.62	0.14	0.27
T. cruzi-negative_928	0.13	0.43	0.12	0.27
T. cruzi-negative_929	0.04	0.32	0.15	0.29
T. cruzi-negative_930	0.08	0.43	0.08	0.17
T. cruzi-negative_931	0.13	0.45	0.07	0.13
T. cruzi-negative_932	0.06	0.36	0.15	0.26
T. cruzi-negative_933	0.07	0.29	0.11	0.28
T. cruzi-negative_934	0.23	0.36	0.35	0.29
T. cruzi-negative_935	0.05	0.37	0.16	0.30
T. cruzi-negative_936	0.12	0.49	0.10	0.20
T. cruzi-negative_937	0.03	0.30	0.17	0.31
T. cruzi-negative_938	0.11	0.45	0.10	0.21
T. cruzi-negative_939	0.11	0.47	0.19	0.31
T. cruzi-negative_940	0.11	0.44	0.16	0.32
T. cruzi-negative_941	0.04	0.36	0.24	0.47
T. cruzi-negative_942	0.09	0.51	0.14	0.23
T. cruzi-negative_943	0.07	0.41	0.15	0.23
T. cruzi-negative_944	0.03	0.25	0.10	0.16
T. cruzi-negative_945	0.19	0.48	0.14	0.17
T. cruzi-negative_946	0.08	0.40	0.10	0.21
T. cruzi-negative_947	0.14	0.48	0.19	0.27

*T. cruz*i-negative_948	0.15	0.47	0.16	0.22
*T. cruz*i-negative_949	0.21	0.60	0.14	0.32
*T. cruz*i-negative_950	0.09	0.64	0.12	0.13
*T. cruz*i-negative_951	0.07	0.38	0.13	0.21
*T. cruz*i-negative_952	0.19	0.56	0.11	0.19
*T. cruz*i-negative_953	0.18	0.36	0.28	0.29
*T. cruz*i-negative_954	0.01	0.24	0.21	0.30
*T. cruz*i-negative_955	0.13	0.42	0.29	0.24
*T. cruz*i-negative_956	0.01	0.25	0.13	0.17
*T. cruz*i-negative_957	0.07	0.39	0.26	0.26
*T. cruz*i-negative_958	0.14	0.43	0.18	0.15
*T. cruz*i-negative_959	0.07	0.43	0.18	0.21
*T. cruz*i-negative_960	0.18	0.50	0.25	0.22
*T. cruz*i-negative_961	0.17	0.42	0.22	0.18
*T. cruz*i-negative_962	0.10	0.46	0.20	0.21
*T. cruz*i-negative_963	0.16	0.47	0.13	0.12
*T. cruz*i-negative_964	0.15	0.52	0.12	0.12
*T. cruz*i-negative_965	0.21	0.52	0.19	0.19
*T. cruz*i-negative_966	0.19	0.35	0.27	0.27
*T. cruz*i-negative_967	0.15	0.58	0.28	0.25
*T. cruz*i-negative_968	0.18	0.53	0.18	0.13
*T. cruz*i-negative_969	0.32	0.59	0.21	0.21
*T. cruz*i-negative_970	0.07	0.46	0.17	0.10
*T. cruz*i-negative_971	0.07	0.43	0.27	0.17
*T. cruz*i-negative_972	0.08	0.43	0.10	0.09
*T. cruz*i-negative_973	0.44	0.61	0.56	0.73
*T. cruz*i-negative_974	0.25	0.22	0.18	0.14
*T. cruz*i-negative_975	0.27	0.24	0.26	0.22
*T. cruz*i-negative_976	0.30	0.21	0.22	0.21
*T. cruz*i-negative_977	0.33	0.41	0.22	0.23
*T. cruz*i-negative_978	0.27	0.25	0.19	0.18
*T. cruz*i-negative_979	0.27	0.22	0.21	0.21
*T. cruz*i-negative_980	0.23	0.26	0.22	0.23
*T. cruz*i-negative_981	0.26	0.21	0.23	0.24
*T. cruz*i-negative_982	0.36	0.28	0.29	0.27
*T. cruz*i-negative_983	0.26	0.13	0.21	0.17
*T. cruz*i-negative_984	0.23	0.14	0.17	0.17
*T. cruz*i-negative_985	0.14	0.16	0.14	0.09
*T. cruz*i-negative_986	0.18	0.15	0.14	0.13
*T. cruz*i-negative_987	0.26	0.23	0.20	0.21
*T. cruz*i-negative_988	0.29	0.23	0.21	0.23
*T. cruz*i-negative_989	0.18	0.14	0.13	0.15

*T. cruz*i-negative_990	0.22	0.17	0.15	0.13
*T. cruz*i-negative_991	0.18	0.14	0.18	0.13
*T. cruz*i-negative_992	0.24	0.19	0.21	0.18
*T. cruz*i-negative_993	0.17	0.22	0.26	0.20
*T. cruz*i-negative_994	0.33	0.22	0.21	0.20
*T. cruz*i-negative_995	0.28	0.26	0.24	0.27
*T. cruz*i-negative_996	0.27	0.26	0.23	0.21
*T. cruz*i-negative_997	0.29	0.30	0.26	0.27
*T. cruz*i-negative_998	0.18	0.14	0.16	0.12
*T. cruz*i-negative_999	0.27	0.22	0.31	0.22
*T. cruz*i-negative_1000	0.24	0.15	0.21	0.19
*T. cruz*i-negative_1001	0.23	0.28	0.26	0.23
*T. cruz*i-negative_1002	0.66	0.21	0.23	0.22
*T. cruz*i-negative_1003	0.42	0.34	0.31	0.37
*T. cruz*i-negative_1004	0.23	0.20	0.21	0.19
*T. cruz*i-negative_1005	0.22	0.23	0.19	0.22
*T. cruz*i-negative_1006	0.20	0.17	0.15	0.17
*T. cruz*i-negative_1007	0.16	0.14	0.21	0.12
*T. cruz*i-negative_1008	0.35	0.17	0.26	0.29
*T. cruz*i-negative_1009	0.28	0.25	0.22	0.31
*T. cruz*i-negative_1010	0.43	0.32	0.45	0.30
*T. cruz*i-negative_1011	0.44	0.25	0.22	0.19
*T. cruz*i-negative_1012	0.27	0.23	0.23	0.25
*T. cruz*i-negative_1013	0.31	0.26	0.24	0.27
*T. cruz*i-negative_1014	0.29	0.19	0.27	0.19
*T. cruz*i-negative_1015	0.21	0.21	0.30	0.24
*T. cruz*i-negative_1016	0.25	0.20	0.21	0.26
*T. cruz*i-negative_1017	0.24	0.24	0.21	0.29
*T. cruz*i-negative_1018	0.26	0.19	0.21	0.20
*T. cruz*i-negative_1019	0.37	0.26	0.23	0.29
*T. cruz*i-negative_1020	0.41	0.30	0.26	0.36
*T. cruz*i-negative_1021	0.18	0.15	0.12	0.19
*T. cruz*i-negative_1022	0.38	0.28	0.28	0.26
*T. cruz*i-negative_1023	0.29	0.16	0.26	0.30
*T. cruz*i-negative_1024	0.17	0.09	0.15	0.17
*T. cruz*i-negative_1025	0.22	0.20	0.19	0.23
*T. cruz*i-negative_1026	0.25	0.22	0.18	0.18
*T. cruz*i-negative_1027	0.33	0.25	0.27	0.23
*T. cruz*i-negative_1028	0.15	0.11	0.15	0.13
*T. cruz*i-negative_1029	0.25	0.22	0.23	0.27
*T. cruz*i-negative_1030	0.13	0.09	0.15	0.13
*T. cruz*i-negative_1031	0.20	0.16	0.27	0.20
*T. cruz*i-negative_1032	0.30	0.13	0.19	0.24

T. cruzi-negative_1033	0.21	0.21	0.19	0.24
T. cruzi-negative_1034	0.25	0.18	0.15	0.19
T. cruzi-negative_1035	0.41	0.25	0.32	0.38
T. cruzi-negative_1036	0.24	0.17	0.25	0.25
T. cruzi-negative_1037	0.46	0.29	0.36	0.43
T. cruzi-negative_1038	0.39	0.25	0.31	0.26
T. cruzi-negative_1039	0.22	0.16	0.25	0.18
T. cruzi-negative_1040	0.29	0.24	0.31	0.47
T. cruzi-negative_1041	0.34	0.23	0.24	0.38
T. cruzi-negative_1042	0.21	0.14	0.15	0.26
T. cruzi-negative_1043	0.65	0.28	0.39	0.48
T. cruzi-negative_1044	0.27	0.28	0.25	0.33
T. cruzi-negative_1045	0.21	0.13	0.16	0.20
T. cruzi-negative_1046	0.18	0.13	0.15	0.16
T. cruzi-negative_1047	0.29	0.19	0.34	0.31
T. cruzi-negative_1048	0.31	0.15	0.25	0.30
T. cruzi-negative_1049	0.17	0.15	0.19	0.28
T. cruzi-negative_1050	0.45	0.30	0.32	0.36
T. cruzi-negative_1051	0.39	0.15	0.21	0.24
T. cruzi-negative_1052	0.33	0.03	0.19	0.01
T. cruzi-negative_1053	0.40	0.28	0.31	0.42
T. cruzi-negative_1054	0.27	0.19	0.14	0.25
T. cruzi-negative_1055	0.35	0.18	0.27	0.34
T. cruzi-negative_1056	0.72	0.12	0.16	0.23
T. cruzi-negative_1057	0.13	0.14	0.02	0.17
T. cruzi-negative_1058	0.29	0.21	0.14	0.28
T. cruzi-negative_1059	0.33	0.13	0.04	0.28
T. cruzi-negative_1060	0.40	0.23	0.17	0.40
T. cruzi-negative_1061	0.17	0.19	0.13	0.23
T. cruzi-negative_1062	0.20	0.19	0.09	0.27
T. cruzi-negative_1063	0.25	0.20	0.08	0.18
T. cruzi-negative_1064	0.21	0.18	0.09	0.17
T. cruzi-negative_1065	0.35	0.29	0.13	0.29
T. cruzi-negative_1066	0.31	0.26	0.11	0.38
T. cruzi-negative_1067	0.30	0.23	0.07	0.33
T. cruzi-negative_1068	0.39	0.32	0.18	0.39
T. cruzi-negative_1069	0.44	0.32	0.31	0.44
T. cruzi-negative_1070	0.35	0.18	0.13	0.28
T. cruzi-negative_1071	0.32	0.24	0.10	0.17
T. cruzi-negative_1072	0.24	0.21	0.07	0.31
T. cruzi-negative_1073	0.23	0.19	0.08	0.18
T. cruzi-negative_1074	0.26	0.16	0.04	0.47

*T. cruz*i-negative_1075	0.40	0.24	0.19	0.40
*T. cruz*i-negative_1076	0.15	0.12	0.01	0.19
*T. cruz*i-negative_1077	0.30	0.43	0.55	0.34
*T. cruz*i-negative_1078	0.27	0.10	0.09	0.25
*T. cruz*i-negative_1079	0.49	0.27	0.20	0.27
*T. cruz*i-negative_1080	0.15	0.11	0.02	0.15
*T. cruz*i-negative_1081	0.30	0.17	0.11	0.25
*T. cruz*i-negative_1082	0.29	0.19	0.12	0.25
*T. cruz*i-negative_1083	0.29	0.20	0.13	0.24
*T. cruz*i-negative_1084	0.28	0.17	0.10	0.17
*T. cruz*i-negative_1085	0.21	0.11	0.05	0.22
*T. cruz*i-negative_1086	0.25	0.15	0.04	0.22
*T. cruz*i-negative_1087	0.29	0.15	0.11	0.19
*T. cruz*i-negative_1088	0.31	0.17	0.08	0.17
*T. cruz*i-negative_1089	0.45	0.30	0.29	0.31
*T. cruz*i-negative_1090	0.15	0.09	0.12	0.15
*T. cruz*i-negative_1091	0.27	0.15	0.07	0.17
*T. cruz*i-negative_1092	0.49	0.24	0.22	0.35
*T. cruz*i-negative_1093	0.34	0.29	0.15	0.28
*T. cruz*i-negative_1094	0.49	0.23	0.20	0.30
*T. cruz*i-negative_1095	0.26	0.12	0.05	0.17
*T. cruz*i-negative_1096	0.17	0.12	0.01	0.13
*T. cruz*i-negative_1097	0.46	0.23	0.16	0.31
*T. cruz*i-negative_1098	0.32	0.17	0.08	0.24
*T. cruz*i-negative_1099	0.41	0.20	0.11	0.29
*T. cruz*i-negative_1100	0.24	0.16	0.11	0.19
*T. cruz*i-negative_1101	0.23	0.17	0.03	0.29
*T. cruz*i-negative_1102	0.53	0.64	0.63	0.52
*T. cruz*i-negative_1103	0.28	0.15	0.07	0.18
*T. cruz*i-negative_1104	0.30	0.22	0.09	0.32
*T. cruz*i-negative_1105	0.32	0.25	0.11	0.24
*T. cruz*i-negative_1106	0.35	0.35	0.09	0.24
*T. cruz*i-negative_1107	0.23	0.21	0.06	0.19
*T. cruz*i-negative_1108	0.29	0.24	0.14	0.24
*T. cruz*i-negative_1109	0.21	0.12	0.11	0.19
*T. cruz*i-negative_1110	0.32	0.26	0.11	0.26
*T. cruz*i-negative_1111	0.20	0.12	0.02	0.11
*T. cruz*i-negative_1112	0.33	0.23	0.13	0.23
*T. cruz*i-negative_1113	0.30	0.25	0.10	0.26
*T. cruz*i-negative_1114	0.21	0.12	0.04	0.17
*T. cruz*i-negative_1115	0.35	0.21	0.07	0.33
*T. cruz*i-negative_1116	0.33	0.16	0.15	0.23
*T. cruz*i-negative_1117	0.26	0.16	0.08	0.28

*T. cruz*i-negative_1118	0.26	0.15	0.06	0.18
*T. cruz*i-negative_1119	0.20	0.14	0.02	0.15
*T. cruz*i-negative_1120	0.29	0.16	0.18	0.18
*T. cruz*i-negative_1121	0.34	0.17	0.10	0.37
*T. cruz*i-negative_1122	0.26	0.15	0.12	0.17
*T. cruz*i-negative_1123	0.31	0.17	0.13	0.30
*T. cruz*i-negative_1124	0.30	0.17	0.12	0.28
*T. cruz*i-negative_1125	0.72	0.64	0.41	0.55
*T. cruz*i-negative_1126	0.38	0.19	0.09	0.21
*T. cruz*i-negative_1127	0.20	0.13	0.04	0.15
*T. cruz*i-negative_1128	0.33	0.18	0.10	0.21
*T. cruz*i-negative_1129	0.27	0.18	0.10	0.22
*T. cruz*i-negative_1130	0.26	0.19	0.06	0.17
*T. cruz*i-negative_1131	0.41	0.25	0.13	0.29
*T. cruz*i-negative_1132	0.44	0.24	0.15	0.34
*T. cruz*i-negative_1133	0.29	0.25	0.07	0.29
*T. cruz*i-negative_1134	0.18	0.15	0.03	0.16
*T. cruz*i-negative_1135	0.31	0.19	0.06	0.23
*T. cruz*i-negative_1136	0.38	0.28	0.15	0.22
*T. cruz*i-negative_1137	0.52	0.35	0.22	0.37
*T. cruz*i-negative_1138	0.46	0.25	0.24	0.30
*T. cruz*i-negative_1139	0.43	0.32	0.16	0.29
*T. cruz*i-negative_1140	0.30	0.32	0.21	0.34
*T. cruz*i-negative_1141	0.41	0.39	0.49	0.33
*T. cruz*i-negative_1142	0.32	0.35	0.30	0.39
*T. cruz*i-negative_1143	0.25	0.36	0.19	0.38
*T. cruz*i-negative_1144	0.42	0.47	0.34	0.60
*T. cruz*i-negative_1145	0.34	0.34	0.23	0.28
*T. cruz*i-negative_1146	0.39	0.30	0.25	0.31
*T. cruz*i-negative_1147	0.37	0.39	0.26	0.36
*T. cruz*i-negative_1148	0.46	0.48	0.29	0.41
*T. cruz*i-negative_1149	0.34	0.35	0.31	0.29
*T. cruz*i-negative_1150	0.35	0.33	0.33	0.43
*T. cruz*i-negative_1151	0.39	0.45	0.30	0.40
*T. cruz*i-negative_1152	0.47	0.50	0.42	0.50
*T. cruz*i-negative_1153	0.38	0.31	0.45	0.34
*T. cruz*i-negative_1154	0.25	0.28	0.24	0.30
*T. cruz*i-negative_1155	0.36	0.34	0.21	0.27
*T. cruz*i-negative_1156	0.37	0.31	0.31	0.39
*T. cruz*i-negative_1157	0.49	0.47	0.55	0.53
*T. cruz*i-negative_1158	0.39	0.51	0.34	0.48
*T. cruz*i-negative_1159	0.40	0.48	0.43	0.50

T. cruzi-negative_1160	0.64	0.72	0.51	0.72
T. cruzi-negative_1161	0.25	0.28	0.21	0.31
T. cruzi-negative_1162	0.29	0.25	0.21	0.30
T. cruzi-negative_1163	0.45	0.55	0.38	0.70
T. cruzi-negative_1164	0.33	0.30	0.21	0.34
T. cruzi-negative_1165	0.24	0.13	0.16	0.15
T. cruzi-negative_1166	0.32	0.35	0.25	0.40
T. cruzi-negative_1167	0.35	0.38	0.26	0.34
T. cruzi-negative_1168	0.46	0.58	0.39	0.49
T. cruzi-negative_1169	0.31	0.30	0.28	0.37
T. cruzi-negative_1170	0.41	0.33	0.32	0.37
T. cruzi-negative_1171	0.24	0.24	0.38	0.36
T. cruzi-negative_1172	0.22	0.11	0.19	0.20
T. cruzi-negative_1173	0.34	0.34	0.38	0.45
T. cruzi-negative_1174	0.35	0.30	0.40	0.33
T. cruzi-negative_1175	0.32	0.38	0.23	0.33
T. cruzi-negative_1176	0.42	0.56	0.43	0.56
T. cruzi-negative_1177	0.89	0.49	0.25	0.29
T. cruzi-negative_1178	0.28	0.31	0.25	0.31
T. cruzi-negative_1179	0.33	0.43	0.28	0.44
T. cruzi-negative_1180	0.19	0.18	0.15	0.25
T. cruzi-negative_1181	0.39	0.50	0.32	0.47
T. cruzi-negative_1182	0.37	0.43	0.31	0.43
T. cruzi-negative_1183	0.39	0.49	0.31	0.46
T. cruzi-negative_1184	0.45	0.59	0.47	0.66
T. cruzi-negative_1185	0.24	0.29	0.23	0.32
T. cruzi-negative_1186	0.30	0.37	0.28	0.38
T. cruzi-negative_1187	0.43	0.53	0.54	0.65
T. cruzi-negative_1188	0.41	0.39	0.39	0.44
T. cruzi-negative_1189	0.35	0.32	0.21	0.39
T. cruzi-negative_1190	0.48	0.54	0.42	0.65
T. cruzi-negative_1191	0.34	0.53	0.08	0.07
T. cruzi-negative_1192	0.49	0.55	0.45	0.57
T. cruzi-negative_1193	0.31	0.39	0.37	0.43
T. cruzi-negative_1194	0.32	0.49	0.39	0.43
T. cruzi-negative_1195	0.31	0.34	0.35	0.39
T. cruzi-negative_1196	0.28	0.29	0.31	0.42
T. cruzi-negative_1197	0.34	0.47	0.31	0.42
T. cruzi-negative_1198	0.25	0.14	0.21	0.18
T. cruzi-negative_1199	0.35	0.31	0.35	0.34
T. cruzi-negative_1200	0.44	0.52	0.58	0.61
T. cruzi-negative_1201	0.33	0.38	0.42	0.50
T. cruzi-negative_1202	0.20	0.28	0.22	0.30

T. cruzi-negative_1203	0.30	0.38	0.40	0.36
T. cruzi-negative_1204	0.30	0.44	0.37	0.40
T. cruzi-negative_1205	0.37	0.42	0.52	0.43
T. cruzi-negative_1206	0.40	0.48	0.46	0.42
T. cruzi-negative_1207	0.33	0.38	0.39	0.54
T. cruzi-negative_1208	0.39	0.44	0.48	0.59
T. cruzi-negative_1209	0.31	0.37	0.34	0.45
T. cruzi-negative_1210	0.22	0.31	0.30	0.32
T. cruzi-negative_1211	0.32	0.37	0.38	0.33
T. cruzi-negative_1212	0.21	0.27	0.29	0.30
T. cruzi-negative_1213	0.20	0.25	0.24	0.34
T. cruzi-negative_1214	0.30	0.30	0.29	0.31
T. cruzi-negative_1215	0.40	0.46	0.57	0.52
T. cruzi-negative_1216	0.24	0.33	0.30	0.34
T. cruzi-negative_1217	0.34	0.46	0.37	0.51
T. cruzi-negative_1218	0.33	0.44	0.41	0.41
T. cruzi-negative_1219	0.42	0.53	0.51	0.47
T. cruzi-negative_1220	0.30	0.50	0.43	0.43
T. cruzi-negative_1221	0.52	0.48	0.48	0.52
T. cruzi-negative_1222	0.53	0.56	0.42	0.51
T. cruzi-negative_1223	0.45	0.43	0.51	0.43
T. cruzi-negative_1224	0.36	0.11	0.30	0.20
T. cruzi-negative_1225	0.18	0.09	0.23	0.22
T. cruzi-negative_1226	0.26	0.12	0.20	0.20
T. cruzi-negative_1227	0.21	0.29	0.37	0.37
T. cruzi-negative_1228	0.22	0.14	0.20	0.33
T. cruzi-negative_1229	0.39	0.17	0.22	0.21
T. cruzi-negative_1230	0.49	0.22	0.31	0.31
T. cruzi-negative_1231	0.30	0.15	0.22	0.26
T. cruzi-negative_1232	0.39	0.24	0.35	0.37
T. cruzi-negative_1233	0.26	0.16	0.21	0.22
T. cruzi-negative_1234	0.30	0.18	0.23	0.27
T. cruzi-negative_1235	0.26	0.09	0.16	0.16
T. cruzi-negative_1236	0.31	0.17	0.26	0.24
T. cruzi-negative_1237	0.35	0.26	0.22	0.22
T. cruzi-negative_1238	0.23	0.07	0.30	0.16
T. cruzi-negative_1239	0.39	0.22	0.30	0.29
T. cruzi-negative_1240	0.29	0.13	0.20	0.21
T. cruzi-negative_1241	0.33	0.17	0.34	0.39
T. cruzi-negative_1242	0.34	0.21	0.25	0.28
T. cruzi-negative_1243	0.25	0.06	0.11	0.16
T. cruzi-negative_1244	0.52	0.43	0.57	0.52

T. cruzi-negative_1245	0.27	0.11	0.49	0.17
T. cruzi-negative_1246	0.29	0.08	0.15	0.17
T. cruzi-negative_1247	0.26	0.16	0.15	0.17
T. cruzi-negative_1248	0.23	0.07	0.13	0.13
T. cruzi-negative_1249	0.16	0.03	0.13	0.23
T. cruzi-negative_1250	0.21	0.08	0.21	0.21
T. cruzi-negative_1251	0.24	0.09	0.17	0.17
T. cruzi-negative_1252	0.26	0.19	0.23	0.21
T. cruzi-negative_1253	0.32	0.14	0.16	0.18
T. cruzi-negative_1254	0.43	0.19	0.29	0.26
T. cruzi-negative_1255	0.21	0.05	0.21	0.26
T. cruzi-negative_1256	0.37	0.16	0.24	0.26
T. cruzi-negative_1257	0.16	0.02	0.17	0.17
T. cruzi-negative_1258	0.27	0.12	0.21	0.21
T. cruzi-negative_1259	0.19	0.08	0.28	0.16
T. cruzi-negative_1260	0.37	0.15	0.23	0.21
T. cruzi-negative_1261	0.30	0.05	0.13	0.17
T. cruzi-negative_1262	0.31	0.20	0.16	0.20
T. cruzi-negative_1263	0.27	0.13	0.17	0.21
T. cruzi-negative_1264	0.31	0.13	0.28	0.22
T. cruzi-negative_1265	0.38	0.21	0.27	0.27
T. cruzi-negative_1266	0.19	0.10	0.21	0.18
T. cruzi-negative_1267	0.17	0.15	0.13	0.17
T. cruzi-negative_1268	0.42	0.21	0.26	0.31
T. cruzi-negative_1269	0.33	0.15	0.16	0.18
T. cruzi-negative_1270	0.36	0.16	0.20	0.36
T. cruzi-negative_1271	0.30	0.10	0.17	0.21
T. cruzi-negative_1272	0.27	0.13	0.24	0.20
T. cruzi-negative_1273	0.24	0.10	0.22	0.17
T. cruzi-negative_1274	0.37	0.23	0.30	0.34
T. cruzi-negative_1275	0.22	0.06	0.16	0.15
T. cruzi-negative_1276	0.16	0.10	0.15	0.14
T. cruzi-negative_1277	0.26	0.11	0.12	0.14
T. cruzi-negative_1278	0.27	0.10	0.16	0.23
T. cruzi-negative_1279	0.24	0.09	0.30	0.22
T. cruzi-negative_1280	0.26	0.09	0.13	0.16
T. cruzi-negative_1281	0.25	0.06	0.14	0.16
T. cruzi-negative_1282	0.31	0.24	0.25	0.33
T. cruzi-negative_1283	0.19	0.06	0.09	0.13
T. cruzi-negative_1284	0.36	0.24	0.30	0.31
T. cruzi-negative_1285	0.40	0.17	0.19	0.20
T. cruzi-negative_1286	0.37	0.16	0.16	0.20
T. cruzi-negative_1287	0.19	0.06	0.09	0.15

T. cruzi-negative_1288	0.38	0.29	0.27	0.36
T. cruzi-negative_1289	0.29	0.12	0.19	0.22
T. cruzi-negative_1290	0.26	0.13	0.14	0.18
T. cruzi-negative_1291	0.27	0.15	0.15	0.20
T. cruzi-negative_1292	0.19	0.03	0.12	0.10
T. cruzi-negative_1293	0.38	0.24	0.23	0.22
T. cruzi-negative_1294	0.29	0.20	0.13	0.19
T. cruzi-negative_1295	0.33	0.19	0.28	0.27
T. cruzi-negative_1296	0.23	0.15	0.18	0.20
T. cruzi-negative_1297	0.22	0.10	0.11	0.14
T. cruzi-negative_1298	0.29	0.32	0.14	0.19
T. cruzi-negative_1299	0.27	0.15	0.17	0.20
T. cruzi-negative_1300	0.27	0.37	0.35	0.29
T. cruzi-negative_1301	0.59	0.11	0.16	0.16
T. cruzi-negative_1302	0.35	0.13	0.19	0.21
T. cruzi-negative_1303	0.31	0.19	0.23	0.29
T. cruzi-negative_1304	0.33	0.16	0.22	0.24
T. cruzi-negative_1305	0.49	0.26	0.30	0.31
T. cruzi-negative_1306	0.46	0.28	0.32	0.31
T. cruzi-negative_1307	0.32	0.17	0.14	0.20
T. cruzi-negative_1308	0.16	0.17	0.17	0.18
T. cruzi-negative_1309	0.15	0.18	0.15	0.23
T. cruzi-negative_1310	0.12	0.15	0.21	0.17
T. cruzi-negative_1311	0.22	0.16	0.17	0.45
T. cruzi-negative_1312	0.20	0.19	0.21	0.25
T. cruzi-negative_1313	0.17	0.13	0.27	0.20
T. cruzi-negative_1314	0.22	0.18	0.16	0.22
T. cruzi-negative_1315	0.26	0.30	0.23	0.23
T. cruzi-negative_1316	0.13	0.11	0.17	0.18
T. cruzi-negative_1317	0.11	0.20	0.13	0.17
T. cruzi-negative_1318	0.24	0.18	0.24	0.22
T. cruzi-negative_1319	0.10	0.10	0.13	0.35
T. cruzi-negative_1320	0.14	0.25	0.28	0.31
T. cruzi-negative_1321	0.15	0.12	0.14	0.32
T. cruzi-negative_1322	0.17	0.19	0.11	0.18
T. cruzi-negative_1323	0.14	0.16	0.09	0.14
T. cruzi-negative_1324	0.17	0.16	0.13	0.26
T. cruzi-negative_1325	0.20	0.22	0.19	0.26
T. cruzi-negative_1326	0.07	0.12	0.12	0.15
T. cruzi-negative_1327	0.15	0.14	0.14	0.28
T. cruzi-negative_1328	0.17	0.17	0.22	0.25
T. cruzi-negative_1329	0.25	0.23	0.17	0.31

T. cruzi-negative_1330	0.17	0.16	0.10	0.18
T. cruzi-negative_1331	0.21	0.25	0.13	0.23
T. cruzi-negative_1332	0.11	0.12	0.08	0.29
T. cruzi-negative_1333	0.20	0.21	0.18	0.32
T. cruzi-negative_1334	0.15	0.14	0.15	0.18
T. cruzi-negative_1335	0.13	0.15	0.11	0.22
T. cruzi-negative_1336	0.15	0.13	0.17	0.20
T. cruzi-negative_1337	0.35	0.31	0.13	0.28
T. cruzi-negative_1338	0.24	0.25	0.17	0.22
T. cruzi-negative_1339	0.10	0.16	0.19	0.10
T. cruzi-negative_1340	0.17	0.14	0.13	0.14
T. cruzi-negative_1341	0.30	0.21	0.12	0.25
T. cruzi-negative_1342	0.20	0.21	0.19	0.25
T. cruzi-negative_1343	0.43	0.15	0.11	0.21
T. cruzi-negative_1344	0.66	0.13	0.08	0.24
T. cruzi-negative_1345	0.15	0.12	0.06	0.17
T. cruzi-negative_1346	0.19	0.24	0.15	0.24
T. cruzi-negative_1347	0.15	0.18	0.11	0.15
T. cruzi-negative_1348	0.16	0.14	0.11	0.22
T. cruzi-negative_1349	0.15	0.14	0.14	0.21
T. cruzi-negative_1350	0.37	0.30	0.26	0.23
T. cruzi-negative_1351	0.15	0.14	0.09	0.14
T. cruzi-negative_1352	0.23	0.08	0.09	0.12
T. cruzi-negative_1353	0.19	0.17	0.12	0.24
T. cruzi-negative_1354	0.13	0.19	0.11	0.12
T. cruzi-negative_1355	0.14	0.14	0.15	0.15
T. cruzi-negative_1356	0.12	0.17	0.13	0.23
T. cruzi-negative_1357	0.08	0.10	0.13	0.12
T. cruzi-negative_1358	0.19	0.20	0.19	0.29
T. cruzi-negative_1359	0.14	0.12	0.09	0.27
T. cruzi-negative_1360	0.38	0.22	0.16	0.31
T. cruzi-negative_1361	0.22	0.18	0.11	0.28
T. cruzi-negative_1362	0.24	0.25	0.16	0.28
T. cruzi-negative_1363	0.21	0.26	0.14	0.31
T. cruzi-negative_1364	0.12	0.16	0.10	0.23
T. cruzi-negative_1365	0.16	0.13	0.09	0.19
T. cruzi-negative_1366	0.19	0.19	0.15	0.20
T. cruzi-negative_1367	0.20	0.18	0.13	0.24
T. cruzi-negative_1368	0.19	0.16	0.15	0.26
T. cruzi-negative_1369	0.39	0.30	0.26	0.49
T. cruzi-negative_1370	0.28	0.29	0.19	0.40
T. cruzi-negative_1371	0.22	0.33	0.20	0.41
T. cruzi-negative_1372	0.22	0.20	0.13	0.52

T. cruzi-negative_1373	0.32	0.32	0.28	0.48
T. cruzi-negative_1374	0.18	0.12	0.09	0.29
T. cruzi-negative_1375	0.17	0.16	0.16	0.26
T. cruzi-negative_1376	0.24	0.28	0.11	0.36
T. cruzi-negative_1377	0.18	0.19	0.12	0.23
T. cruzi-negative_1378	0.36	0.27	0.12	0.28
T. cruzi-negative_1379	0.16	0.18	0.09	0.30
T. cruzi-negative_1380	0.19	0.22	0.13	0.72
T. cruzi-negative_1381	0.17	0.17	0.12	0.30
T. cruzi-negative_1382	0.23	0.19	0.13	0.46
T. cruzi-negative_1383	0.25	0.19	0.25	0.40
T. cruzi-negative_1384	0.16	0.16	0.10	0.30
T. cruzi-negative_1385	0.15	0.19	0.11	0.21
T. cruzi-negative_1386	0.41	0.38	0.23	0.61
T. cruzi-negative_1387	0.15	0.20	0.10	0.31
T. cruzi-negative_1388	0.07	0.37	0.27	0.43
T. cruzi-negative_1389	0.37	0.29	0.29	0.65
T. cruzi-negative_1390	0.25	0.41	0.20	0.45
T. cruzi-negative_1391	0.09	0.19	0.12	0.19
T. cruzi-negative_1392	0.33	0.39	0.42	0.46
T. cruzi-negative_1393	0.17	0.21	0.23	0.28
T. cruzi-negative_1394	0.31	0.20	0.37	0.21
T. cruzi-negative_1395	0.44	0.18	0.28	0.38
T. cruzi-negative_1396	0.20	0.47	0.47	0.29
T. cruzi-negative_1397	0.27	0.43	0.23	0.37
T. cruzi-negative_1398	0.22	0.33	0.28	0.26
T. cruzi-negative_1399	0.14	0.30	0.24	0.31
T. cruzi-negative_1400	0.19	0.25	0.23	0.29
T. cruzi-negative_1401	0.23	0.24	0.25	0.25
T. cruzi-negative_1402	0.22	0.20	0.27	0.34
T. cruzi-negative_1403	0.19	0.13	0.20	0.20
T. cruzi-negative_1404	0.53	0.40	0.50	0.41
T. cruzi-negative_1405	0.22	0.24	0.18	0.24
T. cruzi-negative_1406	0.02	0.24	0.27	0.41
T. cruzi-negative_1407	0.10	0.19	0.18	0.16
T. cruzi-negative_1408	0.30	0.24	0.22	0.28
T. cruzi-negative_1409	0.28	0.19	0.21	0.14
T. cruzi-negative_1410	0.43	0.16	0.39	0.13
T. cruzi-negative_1411	0.32	0.26	0.19	0.21
T. cruzi-negative_1412	0.39	0.32	0.27	0.28
T. cruzi-negative_1413	0.29	0.38	0.45	0.34
T. cruzi-negative_1414	0.37	0.35	0.26	0.39

*T. cruz*i-negative_1415	0.21	0.19	0.23	0.22
*T. cruz*i-negative_1416	0.24	0.27	0.43	0.29
*T. cruz*i-negative_1417	0.28	0.26	0.29	0.29
*T. cruz*i-negative_1418	0.12	0.15	0.15	0.11
*T. cruz*i-negative_1419	0.23	0.13	0.26	0.13
*T. cruz*i-negative_1420	0.30	0.35	0.21	0.26
*T. cruz*i-negative_1421	0.47	0.45	0.55	0.34
*T. cruz*i-negative_1422	0.25	0.20	0.20	0.18
*T. cruz*i-negative_1423	0.23	0.17	0.17	0.23
*T. cruz*i-negative_1424	0.25	0.25	0.26	0.26
*T. cruz*i-negative_1425	0.30	0.23	0.29	0.28
*T. cruz*i-negative_1426	0.25	0.26	0.30	0.32
*T. cruz*i-negative_1427	0.28	0.21	0.25	0.30
*T. cruz*i-negative_1428	0.09	0.21	0.16	0.13
*T. cruz*i-negative_1429	0.22	0.28	0.26	0.16
*T. cruz*i-negative_1430	0.31	0.38	0.36	0.36
*T. cruz*i-negative_1431	0.22	0.29	0.24	0.42
*T. cruz*i-negative_1432	0.29	0.20	0.20	0.30
*T. cruz*i-negative_1433	0.35	0.20	0.27	0.36
*T. cruz*i-negative_1434	0.20	0.02	0.18	0.36
*T. cruz*i-negative_1435	0.29	0.24	0.25	0.35
*T. cruz*i-negative_1436	0.19	0.31	0.22	0.30
*T. cruz*i-negative_1437	0.32	0.35	0.29	0.36
*T. cruz*i-negative_1438	0.30	0.28	0.22	0.22
*T. cruz*i-negative_1439	0.20	0.23	0.20	0.30
*T. cruz*i-negative_1440	0.23	0.23	0.24	0.32
*T. cruz*i-negative_1441	0.14	0.15	0.17	0.25
*T. cruz*i-negative_1442	0.11	0.09	0.15	0.17
*T. cruz*i-negative_1443	0.15	0.11	0.15	0.18
*T. cruz*i-negative_1444	0.28	0.24	0.21	0.19
*T. cruz*i-negative_1445	0.20	0.28	0.33	0.28
*T. cruz*i-negative_1446	0.18	0.27	0.20	0.27
*T. cruz*i-negative_1447	0.27	0.21	0.21	0.28
*T. cruz*i-negative_1448	0.18	0.12	0.16	0.18
*T. cruz*i-negative_1449	0.16	0.17	0.19	0.33
*T. cruz*i-negative_1450	0.17	0.15	0.25	0.27
*T. cruz*i-negative_1451	0.17	0.19	0.17	0.23
*T. cruz*i-negative_1452	0.48	0.31	0.42	0.29
*T. cruz*i-negative_1453	0.18	0.27	0.22	0.39
*T. cruz*i-negative_1454	0.29	0.33	0.33	0.37
*T. cruz*i-negative_1455	0.21	0.22	0.22	0.28
*T. cruz*i-negative_1456	0.17	0.19	0.17	0.22
*T. cruz*i-negative_1457	0.24	0.21	0.24	0.29

T. cruzi-negative_1458	0.14	0.11	0.17	0.19
T. cruzi-negative_1459	0.19	0.22	0.18	0.28
T. cruzi-negative_1460	0.25	0.43	0.42	0.45
T. cruzi-negative_1461	0.32	0.37	0.34	0.49
T. cruzi-negative_1462	0.18	0.24	0.21	0.37
T. cruzi-negative_1463	0.22	0.21	0.19	0.30
T. cruzi-negative_1464	0.12	0.14	0.10	0.16
T. cruzi-negative_1465	0.18	0.16	0.20	0.22
T. cruzi-negative_1466	0.20	0.22	0.21	0.36
T. cruzi-negative_1467	0.29	0.18	0.22	0.25
T. cruzi-negative_1468	0.22	0.44	0.28	0.31
T. cruzi-negative_1469	0.13	0.42	0.15	0.38
T. cruzi-negative_1470	0.18	0.28	0.16	0.31
T. cruzi-negative_1471	0.27	0.39	0.26	0.66
T. cruzi-negative_1472	0.14	0.20	0.17	0.20
T. cruzi-negative_1473	0.17	0.16	0.15	0.51
T. cruzi-negative_1474	0.20	0.25	0.21	0.55
T. cruzi-negative_1475	0.14	0.37	0.15	0.20
T. cruzi-negative_1476	0.29	0.33	0.22	0.27
T. cruzi-negative_1477	0.20	0.27	0.14	0.24
T. cruzi-negative_1478	0.30	0.47	0.41	0.34
T. cruzi-negative_1479	0.21	0.27	0.40	0.20
T. cruzi-negative_1480	0.22	0.35	0.17	0.24
T. cruzi-negative_1481	0.15	0.20	0.11	0.20
T. cruzi-negative_1482	0.18	0.22	0.17	0.19
T. cruzi-negative_1483	0.10	0.21	0.11	0.16
T. cruzi-negative_1484	0.24	0.25	0.21	0.23
T. cruzi-negative_1485	0.17	0.13	0.11	0.11
T. cruzi-negative_1486	0.14	0.36	0.20	0.19
T. cruzi-negative_1487	0.15	0.24	0.14	0.21
T. cruzi-negative_1488	0.10	0.17	0.10	0.15
T. cruzi-negative_1489	0.14	0.23	0.18	0.20
T. cruzi-negative_1490	0.28	0.27	0.12	0.21
T. cruzi-negative_1491	0.10	0.22	0.10	0.16
T. cruzi-negative_1492	0.41	0.45	0.22	0.40
T. cruzi-negative_1493	0.08	0.14	0.06	0.10
T. cruzi-negative_1494	0.13	0.22	0.25	0.21
T. cruzi-negative_1495	0.11	0.56	0.05	0.17
T. cruzi-negative_1496	0.22	0.35	0.18	0.29
T. cruzi-negative_1497	0.16	0.19	0.13	0.19
T. cruzi-negative_1498	0.18	0.21	0.10	0.18
T. cruzi-negative_1499	0.16	0.19	0.18	0.24

*T. cruz*i-negative_1500	0.12	0.17	0.14	0.15
*T. cruz*i-negative_1501	0.16	0.24	0.16	0.20
*T. cruz*i-negative_1502	0.14	0.21	0.17	0.20
*T. cruz*i-negative_1503	0.26	0.26	0.17	0.24
*T. cruz*i-negative_1504	0.14	0.20	0.10	0.15
*T. cruz*i-negative_1505	0.23	0.18	0.12	0.24
*T. cruz*i-negative_1506	0.17	0.16	0.13	0.20
*T. cruz*i-negative_1507	0.24	0.25	0.19	0.21
*T. cruz*i-negative_1508	0.22	0.19	0.17	0.16
*T. cruz*i-negative_1509	0.18	0.26	0.14	0.22
*T. cruz*i-negative_1510	0.28	0.32	0.24	0.30
*T. cruz*i-negative_1511	0.19	0.29	0.18	0.26
*T. cruz*i-negative_1512	0.17	0.18	0.10	0.15
*T. cruz*i-negative_1513	0.18	0.15	0.16	0.21
*T. cruz*i-negative_1514	0.35	0.18	0.17	0.17
*T. cruz*i-negative_1515	0.20	0.18	0.38	0.17
*T. cruz*i-negative_1516	0.25	0.29	0.18	0.25
*T. cruz*i-negative_1517	0.22	0.25	0.16	0.22
*T. cruz*i-negative_1518	0.19	0.25	0.20	0.24
*T. cruz*i-negative_1519	0.11	0.17	0.10	0.14
*T. cruz*i-negative_1520	0.16	0.19	0.12	0.19
*T. cruz*i-negative_1521	0.21	0.23	0.18	0.28
*T. cruz*i-negative_1522	0.25	0.15	0.09	0.17
*T. cruz*i-negative_1523	0.17	0.15	0.21	0.17
*T. cruz*i-negative_1524	0.20	0.15	0.16	0.11
*T. cruz*i-negative_1525	0.17	0.26	0.14	0.21
*T. cruz*i-negative_1526	0.19	0.24	0.17	0.17
*T. cruz*i-negative_1527	0.36	0.47	0.28	0.36
*T. cruz*i-negative_1528	0.60	0.77	0.29	0.50
*T. cruz*i-negative_1529	0.42	0.35	0.22	0.30
*T. cruz*i-negative_1530	0.35	0.23	0.20	0.20
*T. cruz*i-negative_1531	0.18	0.24	0.16	0.20
*T. cruz*i-negative_1532	0.17	0.14	0.13	0.11
*T. cruz*i-negative_1533	0.11	0.27	0.12	0.20
*T. cruz*i-negative_1534	0.26	0.26	0.16	0.19
*T. cruz*i-negative_1535	0.32	0.37	0.27	0.28
*T. cruz*i-negative_1536	0.21	0.23	0.15	0.21
*T. cruz*i-negative_1537	0.20	0.24	0.18	0.23
*T. cruz*i-negative_1538	0.21	0.23	0.20	0.22
*T. cruz*i-negative_1539	0.15	0.20	0.23	0.20
*T. cruz*i-negative_1540	0.09	0.15	0.12	0.16
*T. cruz*i-negative_1541	0.13	0.19	0.11	0.15
*T. cruz*i-negative_1542	0.15	0.13	0.07	0.12

*T. cruz*i-negative_1543	0.28	0.29	0.16	0.27
*T. cruz*i-negative_1544	0.05	0.14	0.08	0.11
*T. cruz*i-negative_1545	0.12	0.15	0.15	0.19
*T. cruz*i-negative_1546	0.18	0.27	0.20	0.28
*T. cruz*i-negative_1547	0.18	0.24	0.29	0.20
*T. cruz*i-negative_1548	0.14	0.20	0.13	0.19
*T. cruz*i-negative_1549	0.18	0.28	0.16	0.19
*T. cruz*i-negative_1550	0.34	0.44	0.25	0.35
*T. cruz*i-negative_1551	0.12	0.18	0.08	0.19
*T. cruz*i-negative_1552	0.13	0.19	0.08	0.11
*T. cruz*i-negative_1553	0.16	0.22	0.14	0.18
*T. cruz*i-negative_1554	0.22	0.35	0.23	0.26
*T. cruz*i-negative_1555	0.23	0.24	0.23	0.27
*T. cruz*i-negative_1556	0.15	0.16	0.16	0.17
*T. cruz*i-negative_1557	0.22	0.26	0.16	0.22
*T. cruz*i-negative_1558	0.27	0.49	0.24	0.27
*T. cruz*i-negative_1559	0.17	0.27	0.21	0.23
*T. cruz*i-negative_1560	0.08	0.09	0.16	0.09
*T. cruz*i-negative_1561	0.24	0.26	0.20	0.21
*T. cruz*i-negative_1562	0.16	0.22	0.29	0.16
*T. cruz*i-negative_1563	0.40	0.50	0.38	0.45
*T. cruz*i-negative_1564	0.30	0.29	0.21	0.21
*T. cruz*i-negative_1565	0.30	0.32	0.29	0.26
*T. cruz*i-negative_1566	0.27	0.30	0.40	0.28
*T. cruz*i-negative_1567	0.17	0.18	0.21	0.16
*T. cruz*i-negative_1568	0.14	0.15	0.16	0.15
*T. cruz*i-negative_1569	0.33	0.31	0.35	0.23
*T. cruz*i-negative_1570	0.20	0.30	0.22	0.20
*T. cruz*i-negative_1571	0.22	0.31	0.21	0.25
*T. cruz*i-negative_1572	0.53	0.29	0.20	0.23
*T. cruz*i-negative_1573	0.16	0.21	0.29	0.25
*T. cruz*i-negative_1574	0.20	0.25	0.21	0.25
*T. cruz*i-negative_1575	0.33	0.20	0.16	0.18
*T. cruz*i-negative_1576	0.13	0.16	0.19	0.15
*T. cruz*i-negative_1577	0.14	0.18	0.15	0.13
*T. cruz*i-negative_1578	0.17	0.23	0.19	0.21
*T. cruz*i-negative_1579	0.26	0.36	0.23	0.27
*T. cruz*i-negative_1580	0.24	0.24	0.21	0.19
*T. cruz*i-negative_1581	0.14	0.19	0.16	0.18
*T. cruz*i-negative_1582	0.15	0.20	0.17	0.16
*T. cruz*i-negative_1583	0.18	0.23	0.36	0.30
*T. cruz*i-negative_1584	0.25	0.27	0.21	0.27

*T. cruz*i-negative_1585	0.18	0.25	0.19	0.23
*T. cruz*i-negative_1586	0.29	0.39	0.35	0.36
*T. cruz*i-negative_1587	0.15	0.17	0.12	0.19
*T. cruz*i-negative_1588	0.15	0.21	0.18	0.18
*T. cruz*i-negative_1589	0.18	0.20	0.20	0.17
*T. cruz*i-negative_1590	0.24	0.25	0.23	0.32
*T. cruz*i-negative_1591	0.26	0.27	0.21	0.32
*T. cruz*i-negative_1592	0.16	0.24	0.20	0.19
*T. cruz*i-negative_1593	0.17	0.24	0.16	0.18
*T. cruz*i-negative_1594	0.22	0.25	0.21	0.20
*T. cruz*i-negative_1595	0.24	0.28	0.27	0.23
*T. cruz*i-negative_1596	0.21	0.18	0.18	0.13
*T. cruz*i-negative_1597	0.16	0.19	0.23	0.19
*T. cruz*i-negative_1598	0.24	0.22	0.22	0.23
*T. cruz*i-negative_1599	0.20	0.12	0.14	0.11
*T. cruz*i-negative_1600	0.19	0.25	0.20	0.21
*T. cruz*i-negative_1601	0.09	0.13	0.11	0.22
*T. cruz*i-negative_1602	0.12	0.16	0.14	0.16
*T. cruz*i-negative_1603	0.23	0.23	0.18	0.17
*T. cruz*i-negative_1604	0.23	0.13	0.13	0.16
*T. cruz*i-negative_1605	0.11	0.16	0.75	0.18
*T. cruz*i-negative_1606	0.21	0.16	0.13	0.20
*T. cruz*i-negative_1607	0.16	0.33	0.26	0.18
*T. cruz*i-negative_1608	0.17	0.23	0.22	0.30
*T. cruz*i-negative_1609	0.20	0.27	0.18	0.33
*T. cruz*i-negative_1610	0.17	0.32	0.17	0.28
*T. cruz*i-negative_1611	0.17	0.15	0.19	0.15
*T. cruz*i-negative_1612	0.13	0.31	0.23	0.15
*T. cruz*i-negative_1613	0.18	0.23	0.27	0.24
*T. cruz*i-negative_1614	0.14	0.13	0.11	0.09
*T. cruz*i-negative_1615	0.17	0.20	0.22	0.22
*T. cruz*i-negative_1616	0.17	0.17	0.14	0.14
*T. cruz*i-negative_1617	0.27	0.23	0.35	0.21
*T. cruz*i-negative_1618	0.15	0.17	0.19	0.14
*T. cruz*i-negative_1619	0.16	0.25	0.18	0.21
*T. cruz*i-negative_1620	0.07	0.14	0.12	0.08
*T. cruz*i-negative_1621	0.13	0.19	0.19	0.18
*T. cruz*i-negative_1622	0.15	0.18	0.16	0.16
*T. cruz*i-negative_1623	0.17	0.22	0.29	0.29
*T. cruz*i-negative_1624	0.34	0.19	0.17	0.20
*T. cruz*i-negative_1625	0.27	0.21	0.15	0.17
*T. cruz*i-negative_1626	0.29	0.33	0.31	0.24
*T. cruz*i-negative_1627	0.17	0.24	0.23	0.18

T. cruzi-negative_1628	0.08	0.10	0.14	0.19
T. cruzi-negative_1629	0.11	0.11	0.13	0.11
T. cruzi-negative_1630	0.14	0.17	0.20	0.18
T. cruzi-negative_1631	0.13	0.13	0.12	0.14
T. cruzi-negative_1632	0.16	0.10	0.12	0.13
T. cruzi-negative_1633	0.20	0.33	0.25	0.26
T. cruzi-negative_1634	0.16	0.25	0.25	0.22
T. cruzi-negative_1635	0.36	0.37	0.34	0.38
T. cruzi-negative_1636	0.12	0.18	0.17	0.17
T. cruzi-negative_1637	0.20	0.23	0.22	0.23
T. cruzi-negative_1638	0.18	0.23	0.19	0.29
T. cruzi-negative_1639	0.11	0.14	0.22	0.19
T. cruzi-negative_1640	0.22	0.27	0.25	0.23
T. cruzi-negative_1641	0.13	0.26	0.16	0.19
T. cruzi-negative_1642	0.17	0.21	0.25	0.19
T. cruzi-negative_1643	0.18	0.11	0.24	0.21
T. cruzi-negative_1644	0.20	0.25	0.17	0.14
T. cruzi-negative_1645	0.12	0.09	0.09	0.16
T. cruzi-negative_1646	0.21	0.18	0.15	0.16
T. cruzi-negative_1647	0.15	0.12	0.09	0.40
T. cruzi-negative_1648	0.39	0.36	0.23	0.26
T. cruzi-negative_1649	0.23	0.14	0.10	0.23
T. cruzi-negative_1650	0.27	0.18	0.13	0.16
T. cruzi-negative_1651	0.21	0.09	0.08	0.12
T. cruzi-negative_1652	0.15	0.09	0.07	0.17
T. cruzi-negative_1653	0.23	0.14	0.18	0.14
T. cruzi-negative_1654	0.21	0.09	0.05	0.18
T. cruzi-negative_1655	0.26	0.08	0.16	0.41
T. cruzi-negative_1656	0.34	0.29	0.22	0.15
T. cruzi-negative_1657	0.23	0.13	0.07	0.15
T. cruzi-negative_1658	0.24	0.17	0.13	0.15
T. cruzi-negative_1659	0.14	0.13	0.11	0.19
T. cruzi-negative_1660	0.14	0.19	0.09	0.12
T. cruzi-negative_1661	0.15	0.09	0.13	0.11
T. cruzi-negative_1662	0.17	0.05	0.05	0.12
T. cruzi-negative_1663	0.16	0.09	0.12	0.19
T. cruzi-negative_1664	0.18	0.31	0.17	0.19
T. cruzi-negative_1665	0.16	0.15	0.16	0.12
T. cruzi-negative_1666	0.17	0.11	0.08	0.18
T. cruzi-negative_1667	0.25	0.14	0.15	0.62
T. cruzi-negative_1668	0.14	0.06	0.08	0.26
T. cruzi-negative_1669	0.23	0.21	0.24	0.13

*T. cruz*i-negative_1670	0.21	0.10	0.08	0.13
*T. cruz*i-negative_1671	0.20	0.10	0.11	0.13
*T. cruz*i-negative_1672	0.12	0.08	0.11	0.09
*T. cruz*i-negative_1673	0.07	0.03	0.05	0.16
*T. cruz*i-negative_1674	0.18	0.11	0.12	0.15
*T. cruz*i-negative_1675	0.17	0.09	0.07	0.08
*T. cruz*i-negative_1676	0.08	0.05	0.04	0.15
*T. cruz*i-negative_1677	0.18	0.13	0.12	0.15
*T. cruz*i-negative_1678	0.14	0.10	0.08	0.19
*T. cruz*i-negative_1679	0.25	0.17	0.18	0.25
*T. cruz*i-negative_1680	0.19	0.19	0.18	0.24
*T. cruz*i-negative_1681	0.22	0.21	0.14	0.17
*T. cruz*i-negative_1682	0.22	0.19	0.12	0.25
*T. cruz*i-negative_1683	0.24	0.22	0.54	0.17
*T. cruz*i-negative_1684	0.21	0.17	0.17	0.08
*T. cruz*i-negative_1685	0.15	0.07	0.08	0.12
*T. cruz*i-negative_1686	0.21	0.14	0.09	0.04
*T. cruz*i-negative_1687	0.09	0.02	0.08	0.16
*T. cruz*i-negative_1688	0.20	0.14	0.10	0.30
*T. cruz*i-negative_1689	0.38	0.35	0.21	0.11
*T. cruz*i-negative_1690	0.24	0.13	0.07	0.10
*T. cruz*i-negative_1691	0.17	0.13	0.08	0.08
*T. cruz*i-negative_1692	0.14	0.07	0.06	0.12
*T. cruz*i-negative_1693	0.24	0.07	0.34	0.04
*T. cruz*i-negative_1694	0.10	0.01	0.01	0.18
*T. cruz*i-negative_1695	0.27	0.22	0.54	0.13
*T. cruz*i-negative_1696	0.15	0.12	0.11	0.16
*T. cruz*i-negative_1697	0.17	0.12	0.11	0.12
*T. cruz*i-negative_1698	0.21	0.19	0.08	0.09
*T. cruz*i-negative_1699	0.14	0.07	0.36	0.14
*T. cruz*i-negative_1700	0.24	0.17	0.14	0.20
*T. cruz*i-negative_1701	0.29	0.16	0.14	0.04
*T. cruz*i-negative_1702	0.15	0.02	0.03	0.18
*T. cruz*i-negative_1703	0.23	0.29	0.22	0.13
*T. cruz*i-negative_1704	0.14	0.16	0.10	0.11
*T. cruz*i-negative_1705	0.09	0.11	0.08	0.12
*T. cruz*i-negative_1706	0.13	0.16	0.10	0.07
*T. cruz*i-negative_1707	0.11	0.06	0.05	0.10
*T. cruz*i-negative_1708	0.14	0.07	0.08	0.10
*T. cruz*i-negative_1709	0.34	0.17	0.07	0.14
*T. cruz*i-negative_1710	0.28	0.07	0.07	0.16
*T. cruz*i-negative_1711	0.31	0.27	0.18	0.14
*T. cruz*i-negative_1712	0.10	0.12	0.13	0.16

T. cruzi-negative_1713	0.18	0.22	0.11	0.17
T. cruzi-negative_1714	0.18	0.15	0.20	0.19
T. cruzi-negative_1715	0.23	0.19	0.17	0.14
T. cruzi-negative_1716	0.21	0.01	0.02	0.13
T. cruzi-negative_1717	0.16	0.14	0.11	0.08
T. cruzi-negative_1718	0.16	0.09	0.06	0.07
T. cruzi-negative_1719	0.06	0.07	0.06	0.18
T. cruzi-negative_1720	0.11	0.15	0.09	0.18
T. cruzi-negative_1721	0.27	0.28	0.14	0.28
T. cruzi-negative_1722	0.27	0.29	0.20	0.12
T. cruzi-negative_1723	0.17	0.14	0.13	0.14
T. cruzi-negative_1724	0.26	0.17	0.15	0.20
T. cruzi-negative_1725	0.19	0.16	0.13	0.16
T. cruzi-negative_1726	0.20	0.17	0.10	0.38
T. cruzi-negative_1727	0.16	0.23	0.21	0.51
T. cruzi-negative_1728	0.22	0.24	0.19	0.61
T. cruzi-negative_1729	0.06	0.11	0.11	0.30
T. cruzi-negative_1730	0.13	0.20	0.21	0.34
T. cruzi-negative_1731	0.07	0.19	0.20	0.28
T. cruzi-negative_1732	0.04	0.15	0.11	0.40
T. cruzi-negative_1733	0.12	0.18	0.17	0.43
T. cruzi-negative_1734	0.16	0.25	0.21	0.64
T. cruzi-negative_1735	0.23	0.29	0.28	0.54
T. cruzi-negative_1736	0.20	0.14	0.13	0.56
T. cruzi-negative_1737	0.19	0.25	0.23	0.48
T. cruzi-negative_1738	0.10	0.12	0.16	0.26
T. cruzi-negative_1739	0.16	0.22	0.25	0.36
T. cruzi-negative_1740	0.10	0.17	0.20	0.46
T. cruzi-negative_1741	0.10	0.13	0.16	0.39
T. cruzi-negative_1742	0.07	0.10	0.12	0.33
T. cruzi-negative_1743	0.11	0.20	0.17	0.50
T. cruzi-negative_1744	0.07	0.13	0.12	0.32
T. cruzi-negative_1745	0.08	0.15	0.15	0.38
T. cruzi-negative_1746	0.06	0.12	0.13	0.31
T. cruzi-negative_1747	0.06	0.13	0.14	0.29
T. cruzi-negative_1748	0.08	0.14	0.16	0.42
T. cruzi-negative_1749	0.07	0.12	0.13	0.38
T. cruzi-negative_1750	0.05	0.10	0.11	0.40
T. cruzi-negative_1751	0.09	0.17	0.13	0.36
T. cruzi-negative_1752	0.10	0.17	0.16	0.35
T. cruzi-negative_1753	0.22	0.26	0.24	0.48
T. cruzi-negative_1754	0.08	0.15	0.18	0.33

T. cruzi-negative_1755	0.13	0.20	0.29	0.36
T. cruzi-negative_1756	0.11	0.14	0.17	0.50
T. cruzi-negative_1757	0.20	0.20	0.21	0.57
T. cruzi-negative_1758	0.09	0.14	0.15	0.40
T. cruzi-negative_1759	0.12	0.15	0.17	0.42
T. cruzi-negative_1760	0.14	0.16	0.13	0.33
T. cruzi-negative_1761	0.10	0.17	0.20	0.29
T. cruzi-negative_1762	0.08	0.15	0.16	0.36
T. cruzi-negative_1763	0.07	0.13	0.17	0.25
T. cruzi-negative_1764	0.09	0.14	0.13	0.36
T. cruzi-negative_1765	0.11	0.16	0.17	0.39
T. cruzi-negative_1766	0.11	0.15	0.15	0.39
T. cruzi-negative_1767	0.08	0.14	0.14	0.37
T. cruzi-negative_1768	0.23	0.29	0.32	0.39
T. cruzi-negative_1769	0.07	0.09	0.09	0.24
T. cruzi-negative_1770	0.11	0.16	0.15	0.33
T. cruzi-negative_1771	0.15	0.17	0.18	0.39
T. cruzi-negative_1772	0.04	0.16	0.13	0.34
T. cruzi-negative_1773	0.11	0.18	0.16	0.36
T. cruzi-negative_1774	0.09	0.14	0.15	0.35
T. cruzi-negative_1775	0.09	0.14	0.15	0.33
T. cruzi-negative_1776	0.03	0.08	0.10	0.28
T. cruzi-negative_1777	0.02	0.11	0.15	0.27
T. cruzi-negative_1778	0.08	0.17	0.27	0.42
T. cruzi-negative_1779	0.14	0.16	0.25	0.34
T. cruzi-negative_1780	0.06	0.14	0.13	0.37
T. cruzi-negative_1781	0.09	0.15	0.20	0.42
T. cruzi-negative_1782	0.11	0.15	0.19	0.70
T. cruzi-negative_1783	0.18	0.20	0.22	0.36
T. cruzi-negative_1784	0.04	0.09	0.11	0.30
T. cruzi-negative_1785	0.10	0.18	0.25	0.37
T. cruzi-negative_1786	0.12	0.17	0.21	0.43
T. cruzi-negative_1787	0.09	0.18	0.15	0.29
T. cruzi-negative_1788	0.13	0.19	0.26	0.41
T. cruzi-negative_1789	0.13	0.21	0.18	0.43
T. cruzi-negative_1790	0.14	0.16	0.20	0.42
T. cruzi-negative_1791	0.10	0.14	0.15	0.35
T. cruzi-negative_1792	0.15	0.16	0.15	0.38
T. cruzi-negative_1793	0.10	0.13	0.11	0.32
T. cruzi-negative_1794	0.05	0.13	0.17	0.30
T. cruzi-negative_1795	0.20	0.32	0.27	0.38
T. cruzi-negative_1796	0.11	0.18	0.17	0.44
T. cruzi-negative_1797	0.09	0.16	0.16	0.40

T. cruzi-negative_1798	0.12	0.16	0.21	0.42
T. cruzi-negative_1799	0.15	0.20	0.18	0.42
T. cruzi-negative_1800	0.03	0.12	0.12	0.30
T. cruzi-negative_1801	0.09	0.14	0.15	0.33
T. cruzi-negative_1802	0.06	0.14	0.14	0.31
T. cruzi-negative_1803	0.14	0.29	0.21	0.51
T. cruzi-negative_1804	0.08	0.12	0.12	0.33
T. cruzi-negative_1805	0.09	0.22	0.23	0.38
T. cruzi-negative_1806	0.14	0.27	0.22	0.42
T. cruzi-negative_1807	0.08	0.17	0.15	0.42
T. cruzi-negative_1808	0.13	0.21	0.15	0.37
T. cruzi-negative_1809	0.17	0.20	0.22	0.35
T. cruzi-negative_1810	0.08	0.19	0.16	0.32
T. cruzi-negative_1811	0.21	0.30	0.18	0.27
T. cruzi-negative_1812	0.28	0.36	0.20	0.34
T. cruzi-negative_1813	0.25	0.27	0.21	0.26
T. cruzi-negative_1814	0.28	0.32	0.39	0.79
T. cruzi-negative_1815	0.19	0.25	0.15	0.19
T. cruzi-negative_1816	0.24	0.24	0.18	0.22
T. cruzi-negative_1817	0.19	0.24	0.14	0.20
T. cruzi-negative_1818	0.16	0.20	0.12	0.19
T. cruzi-negative_1819	0.40	0.53	0.32	0.44
T. cruzi-negative_1820	0.18	0.18	0.15	0.20
T. cruzi-negative_1821	0.22	0.24	0.16	0.24
T. cruzi-negative_1822	0.21	0.26	0.17	0.25
T. cruzi-negative_1823	0.23	0.26	0.19	0.22
T. cruzi-negative_1824	0.26	0.28	0.26	0.27
T. cruzi-negative_1825	0.25	0.32	0.22	0.26
T. cruzi-negative_1826	0.20	0.25	0.21	0.29
T. cruzi-negative_1827	0.18	0.22	0.15	0.23
T. cruzi-negative_1828	0.20	0.29	0.21	0.26
T. cruzi-negative_1829	0.16	0.18	0.14	0.15
T. cruzi-negative_1830	0.19	0.18	0.12	0.20
T. cruzi-negative_1831	0.16	0.17	0.13	0.15
T. cruzi-negative_1832	0.26	0.23	0.17	0.27
T. cruzi-negative_1833	0.20	0.24	0.16	0.26
T. cruzi-negative_1834	0.17	0.20	0.17	0.21
T. cruzi-negative_1835	0.13	0.32	0.15	0.20
T. cruzi-negative_1836	0.33	0.33	0.38	0.36
T. cruzi-negative_1837	0.20	0.25	0.28	0.20
T. cruzi-negative_1838	0.19	0.26	0.17	0.24
T. cruzi-negative_1839	0.25	0.32	0.28	0.24

*T. cruz*i-negative_1840	0.23	0.24	0.18	0.23
*T. cruz*i-negative_1841	0.25	0.32	0.37	0.30
*T. cruz*i-negative_1842	0.25	0.32	0.27	0.33
*T. cruz*i-negative_1843	0.12	0.16	0.09	0.12
*T. cruz*i-negative_1844	0.25	0.38	0.31	0.30
*T. cruz*i-negative_1845	0.15	0.23	0.18	0.17
*T. cruz*i-negative_1846	0.17	0.36	0.19	0.21
*T. cruz*i-negative_1847	0.22	0.36	0.27	0.22
*T. cruz*i-negative_1848	0.31	0.31	0.45	0.28
*T. cruz*i-negative_1849	0.16	0.24	0.12	0.19
*T. cruz*i-negative_1850	0.24	0.24	0.19	0.23
*T. cruz*i-negative_1851	0.14	0.17	0.23	0.14
*T. cruz*i-negative_1852	0.12	0.21	0.12	0.33
*T. cruz*i-negative_1853	0.14	0.20	0.10	0.16
*T. cruz*i-negative_1854	0.27	0.34	0.26	0.33
*T. cruz*i-negative_1855	0.16	0.23	0.13	0.14
*T. cruz*i-negative_1856	0.46	0.43	0.54	0.57
*T. cruz*i-negative_1857	0.18	0.27	0.18	0.23
*T. cruz*i-negative_1858	0.29	0.24	0.38	0.32
*T. cruz*i-negative_1859	0.13	0.16	0.16	0.15
*T. cruz*i-negative_1860	0.14	0.27	0.16	0.19
*T. cruz*i-negative_1861	0.20	0.32	0.16	0.20
*T. cruz*i-negative_1862	0.17	0.35	0.15	0.16
*T. cruz*i-negative_1863	0.45	0.46	0.34	0.40
*T. cruz*i-negative_1864	0.22	0.26	0.18	0.21
*T. cruz*i-negative_1865	0.18	0.25	0.18	0.24
*T. cruz*i-negative_1866	0.19	0.19	0.13	0.19
*T. cruz*i-negative_1867	0.11	0.10	0.45	0.12
*T. cruz*i-negative_1868	0.10	0.16	0.10	0.13
*T. cruz*i-negative_1869	0.20	0.25	0.22	0.24
*T. cruz*i-negative_1870	0.20	0.28	0.19	0.24
*T. cruz*i-negative_1871	0.14	0.18	0.19	0.13
*T. cruz*i-negative_1872	0.24	0.31	0.32	0.25
*T. cruz*i-negative_1873	0.25	0.36	0.29	0.24
*T. cruz*i-negative_1874	0.20	0.17	0.38	0.21
*T. cruz*i-negative_1875	0.27	0.32	0.25	0.29
*T. cruz*i-negative_1876	0.19	0.31	0.14	0.22
*T. cruz*i-negative_1877	0.25	0.31	0.21	0.27
*T. cruz*i-negative_1878	0.21	0.33	0.31	0.32
*T. cruz*i-negative_1879	0.22	0.25	0.21	0.21
*T. cruz*i-negative_1880	0.17	0.22	0.33	0.24
*T. cruz*i-negative_1881	0.19	0.23	0.14	0.22
*T. cruz*i-negative_1882	0.16	0.22	0.38	0.18

T. cruzi-negative_1883	0.61	0.44	0.57	0.48
T. cruzi-negative_1884	0.40	0.48	0.33	0.62
T. cruzi-negative_1885	0.23	0.24	0.26	0.32
T. cruzi-negative_1886	0.18	0.22	0.18	0.30
T. cruzi-negative_1887	0.27	0.21	0.20	0.19
T. cruzi-negative_1888	0.22	0.23	0.19	0.21
T. cruzi-negative_1889	0.19	0.22	0.14	0.23
T. cruzi-negative_1890	0.16	0.26	0.15	0.17
T. cruzi-negative_1891	0.18	0.21	0.18	0.19
T. cruzi-negative_1892	0.40	0.55	0.51	0.55
T. cruzi-negative_1893	0.21	0.36	0.13	0.19
T. cruzi-negative_1894	0.22	0.21	0.15	0.21
T. cruzi-negative_1895	0.24	0.25	0.18	0.25
T. cruzi-negative_1896	0.17	0.25	0.17	0.35
T. cruzi-negative_1897	0.26	0.31	0.22	0.32
T. cruzi-negative_1898	0.16	0.28	0.17	0.24
T. cruzi-negative_1899	0.35	0.44	0.36	0.44
T. cruzi-negative_1900	0.33	0.31	0.23	0.33
T. cruzi-negative_1901	0.24	0.28	0.26	0.31
T. cruzi-negative_1902	0.11	0.12	0.11	0.13
T. cruzi-negative_1903	0.11	0.16	0.10	0.15
T. cruzi-negative_1904	0.16	0.23	0.19	0.25
T. cruzi-negative_1905	0.26	0.39	0.23	0.31
T. cruzi-negative_1906	0.32	0.42	0.31	0.38
T. cruzi-negative_1907	0.20	0.55	0.22	0.32
T. cruzi-negative_1908	0.23	0.20	0.13	0.19
T. cruzi-negative_1909	0.28	0.28	0.17	0.26
T. cruzi-negative_1910	0.13	0.24	0.15	0.24
T. cruzi-negative_1911	0.15	0.29	0.13	0.22
T. cruzi-negative_1912	0.16	0.30	0.17	0.30
T. cruzi-negative_1913	0.47	0.31	0.17	0.24
T. cruzi-negative_1914	0.18	0.29	0.14	0.23
T. cruzi-negative_1915	0.20	0.73	0.31	0.33
T. cruzi-negative_1916	0.08	0.13	0.09	0.12
T. cruzi-negative_1917	0.07	0.16	0.10	0.15
T. cruzi-negative_1918	0.15	0.21	0.17	0.23
T. cruzi-negative_1919	0.11	0.17	0.21	0.26
T. cruzi-negative_1920	0.16	0.20	0.16	0.21
T. cruzi-negative_1921	0.13	0.26	0.18	0.22
T. cruzi-negative_1922	0.12	0.23	0.11	0.23
T. cruzi-negative_1923	0.11	0.25	0.14	0.21
T. cruzi-negative_1924	0.22	0.27	0.19	0.26

*T. cruz*i-negative_1925	0.13	0.15	0.12	0.18
*T. cruz*i-negative_1926	0.17	0.27	0.18	0.27
*T. cruz*i-negative_1927	0.09	0.19	0.10	0.16
*T. cruz*i-negative_1928	0.11	0.22	0.13	0.25
*T. cruz*i-negative_1929	0.09	0.24	0.19	0.20
*T. cruz*i-negative_1930	0.17	0.28	0.18	0.27
*T. cruz*i-negative_1931	0.09	0.22	0.12	0.16
*T. cruz*i-negative_1932	0.26	0.37	0.26	0.34
*T. cruz*i-negative_1933	0.13	0.20	0.12	0.19
*T. cruz*i-negative_1934	0.18	0.28	0.16	0.22
*T. cruz*i-negative_1935	0.12	0.20	0.18	0.18
*T. cruz*i-negative_1936	0.08	0.17	0.11	0.15
*T. cruz*i-negative_1937	0.08	0.12	0.08	0.12
*T. cruz*i-negative_1938	0.11	0.20	0.12	0.19
*T. cruz*i-negative_1939	0.10	0.19	0.12	0.17
*T. cruz*i-negative_1940	0.22	0.25	0.16	0.20
*T. cruz*i-negative_1941	0.22	0.27	0.15	0.24
*T. cruz*i-negative_1942	0.07	0.22	0.08	0.12
*T. cruz*i-negative_1943	0.12	0.17	0.14	0.16
*T. cruz*i-negative_1944	0.13	0.26	0.16	0.19
*T. cruz*i-negative_1945	0.06	0.16	0.09	0.12
*T. cruz*i-negative_1946	0.11	0.20	0.20	0.17
*T. cruz*i-negative_1947	0.27	0.43	0.24	0.32
*T. cruz*i-negative_1948	0.16	0.25	0.14	0.23
*T. cruz*i-negative_1949	0.14	0.20	0.14	0.16
*T. cruz*i-negative_1950	0.12	0.20	0.14	0.17
*T. cruz*i-negative_1951	0.16	0.23	0.16	0.20
*T. cruz*i-negative_1952	0.11	0.24	0.11	0.15
*T. cruz*i-negative_1953	0.11	0.19	0.12	0.13
*T. cruz*i-negative_1954	0.08	0.33	0.13	0.17
*T. cruz*i-negative_1955	0.08	0.27	0.19	0.14
*T. cruz*i-negative_1956	0.24	0.39	0.22	0.34
*T. cruz*i-negative_1957	0.14	0.18	0.13	0.22
*T. cruz*i-negative_1958	0.06	0.11	0.06	0.10
*T. cruz*i-negative_1959	0.06	0.13	0.10	0.13
*T. cruz*i-negative_1960	0.21	0.33	0.17	0.29
*T. cruz*i-negative_1961	0.18	0.48	0.20	0.26
*T. cruz*i-negative_1962	0.15	0.26	0.18	0.22
*T. cruz*i-negative_1963	0.10	0.28	0.15	0.19
*T. cruz*i-negative_1964	0.22	0.44	0.17	0.39
*T. cruz*i-negative_1965	0.18	0.30	0.17	0.26
*T. cruz*i-negative_1966	0.21	0.34	0.18	0.31
*T. cruz*i-negative_1967	0.14	0.29	0.14	0.24

T. cruzi-negative_1968	0.10	0.25	0.14	0.19
T. cruzi-negative_1969	0.08	0.34	0.15	0.17
T. cruzi-negative_1970	0.14	0.32	0.19	0.29
T. cruzi-negative_1971	0.26	0.64	0.27	0.46
T. cruzi-negative_1972	0.18	0.42	0.19	0.32
T. cruzi-negative_1973	0.23	0.49	0.22	0.38
T. cruzi-negative_1974	0.14	0.28	0.41	0.24
T. cruzi-negative_1975	0.13	0.29	0.14	0.23
T. cruzi-negative_1976	0.11	0.28	0.19	0.20
T. cruzi-negative_1977	0.12	0.34	0.19	0.19
T. cruzi-negative_1978	0.18	0.34	0.23	0.39
T. cruzi-negative_1979	0.28	0.26	0.19	0.28
T. cruzi-negative_1980	0.20	0.18	0.14	0.20
T. cruzi-negative_1981	0.17	0.14	0.13	0.14
T. cruzi-negative_1982	0.17	0.15	0.14	0.15
T. cruzi-negative_1983	0.35	0.36	0.47	0.31
T. cruzi-negative_1984	0.17	0.18	0.16	0.18
T. cruzi-negative_1985	0.42	0.20	0.18	0.29
T. cruzi-negative_1986	0.20	0.13	0.10	0.17
T. cruzi-negative_1987	0.25	0.25	0.17	0.23
T. cruzi-negative_1988	0.34	0.31	0.30	0.29
T. cruzi-negative_1989	0.21	0.35	0.22	0.28
T. cruzi-negative_1990	0.17	0.21	0.20	0.17
T. cruzi-negative_1991	0.26	0.40	0.51	0.29
T. cruzi-negative_1992	0.22	0.27	0.26	0.33
T. cruzi-negative_1993	0.56	0.25	0.26	0.35
T. cruzi-negative_1994	0.18	0.12	0.08	0.14
T. cruzi-negative_1995	0.17	0.13	0.10	0.14
T. cruzi-negative_1996	0.16	0.17	0.13	0.16
T. cruzi-negative_1997	0.14	0.17	0.11	0.15
T. cruzi-negative_1998	0.19	0.19	0.26	0.19
T. cruzi-negative_1999	0.16	0.21	0.44	0.19
T. cruzi-negative_2000	0.47	0.20	0.17	0.28
T. cruzi-negative_2001	0.23	0.14	0.11	0.18
T. cruzi-negative_2002	0.23	0.17	0.11	0.17
T. cruzi-negative_2003	0.24	0.19	0.17	0.25
T. cruzi-negative_2004	0.11	0.15	0.19	0.13
T. cruzi-negative_2005	0.17	0.25	0.15	0.22
T. cruzi-negative_2006	0.17	0.17	0.19	0.18
T. cruzi-negative_2007	0.23	0.31	0.36	0.25
T. cruzi-negative_2008	0.31	0.18	0.13	0.20
T. cruzi-negative_2009	0.21	0.38	0.12	0.19

*T. cruz*i-negative_2010	0.24	0.14	0.09	0.17
*T. cruz*i-negative_2011	0.16	0.19	0.13	0.18
*T. cruz*i-negative_2012	0.17	0.23	0.16	0.24
*T. cruz*i-negative_2013	0.13	0.17	0.12	0.18
*T. cruz*i-negative_2014	0.13	0.17	0.13	0.15
*T. cruz*i-negative_2015	0.19	0.35	0.27	0.21
*T. cruz*i-negative_2016	0.46	0.21	0.16	0.25
*T. cruz*i-negative_2017	0.18	0.14	0.10	0.18
*T. cruz*i-negative_2018	0.22	0.28	0.14	0.23
*T. cruz*i-negative_2019	0.20	0.22	0.14	0.19
*T. cruz*i-negative_2020	0.12	0.18	0.11	0.17
*T. cruz*i-negative_2021	0.14	0.14	0.09	0.16
*T. cruz*i-negative_2022	0.14	0.19	0.14	0.17
*T. cruz*i-negative_2023	0.18	0.21	0.28	0.16
*T. cruz*i-negative_2024	0.28	0.45	0.22	0.45
*T. cruz*i-negative_2025	0.27	0.16	0.08	0.21
*T. cruz*i-negative_2026	0.26	0.19	0.13	0.30
*T. cruz*i-negative_2027	0.16	0.13	0.10	0.16
*T. cruz*i-negative_2028	0.14	0.17	0.12	0.19
*T. cruz*i-negative_2029	0.18	0.28	0.24	0.30
*T. cruz*i-negative_2030	0.16	0.24	0.23	0.17
*T. cruz*i-negative_2031	0.11	0.20	0.28	0.12
*T. cruz*i-negative_2032	0.43	0.29	0.15	0.24
*T. cruz*i-negative_2033	0.17	0.32	0.08	0.15
*T. cruz*i-negative_2034	0.19	0.18	0.14	0.20
*T. cruz*i-negative_2035	0.13	0.12	0.08	0.11
*T. cruz*i-negative_2036	0.12	0.15	0.10	0.14
*T. cruz*i-negative_2037	0.18	0.23	0.17	0.21
*T. cruz*i-negative_2038	0.16	0.22	0.22	0.29
*T. cruz*i-negative_2039	0.16	0.24	0.33	0.22
*T. cruz*i-negative_2040	0.33	0.19	0.15	0.20
*T. cruz*i-negative_2041	0.23	0.16	0.11	0.19
*T. cruz*i-negative_2042	0.16	0.16	0.09	0.13
*T. cruz*i-negative_2043	0.14	0.14	0.09	0.13
*T. cruz*i-negative_2044	0.13	0.18	0.12	0.17
*T. cruz*i-negative_2045	0.15	0.30	0.16	0.21
*T. cruz*i-negative_2046	0.16	0.23	0.20	0.19
*T. cruz*i-negative_2047	0.21	0.49	0.44	0.29
*T. cruz*i-negative_2048	0.25	0.26	0.10	0.23
*T. cruz*i-negative_2049	0.21	0.20	0.14	0.26
*T. cruz*i-negative_2050	0.16	0.17	0.11	0.19
*T. cruz*i-negative_2051	0.24	0.29	0.24	0.39
*T. cruz*i-negative_2052	0.18	0.33	0.19	0.21

T. cruzi-negative_2053	0.12	0.23	0.08	0.12
T. cruzi-negative_2054	0.24	0.31	0.24	0.37
T. cruzi-negative_2055	0.19	0.36	0.37	0.28
T. cruzi-negative_2056	0.42	0.26	0.17	0.29
T. cruzi-negative_2057	0.22	0.33	0.12	0.25
T. cruzi-negative_2058	0.31	0.26	0.13	0.26
T. cruzi-negative_2059	0.24	0.25	0.20	0.23
T. cruzi-negative_2060	0.31	0.28	0.21	0.27
T. cruzi-negative_2061	0.33	0.27	0.22	0.31
T. cruzi-negative_2062	0.17	0.16	0.18	0.15
T. cruzi-negative_2063	0.69	0.74	0.67	0.73
T. cruzi-negative_2064	0.13	0.17	0.09	0.10
T. cruzi-negative_2065	0.19	0.24	0.14	0.16
T. cruzi-negative_2066	0.33	0.39	0.24	0.25
T. cruzi-negative_2067	0.27	0.26	0.21	0.19
T. cruzi-negative_2068	0.20	0.25	0.13	0.15
T. cruzi-negative_2069	0.15	0.19	0.12	0.09
T. cruzi-negative_2070	0.11	0.18	0.18	0.14
T. cruzi-negative_2071	0.26	0.35	0.31	0.26
T. cruzi-negative_2072	0.20	0.26	0.23	0.18
T. cruzi-negative_2073	0.31	0.41	0.45	0.40
T. cruzi-negative_2074	0.46	0.51	0.56	0.57
T. cruzi-negative_2075	0.15	0.21	0.16	0.14
T. cruzi-negative_2076	0.13	0.21	0.13	0.14
T. cruzi-negative_2077	0.24	0.23	0.25	0.16
T. cruzi-negative_2078	0.13	0.27	0.16	0.23
T. cruzi-negative_2079	0.31	0.42	0.45	0.48
T. cruzi-negative_2080	0.16	0.35	0.17	0.15
T. cruzi-negative_2081	0.22	0.25	0.21	0.20
T. cruzi-negative_2082	0.21	0.25	0.18	0.19
T. cruzi-negative_2083	0.21	0.23	0.14	0.16
T. cruzi-negative_2084	0.27	0.35	0.43	0.33
T. cruzi-negative_2085	0.20	0.21	0.15	0.17
T. cruzi-negative_2086	0.14	0.25	0.16	0.21
T. cruzi-negative_2087	0.17	0.33	0.17	0.17
T. cruzi-negative_2088	0.15	0.18	0.15	0.17
T. cruzi-negative_2089	0.26	0.21	0.24	0.22
T. cruzi-negative_2090	0.14	0.17	0.11	0.10
T. cruzi-negative_2091	0.33	0.49	0.24	0.34
T. cruzi-negative_2092	0.14	0.21	0.16	0.57
T. cruzi-negative_2093	0.14	0.16	0.12	0.12
T. cruzi-negative_2094	0.10	0.22	0.26	0.26

T. cruzi-negative_2095	0.13	0.22	0.14	0.16
T. cruzi-negative_2096	0.10	0.12	0.10	0.11
T. cruzi-negative_2097	0.14	0.16	0.11	0.12
T. cruzi-negative_2098	0.16	0.18	0.14	0.15
T. cruzi-negative_2099	0.25	0.30	0.15	0.23
T. cruzi-negative_2100	0.33	0.31	0.24	0.35
T. cruzi-negative_2101	0.23	0.20	0.13	0.15
T. cruzi-negative_2102	0.13	0.25	0.16	0.20
T. cruzi-negative_2103	0.13	0.21	0.09	0.15
T. cruzi-negative_2104	0.14	0.21	0.12	0.14
T. cruzi-negative_2105	0.17	0.19	0.15	0.17
T. cruzi-negative_2106	0.14	0.17	0.16	0.11
T. cruzi-negative_2107	0.17	0.23	0.12	0.16
T. cruzi-negative_2108	0.27	0.26	0.13	0.17
T. cruzi-negative_2109	0.15	0.29	0.16	0.21
T. cruzi-negative_2110	0.20	0.23	0.14	0.16
T. cruzi-negative_2111	0.14	0.14	0.11	0.12
T. cruzi-negative_2112	0.17	0.26	0.19	0.19
T. cruzi-negative_2113	0.15	0.28	0.14	0.15
T. cruzi-negative_2114	0.19	0.39	0.23	0.22
T. cruzi-negative_2115	0.26	0.32	0.17	0.24
T. cruzi-negative_2116	0.11	0.16	0.10	0.10
T. cruzi-negative_2117	0.14	0.17	0.12	0.14
T. cruzi-negative_2118	0.17	0.25	0.24	0.14
T. cruzi-negative_2119	0.14	0.20	0.13	0.15
T. cruzi-negative_2120	0.12	0.12	0.11	0.16
T. cruzi-negative_2121	0.24	0.33	0.19	0.24
T. cruzi-negative_2122	0.06	0.14	0.09	0.06
T. cruzi-negative_2123	0.19	0.25	0.13	0.15
T. cruzi-negative_2124	0.13	0.18	0.16	0.14
T. cruzi-negative_2125	0.17	0.15	0.14	0.16
T. cruzi-negative_2126	0.13	0.19	0.14	0.17
T. cruzi-negative_2127	0.18	0.21	0.16	0.20
T. cruzi-negative_2128	0.10	0.13	0.09	0.11
T. cruzi-negative_2129	0.24	0.27	0.25	0.27
T. cruzi-negative_2130	0.12	0.12	0.09	0.10
T. cruzi-negative_2131	0.15	0.21	0.12	0.16
T. cruzi-negative_2132	0.13	0.18	0.22	0.15
T. cruzi-negative_2133	0.11	0.24	0.22	0.21
T. cruzi-negative_2134	0.19	0.11	0.11	0.11
T. cruzi-negative_2135	0.19	0.24	0.20	0.23
T. cruzi-negative_2136	0.18	0.26	0.24	0.19
T. cruzi-negative_2137	0.16	0.28	0.17	0.18

T. cruzi-negative_2138	0.15	0.45	0.27	0.17
T. cruzi-negative_2139	0.11	0.18	0.11	0.11
T. cruzi-negative_2140	0.20	0.29	0.25	0.23
T. cruzi-negative_2141	0.23	0.27	0.19	0.20
T. cruzi-negative_2142	0.18	0.26	0.22	0.23
T. cruzi-negative_2143	0.15	0.20	0.27	0.16
T. cruzi-negative_2144	0.19	0.23	0.26	0.22
T. cruzi-negative_2145	0.19	0.29	0.38	0.19
T. cruzi-negative_2146	0.19	0.21	0.17	0.18
T. cruzi-negative_2147	0.29	0.29	0.19	0.27
T. cruzi-negative_2148	0.44	0.65	0.50	0.41
T. cruzi-negative_2149	0.21	0.24	0.20	0.17
T. cruzi-negative_2150	0.17	0.21	0.15	0.19
T. cruzi-negative_2151	0.34	0.34	0.36	0.34
T. cruzi-negative_2152	0.25	0.27	0.19	0.27
T. cruzi-negative_2153	0.23	0.28	0.22	0.21
T. cruzi-negative_2154	0.16	0.18	0.12	0.15
T. cruzi-negative_2155	0.23	0.21	0.14	0.18
T. cruzi-negative_2156	0.19	0.18	0.22	0.17
T. cruzi-negative_2157	0.31	0.24	0.12	0.16
T. cruzi-negative_2158	0.20	0.31	0.17	0.14
T. cruzi-negative_2159	0.25	0.27	0.36	0.23
T. cruzi-negative_2160	0.28	0.32	0.21	0.29
T. cruzi-negative_2161	0.21	0.31	0.17	0.17
T. cruzi-negative_2162	0.16	0.21	0.15	0.16
T. cruzi-negative_2163	0.17	0.23	0.11	0.22
T. cruzi-negative_2164	0.18	0.18	0.23	0.11
T. cruzi-negative_2165	0.33	0.36	0.22	0.23
T. cruzi-negative_2166	0.37	0.39	0.26	0.26
T. cruzi-negative_2167	0.19	0.22	0.39	0.19
T. cruzi-negative_2168	0.27	0.32	0.45	0.26
T. cruzi-negative_2169	0.25	0.34	0.20	0.23
T. cruzi-negative_2170	0.23	0.24	0.19	0.21
T. cruzi-negative_2171	0.83	0.59	0.80	0.76
T. cruzi-negative_2172	0.24	0.16	0.30	0.19
T. cruzi-negative_2173	0.19	0.19	0.14	0.15
T. cruzi-negative_2174	0.20	0.19	0.15	0.15
T. cruzi-negative_2175	0.20	0.34	0.17	0.18
T. cruzi-negative_2176	0.18	0.20	0.21	0.21
T. cruzi-negative_2177	0.18	0.22	0.38	0.28
T. cruzi-negative_2178	0.11	0.17	0.09	0.11
T. cruzi-negative_2179	0.16	0.30	0.17	0.12

T. cruzi-negative_2180	0.14	0.21	0.12	0.12
T. cruzi-negative_2181	0.35	0.38	0.23	0.45
T. cruzi-negative_2182	0.26	0.42	0.27	0.32
T. cruzi-negative_2183	0.14	0.29	0.10	0.16
T. cruzi-negative_2184	0.26	0.17	0.19	0.23
T. cruzi-negative_2185	0.24	0.30	0.19	0.20
T. cruzi-negative_2186	0.15	0.17	0.14	0.17
T. cruzi-negative_2187	0.21	0.24	0.16	0.14
T. cruzi-negative_2188	0.13	0.10	0.12	0.05
T. cruzi-negative_2189	0.22	0.20	0.17	0.15
T. cruzi-negative_2190	0.64	0.64	0.49	0.54
T. cruzi-negative_2191	0.17	0.20	0.09	0.22
T. cruzi-negative_2192	0.28	0.21	0.11	0.15
T. cruzi-negative_2193	0.19	0.23	0.11	0.14
T. cruzi-negative_2194	0.38	0.16	0.12	0.20
T. cruzi-negative_2195	0.20	0.14	0.28	0.13
T. cruzi-negative_2196	0.23	0.31	0.22	0.24
T. cruzi-negative_2197	0.21	0.28	0.18	0.26
T. cruzi-negative_2198	0.10	0.20	0.08	0.10
T. cruzi-negative_2199	0.15	0.08	0.11	0.14
T. cruzi-negative_2200	0.21	0.20	0.15	0.15
T. cruzi-negative_2201	0.19	0.17	0.14	0.14
T. cruzi-negative_2202	0.11	0.15	0.07	0.09
T. cruzi-negative_2203	0.32	0.27	0.33	0.18
T. cruzi-negative_2204	0.10	0.10	0.08	0.06
T. cruzi-negative_2205	0.16	0.19	0.15	0.13
T. cruzi-negative_2206	0.09	0.18	0.11	0.11
T. cruzi-negative_2207	0.14	0.12	0.10	0.12
T. cruzi-negative_2208	0.17	0.21	0.13	0.13
T. cruzi-negative_2209	0.16	0.19	0.11	0.13
T. cruzi-negative_2210	0.30	0.37	0.26	0.23
T. cruzi-negative_2211	0.10	0.11	0.09	0.11
T. cruzi-negative_2212	0.15	0.18	0.12	0.09
T. cruzi-negative_2213	0.12	0.18	0.17	0.14
T. cruzi-negative_2214	0.20	0.36	0.26	0.22
T. cruzi-negative_2215	0.22	0.30	0.19	0.29
T. cruzi-negative_2216	0.24	0.24	0.24	0.21
T. cruzi-negative_2217	0.16	0.19	0.11	0.13
T. cruzi-negative_2218	0.26	0.34	0.27	0.20
T. cruzi-negative_2219	0.21	0.20	0.24	0.15
T. cruzi-negative_2220	0.24	0.37	0.20	0.22
T. cruzi-negative_2221	0.25	0.18	0.14	0.19
T. cruzi-negative_2222	0.15	0.43	0.15	0.14

*T. cruz*i-negative_2223	0.16	0.29	0.20	0.17
*T. cruz*i-negative_2224	0.23	0.31	0.20	0.26
*T. cruz*i-negative_2225	0.10	0.12	0.07	0.08
*T. cruz*i-negative_2226	0.20	0.23	0.14	0.17
*T. cruz*i-negative_2227	0.14	0.18	0.10	0.11
*T. cruz*i-negative_2228	0.19	0.22	0.31	0.20
*T. cruz*i-negative_2229	0.26	0.20	0.28	0.21
*T. cruz*i-negative_2230	0.26	0.33	0.32	0.25
*T. cruz*i-negative_2231	0.24	0.23	0.27	0.22
*T. cruz*i-negative_2232	0.36	0.34	0.42	0.36
*T. cruz*i-negative_2233	0.13	0.16	0.27	0.24
*T. cruz*i-negative_2234	0.17	0.27	0.33	0.34
*T. cruz*i-negative_2235	0.16	0.18	0.27	0.22
*T. cruz*i-negative_2236	0.28	0.29	0.36	0.27
*T. cruz*i-negative_2237	0.19	0.23	0.25	0.23
*T. cruz*i-negative_2238	0.23	0.14	0.25	0.19
*T. cruz*i-negative_2239	0.19	0.19	0.23	0.17
*T. cruz*i-negative_2240	0.19	0.23	0.25	0.21
*T. cruz*i-negative_2241	0.20	0.29	0.30	0.24
*T. cruz*i-negative_2242	0.27	0.23	0.43	0.29
*T. cruz*i-negative_2243	0.15	0.26	0.28	0.25
*T. cruz*i-negative_2244	0.20	0.19	0.29	0.29
*T. cruz*i-negative_2245	0.18	0.15	0.25	0.26
*T. cruz*i-negative_2246	0.12	0.14	0.21	0.16
*T. cruz*i-negative_2247	0.12	0.14	0.20	0.17
*T. cruz*i-negative_2248	0.16	0.22	0.21	0.25
*T. cruz*i-negative_2249	0.23	0.20	0.44	0.29
*T. cruz*i-negative_2250	0.18	0.19	0.26	0.20
*T. cruz*i-negative_2251	0.17	0.19	0.25	0.26
*T. cruz*i-negative_2252	0.15	0.13	0.21	0.20
*T. cruz*i-negative_2253	0.18	0.13	0.20	0.16
*T. cruz*i-negative_2254	0.15	0.14	0.26	0.18
*T. cruz*i-negative_2255	0.26	0.35	0.26	0.22
*T. cruz*i-negative_2256	0.15	0.15	0.23	0.21
*T. cruz*i-negative_2257	0.25	0.19	0.38	0.22
*T. cruz*i-negative_2258	0.17	0.23	0.28	0.23
*T. cruz*i-negative_2259	0.21	0.19	0.29	0.28
*T. cruz*i-negative_2260	0.22	0.19	0.27	0.24
*T. cruz*i-negative_2261	0.16	0.16	0.24	0.17
*T. cruz*i-negative_2262	0.16	0.23	0.22	0.20
*T. cruz*i-negative_2263	0.26	0.19	0.22	0.20
*T. cruz*i-negative_2264	0.23	0.26	0.34	0.39

T. cruzi-negative_2265	0.22	0.23	0.36	0.24
T. cruzi-negative_2266	0.15	0.18	0.22	0.20
T. cruzi-negative_2267	0.15	0.22	0.18	0.21
T. cruzi-negative_2268	0.45	0.19	0.22	0.25
T. cruzi-negative_2269	0.15	0.13	0.22	0.18
T. cruzi-negative_2270	0.26	0.28	0.37	0.35
T. cruzi-negative_2271	0.11	0.14	0.19	0.22
T. cruzi-negative_2272	0.21	0.23	0.31	0.33
T. cruzi-negative_2273	0.16	0.14	0.25	0.28
T. cruzi-negative_2274	0.19	0.22	0.26	0.30
T. cruzi-negative_2275	0.19	0.22	0.25	0.25
T. cruzi-negative_2276	0.21	0.14	0.21	0.25
T. cruzi-negative_2277	0.17	0.21	0.24	0.23
T. cruzi-negative_2278	0.23	0.23	0.31	0.31
T. cruzi-negative_2279	0.28	0.19	0.24	0.25
T. cruzi-negative_2280	0.15	0.18	0.17	0.25
T. cruzi-negative_2281	0.24	0.17	0.27	0.23
T. cruzi-negative_2282	0.10	0.14	0.20	0.15
T. cruzi-negative_2283	0.12	0.17	0.19	0.18
T. cruzi-negative_2284	0.21	0.43	0.26	0.24
T. cruzi-negative_2285	0.14	0.13	0.21	0.17
T. cruzi-negative_2286	0.10	0.09	0.18	0.16
T. cruzi-negative_2287	0.11	0.09	0.13	0.18
T. cruzi-negative_2288	0.21	0.26	0.26	0.37
T. cruzi-negative_2289	0.15	0.27	0.22	0.19
T. cruzi-negative_2290	0.09	0.11	0.15	0.14
T. cruzi-negative_2291	0.19	0.21	0.25	0.24
T. cruzi-negative_2292	0.17	0.23	0.31	0.23
T. cruzi-negative_2293	0.16	0.17	0.23	0.21
T. cruzi-negative_2294	0.14	0.10	0.20	0.18
T. cruzi-negative_2295	0.13	0.12	0.13	0.16
T. cruzi-negative_2296	0.09	0.14	0.13	0.21
T. cruzi-negative_2297	0.12	0.11	0.19	0.16
T. cruzi-negative_2298	0.09	0.11	0.17	0.20
T. cruzi-negative_2299	0.09	0.09	0.16	0.17
T. cruzi-negative_2300	0.07	0.11	0.16	0.19
T. cruzi-negative_2301	0.15	0.13	0.25	0.20
T. cruzi-negative_2302	0.09	0.06	0.19	0.13
T. cruzi-negative_2303	0.21	0.23	0.16	0.22
T. cruzi-negative_2304	0.22	0.30	0.34	0.39
T. cruzi-negative_2305	0.10	0.15	0.22	0.17
T. cruzi-negative_2306	0.14	0.15	0.21	0.22
T. cruzi-negative_2307	0.38	0.44	0.64	0.73

T. cruzi-negative_2308	0.12	0.11	0.17	0.14
T. cruzi-negative_2309	0.08	0.10	0.14	0.11
T. cruzi-negative_2310	0.20	0.14	0.24	0.29
T. cruzi-negative_2311	0.11	0.21	0.16	0.20
T. cruzi-negative_2312	0.23	0.25	0.17	0.16
T. cruzi-negative_2313	0.24	0.28	0.26	0.24
T. cruzi-negative_2314	0.39	0.34	0.32	0.20
T. cruzi-negative_2315	0.21	0.26	0.23	0.15
T. cruzi-negative_2316	0.25	0.29	0.27	0.20
T. cruzi-negative_2317	0.20	0.23	0.21	0.21
T. cruzi-negative_2318	0.28	0.35	0.29	0.31
T. cruzi-negative_2319	0.22	0.26	0.22	0.18
T. cruzi-negative_2320	0.29	0.25	0.26	0.25
T. cruzi-negative_2321	0.45	0.25	0.28	0.24
T. cruzi-negative_2322	0.23	0.22	0.23	0.20
T. cruzi-negative_2323	0.37	0.39	0.27	0.31
T. cruzi-negative_2324	0.20	0.21	0.18	0.17
T. cruzi-negative_2325	0.17	0.30	0.13	0.15
T. cruzi-negative_2326	0.22	0.26	0.33	0.22
T. cruzi-negative_2327	0.28	0.31	0.24	0.29
T. cruzi-negative_2328	0.28	0.31	0.30	0.29
T. cruzi-negative_2329	0.25	0.25	0.36	0.18
T. cruzi-negative_2330	0.30	0.21	0.25	0.15
T. cruzi-negative_2331	0.20	0.19	0.18	0.18
T. cruzi-negative_2332	0.34	0.37	0.29	0.43
T. cruzi-negative_2333	0.33	0.39	0.29	0.30
T. cruzi-negative_2334	0.22	0.28	0.22	0.15
T. cruzi-negative_2335	0.22	0.23	0.28	0.14
T. cruzi-negative_2336	0.27	0.22	0.26	0.26
T. cruzi-negative_2337	0.17	0.20	0.30	0.10
T. cruzi-negative_2338	0.16	0.18	0.20	0.13
T. cruzi-negative_2339	0.17	0.24	0.27	0.13
T. cruzi-negative_2340	0.24	0.27	0.28	0.34
T. cruzi-negative_2341	0.34	0.29	0.25	0.29
T. cruzi-negative_2342	0.19	0.24	0.18	0.15
T. cruzi-negative_2343	0.15	0.15	0.16	0.14
T. cruzi-negative_2344	0.10	0.17	0.11	0.08
T. cruzi-negative_2345	0.21	0.19	0.24	0.36
T. cruzi-negative_2346	0.41	0.34	0.24	0.38
T. cruzi-negative_2347	0.22	0.17	0.22	0.17
T. cruzi-negative_2348	0.19	0.19	0.19	0.14
T. cruzi-negative_2349	0.22	0.28	0.27	0.16

*T. cruz*i-negative_2350	0.16	0.17	0.14	0.13
*T. cruz*i-negative_2351	0.18	0.22	0.17	0.15
*T. cruz*i-negative_2352	0.33	0.28	0.31	0.36
*T. cruz*i-negative_2353	0.20	0.28	0.28	0.16
*T. cruz*i-negative_2354	0.26	0.26	0.21	0.30
*T. cruz*i-negative_2355	0.22	0.23	0.19	0.13
*T. cruz*i-negative_2356	0.38	0.38	0.32	0.23
*T. cruz*i-negative_2357	0.19	0.20	0.18	0.17
*T. cruz*i-negative_2358	0.16	0.19	0.16	0.14
*T. cruz*i-negative_2359	0.24	0.33	0.28	0.26
*T. cruz*i-negative_2360	0.24	0.28	0.25	0.24
*T. cruz*i-negative_2361	0.29	0.33	0.28	0.31
*T. cruz*i-negative_2362	0.25	0.34	0.27	0.29
*T. cruz*i-negative_2363	0.17	0.22	0.18	0.12
*T. cruz*i-negative_2364	0.25	0.33	0.20	0.09
*T. cruz*i-negative_2365	0.39	0.39	0.39	0.48
*T. cruz*i-negative_2366	0.21	0.33	0.32	0.14
*T. cruz*i-negative_2367	0.31	0.30	0.26	0.32
*T. cruz*i-negative_2368	0.19	0.22	0.18	0.19
*T. cruz*i-negative_2369	0.20	0.24	0.27	0.23
*T. cruz*i-negative_2370	0.21	0.30	0.21	0.28
*T. cruz*i-negative_2371	0.27	0.29	0.24	0.21
*T. cruz*i-negative_2372	0.35	0.49	0.30	0.30
*T. cruz*i-negative_2373	0.13	0.16	0.14	0.10
*T. cruz*i-negative_2374	0.16	0.28	0.33	0.27
*T. cruz*i-negative_2375	0.14	0.22	0.19	0.19
*T. cruz*i-negative_2376	0.16	0.19	0.19	0.18
*T. cruz*i-negative_2377	0.17	0.18	0.34	0.15
*T. cruz*i-negative_2378	0.28	0.32	0.27	0.24
*T. cruz*i-negative_2379	0.16	0.18	0.16	0.10
*T. cruz*i-negative_2380	0.19	0.22	0.20	0.19
*T. cruz*i-negative_2381	0.17	0.20	0.17	0.18
*T. cruz*i-negative_2382	0.16	0.19	0.14	0.19
*T. cruz*i-negative_2383	0.22	0.29	0.20	0.26
*T. cruz*i-negative_2384	0.15	0.24	0.20	0.17
*T. cruz*i-negative_2385	0.19	0.17	0.19	0.14
*T. cruz*i-negative_2386	0.18	0.23	0.24	0.29
*T. cruz*i-negative_2387	0.22	0.28	0.22	0.20
*T. cruz*i-negative_2388	0.30	0.30	0.27	0.23
*T. cruz*i-negative_2389	0.23	0.24	0.22	0.17
*T. cruz*i-negative_2390	0.14	0.19	0.14	0.11
*T. cruz*i-negative_2391	0.22	0.25	0.22	0.21
*T. cruz*i-negative_2392	0.45	0.76	0.39	0.56

T. cruzi-negative_2393	0.18	0.32	0.24	0.18
T. cruzi-negative_2394	0.22	0.31	0.26	0.19
T. cruzi-negative_2395	0.45	0.43	0.43	0.29
T. cruzi-negative_2396	0.27	0.26	0.27	0.30
T. cruzi-negative_2397	0.18	0.15	0.18	0.21
T. cruzi-negative_2398	0.34	0.38	0.25	0.32
T. cruzi-negative_2399	0.18	0.26	0.23	0.23
T. cruzi-negative_2400	0.22	0.26	0.24	0.24
T. cruzi-negative_2401	0.19	0.17	0.19	0.17
T. cruzi-negative_2402	0.21	0.24	0.16	0.19
T. cruzi-negative_2403	0.20	0.26	0.19	0.21
T. cruzi-negative_2404	0.17	0.22	0.16	0.17
T. cruzi-negative_2405	0.34	0.39	0.27	0.42
T. cruzi-negative_2406	0.17	0.21	0.15	0.14
T. cruzi-negative_2407	0.30	0.29	0.30	0.27
T. cruzi-negative_2408	0.30	0.40	0.27	0.31
T. cruzi-negative_2409	0.15	0.18	0.19	0.15
T. cruzi-negative_2410	0.27	0.36	0.26	0.32
T. cruzi-negative_2411	0.27	0.30	0.24	0.28
T. cruzi-negative_2412	0.20	0.24	0.21	0.18
T. cruzi-negative_2413	0.28	0.25	0.39	0.32
T. cruzi-negative_2414	0.15	0.18	0.15	0.13
T. cruzi-negative_2415	0.31	0.36	0.30	0.29
T. cruzi-negative_2416	0.33	0.49	0.28	0.36
T. cruzi-negative_2417	0.44	0.58	0.32	0.44
T. cruzi-negative_2418	0.21	0.28	0.15	0.17
T. cruzi-negative_2419	0.23	0.30	0.26	0.25
T. cruzi-negative_2420	0.21	0.28	0.17	0.19
T. cruzi-negative_2421	0.35	0.32	0.34	0.50
T. cruzi-negative_2422	0.27	0.34	0.26	0.28
T. cruzi-negative_2423	0.20	0.19	0.22	0.16
T. cruzi-negative_2424	0.14	0.12	0.11	0.08
T. cruzi-negative_2425	0.32	0.32	0.27	0.26
T. cruzi-negative_2426	0.29	0.39	0.31	0.26
T. cruzi-negative_2427	0.19	0.26	0.20	0.18
T. cruzi-negative_2428	0.34	0.40	0.24	0.29
T. cruzi-negative_2429	0.22	0.19	0.23	0.30
T. cruzi-negative_2430	0.38	0.45	0.30	0.32
T. cruzi-negative_2431	0.12	0.14	0.28	0.11
T. cruzi-negative_2432	0.22	0.29	0.19	0.22
T. cruzi-negative_2433	0.23	0.28	0.22	0.24
T. cruzi-negative_2434	0.31	0.40	0.31	0.30

T. cruzi-negative_2435	0.28	0.33	0.26	0.29
T. cruzi-negative_2436	0.20	0.24	0.21	0.17
T. cruzi-negative_2437	0.21	0.17	0.21	0.26
T. cruzi-negative_2438	0.23	0.31	0.20	0.20
T. cruzi-negative_2439	0.26	0.37	0.28	0.23
T. cruzi-negative_2440	0.30	0.44	0.27	0.35
T. cruzi-negative_2441	0.15	0.22	0.25	0.21
T. cruzi-negative_2442	0.28	0.34	0.38	0.28
T. cruzi-negative_2443	0.20	0.26	0.18	0.23
T. cruzi-negative_2444	0.17	0.22	0.18	0.19
T. cruzi-negative_2445	0.21	0.18	0.19	0.22
T. cruzi-negative_2446	0.40	0.10	0.30	0.06
T. cruzi-negative_2447	0.28	0.47	0.29	0.36
T. cruzi-negative_2448	0.25	0.31	0.24	0.23
T. cruzi-negative_2449	0.21	0.34	0.24	0.22
T. cruzi-negative_2450	0.16	0.20	0.16	0.14
T. cruzi-negative_2451	0.19	0.19	0.18	0.15
T. cruzi-negative_2452	0.23	0.27	0.19	0.20
T. cruzi-negative_2453	0.23	0.28	0.23	0.27
T. cruzi-negative_2454	0.22	0.36	0.20	0.23
T. cruzi-negative_2455	0.12	0.21	0.13	0.12
T. cruzi-negative_2456	0.35	0.41	0.33	0.33
T. cruzi-negative_2457	0.12	0.20	0.13	0.12
T. cruzi-negative_2458	0.22	0.27	0.20	0.17
T. cruzi-negative_2459	0.19	0.25	0.27	0.18
T. cruzi-negative_2460	0.18	0.24	0.17	0.20
T. cruzi-negative_2461	0.24	0.28	0.22	0.32
T. cruzi-negative_2462	0.25	0.35	0.24	0.26
T. cruzi-negative_2463	0.15	0.18	0.24	0.19
T. cruzi-negative_2464	0.30	0.39	0.26	0.26
T. cruzi-negative_2465	0.14	0.25	0.22	0.15
T. cruzi-negative_2466	0.19	0.26	0.19	0.18
T. cruzi-negative_2467	0.23	0.28	0.21	0.25
T. cruzi-negative_2468	0.27	0.29	0.36	0.22
T. cruzi-negative_2469	0.12	0.15	0.10	0.10
T. cruzi-negative_2470	0.19	0.26	0.17	0.19
T. cruzi-negative_2471	0.21	0.28	0.24	0.21
T. cruzi-negative_2472	0.31	0.46	0.28	0.33
T. cruzi-negative_2473	0.09	0.26	0.12	0.13
T. cruzi-negative_2474	0.43	0.41	0.22	0.20
T. cruzi-negative_2475	0.27	0.61	0.23	0.46
T. cruzi-negative_2476	0.59	0.52	0.21	0.43
T. cruzi-negative_2477	0.26	0.40	0.33	0.31

T. cruzi-negative_2478	0.13	0.28	0.17	0.22
T. cruzi-negative_2479	0.10	0.18	0.15	0.14
T. cruzi-negative_2480	0.21	0.34	0.23	0.27
T. cruzi-negative_2481	0.25	0.41	0.31	0.20
T. cruzi-negative_2482	0.40	0.58	0.31	0.32
T. cruzi-negative_2483	0.31	0.42	0.29	0.26
T. cruzi-negative_2484	0.18	0.30	0.21	0.27
T. cruzi-negative_2485	0.26	0.34	0.30	0.31
T. cruzi-negative_2486	0.27	0.44	0.28	0.29
T. cruzi-negative_2487	0.18	0.33	0.21	0.24
T. cruzi-negative_2488	0.22	0.37	0.27	0.29
T. cruzi-negative_2489	0.15	0.24	0.15	0.19
T. cruzi-negative_2490	0.19	0.28	0.26	0.19
T. cruzi-negative_2491	0.17	0.26	0.19	0.20
T. cruzi-negative_2492	0.18	0.23	0.19	0.21
T. cruzi-negative_2493	0.13	0.27	0.14	0.16
T. cruzi-negative_2494	0.36	0.44	0.29	0.39
T. cruzi-negative_2495	0.21	0.30	0.25	0.31
T. cruzi-negative_2496	0.26	0.38	0.25	0.28
T. cruzi-negative_2497	0.19	0.37	0.24	0.19
T. cruzi-negative_2498	0.33	0.36	0.30	0.36
T. cruzi-negative_2499	0.22	0.27	0.21	0.21
T. cruzi-negative_2500	0.23	0.22	0.22	0.20
T. cruzi-negative_2501	0.31	0.38	0.28	0.32
T. cruzi-negative_2502	0.25	0.26	0.31	0.18
T. cruzi-negative_2503	0.42	0.60	0.42	0.45
T. cruzi-negative_2504	0.22	0.34	0.21	0.35
T. cruzi-negative_2505	0.37	0.50	0.40	0.31
T. cruzi-negative_2506	0.15	0.17	0.16	0.10
T. cruzi-negative_2507	0.09	0.22	0.14	0.17
T. cruzi-negative_2508	0.38	0.31	0.26	0.27
T. cruzi-negative_2509	0.19	0.28	0.19	0.21
T. cruzi-negative_2510	0.24	0.32	0.21	0.27
T. cruzi-negative_2511	0.45	0.48	0.39	0.35
T. cruzi-negative_2512	0.22	0.25	0.20	0.19
T. cruzi-negative_2513	0.40	0.51	0.21	0.30
T. cruzi-negative_2514	0.40	0.41	0.36	0.34
T. cruzi-negative_2515	0.16	0.24	0.35	0.14
T. cruzi-negative_2516	0.31	0.38	0.27	0.30
T. cruzi-negative_2517	0.31	0.37	0.30	0.29
T. cruzi-negative_2518	0.47	0.38	0.37	0.28
T. cruzi-negative_2519	0.23	0.27	0.22	0.20

*T. cruz*i-negative_2520	0.23	0.27	0.26	0.20
*T. cruz*i-negative_2521	0.22	0.27	0.16	0.15
*T. cruz*i-negative_2522	0.23	0.42	0.19	0.30
*T. cruz*i-negative_2523	0.54	0.35	0.34	0.28
*T. cruz*i-negative_2524	0.10	0.49	0.42	0.42
*T. cruz*i-negative_2525	0.38	0.43	0.36	0.34
*T. cruz*i-negative_2526	0.32	0.32	0.31	0.33
*T. cruz*i-negative_2527	0.25	0.29	0.26	0.24
*T. cruz*i-negative_2528	0.31	0.28	0.32	0.22
*T. cruz*i-negative_2529	0.12	0.14	0.16	0.11
*T. cruz*i-negative_2530	0.10	0.13	0.12	0.10
*T. cruz*i-negative_2531	0.14	0.16	0.19	0.17
*T. cruz*i-negative_2532	0.30	0.39	0.32	0.28
*T. cruz*i-negative_2533	0.21	0.27	0.23	0.24
*T. cruz*i-negative_2534	0.19	0.22	0.17	0.16
*T. cruz*i-negative_2535	0.24	0.30	0.22	0.23
*T. cruz*i-negative_2536	0.45	0.52	0.34	0.41
*T. cruz*i-negative_2537	0.32	0.39	0.24	0.23
*T. cruz*i-negative_2538	0.25	0.27	0.21	0.16
*T. cruz*i-negative_2539	0.26	0.25	0.23	0.21
*T. cruz*i-negative_2540	0.13	0.14	0.14	0.12
*T. cruz*i-negative_2541	0.64	0.60	0.43	0.54
*T. cruz*i-negative_2542	0.13	0.16	0.12	0.11
*T. cruz*i-negative_2543	0.19	0.22	0.20	0.16
*T. cruz*i-negative_2544	0.12	0.18	0.13	0.13
*T. cruz*i-negative_2545	0.21	0.23	0.16	0.12
*T. cruz*i-negative_2546	0.26	0.34	0.23	0.20
*T. cruz*i-negative_2547	0.26	0.31	0.22	0.23
*T. cruz*i-negative_2548	0.29	0.30	0.26	0.25
*T. cruz*i-negative_2549	0.20	0.28	0.22	0.20
*T. cruz*i-negative_2550	0.36	0.31	0.28	0.27
*T. cruz*i-negative_2551	0.14	0.18	0.16	0.14
*T. cruz*i-negative_2552	0.44	0.42	0.35	0.34
*T. cruz*i-negative_2553	0.20	0.24	0.19	0.11
*T. cruz*i-negative_2554	0.49	0.53	0.44	0.29
*T. cruz*i-negative_2555	0.21	0.25	0.21	0.18
*T. cruz*i-negative_2556	0.31	0.38	0.17	0.24
*T. cruz*i-negative_2557	0.10	0.16	0.09	0.11
*T. cruz*i-negative_2558	0.19	0.31	0.14	0.15
*T. cruz*i-negative_2559	0.39	0.65	0.27	0.44
*T. cruz*i-negative_2560	0.18	0.30	0.15	0.18
*T. cruz*i-negative_2561	0.28	0.45	0.25	0.27
*T. cruz*i-negative_2562	0.23	0.37	0.24	0.22

*T. cruz*i-negative_2563	0.15	0.25	0.12	0.14
*T. cruz*i-negative_2564	0.20	0.42	0.15	0.17
*T. cruz*i-negative_2565	0.22	0.35	0.17	0.23
*T. cruz*i-negative_2566	0.21	0.34	0.20	0.20
*T. cruz*i-negative_2567	0.16	0.25	0.15	0.22
*T. cruz*i-negative_2568	0.11	0.29	0.11	0.17
*T. cruz*i-negative_2569	0.33	0.49	0.25	0.23
*T. cruz*i-negative_2570	0.21	0.30	0.16	0.14
*T. cruz*i-negative_2571	0.21	0.31	0.16	0.22
*T. cruz*i-negative_2572	0.17	0.21	0.12	0.14
*T. cruz*i-negative_2573	0.23	0.38	0.22	0.26
*T. cruz*i-negative_2574	0.20	0.32	0.15	0.18
*T. cruz*i-negative_2575	0.18	0.26	0.12	0.18
*T. cruz*i-negative_2576	0.30	0.72	0.22	0.28
*T. cruz*i-negative_2577	0.14	0.24	0.15	0.09
*T. cruz*i-negative_2578	0.23	0.29	0.14	0.19
*T. cruz*i-negative_2579	0.21	0.27	0.16	0.15
*T. cruz*i-negative_2580	0.14	0.23	0.12	0.14
*T. cruz*i-negative_2581	0.28	0.47	0.26	0.27
*T. cruz*i-negative_2582	0.16	0.31	0.15	0.15
*T. cruz*i-negative_2583	0.10	0.21	0.10	0.11
*T. cruz*i-negative_2584	0.19	0.36	0.15	0.23
*T. cruz*i-negative_2585	0.16	0.30	0.12	0.14
*T. cruz*i-negative_2586	0.17	0.23	0.13	0.14
*T. cruz*i-negative_2587	0.17	0.30	0.13	0.12
*T. cruz*i-negative_2588	0.13	0.21	0.12	0.12
*T. cruz*i-negative_2589	0.12	0.17	0.09	0.11
*T. cruz*i-negative_2590	0.16	0.28	0.14	0.17
*T. cruz*i-negative_2591	0.10	0.16	0.07	0.15
*T. cruz*i-negative_2592	0.23	0.47	0.16	0.28
*T. cruz*i-negative_2593	0.18	0.26	0.14	0.14
*T. cruz*i-negative_2594	0.22	0.31	0.11	0.19
*T. cruz*i-negative_2595	0.21	0.31	0.19	0.25
*T. cruz*i-negative_2596	0.18	0.27	0.14	0.18
*T. cruz*i-negative_2597	0.17	0.29	0.13	0.19
*T. cruz*i-negative_2598	0.21	0.39	0.24	0.25
*T. cruz*i-negative_2599	0.18	0.23	0.12	0.19
*T. cruz*i-negative_2600	0.18	0.38	0.14	0.25
*T. cruz*i-negative_2601	0.11	0.13	0.08	0.10
*T. cruz*i-negative_2602	0.27	0.38	0.16	0.30
*T. cruz*i-negative_2603	0.15	0.23	0.10	0.17
*T. cruz*i-negative_2604	0.17	0.26	0.12	0.19

T. cruzi-negative_2605	0.20	0.30	0.15	0.25
T. cruzi-negative_2606	0.31	0.43	0.26	0.36
T. cruzi-negative_2607	0.23	0.31	0.14	0.24
T. cruzi-negative_2608	0.24	0.41	0.17	0.30
T. cruzi-negative_2609	0.27	0.43	0.21	0.30
T. cruzi-negative_2610	0.11	0.13	0.07	0.11
T. cruzi-negative_2611	0.19	0.27	0.13	0.21
T. cruzi-negative_2612	0.17	0.28	0.13	0.20
T. cruzi-negative_2613	0.18	0.27	0.13	0.21
T. cruzi-negative_2614	0.30	0.43	0.24	0.27
T. cruzi-negative_2615	0.13	0.21	0.10	0.17
T. cruzi-negative_2616	0.23	0.42	0.18	0.22
T. cruzi-negative_2617	0.17	0.24	0.14	0.19
T. cruzi-negative_2618	0.19	0.20	0.12	0.19
T. cruzi-negative_2619	0.13	0.14	0.09	0.15
T. cruzi-negative_2620	0.21	0.36	0.18	0.32
T. cruzi-negative_2621	0.30	0.49	0.21	0.27
T. cruzi-negative_2622	0.13	0.20	0.11	0.13
T. cruzi-negative_2623	0.23	0.31	0.23	0.25
T. cruzi-negative_2624	0.21	0.39	0.15	0.19
T. cruzi-negative_2625	0.27	0.37	0.17	0.32
T. cruzi-negative_2626	0.31	0.39	0.17	0.36
T. cruzi-negative_2627	0.31	0.42	0.35	0.34
T. cruzi-negative_2628	0.20	0.34	0.15	0.27
T. cruzi-negative_2629	0.24	0.46	0.17	0.30
T. cruzi-negative_2630	0.27	0.38	0.20	0.28
T. cruzi-negative_2631	0.24	0.31	0.16	0.26
T. cruzi-negative_2632	0.24	0.34	0.18	0.26
T. cruzi-negative_2633	0.23	0.32	0.25	0.40
T. cruzi-negative_2634	0.24	0.40	0.21	0.27
T. cruzi-negative_2635	0.23	0.33	0.19	0.29
T. cruzi-negative_2636	0.14	0.24	0.11	0.23
T. cruzi-negative_2637	0.29	0.53	0.25	0.38
T. cruzi-negative_2638	0.29	0.39	0.42	0.20
T. cruzi-negative_2639	0.25	0.42	0.18	0.26
T. cruzi-negative_2640	0.22	0.19	0.06	0.13
T. cruzi-negative_2641	0.24	0.21	0.11	0.26
T. cruzi-negative_2642	0.21	0.21	0.10	0.22
T. cruzi-negative_2643	0.21	0.23	0.06	0.15
T. cruzi-negative_2644	0.29	0.40	0.18	0.27
T. cruzi-negative_2645	0.26	0.29	0.15	0.32
T. cruzi-negative_2646	0.26	0.29	0.23	0.38
T. cruzi-negative_2647	0.25	0.31	0.18	0.31

T. cruzi-negative_2648	0.28	0.34	0.14	0.25
T. cruzi-negative_2649	0.28	0.29	0.15	0.30
T. cruzi-negative_2650	0.19	0.20	0.10	0.17
T. cruzi-negative_2651	0.29	0.33	0.21	0.24
T. cruzi-negative_2652	0.21	0.41	0.10	0.18
T. cruzi-negative_2653	0.28	0.30	0.16	0.33
T. cruzi-negative_2654	0.25	0.27	0.08	0.25
T. cruzi-negative_2655	0.17	0.20	0.05	0.15
T. cruzi-negative_2656	0.24	0.29	0.09	0.22
T. cruzi-negative_2657	0.21	0.30	0.09	0.18
T. cruzi-negative_2658	0.18	0.22	0.07	0.17
T. cruzi-negative_2659	0.20	0.22	0.07	0.19
T. cruzi-negative_2660	0.23	0.29	0.11	0.28
T. cruzi-negative_2661	0.33	0.33	0.16	0.34
T. cruzi-negative_2662	0.26	0.38	0.14	0.31
T. cruzi-negative_2663	0.22	0.26	0.09	0.21
T. cruzi-negative_2664	0.25	0.34	0.11	0.25
T. cruzi-negative_2665	0.31	0.34	0.14	0.24
T. cruzi-negative_2666	0.27	0.34	0.13	0.26
T. cruzi-negative_2667	0.50	0.63	0.33	0.53
T. cruzi-negative_2668	0.41	0.62	0.23	0.44
T. cruzi-negative_2669	0.36	0.39	0.15	0.40
T. cruzi-negative_2670	0.25	0.28	0.09	0.24
T. cruzi-negative_2671	0.32	0.40	0.51	0.34
T. cruzi-negative_2672	0.33	0.42	0.13	0.30
T. cruzi-negative_2673	0.25	0.29	0.14	0.30
T. cruzi-negative_2674	0.44	0.63	0.27	0.58
T. cruzi-negative_2675	0.31	0.38	0.14	0.18
T. cruzi-negative_2676	0.38	0.47	0.17	0.40
T. cruzi-negative_2677	0.46	0.49	0.40	0.59
T. cruzi-negative_2678	0.25	0.34	0.17	0.41
T. cruzi-negative_2679	0.28	0.34	0.11	0.31
T. cruzi-negative_2680	0.33	0.38	0.15	0.33
T. cruzi-negative_2681	0.28	0.34	0.13	0.32
T. cruzi-negative_2682	0.25	0.32	0.10	0.22
T. cruzi-negative_2683	0.27	0.31	0.12	0.25
T. cruzi-negative_2684	0.35	0.42	0.22	0.38
T. cruzi-negative_2685	0.22	0.25	0.11	0.31
T. cruzi-negative_2686	0.20	0.22	0.09	0.23
T. cruzi-negative_2687	0.28	0.37	0.31	0.38
T. cruzi-negative_2688	0.25	0.33	0.19	0.26
T. cruzi-negative_2689	0.30	0.29	0.22	0.24

T. cruzi-negative_2690	0.22	0.28	0.11	0.30
T. cruzi-negative_2691	0.29	0.23	0.14	0.25
T. cruzi-negative_2692	0.28	0.41	0.43	0.44
T. cruzi-negative_2693	0.29	0.37	0.15	0.36
T. cruzi-negative_2694	0.31	0.46	0.14	0.52
T. cruzi-negative_2695	0.30	0.48	0.15	0.40
T. cruzi-negative_2696	0.39	0.39	0.14	0.42
T. cruzi-negative_2697	0.40	0.50	0.17	0.64
T. cruzi-negative_2698	0.76	0.72	0.83	1.33
T. cruzi-negative_2699	0.47	0.47	0.19	0.38
T. cruzi-negative_2700	0.27	0.38	0.17	0.51
T. cruzi-negative_2701	0.39	0.62	0.29	0.68
T. cruzi-negative_2702	0.25	0.29	0.08	0.28
T. cruzi-negative_2703	0.21	0.25	0.06	0.20
T. cruzi-negative_2704	0.35	0.58	0.28	0.38
T. cruzi-negative_2705	0.28	0.37	0.17	0.46
T. cruzi-negative_2706	0.27	0.34	0.12	0.27
T. cruzi-negative_2707	0.36	0.44	0.23	0.53
T. cruzi-negative_2708	0.23	0.30	0.07	0.23
T. cruzi-negative_2709	0.31	0.45	0.13	0.34
T. cruzi-negative_2710	0.30	0.39	0.11	0.34
T. cruzi-negative_2711	0.34	0.46	0.44	0.48
T. cruzi-negative_2712	0.31	0.42	0.20	0.38
T. cruzi-negative_2713	0.41	0.29	0.10	0.27
T. cruzi-negative_2714	0.24	0.40	0.14	0.20
T. cruzi-negative_2715	0.25	0.36	0.06	0.30
T. cruzi-negative_2716	0.24	0.37	0.10	0.33
T. cruzi-negative_2717	0.17	0.21	0.09	0.25
T. cruzi-negative_2718	0.27	0.34	0.12	0.33
T. cruzi-negative_2719	0.24	0.31	0.11	0.32
T. cruzi-negative_2720	0.21	0.29	0.07	0.22
T. cruzi-negative_2721	0.23	0.27	0.23	0.26
T. cruzi-negative_2722	0.25	0.29	0.08	0.21
T. cruzi-negative_2723	0.32	0.44	0.13	0.40
T. cruzi-negative_2724	0.14	0.13	0.21	0.30
T. cruzi-negative_2725	0.19	0.21	0.24	0.28
T. cruzi-negative_2726	0.14	0.12	0.30	0.19
T. cruzi-negative_2727	0.21	0.15	0.21	0.26
T. cruzi-negative_2728	0.19	0.23	0.19	0.25
T. cruzi-negative_2729	0.23	0.22	0.35	0.27
T. cruzi-negative_2730	0.17	0.10	0.16	0.25
T. cruzi-negative_2731	0.13	0.04	0.19	0.25
T. cruzi-negative_2732	0.27	0.56	0.21	0.44

T. cruzi-negative_2733	0.24	0.47	0.19	0.31
T. cruzi-negative_2734	0.25	0.40	0.21	0.36
T. cruzi-negative_2735	0.23	0.36	0.21	0.32
T. cruzi-negative_2736	0.27	0.35	0.22	0.26
T. cruzi-negative_2737	0.30	0.59	0.34	0.50
T. cruzi-negative_2738	0.16	0.25	0.15	0.24
T. cruzi-negative_2739	0.13	0.24	0.13	0.21
T. cruzi-negative_2740	0.12	0.17	0.12	0.20
T. cruzi-negative_2741	0.10	0.20	0.13	0.25
T. cruzi-negative_2742	0.18	0.32	0.15	0.30
T. cruzi-negative_2743	0.31	0.44	0.24	0.38
T. cruzi-negative_2744	0.26	0.31	0.16	0.22
T. cruzi-negative_2745	0.24	0.34	0.18	0.32
T. cruzi-negative_2746	0.22	0.34	0.14	0.30
T. cruzi-negative_2747	0.18	0.27	0.28	0.26
T. cruzi-negative_2748	0.28	0.47	0.31	0.31
T. cruzi-negative_2749	0.33	0.53	0.22	0.35
T. cruzi-negative_2750	0.16	0.37	0.19	0.32
T. cruzi-negative_2751	0.18	0.34	0.17	0.26
T. cruzi-negative_2752	0.18	0.17	0.12	0.15
T. cruzi-negative_2753	0.11	0.22	0.10	0.22
T. cruzi-negative_2754	0.15	0.37	0.12	0.16
T. cruzi-negative_2755	0.23	0.31	0.15	0.30
T. cruzi-negative_2756	0.30	0.39	0.29	0.31
T. cruzi-negative_2757	0.29	0.49	0.27	0.43
T. cruzi-negative_2758	0.24	0.28	0.16	0.24
T. cruzi-negative_2759	0.18	0.32	0.20	0.32
T. cruzi-negative_2760	0.38	0.41	0.25	0.27
T. cruzi-negative_2761	0.11	0.18	0.12	0.21
T. cruzi-negative_2762	0.36	0.50	0.19	0.40
T. cruzi-negative_2763	0.24	0.37	0.17	0.38
T. cruzi-negative_2764	0.19	0.33	0.15	0.25
T. cruzi-negative_2765	0.13	0.19	0.10	0.21
T. cruzi-negative_2766	0.26	0.43	0.22	0.38
T. cruzi-negative_2767	0.20	0.38	0.21	0.34
T. cruzi-negative_2768	0.31	0.32	0.17	0.26
T. cruzi-negative_2769	0.25	0.25	0.16	0.28
T. cruzi-negative_2770	0.31	0.59	0.27	0.45
T. cruzi-negative_2771	0.39	0.49	0.25	0.49
T. cruzi-negative_2772	0.14	0.22	0.10	0.20
T. cruzi-negative_2773	0.42	0.56	0.27	0.51
T. cruzi-negative_2774	0.17	0.47	0.14	0.34

*T. cruz*i-negative_2775	0.38	0.68	0.36	0.55
*T. cruz*i-negative_2776	0.36	0.29	0.23	0.24
*T. cruz*i-negative_2777	0.33	0.41	0.21	0.43
*T. cruz*i-negative_2778	0.30	0.49	0.44	0.34
*T. cruz*i-negative_2779	0.23	0.32	0.17	0.27
*T. cruz*i-negative_2780	0.28	0.33	0.18	0.27
*T. cruz*i-negative_2781	0.16	0.26	0.13	0.20
*T. cruz*i-negative_2782	0.31	0.67	0.27	0.50
*T. cruz*i-negative_2783	0.31	0.46	0.26	0.40
*T. cruz*i-negative_2784	0.36	0.37	0.16	0.29
*T. cruz*i-negative_2785	0.31	0.49	0.21	0.34
*T. cruz*i-negative_2786	0.27	0.50	0.19	0.23
*T. cruz*i-negative_2787	0.21	0.33	0.15	0.24
*T. cruz*i-negative_2788	0.37	0.51	0.21	0.38
*T. cruz*i-negative_2789	0.25	0.46	0.21	0.32
*T. cruz*i-negative_2790	0.25	0.19	0.10	0.18
*T. cruz*i-negative_2791	0.29	0.32	0.22	0.42
*T. cruz*i-negative_2792	0.34	0.45	0.67	0.31
*T. cruz*i-negative_2793	0.27	0.36	0.19	0.35
*T. cruz*i-negative_2794	0.21	0.40	0.14	0.29
*T. cruz*i-negative_2795	0.22	0.34	0.17	0.26
*T. cruz*i-negative_2796	0.37	0.56	0.21	0.38
*T. cruz*i-negative_2797	0.37	0.51	0.38	0.41
*T. cruz*i-negative_2798	0.31	0.59	0.21	0.46
*T. cruz*i-negative_2799	0.14	0.29	0.18	0.29
*T. cruz*i-negative_2800	0.20	0.23	0.10	0.15
*T. cruz*i-negative_2801	0.23	0.32	0.14	0.18
*T. cruz*i-negative_2802	0.30	0.42	0.24	0.16
*T. cruz*i-negative_2803	0.20	0.23	0.11	0.13
*T. cruz*i-negative_2804	0.32	0.53	0.26	0.25
*T. cruz*i-negative_2805	0.24	0.31	0.15	0.16
*T. cruz*i-negative_2806	0.27	0.32	0.16	0.19
*T. cruz*i-negative_2807	0.17	0.24	0.14	0.19
*T. cruz*i-negative_2808	0.42	0.50	0.16	0.23
*T. cruz*i-negative_2809	0.45	0.62	0.21	0.45
*T. cruz*i-negative_2810	0.34	0.45	0.24	0.34
*T. cruz*i-negative_2811	0.19	0.36	0.16	0.26
*T. cruz*i-negative_2812	0.34	0.51	0.21	0.34
*T. cruz*i-negative_2813	0.32	0.54	0.15	0.25
*T. cruz*i-negative_2814	0.33	0.33	0.18	0.25
*T. cruz*i-negative_2815	0.47	0.68	0.36	0.54
*T. cruz*i-negative_2816	0.26	0.12	0.07	0.19
*T. cruz*i-negative_2817	0.33	0.27	0.24	0.37

*T. cruz*i-negative_2818	0.16	0.25	0.10	0.30
*T. cruz*i-negative_2819	0.21	0.12	0.09	0.21
*T. cruz*i-negative_2820	0.19	0.16	0.14	0.40
*T. cruz*i-negative_2821	0.25	0.23	0.18	0.25
*T. cruz*i-negative_2822	0.20	0.17	0.08	0.20
*T. cruz*i-negative_2823	0.16	0.20	0.13	0.26
*T. cruz*i-negative_2824	0.20	0.16	0.05	0.20
*T. cruz*i-negative_2825	0.29	0.37	0.21	0.26
*T. cruz*i-negative_2826	0.13	0.17	0.10	0.21
*T. cruz*i-negative_2827	0.11	0.12	0.07	0.16
*T. cruz*i-negative_2828	0.25	0.10	0.13	0.42
*T. cruz*i-negative_2829	0.20	0.28	0.20	0.30
*T. cruz*i-negative_2830	0.23	0.25	0.29	0.27
*T. cruz*i-negative_2831	0.32	0.25	0.18	0.31
*T. cruz*i-negative_2832	0.22	0.14	0.09	0.20
*T. cruz*i-negative_2833	0.47	0.19	0.12	0.20
*T. cruz*i-negative_2834	0.22	0.25	0.14	0.30
*T. cruz*i-negative_2835	0.21	0.26	0.18	0.19
*T. cruz*i-negative_2836	0.27	0.30	0.35	0.64
*T. cruz*i-negative_2837	0.20	0.30	0.19	0.25
*T. cruz*i-negative_2838	0.21	0.31	0.16	0.28
*T. cruz*i-negative_2839	0.43	0.42	0.27	0.43
*T. cruz*i-negative_2840	0.13	0.19	0.05	0.16
*T. cruz*i-negative_2841	0.29	0.34	0.20	0.30
*T. cruz*i-negative_2842	0.15	0.33	0.10	0.22
*T. cruz*i-negative_2843	0.15	0.18	0.05	0.30
*T. cruz*i-negative_2844	0.44	0.39	0.19	0.71
*T. cruz*i-negative_2845	0.21	0.18	0.16	0.22
*T. cruz*i-negative_2846	0.15	0.17	0.06	0.19
*T. cruz*i-negative_2847	0.30	0.38	0.19	0.35
*T. cruz*i-negative_2848	0.21	0.23	0.22	0.28
*T. cruz*i-negative_2849	0.06	0.11	0.02	0.11
*T. cruz*i-negative_2850	0.12	0.16	0.17	0.20
*T. cruz*i-negative_2851	0.13	0.29	0.16	0.38
*T. cruz*i-negative_2852	0.28	0.43	0.23	0.62
*T. cruz*i-negative_2853	0.15	0.20	0.08	0.21
*T. cruz*i-negative_2854	0.14	0.19	0.14	0.22
*T. cruz*i-negative_2855	0.13	0.21	0.21	0.25
*T. cruz*i-negative_2856	0.13	0.06	0.08	0.18
*T. cruz*i-negative_2857	0.14	0.18	0.08	0.21
*T. cruz*i-negative_2858	0.26	0.26	0.16	0.29
*T. cruz*i-negative_2859	0.20	0.22	0.12	0.24

T. cruzi-negative_2860	0.19	0.21	0.14	0.39
T. cruzi-negative_2861	0.07	0.08	0.02	0.17
T. cruzi-negative_2862	0.16	0.23	0.12	0.29
T. cruzi-negative_2863	0.14	0.28	0.15	0.28
T. cruzi-negative_2864	0.21	0.32	0.34	0.29
T. cruzi-negative_2865	0.19	0.27	0.14	0.23
T. cruzi-negative_2866	0.15	0.16	0.11	0.21
T. cruzi-negative_2867	0.42	0.32	0.21	0.30
T. cruzi-negative_2868	0.11	0.16	0.10	0.34
T. cruzi-negative_2869	0.28	0.38	0.26	0.40
T. cruzi-negative_2870	0.16	0.25	0.15	0.31
T. cruzi-negative_2871	0.26	0.45	0.31	0.36
T. cruzi-negative_2872	0.10	0.17	0.10	0.21
T. cruzi-negative_2873	0.16	0.25	0.18	0.28
T. cruzi-negative_2874	0.12	0.21	0.12	0.21
T. cruzi-negative_2875	0.22	0.29	0.15	0.24
T. cruzi-negative_2876	0.23	0.39	0.17	0.61
T. cruzi-negative_2877	0.32	0.42	0.29	0.48
T. cruzi-negative_2878	0.17	0.29	0.21	0.35
T. cruzi-negative_2879	0.21	0.28	0.20	0.28
T. cruzi-negative_2880	0.18	0.23	0.15	0.23
T. cruzi-negative_2881	0.21	0.30	0.14	0.24
T. cruzi-negative_2882	0.15	0.19	0.11	0.27
T. cruzi-negative_2883	0.15	0.29	0.16	0.29
T. cruzi-negative_2884	0.22	0.30	0.23	0.55
T. cruzi-negative_2885	0.44	0.27	0.25	0.33
T. cruzi-negative_2886	0.07	0.09	0.10	0.22
T. cruzi-negative_2887	0.01	0.02	0.18	0.23
T. cruzi-negative_2888	0.16	0.29	0.19	0.30
T. cruzi-negative_2889	0.19	0.21	0.19	0.25
T. cruzi-negative_2890	0.27	0.43	0.34	0.36
T. cruzi-negative_2891	0.20	0.29	0.19	0.27
T. cruzi-negative_2892	0.26	0.55	0.33	0.73
T. cruzi-negative_2893	0.24	0.29	0.23	0.42
T. cruzi-negative_2894	0.20	0.19	0.21	0.26
T. cruzi-negative_2895	0.09	0.06	0.74	0.13
T. cruzi-negative_2896	0.15	0.14	0.15	0.22
T. cruzi-negative_2897	0.20	0.18	0.36	0.29
T. cruzi-negative_2898	0.27	0.19	0.18	0.28
T. cruzi-negative_2899	0.12	0.29	0.08	0.24
T. cruzi-negative_2900	0.16	0.46	0.20	0.07
T. cruzi-negative_2901	0.05	0.40	0.22	0.09
T. cruzi-negative_2902	0.17	0.41	0.23	0.06

T. cruzi-negative_2903	0.01	0.19	0.09	0.02
T. cruzi-negative_2904	0.22	0.24	0.23	0.06
T. cruzi-negative_2905	0.14	0.33	0.30	0.14
T. cruzi-negative_2906	0.13	0.25	0.30	0.26
T. cruzi-negative_2907	0.05	0.37	0.20	0.11
T. cruzi-negative_2908	0.08	0.23	0.20	0.07
T. cruzi-negative_2909	0.15	0.40	0.25	0.08
T. cruzi-negative_2910	0.02	0.21	0.14	0.05
T. cruzi-negative_2911	0.07	0.21	0.14	0.02
T. cruzi-negative_2912	0.15	0.30	0.27	0.01
T. cruzi-negative_2913	0.13	0.27	0.12	0.08
T. cruzi-negative_2914	0.19	0.43	0.23	0.18
T. cruzi-negative_2915	0.11	0.46	0.24	0.09
T. cruzi-negative_2916	0.04	0.42	0.28	0.14
T. cruzi-negative_2917	0.05	0.28	0.17	0.03
T. cruzi-negative_2918	0.07	0.22	0.10	0.04
T. cruzi-negative_2919	0.08	0.19	0.23	0.01
T. cruzi-negative_2920	0.26	0.14	0.09	0.07
T. cruzi-negative_2921	0.09	0.29	0.39	0.24
T. cruzi-negative_2922	0.04	0.32	0.40	0.10
T. cruzi-negative_2923	0.23	0.43	0.27	0.19
T. cruzi-negative_2924	0.04	0.17	0.08	0.06
T. cruzi-negative_2925	0.08	0.17	0.19	0.06
T. cruzi-negative_2926	0.05	0.21	0.19	0.05
T. cruzi-negative_2927	0.10	0.19	0.12	0.01
T. cruzi-negative_2928	0.11	0.13	0.05	0.01
T. cruzi-negative_2929	0.01	0.49	0.05	0.03
T. cruzi-negative_2930	0.09	0.25	0.11	0.05
T. cruzi-negative_2931	0.06	0.32	0.09	0.06
T. cruzi-negative_2932	0.08	0.25	0.19	0.08
T. cruzi-negative_2933	0.07	0.22	0.21	0.03
T. cruzi-negative_2934	0.14	0.37	0.16	0.11
T. cruzi-negative_2935	0.09	0.31	0.47	0.11
T. cruzi-negative_2936	0.07	0.28	0.14	0.01
T. cruzi-negative_2937	0.13	0.44	0.32	0.21
T. cruzi-negative_2938	0.09	0.32	0.22	0.14
T. cruzi-negative_2939	0.07	0.28	0.17	0.06
T. cruzi-negative_2940	0.07	0.35	0.21	0.14
T. cruzi-negative_2941	0.07	0.33	0.13	0.02
T. cruzi-negative_2942	0.17	0.42	0.21	0.08
T. cruzi-negative_2943	0.06	0.23	0.18	0.08
T. cruzi-negative_2944	0.04	0.15	0.07	0.01

T. cruzi-negative_2945	0.10	0.28	0.16	0.11
T. cruzi-negative_2946	0.01	0.25	0.21	0.09
T. cruzi-negative_2947	0.07	0.20	0.10	0.07
T. cruzi-negative_2948	0.01	0.18	0.06	0.03
T. cruzi-negative_2949	0.07	0.15	0.05	0.01
T. cruzi-negative_2950	0.09	0.29	0.12	0.06
T. cruzi-negative_2951	0.05	0.17	0.14	0.04
T. cruzi-negative_2952	0.12	0.49	0.11	0.03
T. cruzi-negative_2953	0.04	0.15	0.11	0.11
T. cruzi-negative_2954	0.13	0.27	0.25	0.16
T. cruzi-negative_2955	0.14	0.43	0.18	0.11
T. cruzi-negative_2956	0.01	0.31	0.08	0.08
T. cruzi-negative_2957	0.02	0.15	0.05	0.03
T. cruzi-negative_2958	0.09	0.28	0.09	0.04
T. cruzi-negative_2959	0.17	0.39	0.21	0.10
T. cruzi-negative_2960	0.06	0.19	0.15	0.06
T. cruzi-negative_2961	0.03	0.17	0.20	0.15
T. cruzi-negative_2962	0.04	0.22	0.05	0.07
T. cruzi-negative_2963	0.17	0.38	0.11	0.13
T. cruzi-negative_2964	0.01	0.29	0.09	0.11
T. cruzi-negative_2965	0.04	0.22	0.11	0.06
T. cruzi-negative_2966	0.03	0.18	0.05	0.01
T. cruzi-negative_2967	0.08	0.09	0.06	0.02
T. cruzi-negative_2968	0.05	0.10	0.10	0.01
T. cruzi-negative_2969	0.05	0.19	0.12	0.12
T. cruzi-negative_2970	0.01	0.18	0.15	0.15
T. cruzi-negative_2971	0.15	0.34	0.11	0.14
T. cruzi-negative_2972	0.01	0.19	0.11	0.07
T. cruzi-negative_2973	0.21	0.40	0.19	0.11
T. cruzi-negative_2974	0.03	0.41	0.26	0.13
T. cruzi-negative_2975	0.05	0.13	0.04	0.04
T. cruzi-negative_2976	0.10	0.23	0.07	0.18
T. cruzi-negative_2977	0.01	0.26	0.26	0.15
T. cruzi-negative_2978	0.09	0.37	0.17	0.17
T. cruzi-negative_2979	0.08	0.09	0.08	0.12
T. cruzi-negative_2980	0.09	0.34	0.16	0.17
T. cruzi-negative_2981	0.09	0.34	0.22	0.12
T. cruzi-negative_2982	0.08	0.31	0.13	0.21
T. cruzi-negative_2983	0.03	0.11	0.09	0.17
T. cruzi-negative_2984	0.11	0.19	0.16	0.12
T. cruzi-negative_2985	0.11	0.19	0.17	0.12
T. cruzi-negative_2986	0.04	0.34	0.20	0.47
T. cruzi-negative_2987	0.17	0.33	0.17	0.33

T. cruzi-negative_2988	0.13	0.49	0.24	0.29
T. cruzi-negative_2989	0.12	0.31	0.15	0.46
T. cruzi-negative_2990	0.19	0.40	0.60	0.52
T. cruzi-negative_2991	0.03	0.45	0.27	0.61
T. cruzi-negative_2992	0.31	0.59	0.24	0.59
T. cruzi-negative_2993	0.10	0.34	0.17	0.39
T. cruzi-negative_2994	0.06	0.31	0.14	0.44
T. cruzi-negative_2995	0.14	0.31	0.16	0.28
T. cruzi-negative_2996	0.01	0.55	0.22	0.14
T. cruzi-negative_2997	0.17	0.24	0.15	0.42
T. cruzi-negative_2998	0.15	0.35	0.61	0.58
T. cruzi-negative_2999	0.38	0.55	0.17	0.60
T. cruzi-negative_3000	0.10	0.27	0.12	0.26
T. cruzi-negative_3001	0.28	0.34	0.20	0.33
T. cruzi-negative_3002	0.04	0.18	0.14	0.28
T. cruzi-negative_3003	0.57	0.50	0.30	0.47
T. cruzi-negative_3004	0.10	0.39	0.16	0.23
T. cruzi-negative_3005	0.27	0.41	0.23	0.65
T. cruzi-negative_3006	0.16	0.37	0.53	0.74
T. cruzi-negative_3007	0.23	0.54	0.18	0.66
T. cruzi-negative_3008	0.15	0.43	0.17	0.45
T. cruzi-negative_3009	0.15	0.33	0.21	0.40
T. cruzi-negative_3010	0.26	0.39	0.19	0.49
T. cruzi-negative_3011	0.02	0.22	0.12	0.18
T. cruzi-negative_3012	0.11	0.42	0.16	0.27
T. cruzi-negative_3013	0.25	0.47	0.25	0.72
T. cruzi-negative_3014	0.15	0.41	0.20	0.16
T. cruzi-negative_3015	0.10	0.35	0.20	0.51
T. cruzi-negative_3016	0.18	0.44	0.18	0.38
T. cruzi-negative_3017	0.12	0.36	0.19	0.31
T. cruzi-negative_3018	0.23	0.48	0.22	0.62
T. cruzi-negative_3019	0.16	0.33	0.16	0.27
T. cruzi-negative_3020	0.10	0.50	0.21	0.35
T. cruzi-negative_3021	0.24	0.18	0.20	0.56
T. cruzi-negative_3022	0.23	0.46	0.58	0.69
T. cruzi-negative_3023	0.07	0.30	0.13	0.76
T. cruzi-negative_3024	0.09	0.28	0.15	0.30
T. cruzi-negative_3025	0.22	0.26	0.22	0.48
T. cruzi-negative_3026	0.19	0.39	0.24	0.56
T. cruzi-negative_3027	0.12	0.44	0.18	0.23
T. cruzi-negative_3028	0.21	0.35	0.15	0.26
T. cruzi-negative_3029	0.15	0.23	0.18	0.14

*T. cruz*i-negative_3030	0.21	0.36	0.67	0.74
*T. cruz*i-negative_3031	0.01	0.25	0.10	0.31
*T. cruz*i-negative_3032	0.15	0.32	0.15	0.29
*T. cruz*i-negative_3033	0.15	0.20	0.26	0.18
*T. cruz*i-negative_3034	0.13	0.32	0.16	0.60
*T. cruz*i-negative_3035	0.03	0.35	0.16	0.17
*T. cruz*i-negative_3036	0.30	0.71	0.23	0.30
*T. cruz*i-negative_3037	0.20	0.26	0.22	0.74
*T. cruz*i-negative_3038	0.37	0.56	0.65	0.79
*T. cruz*i-negative_3039	0.10	0.20	0.10	0.38
*T. cruz*i-negative_3040	0.24	0.35	0.17	0.45
*T. cruz*i-negative_3041	0.19	0.29	0.19	0.38
*T. cruz*i-negative_3042	0.10	0.35	0.27	0.56
*T. cruz*i-negative_3043	0.40	0.44	0.21	0.38
*T. cruz*i-negative_3044	0.13	0.62	0.24	0.25
*T. cruz*i-negative_3045	0.24	0.35	0.21	0.46
*T. cruz*i-negative_3046	0.33	0.49	0.66	0.53
*T. cruz*i-negative_3047	0.17	0.40	0.18	0.32
*T. cruz*i-negative_3048	0.49	0.51	0.31	0.53
*T. cruz*i-negative_3049	0.06	0.39	0.23	0.39
*T. cruz*i-negative_3050	0.18	0.41	0.24	0.55
*T. cruz*i-negative_3051	0.39	0.60	0.37	0.37
*T. cruz*i-negative_3052	0.15	0.27	0.13	0.23
*T. cruz*i-negative_3053	0.19	0.23	0.17	0.42
*T. cruz*i-negative_3054	0.18	0.38	0.27	0.27
*T. cruz*i-negative_3055	0.18	0.28	0.24	0.46
*T. cruz*i-negative_3056	0.04	0.22	0.14	0.24
*T. cruz*i-negative_3057	0.21	0.35	0.17	0.34
*T. cruz*i-negative_3058	0.35	0.59	0.33	0.74
*T. cruz*i-negative_3059	0.02	0.43	0.24	0.37
*T. cruz*i-negative_3060	0.04	0.45	0.13	0.24
*T. cruz*i-negative_3061	0.29	0.40	0.32	0.33
*T. cruz*i-negative_3062	0.14	0.41	0.65	0.53
*T. cruz*i-negative_3063	0.25	0.58	0.29	0.73
*T. cruz*i-negative_3064	0.38	0.69	0.34	0.65
*T. cruz*i-negative_3065	0.03	0.35	0.16	0.31
*T. cruz*i-negative_3066	0.10	0.34	0.16	0.31
*T. cruz*i-negative_3067	0.39	0.51	0.25	0.43
*T. cruz*i-negative_3068	0.21	0.20	0.10	0.14
*T. cruz*i-negative_3069	0.09	0.15	0.07	0.10
*T. cruz*i-negative_3070	0.21	0.27	0.18	0.20
*T. cruz*i-negative_3071	0.10	0.24	0.15	0.20
*T. cruz*i-negative_3072	0.12	0.22	0.11	0.10

*T. cruz*i-negative_3073	0.18	0.27	0.21	0.16
*T. cruz*i-negative_3074	0.43	0.61	0.54	0.31
*T. cruz*i-negative_3075	0.10	0.19	0.14	0.13
*T. cruz*i-negative_3076	0.06	0.12	0.05	0.08
*T. cruz*i-negative_3077	0.22	0.33	0.22	0.24
*T. cruz*i-negative_3078	0.11	0.25	0.12	0.17
*T. cruz*i-negative_3079	0.11	0.19	0.14	0.18
*T. cruz*i-negative_3080	0.04	0.10	0.14	0.19
*T. cruz*i-negative_3081	0.17	0.23	0.13	0.16
*T. cruz*i-negative_3082	0.06	0.19	0.09	0.11
*T. cruz*i-negative_3083	0.21	0.15	0.14	0.13
*T. cruz*i-negative_3084	0.02	0.18	0.09	0.14
*T. cruz*i-negative_3085	0.09	0.15	0.07	0.09
*T. cruz*i-negative_3086	0.12	0.18	0.11	0.13
*T. cruz*i-negative_3087	0.19	0.28	0.20	0.23
*T. cruz*i-negative_3088	0.08	0.20	0.11	0.19
*T. cruz*i-negative_3089	0.03	0.15	0.05	0.07
*T. cruz*i-negative_3090	0.05	0.11	0.05	0.06
*T. cruz*i-negative_3091	0.13	0.18	0.10	0.11
*T. cruz*i-negative_3092	0.09	0.16	0.08	0.12
*T. cruz*i-negative_3093	0.14	0.24	0.13	0.15
*T. cruz*i-negative_3094	0.12	0.20	0.12	0.18
*T. cruz*i-negative_3095	0.08	0.15	0.08	0.15
*T. cruz*i-negative_3096	0.09	0.15	0.09	0.24
*T. cruz*i-negative_3097	0.13	0.27	0.14	0.20
*T. cruz*i-negative_3098	0.11	0.20	0.18	0.19
*T. cruz*i-negative_3099	0.02	0.12	0.04	0.12
*T. cruz*i-negative_3100	0.12	0.18	0.10	0.15
*T. cruz*i-negative_3101	0.10	0.16	0.09	0.11
*T. cruz*i-negative_3102	0.17	0.21	0.18	0.23
*T. cruz*i-negative_3103	0.24	0.34	0.20	0.31
*T. cruz*i-negative_3104	0.10	0.25	0.08	0.25
*T. cruz*i-negative_3105	0.06	0.22	0.12	0.31
*T. cruz*i-negative_3106	0.04	0.12	0.05	0.11
*T. cruz*i-negative_3107	0.15	0.25	0.14	0.20
*T. cruz*i-negative_3108	0.14	0.25	0.11	0.22
*T. cruz*i-negative_3109	0.16	0.29	0.14	0.26
*T. cruz*i-negative_3110	0.07	0.19	0.11	0.19
*T. cruz*i-negative_3111	0.09	0.16	0.17	0.16
*T. cruz*i-negative_3112	0.16	0.22	0.10	0.24
*T. cruz*i-negative_3113	0.05	0.20	0.10	0.20
*T. cruz*i-negative_3114	0.09	0.19	0.06	0.16

T. cruzi-negative_3115	0.10	0.20	0.21	0.19
T. cruzi-negative_3116	0.09	0.16	0.07	0.15
T. cruzi-negative_3117	0.21	0.25	0.17	0.24
T. cruzi-negative_3118	0.01	0.15	0.07	0.10
T. cruzi-negative_3119	0.18	0.21	0.12	0.21
T. cruzi-negative_3120	0.05	0.17	0.15	0.18
T. cruzi-negative_3121	0.06	0.20	0.09	0.15
T. cruzi-negative_3122	0.20	0.32	0.12	0.22
T. cruzi-negative_3123	0.01	0.16	0.07	0.12
T. cruzi-negative_3124	0.11	0.19	0.10	0.13
T. cruzi-negative_3125	0.12	0.22	0.12	0.16
T. cruzi-negative_3126	0.06	0.15	0.11	0.16
T. cruzi-negative_3127	0.21	0.27	0.18	0.35
T. cruzi-negative_3128	0.14	0.21	0.13	0.25
T. cruzi-negative_3129	0.06	0.24	0.10	0.17
T. cruzi-negative_3130	0.08	0.15	0.09	0.16
T. cruzi-negative_3131	0.15	0.23	0.15	0.19
T. cruzi-negative_3132	0.01	0.10	0.03	0.11
T. cruzi-negative_3133	0.04	0.08	0.09	0.14
T. cruzi-negative_3134	0.09	0.10	0.06	0.09
T. cruzi-negative_3135	0.58	0.16	0.10	0.17
T. cruzi-negative_3136	0.07	0.18	0.11	0.15
T. cruzi-negative_3137	0.05	0.13	0.13	0.11
T. cruzi-negative_3138	0.07	0.10	0.05	0.08
T. cruzi-negative_3139	0.15	0.22	0.13	0.25
T. cruzi-negative_3140	0.05	0.16	0.11	0.15
T. cruzi-negative_3141	0.09	0.17	0.10	0.12
T. cruzi-negative_3142	0.18	0.30	0.15	0.20
T. cruzi-negative_3143	0.14	0.30	0.31	0.30
T. cruzi-negative_3144	0.01	0.18	0.07	0.11
T. cruzi-negative_3145	0.23	0.35	0.17	0.21
T. cruzi-negative_3146	0.05	0.20	0.10	0.15
T. cruzi-negative_3147	0.20	0.36	0.19	0.25
T. cruzi-negative_3148	0.68	0.92	0.74	0.74
T. cruzi-negative_3149	0.27	0.13	0.08	0.08
T. cruzi-negative_3150	0.13	0.43	0.11	0.14
T. cruzi-negative_3151	0.12	0.13	0.06	0.08
T. cruzi-negative_3152	0.10	0.16	0.07	0.17
T. cruzi-negative_3153	0.34	0.25	0.11	0.27
T. cruzi-negative_3154	0.05	0.32	0.20	0.24
T. cruzi-negative_3155	0.25	0.23	0.17	0.22
T. cruzi-negative_3156	0.16	0.38	0.23	0.33
T. cruzi-negative_3157	0.02	0.21	0.14	0.17

T. cruzi-negative_3158	0.41	0.49	0.31	0.54
T. cruzi-negative_3159	0.07	0.20	0.16	0.23
T. cruzi-negative_3160	0.23	0.29	0.25	0.33
T. cruzi-negative_3161	0.12	0.30	0.18	0.30
T. cruzi-negative_3162	0.08	0.30	0.19	0.33
T. cruzi-negative_3163	0.07	0.25	0.18	0.26
T. cruzi-negative_3164	0.17	0.25	0.17	0.26
T. cruzi-negative_3165	0.06	0.26	0.17	0.23
T. cruzi-negative_3166	0.09	0.21	0.15	0.23
T. cruzi-negative_3167	0.30	0.42	0.33	0.42
T. cruzi-negative_3168	0.02	0.23	0.17	0.26
T. cruzi-negative_3169	0.09	0.20	0.18	0.26
T. cruzi-negative_3170	0.12	0.31	0.20	0.35
T. cruzi-negative_3171	0.12	0.14	0.16	0.22
T. cruzi-negative_3172	0.16	0.23	0.35	0.23
T. cruzi-negative_3173	0.09	0.31	0.24	0.29
T. cruzi-negative_3174	0.14	0.24	0.18	0.31
T. cruzi-negative_3175	0.08	0.30	0.19	0.26
T. cruzi-negative_3176	0.09	0.20	0.18	0.28
T. cruzi-negative_3177	0.15	0.20	0.21	0.25
T. cruzi-negative_3178	0.13	0.33	0.21	0.34
T. cruzi-negative_3179	0.15	0.26	0.18	0.26
T. cruzi-negative_3180	0.09	0.25	0.16	0.28
T. cruzi-negative_3181	0.06	0.21	0.17	0.25
T. cruzi-negative_3182	0.11	0.25	0.24	0.32
T. cruzi-negative_3183	0.09	0.23	0.23	0.29
T. cruzi-negative_3184	0.07	0.27	0.13	0.23
T. cruzi-negative_3185	0.16	0.24	0.17	0.25
T. cruzi-negative_3186	0.21	0.37	0.24	0.32
T. cruzi-negative_3187	0.01	0.17	0.15	0.22
T. cruzi-negative_3188	0.19	0.23	0.17	0.29
T. cruzi-negative_3189	0.06	0.26	0.13	0.20
T. cruzi-negative_3190	0.11	0.23	0.15	0.28
T. cruzi-negative_3191	0.23	0.45	0.29	0.45
T. cruzi-negative_3192	0.06	0.18	0.10	0.16
T. cruzi-negative_3193	0.16	0.26	0.17	0.29
T. cruzi-negative_3194	0.04	0.17	0.11	0.21
T. cruzi-negative_3195	0.09	0.28	0.18	0.25
T. cruzi-negative_3196	0.12	0.24	0.17	0.22
T. cruzi-negative_3197	0.06	0.21	0.18	0.30
T. cruzi-negative_3198	0.06	0.11	0.15	0.19
T. cruzi-negative_3199	0.22	0.18	0.19	0.31

*T. cruz*i-negative_3200	0.05	0.31	0.25	0.38
*T. cruz*i-negative_3201	0.12	0.30	0.26	0.35
*T. cruz*i-negative_3202	0.06	0.18	0.15	0.24
*T. cruz*i-negative_3203	0.24	0.38	0.29	0.43
*T. cruz*i-negative_3204	0.01	0.23	0.16	0.19
*T. cruz*i-negative_3205	0.12	0.22	0.31	0.21
*T. cruz*i-negative_3206	0.07	0.12	0.13	0.19
*T. cruz*i-negative_3207	0.11	0.27	0.17	0.25
*T. cruz*i-negative_3208	0.17	0.43	0.23	0.36
*T. cruz*i-negative_3209	0.03	0.14	0.13	0.17
*T. cruz*i-negative_3210	0.18	0.22	0.18	0.27
*T. cruz*i-negative_3211	0.05	0.13	0.15	0.18
*T. cruz*i-negative_3212	0.06	0.14	0.11	0.14
*T. cruz*i-negative_3213	0.13	0.15	0.22	0.30
*T. cruz*i-negative_3214	0.08	0.16	0.25	0.26
*T. cruz*i-negative_3215	0.20	0.28	0.24	0.29
*T. cruz*i-negative_3216	0.06	0.26	0.24	0.23
*T. cruz*i-negative_3217	0.04	0.19	0.19	0.28
*T. cruz*i-negative_3218	0.07	0.10	0.26	0.23
*T. cruz*i-negative_3219	0.18	0.22	0.32	0.32
*T. cruz*i-negative_3220	0.08	0.17	0.18	0.21
*T. cruz*i-negative_3221	0.14	0.21	0.23	0.35
*T. cruz*i-negative_3222	0.05	0.07	0.23	0.30
*T. cruz*i-negative_3223	0.05	0.10	0.15	0.18
*T. cruz*i-negative_3224	0.10	0.22	0.46	0.26
*T. cruz*i-negative_3225	0.06	0.19	0.15	0.22
*T. cruz*i-negative_3226	0.07	0.18	0.17	0.27
*T. cruz*i-negative_3227	0.12	0.12	0.20	0.21
*T. cruz*i-negative_3228	0.19	0.31	0.31	0.27
*T. cruz*i-negative_3229	0.05	0.11	0.17	0.25
*T. cruz*i-negative_3230	0.05	0.10	0.13	0.16
*T. cruz*i-negative_3231	0.09	0.14	0.14	0.17
*T. cruz*i-negative_3232	0.15	0.27	0.77	0.51
*T. cruz*i-negative_3233	0.11	0.20	0.29	0.34
*T. cruz*i-negative_3234	0.10	0.13	0.19	0.31
*T. cruz*i-negative_3235	0.24	0.17	0.18	0.24
*T. cruz*i-negative_3236	0.13	0.30	0.23	0.27
*T. cruz*i-negative_3237	0.08	0.26	0.16	0.24
*T. cruz*i-negative_3238	0.16	0.34	0.28	0.24
*T. cruz*i-negative_3239	0.14	0.34	0.28	0.20
*T. cruz*i-negative_3240	0.09	0.23	0.14	0.21
*T. cruz*i-negative_3241	0.11	0.23	0.21	0.28
*T. cruz*i-negative_3242	0.04	0.18	0.12	0.16

T. cruzi-negative_3243	0.11	0.14	0.17	0.19
T. cruzi-negative_3244	0.18	0.36	0.21	0.29
T. cruzi-negative_3245	0.01	0.19	0.16	0.16
T. cruzi-negative_3246	0.22	0.35	0.25	0.22
T. cruzi-negative_3247	0.09	0.30	0.20	0.19
T. cruzi-negative_3248	0.12	0.25	0.15	0.24
T. cruzi-negative_3249	0.18	0.22	0.21	0.34
T. cruzi-negative_3250	0.02	0.15	0.12	0.16
T. cruzi-negative_3251	0.08	0.11	0.42	0.20
T. cruzi-negative_3252	0.07	0.17	0.13	0.20
T. cruzi-negative_3253	0.07	0.13	0.13	0.14
T. cruzi-negative_3254	0.17	0.27	0.33	0.31
T. cruzi-negative_3255	0.13	0.17	0.16	0.13
T. cruzi-negative_3256	0.17	0.29	0.18	0.27
T. cruzi-negative_3257	0.19	0.29	0.23	0.30
T. cruzi-negative_3258	0.05	0.21	0.13	0.19
T. cruzi-negative_3259	0.08	0.11	0.12	0.18
T. cruzi-negative_3260	0.15	0.34	0.18	0.30
T. cruzi-negative_3261	0.08	0.25	0.34	0.26
T. cruzi-negative_3262	0.27	0.50	0.21	0.27
T. cruzi-negative_3263	0.04	0.24	0.13	0.19
T. cruzi-negative_3264	0.12	0.17	0.14	0.18
T. cruzi-negative_3265	0.18	0.19	0.11	0.19
T. cruzi-negative_3266	0.06	0.22	0.16	0.25
T. cruzi-negative_3267	0.31	0.29	0.20	0.28
T. cruzi-negative_3268	0.01	0.24	0.12	0.18
T. cruzi-negative_3269	0.34	0.51	0.35	0.41
T. cruzi-negative_3270	0.16	0.47	0.21	0.26
T. cruzi-negative_3271	0.11	0.22	0.14	0.15
T. cruzi-negative_3272	0.05	0.14	0.10	0.19
T. cruzi-negative_3273	0.08	0.28	0.34	0.16
T. cruzi-negative_3274	0.11	0.27	0.13	0.22
T. cruzi-negative_3275	0.32	0.30	0.18	0.28
T. cruzi-negative_3276	0.02	0.24	0.10	0.16
T. cruzi-negative_3277	0.13	0.27	0.27	0.19
T. cruzi-negative_3278	0.08	0.34	0.22	0.23
T. cruzi-negative_3279	0.11	0.19	0.18	0.19
T. cruzi-negative_3280	0.21	0.26	0.16	0.23
T. cruzi-negative_3281	0.22	0.30	0.38	0.26
T. cruzi-negative_3282	0.09	0.12	0.10	0.15
T. cruzi-negative_3283	0.22	0.19	0.14	0.20
T. cruzi-negative_3284	0.03	0.13	0.07	0.13

*T. cruz*i-negative_3285	0.08	0.16	0.24	0.17
*T. cruz*i-negative_3286	0.02	0.13	0.09	0.16
*T. cruz*i-negative_3287	0.08	0.18	0.14	0.14
*T. cruz*i-negative_3288	0.15	0.23	0.12	0.20
*T. cruz*i-negative_3289	0.16	0.25	0.18	0.25
*T. cruz*i-negative_3290	0.04	0.13	0.07	0.12
*T. cruz*i-negative_3291	0.14	0.16	0.14	0.23
*T. cruz*i-negative_3292	0.06	0.18	0.12	0.18
*T. cruz*i-negative_3293	0.12	0.27	0.20	0.18
*T. cruz*i-negative_3294	0.01	0.15	0.09	0.11
*T. cruz*i-negative_3295	0.12	0.22	0.14	0.16
*T. cruz*i-negative_3296	0.15	0.16	0.10	0.19
*T. cruz*i-negative_3297	0.07	0.18	0.12	0.15
*T. cruz*i-negative_3298	0.22	0.41	0.17	0.24
*T. cruz*i-negative_3299	0.07	0.09	0.07	0.13
*T. cruz*i-negative_3300	0.24	0.51	0.23	0.29
*T. cruz*i-negative_3301	0.19	0.36	0.33	0.27
*T. cruz*i-negative_3302	0.45	0.75	0.45	0.52
*T. cruz*i-negative_3303	0.06	0.41	0.38	0.30
*T. cruz*i-negative_3304	0.02	0.23	0.12	0.19
*T. cruz*i-negative_3305	0.39	0.36	0.28	0.39
*T. cruz*i-negative_3306	0.11	0.13	0.11	0.16
*T. cruz*i-negative_3307	0.06	0.15	0.08	0.12
*T. cruz*i-negative_3308	0.09	0.16	0.11	0.11
*T. cruz*i-negative_3309	0.08	0.18	0.20	0.15
*T. cruz*i-negative_3310	0.13	0.27	0.26	0.24
*T. cruz*i-negative_3311	0.04	0.18	0.12	0.13
*T. cruz*i-negative_3312	0.06	0.10	0.21	0.15
*T. cruz*i-negative_3313	0.45	0.32	0.15	0.28
*T. cruz*i-negative_3314	0.12	0.17	0.08	0.16
*T. cruz*i-negative_3315	0.21	0.22	0.16	0.27
*T. cruz*i-negative_3316	0.03	0.24	0.12	0.19
*T. cruz*i-negative_3317	0.08	0.28	0.11	0.16
*T. cruz*i-negative_3318	0.04	0.17	0.13	0.15
*T. cruz*i-negative_3319	0.17	0.22	0.11	0.15
*T. cruz*i-negative_3320	0.29	0.19	0.14	0.14
*T. cruz*i-negative_3321	0.14	0.22	0.12	0.13
*T. cruz*i-negative_3322	0.21	0.19	0.10	0.17
*T. cruz*i-negative_3323	0.22	0.15	0.12	0.13
*T. cruz*i-negative_3324	0.13	0.18	0.18	0.15
*T. cruz*i-negative_3325	0.14	0.14	0.10	0.11
*T. cruz*i-negative_3326	0.07	0.10	0.25	0.07
*T. cruz*i-negative_3327	0.11	0.20	0.06	0.09

*T. cruz*i-negative_3328	0.07	0.12	0.04	0.05
*T. cruz*i-negative_3329	0.17	0.22	0.12	0.14
*T. cruz*i-negative_3330	0.14	0.15	0.07	0.11
*T. cruz*i-negative_3331	0.15	0.18	0.10	0.15
*T. cruz*i-negative_3332	0.15	0.59	0.39	0.16
*T. cruz*i-negative_3333	0.12	0.10	0.18	0.10
*T. cruz*i-negative_3334	0.08	0.13	0.12	0.09
*T. cruz*i-negative_3335	0.15	0.27	0.24	0.13
*T. cruz*i-negative_3336	0.11	0.16	0.11	0.10
*T. cruz*i-negative_3337	0.11	0.20	0.15	0.15
*T. cruz*i-negative_3338	0.12	0.22	0.13	0.11
*T. cruz*i-negative_3339	0.30	0.28	0.14	0.21
*T. cruz*i-negative_3340	0.16	0.17	0.19	0.12
*T. cruz*i-negative_3341	0.08	0.12	0.10	0.09
*T. cruz*i-negative_3342	0.10	0.18	0.08	0.11
*T. cruz*i-negative_3343	0.12	0.14	0.09	0.10
*T. cruz*i-negative_3344	0.12	0.16	0.11	0.09
*T. cruz*i-negative_3345	0.11	0.17	0.11	0.10
*T. cruz*i-negative_3346	0.12	0.16	0.10	0.15
*T. cruz*i-negative_3347	0.19	0.16	0.08	0.11
*T. cruz*i-negative_3348	0.15	0.16	0.18	0.24
*T. cruz*i-negative_3349	0.04	0.06	0.04	0.07
*T. cruz*i-negative_3350	0.09	0.17	0.10	0.12
*T. cruz*i-negative_3351	0.08	0.12	0.09	0.07
*T. cruz*i-negative_3352	0.07	0.30	0.06	0.07
*T. cruz*i-negative_3353	0.24	0.30	0.12	0.13
*T. cruz*i-negative_3354	0.15	0.24	0.08	0.17
*T. cruz*i-negative_3355	0.16	0.17	0.43	0.14
*T. cruz*i-negative_3356	0.19	0.29	0.34	0.22
*T. cruz*i-negative_3357	0.12	0.22	0.28	0.13
*T. cruz*i-negative_3358	0.07	0.14	0.19	0.09
*T. cruz*i-negative_3359	0.06	0.15	0.06	0.07
*T. cruz*i-negative_3360	0.12	0.16	0.10	0.11
*T. cruz*i-negative_3361	0.08	0.12	0.07	0.08
*T. cruz*i-negative_3362	0.12	0.14	0.08	0.12
*T. cruz*i-negative_3363	0.15	0.16	0.10	0.13
*T. cruz*i-negative_3364	0.11	0.24	0.08	0.08
*T. cruz*i-negative_3365	0.12	0.11	0.09	0.08
*T. cruz*i-negative_3366	0.10	0.14	0.16	0.11
*T. cruz*i-negative_3367	0.08	0.12	0.14	0.09
*T. cruz*i-negative_3368	0.23	0.30	0.12	0.17
*T. cruz*i-negative_3369	0.08	0.09	0.17	0.17

T. cruzi-negative_3370	0.08	0.14	0.09	0.09
T. cruzi-negative_3371	0.20	0.18	0.14	0.16
T. cruzi-negative_3372	0.10	0.14	0.07	0.09
T. cruzi-negative_3373	0.12	0.11	0.14	0.12
T. cruzi-negative_3374	0.25	0.32	0.36	0.32
T. cruzi-negative_3375	0.08	0.12	0.06	0.07
T. cruzi-negative_3376	0.07	0.11	0.08	0.05
T. cruzi-negative_3377	0.13	0.16	0.14	0.09
T. cruzi-negative_3378	0.15	0.21	0.08	0.13
T. cruzi-negative_3379	0.18	0.18	0.15	0.11
T. cruzi-negative_3380	0.09	0.18	0.11	0.13
T. cruzi-negative_3381	0.07	0.37	0.13	0.08
T. cruzi-negative_3382	0.10	0.18	0.10	0.09
T. cruzi-negative_3383	0.07	0.11	0.06	0.08
T. cruzi-negative_3384	0.13	0.21	0.21	0.10
T. cruzi-negative_3385	0.17	0.27	0.14	0.13
T. cruzi-negative_3386	0.16	0.22	0.13	0.17
T. cruzi-negative_3387	0.16	0.18	0.16	0.18
T. cruzi-negative_3388	0.09	0.16	0.10	0.12
T. cruzi-negative_3389	0.35	0.20	0.38	0.20
T. cruzi-negative_3390	0.04	0.11	0.06	0.05
T. cruzi-negative_3391	0.09	0.10	0.08	0.07
T. cruzi-negative_3392	0.20	0.24	0.14	0.17
T. cruzi-negative_3393	0.12	0.14	0.13	0.13
T. cruzi-negative_3394	0.09	0.10	0.14	0.30
T. cruzi-negative_3395	0.14	0.19	0.13	0.12
T. cruzi-negative_3396	0.13	0.21	0.11	0.12
T. cruzi-negative_3397	0.13	0.15	0.37	0.11
T. cruzi-negative_3398	0.10	0.16	0.11	0.08
T. cruzi-negative_3399	0.11	0.14	0.29	0.25
T. cruzi-negative_3400	0.08	0.22	0.08	0.13
T. cruzi-negative_3401	0.17	0.18	0.14	0.14
T. cruzi-negative_3402	0.11	0.10	0.07	0.09
T. cruzi-negative_3403	0.10	0.28	0.16	0.21
T. cruzi-negative_3404	0.10	0.35	0.16	0.17
T. cruzi-negative_3405	0.04	0.27	0.20	0.14
T. cruzi-negative_3406	0.15	0.28	0.16	0.21
T. cruzi-negative_3407	0.05	0.35	0.14	0.15
T. cruzi-negative_3408	0.08	0.19	0.16	0.21
T. cruzi-negative_3409	0.08	0.22	0.14	0.15
T. cruzi-negative_3410	0.04	0.20	0.19	0.11
T. cruzi-negative_3411	0.05	0.34	0.12	0.16
T. cruzi-negative_3412	0.06	0.19	0.09	0.10

T. cruzi-negative_3413	0.12	0.39	0.18	0.22
T. cruzi-negative_3414	0.07	0.34	0.17	0.14
T. cruzi-negative_3415	0.10	0.37	0.13	0.18
T. cruzi-negative_3416	0.07	0.11	0.31	0.17
T. cruzi-negative_3417	0.02	0.09	0.15	0.14
T. cruzi-negative_3418	0.04	0.19	0.11	0.12
T. cruzi-negative_3419	0.18	0.38	0.24	0.19
T. cruzi-negative_3420	0.02	0.28	0.11	0.13
T. cruzi-negative_3421	0.06	0.28	0.17	0.20
T. cruzi-negative_3422	0.08	0.35	0.16	0.14
T. cruzi-negative_3423	0.21	0.54	0.21	0.27
T. cruzi-negative_3424	0.05	0.18	0.22	0.22
T. cruzi-negative_3425	0.01	0.09	0.13	0.16
T. cruzi-negative_3426	0.11	0.19	0.15	0.17
T. cruzi-negative_3427	0.05	0.18	0.12	0.15
T. cruzi-negative_3428	0.07	0.18	0.15	0.15
T. cruzi-negative_3429	0.05	0.34	0.12	0.15
T. cruzi-negative_3430	0.08	0.64	0.15	0.18
T. cruzi-negative_3431	0.05	0.42	0.14	0.14
T. cruzi-negative_3432	0.12	0.17	0.24	0.23
T. cruzi-negative_3433	0.09	0.08	0.09	0.10
T. cruzi-negative_3434	0.12	0.16	0.13	0.18
T. cruzi-negative_3435	0.05	0.13	0.13	0.15
T. cruzi-negative_3436	0.01	0.09	0.07	0.12
T. cruzi-negative_3437	0.15	0.29	0.17	0.20
T. cruzi-negative_3438	0.07	0.23	0.14	0.16
T. cruzi-negative_3439	0.12	0.33	0.32	0.22
T. cruzi-negative_3440	0.08	0.15	0.20	0.32
T. cruzi-negative_3441	0.08	0.17	0.19	0.16
T. cruzi-negative_3442	0.04	0.14	0.16	0.15
T. cruzi-negative_3443	0.01	0.11	0.07	0.10
T. cruzi-negative_3444	0.09	0.17	0.13	0.16
T. cruzi-negative_3445	0.02	0.13	0.09	0.11
T. cruzi-negative_3446	0.08	0.24	0.10	0.14
T. cruzi-negative_3447	0.15	0.35	0.24	0.16
T. cruzi-negative_3448	0.01	0.05	0.11	0.10
T. cruzi-negative_3449	0.04	0.06	0.11	0.15
T. cruzi-negative_3450	0.08	0.10	0.16	0.19
T. cruzi-negative_3451	0.05	0.16	0.12	0.17
T. cruzi-negative_3452	0.10	0.20	0.12	0.20
T. cruzi-negative_3453	0.03	0.13	0.12	0.09
T. cruzi-negative_3454	0.08	0.26	0.12	0.16

*T. cruz*i-negative_3455	0.12	0.23	0.12	0.20
*T. cruz*i-negative_3456	0.06	0.47	0.14	0.14
*T. cruz*i-negative_3457	0.04	0.08	0.11	0.13
*T. cruz*i-negative_3458	0.16	0.21	0.26	0.39
*T. cruz*i-negative_3459	0.01	0.15	0.10	0.14
*T. cruz*i-negative_3460	0.03	0.15	0.11	0.13
*T. cruz*i-negative_3461	0.12	0.25	0.18	0.17
*T. cruz*i-negative_3462	0.10	0.37	0.22	0.23
*T. cruz*i-negative_3463	0.10	0.24	0.17	0.19
*T. cruz*i-negative_3464	0.07	0.60	0.15	0.21
*T. cruz*i-negative_3465	0.02	0.08	0.11	0.14
*T. cruz*i-negative_3466	0.10	0.13	0.14	0.16
*T. cruz*i-negative_3467	0.01	0.11	0.10	0.14
*T. cruz*i-negative_3468	0.11	0.21	0.15	0.17
*T. cruz*i-negative_3469	0.09	0.16	0.17	0.19
*T. cruz*i-negative_3470	0.03	0.24	0.15	0.18
*T. cruz*i-negative_3471	0.23	0.25	0.19	0.20
*T. cruz*i-negative_3472	0.01	0.61	0.11	0.17
*T. cruz*i-negative_3473	0.09	0.76	0.21	0.27
*T. cruz*i-negative_3474	0.02	0.06	0.07	0.07
*T. cruz*i-negative_3475	0.04	0.11	0.08	0.20
*T. cruz*i-negative_3476	0.16	0.20	0.20	0.27
*T. cruz*i-negative_3477	0.02	0.23	0.12	0.19
*T. cruz*i-negative_3478	0.05	0.16	0.17	0.27
*T. cruz*i-negative_3479	0.11	0.16	0.13	0.14
*T. cruz*i-negative_3480	0.10	0.08	0.19	0.20
*T. cruz*i-negative_3481	0.04	0.12	0.15	0.17
*T. cruz*i-negative_3482	0.08	0.10	0.12	0.15
*T. cruz*i-negative_3483	0.07	0.14	0.21	0.25
*T. cruz*i-negative_3484	0.05	0.13	0.11	0.16
*T. cruz*i-negative_3485	0.10	0.20	0.22	0.27
*T. cruz*i-negative_3486	0.06	0.13	0.12	0.11
*T. cruz*i-negative_3487	0.14	0.23	0.32	0.17
*T. cruz*i-negative_3488	0.10	0.24	0.11	0.19
*T. cruz*i-negative_3489	0.11	0.28	0.11	0.20
*T. cruz*i-negative_3490	0.15	0.02	0.17	0.27
*T. cruz*i-negative_3491	0.07	0.30	0.15	0.27
*T. cruz*i-negative_3492	0.04	0.23	0.12	0.17
*T. cruz*i-negative_3493	0.13	0.23	0.14	0.15
*T. cruz*i-negative_3494	0.09	0.21	0.16	0.20
*T. cruz*i-negative_3495	0.20	0.35	0.15	0.25
*T. cruz*i-negative_3496	0.02	0.23	0.12	0.18
*T. cruz*i-negative_3497	0.25	0.33	0.19	0.27

*T. cruz*i-negative_3498	0.11	0.03	0.16	0.22
*T. cruz*i-negative_3499	0.04	0.17	0.14	0.17
*T. cruz*i-negative_3500	0.15	0.27	0.44	0.51
*T. cruz*i-negative_3501	0.03	0.22	0.13	0.19
*T. cruz*i-negative_3502	0.17	0.34	0.17	0.26
*T. cruz*i-negative_3503	0.06	0.24	0.12	0.19
*T. cruz*i-negative_3504	0.10	0.18	0.16	0.24
*T. cruz*i-negative_3505	0.03	0.18	0.08	0.12
*T. cruz*i-negative_3506	0.14	0.03	0.11	0.19
*T. cruz*i-negative_3507	0.18	0.32	0.24	0.32
*T. cruz*i-negative_3508	0.06	0.25	0.11	0.20
*T. cruz*i-negative_3509	0.06	0.27	0.16	0.22
*T. cruz*i-negative_3510	0.01	0.12	0.04	0.10
*T. cruz*i-negative_3511	0.16	0.27	0.13	0.23
*T. cruz*i-negative_3512	0.07	0.24	0.18	0.24
*T. cruz*i-negative_3513	0.08	0.18	0.10	0.17
*T. cruz*i-negative_3514	0.16	0.02	0.14	0.35
*T. cruz*i-negative_3515	0.01	0.14	0.20	0.13
*T. cruz*i-negative_3516	0.14	0.37	0.15	0.21
*T. cruz*i-negative_3517	0.15	0.30	0.17	0.29
*T. cruz*i-negative_3518	0.20	0.41	0.25	0.42
*T. cruz*i-negative_3519	0.13	0.35	0.18	0.29
*T. cruz*i-negative_3520	0.59	0.02	1.83	0.32
*T. cruz*i-negative_3521	0.24	0.18	0.10	0.17
*T. cruz*i-negative_3522	0.18	0.33	0.15	0.36
*T. cruz*i-negative_3523	0.01	0.20	0.10	0.17
*T. cruz*i-negative_3524	0.07	0.22	0.09	0.18
*T. cruz*i-negative_3525	0.07	0.20	0.17	0.37
*T. cruz*i-negative_3526	0.09	0.30	0.09	0.21
*T. cruz*i-negative_3527	0.13	0.28	0.15	0.19
*T. cruz*i-negative_3528	0.06	0.02	0.11	0.17
*T. cruz*i-negative_3529	0.13	0.25	0.13	0.19
*T. cruz*i-negative_3530	0.09	0.20	0.10	0.18
*T. cruz*i-negative_3531	0.12	0.33	0.18	0.27
*T. cruz*i-negative_3532	0.06	0.24	0.14	0.20
*T. cruz*i-negative_3533	0.07	0.23	0.10	0.20
*T. cruz*i-negative_3534	0.11	0.24	0.13	0.22
*T. cruz*i-negative_3535	0.09	0.17	0.13	0.15
*T. cruz*i-negative_3536	0.17	0.07	0.19	0.23
*T. cruz*i-negative_3537	0.02	0.17	0.12	0.15
*T. cruz*i-negative_3538	0.15	0.31	0.28	0.39
*T. cruz*i-negative_3539	0.01	0.16	0.09	0.14

*T. cruz*i-negative_3540	0.16	0.30	0.15	0.22
*T. cruz*i-negative_3541	0.09	0.31	0.15	0.23
*T. cruz*i-negative_3542	0.06	0.23	0.10	0.17
*T. cruz*i-negative_3543	0.10	0.20	0.11	0.17
*T. cruz*i-negative_3544	0.05	0.03	0.09	0.17
*T. cruz*i-negative_3545	0.12	0.22	0.15	0.24
*T. cruz*i-negative_3546	0.02	0.22	0.08	0.16
*T. cruz*i-negative_3547	0.09	0.23	0.12	0.19
*T. cruz*i-negative_3548	0.09	0.23	0.09	0.18
*T. cruz*i-negative_3549	0.06	0.21	0.08	0.17
*T. cruz*i-negative_3550	0.17	0.41	0.22	0.30
*T. cruz*i-negative_3551	0.02	0.19	0.10	0.15
*T. cruz*i-negative_3552	0.37	0.04	0.27	0.36
*T. cruz*i-negative_3553	0.09	0.35	0.23	0.24
*T. cruz*i-negative_3554	0.06	0.28	0.15	0.31
*T. cruz*i-negative_3555	0.12	0.30	0.13	0.24
*T. cruz*i-negative_3556	0.14	0.29	0.12	0.23
*T. cruz*i-negative_3557	0.15	0.37	0.18	0.33
*T. cruz*i-negative_3558	0.10	0.40	0.24	0.30
*T. cruz*i-negative_3559	0.07	0.25	0.11	0.20
*T. cruz*i-negative_3560	0.08	0.05	0.10	0.19
*T. cruz*i-negative_3561	0.17	0.36	0.18	0.25
*T. cruz*i-negative_3562	0.06	0.27	0.12	0.20
*T. cruz*i-negative_3563	0.09	0.29	0.11	0.23
*T. cruz*i-negative_3564	0.25	0.45	0.20	0.33
*T. cruz*i-negative_3565	0.05	0.27	0.14	0.23
*T. cruz*i-negative_3566	0.12	0.37	0.14	0.24
*T. cruz*i-negative_3567	0.12	0.27	0.14	0.21
*T. cruz*i-negative_3568	0.14	0.06	0.22	0.26
*T. cruz*i-negative_3569	0.28	0.35	0.21	0.11
*T. cruz*i-negative_3570	0.05	0.25	0.17	0.09
*T. cruz*i-negative_3571	0.11	0.23	0.13	0.08
*T. cruz*i-negative_3572	0.07	0.18	0.10	0.06
*T. cruz*i-negative_3573	0.13	0.29	0.14	0.08
*T. cruz*i-negative_3574	0.04	0.20	0.16	0.09
*T. cruz*i-negative_3575	0.16	0.34	0.20	0.15
*T. cruz*i-negative_3576	0.08	0.27	0.17	0.09
*T. cruz*i-negative_3577	0.04	0.19	0.14	0.05
*T. cruz*i-negative_3578	0.18	0.26	0.20	0.13
*T. cruz*i-negative_3579	0.07	0.20	0.23	0.07
*T. cruz*i-negative_3580	0.25	0.36	0.32	0.16
*T. cruz*i-negative_3581	0.06	0.27	0.16	0.08
*T. cruz*i-negative_3582	0.19	0.38	0.25	0.15

T. cruzi-negative_3583	0.02	0.24	0.17	0.08
T. cruzi-negative_3584	0.10	0.25	0.13	0.07
T. cruzi-negative_3585	0.16	0.29	0.16	0.09
T. cruzi-negative_3586	0.06	0.22	0.13	0.05
T. cruzi-negative_3587	0.13	0.24	0.17	0.10
T. cruzi-negative_3588	0.04	0.17	0.10	0.07
T. cruzi-negative_3589	0.13	0.20	0.10	0.04
T. cruzi-negative_3590	0.10	0.26	0.21	0.14
T. cruzi-negative_3591	0.09	0.28	0.19	0.10
T. cruzi-negative_3592	0.06	0.22	0.11	0.06
T. cruzi-negative_3593	0.10	0.24	0.14	0.06
T. cruzi-negative_3594	0.03	0.15	0.07	0.02
T. cruzi-negative_3595	0.12	0.20	0.11	0.05
T. cruzi-negative_3596	0.10	0.23	0.13	0.09
T. cruzi-negative_3597	0.16	0.28	0.23	0.08
T. cruzi-negative_3598	0.01	0.18	0.09	0.06
T. cruzi-negative_3599	0.13	0.28	0.14	0.09
T. cruzi-negative_3600	0.12	0.29	0.13	0.10
T. cruzi-negative_3601	0.07	0.25	0.13	0.05
T. cruzi-negative_3602	0.04	0.15	0.11	0.06
T. cruzi-negative_3603	0.08	0.16	0.11	0.05
T. cruzi-negative_3604	0.14	0.25	0.11	0.10
T. cruzi-negative_3605	0.06	0.16	0.08	0.02
T. cruzi-negative_3606	0.08	0.24	0.14	0.05
T. cruzi-negative_3607	0.04	0.15	0.08	0.03
T. cruzi-negative_3608	0.08	0.20	0.09	0.03
T. cruzi-negative_3609	0.12	0.19	0.17	0.08
T. cruzi-negative_3610	0.04	0.17	0.10	0.05
T. cruzi-negative_3611	0.22	0.24	0.17	0.11
T. cruzi-negative_3612	0.01	0.17	0.10	0.06
T. cruzi-negative_3613	0.08	0.16	0.08	0.03
T. cruzi-negative_3614	0.10	0.22	0.11	0.06
T. cruzi-negative_3615	0.15	0.27	0.15	0.16
T. cruzi-negative_3616	0.08	0.26	0.13	0.08
T. cruzi-negative_3617	0.12	0.24	0.15	0.09
T. cruzi-negative_3618	0.09	0.35	0.25	0.13
T. cruzi-negative_3619	0.08	0.17	0.09	0.04
T. cruzi-negative_3620	0.36	0.42	0.23	0.23
T. cruzi-negative_3621	0.14	0.38	0.17	0.12
T. cruzi-negative_3622	0.07	0.14	0.07	0.03
T. cruzi-negative_3623	0.13	0.24	0.14	0.06
T. cruzi-negative_3624	0.17	0.39	0.20	0.11

*T. cruz*i-negative_3625	0.08	0.20	0.11	0.05
*T. cruz*i-negative_3626	0.10	0.24	0.18	0.07
*T. cruz*i-negative_3627	0.31	0.37	0.17	0.13
*T. cruz*i-negative_3628	0.05	0.23	0.10	0.10
*T. cruz*i-negative_3629	0.15	0.31	0.11	0.09
*T. cruz*i-negative_3630	0.07	0.21	0.11	0.06
*T. cruz*i-negative_3631	0.03	0.15	0.07	0.03
*T. cruz*i-negative_3632	0.23	0.33	0.19	0.14
*T. cruz*i-negative_3633	0.04	0.21	0.18	0.07
*T. cruz*i-negative_3634	0.06	0.19	0.11	0.08
*T. cruz*i-negative_3635	0.22	0.31	0.14	0.11
*T. cruz*i-negative_3636	0.03	0.19	0.10	0.07
*T. cruz*i-negative_3637	0.10	0.21	0.10	0.04
*T. cruz*i-negative_3638	0.14	0.35	0.19	0.11
*T. cruz*i-negative_3639	0.01	0.16	0.10	0.02
*T. cruz*i-negative_3640	0.27	0.43	0.22	0.15
*T. cruz*i-negative_3641	0.18	0.37	0.41	0.14
*T. cruz*i-negative_3642	0.02	0.25	0.13	0.09
*T. cruz*i-negative_3643	0.10	0.24	0.10	0.05
*T. cruz*i-negative_3644	0.04	0.17	0.10	0.06
*T. cruz*i-negative_3645	0.22	0.34	0.16	0.11
*T. cruz*i-negative_3646	0.11	0.33	0.22	0.13
*T. cruz*i-negative_3647	0.15	0.33	0.15	0.12
*T. cruz*i-negative_3648	0.44	0.35	0.14	0.11
*T. cruz*i-negative_3649	0.14	0.24	0.09	0.04
*T. cruz*i-negative_3650	0.20	0.28	0.17	0.08
*T. cruz*i-negative_3651	0.22	0.41	0.20	0.12
*T. cruz*i-negative_3652	0.04	0.22	0.12	0.08
*T. cruz*i-negative_3653	0.19	0.23	0.13	0.17
*T. cruz*i-negative_3654	0.15	0.48	0.19	0.20
*T. cruz*i-negative_3655	0.21	0.31	0.17	0.26
*T. cruz*i-negative_3656	0.23	0.31	0.22	0.29
*T. cruz*i-negative_3657	0.01	0.18	0.26	0.16
*T. cruz*i-negative_3658	0.07	0.64	0.15	0.16
*T. cruz*i-negative_3659	0.20	0.41	0.20	0.14
*T. cruz*i-negative_3660	0.08	0.24	0.12	0.14
*T. cruz*i-negative_3661	0.15	0.26	0.16	0.19
*T. cruz*i-negative_3662	0.04	0.57	0.16	0.15
*T. cruz*i-negative_3663	0.05	0.21	0.12	0.14
*T. cruz*i-negative_3664	0.20	0.18	0.16	0.16
*T. cruz*i-negative_3665	0.15	0.22	0.17	0.20
*T. cruz*i-negative_3666	0.01	0.70	0.14	0.11
*T. cruz*i-negative_3667	0.04	0.67	0.09	0.11

T. cruzi-negative_3668	0.19	0.64	0.17	0.18
T. cruzi-negative_3669	0.07	0.47	0.41	0.12
T. cruzi-negative_3670	0.02	0.31	0.08	0.10
T. cruzi-negative_3671	0.24	0.31	0.34	0.27
T. cruzi-negative_3672	0.01	0.14	0.13	0.14
T. cruzi-negative_3673	0.17	0.15	0.17	0.17
T. cruzi-negative_3674	0.07	0.56	0.15	0.16
T. cruzi-negative_3675	0.18	0.47	0.21	0.21
T. cruzi-negative_3676	0.07	0.43	0.17	0.20
T. cruzi-negative_3677	0.10	0.28	0.42	0.18
T. cruzi-negative_3678	0.13	0.25	0.19	0.22
T. cruzi-negative_3679	0.05	0.16	0.16	0.18
T. cruzi-negative_3680	0.08	0.10	0.45	0.18
T. cruzi-negative_3681	0.18	0.16	0.33	0.20
T. cruzi-negative_3682	0.06	0.54	0.31	0.16
T. cruzi-negative_3683	0.09	0.30	0.17	0.21
T. cruzi-negative_3684	0.20	0.36	0.32	0.27
T. cruzi-negative_3685	0.17	0.32	0.22	0.29
T. cruzi-negative_3686	0.01	0.10	0.10	0.14
T. cruzi-negative_3687	0.19	0.28	0.37	0.26
T. cruzi-negative_3688	0.11	0.25	0.16	0.27
T. cruzi-negative_3689	0.12	0.19	0.14	0.20
T. cruzi-negative_3690	0.08	0.32	0.17	0.20
T. cruzi-negative_3691	0.08	0.19	0.16	0.17
T. cruzi-negative_3692	0.09	0.16	0.22	0.16
T. cruzi-negative_3693	0.15	0.16	0.17	0.16
T. cruzi-negative_3694	0.08	0.16	0.16	0.19
T. cruzi-negative_3695	0.17	0.22	0.13	0.28
T. cruzi-negative_3696	0.05	0.11	0.12	0.14
T. cruzi-negative_3697	0.09	0.15	0.15	0.16
T. cruzi-negative_3698	0.10	0.26	0.15	0.22
T. cruzi-negative_3699	0.18	0.21	0.26	0.30
T. cruzi-negative_3700	0.05	0.18	0.15	0.19
T. cruzi-negative_3701	0.03	0.09	0.11	0.12
T. cruzi-negative_3702	0.07	0.09	0.12	0.16
T. cruzi-negative_3703	0.08	0.13	0.12	0.18
T. cruzi-negative_3704	0.05	0.15	0.09	0.20
T. cruzi-negative_3705	0.05	0.12	0.10	0.12
T. cruzi-negative_3706	0.01	0.17	0.08	0.09
T. cruzi-negative_3707	0.11	0.17	0.15	0.18
T. cruzi-negative_3708	0.17	0.24	0.20	0.20
T. cruzi-negative_3709	0.01	0.10	0.08	0.11

*T. cruz*i-negative_3710	0.20	0.22	0.19	0.23
*T. cruz*i-negative_3711	0.11	0.22	0.23	0.23
*T. cruz*i-negative_3712	0.05	0.14	0.15	0.17
*T. cruz*i-negative_3713	0.16	0.23	0.18	0.22
*T. cruz*i-negative_3714	0.16	0.40	0.32	0.26
*T. cruz*i-negative_3715	0.05	0.08	0.09	0.09
*T. cruz*i-negative_3716	0.06	0.08	0.08	0.11
*T. cruz*i-negative_3717	0.08	0.09	0.10	0.13
*T. cruz*i-negative_3718	0.16	0.20	0.22	0.21
*T. cruz*i-negative_3719	0.04	0.12	0.12	0.14
*T. cruz*i-negative_3720	0.08	0.16	0.09	0.15
*T. cruz*i-negative_3721	0.12	0.17	0.15	0.17
*T. cruz*i-negative_3722	0.07	0.20	0.29	0.19
*T. cruz*i-negative_3723	0.02	0.11	0.11	0.11
*T. cruz*i-negative_3724	0.10	0.17	0.18	0.15
*T. cruz*i-negative_3725	0.01	0.07	0.09	0.10
*T. cruz*i-negative_3726	0.08	0.11	0.11	0.13
*T. cruz*i-negative_3727	0.11	0.15	0.10	0.17
*T. cruz*i-negative_3728	0.02	0.08	0.07	0.11
*T. cruz*i-negative_3729	0.08	0.13	0.14	0.15
*T. cruz*i-negative_3730	0.05	0.15	0.15	0.12
*T. cruz*i-negative_3731	0.02	0.10	0.11	0.11
*T. cruz*i-negative_3732	0.06	0.13	0.10	0.11
*T. cruz*i-negative_3733	0.10	0.13	0.11	0.16
*T. cruz*i-negative_3734	0.02	0.09	0.14	0.11
*T. cruz*i-negative_3735	0.22	0.18	0.12	0.20
*T. cruz*i-negative_3736	0.10	0.22	0.21	0.28
*T. cruz*i-negative_3737	0.07	0.18	0.07	0.12
*T. cruz*i-negative_3738	0.13	0.15	0.15	0.12
*T. cruz*i-negative_3739	0.12	0.16	0.13	0.10
*T. cruz*i-negative_3740	0.10	0.17	0.14	0.11
*T. cruz*i-negative_3741	0.09	0.11	0.12	0.07
*T. cruz*i-negative_3742	0.25	0.59	0.38	0.55
*T. cruz*i-negative_3743	0.24	0.34	0.34	0.24
*T. cruz*i-negative_3744	0.20	0.27	0.20	0.22
*T. cruz*i-negative_3745	0.32	0.40	0.46	0.35
*T. cruz*i-negative_3746	0.21	0.28	0.24	0.19
*T. cruz*i-negative_3747	0.23	0.25	0.26	0.18
*T. cruz*i-negative_3748	0.18	0.24	0.23	0.19
*T. cruz*i-negative_3749	0.12	0.18	0.16	0.11
*T. cruz*i-negative_3750	0.17	0.21	0.18	0.13
*T. cruz*i-negative_3751	0.23	0.24	0.22	0.13
*T. cruz*i-negative_3752	0.19	0.25	0.19	0.14

*T. cruz*i-negative_3753	0.45	0.65	0.44	0.65
*T. cruz*i-negative_3754	0.25	0.18	0.23	0.13
*T. cruz*i-negative_3755	0.12	0.14	0.14	0.10
*T. cruz*i-negative_3756	0.09	0.12	0.11	0.05
*T. cruz*i-negative_3757	0.11	0.14	0.14	0.11
*T. cruz*i-negative_3758	0.21	0.26	0.20	0.17
*T. cruz*i-negative_3759	0.16	0.18	0.19	0.10
*T. cruz*i-negative_3760	0.13	0.09	0.09	0.05
*T. cruz*i-negative_3761	0.14	0.16	0.13	0.07
*T. cruz*i-negative_3762	0.11	0.09	0.10	0.06
*T. cruz*i-negative_3763	0.19	0.21	0.24	0.15
*T. cruz*i-negative_3764	0.18	0.15	0.27	0.11
*T. cruz*i-negative_3765	0.15	0.15	0.23	0.13
*T. cruz*i-negative_3766	0.17	0.18	0.19	0.09
*T. cruz*i-negative_3767	0.30	0.21	0.30	0.15
*T. cruz*i-negative_3768	0.14	0.21	0.17	0.13
*T. cruz*i-negative_3769	0.38	0.16	0.18	0.10
*T. cruz*i-negative_3770	0.05	0.08	0.12	0.05
*T. cruz*i-negative_3771	0.12	0.23	0.25	0.14
*T. cruz*i-negative_3772	0.21	0.23	0.26	0.17
*T. cruz*i-negative_3773	0.11	0.11	0.17	0.06
*T. cruz*i-negative_3774	0.12	0.13	0.17	0.07
*T. cruz*i-negative_3775	0.12	0.14	0.20	0.09
*T. cruz*i-negative_3776	0.07	0.06	0.08	0.03
*T. cruz*i-negative_3777	0.10	0.18	0.26	0.10
*T. cruz*i-negative_3778	0.09	0.17	0.16	0.12
*T. cruz*i-negative_3779	0.05	0.20	0.18	0.11
*T. cruz*i-negative_3780	0.08	0.10	0.12	0.06
*T. cruz*i-negative_3781	0.20	0.29	0.30	0.22
*T. cruz*i-negative_3782	0.10	0.10	0.42	0.04
*T. cruz*i-negative_3783	0.11	0.14	0.16	0.07
*T. cruz*i-negative_3784	0.19	0.19	0.30	0.11
*T. cruz*i-negative_3785	0.16	0.26	0.23	0.14
*T. cruz*i-negative_3786	0.07	0.18	0.18	0.10
*T. cruz*i-negative_3787	0.07	0.18	0.15	0.14
*T. cruz*i-negative_3788	0.25	0.72	0.34	0.52
*T. cruz*i-negative_3789	0.15	0.14	0.15	0.08
*T. cruz*i-negative_3790	0.14	0.20	0.17	0.09
*T. cruz*i-negative_3791	0.08	0.10	0.11	0.01
*T. cruz*i-negative_3792	0.11	0.06	0.09	0.06
*T. cruz*i-negative_3793	0.13	0.17	0.21	0.13
*T. cruz*i-negative_3794	0.10	0.24	0.18	0.11

T. cruzi-negative_3795	0.05	0.16	0.16	0.06
T. cruzi-negative_3796	0.16	0.35	0.25	0.17
T. cruzi-negative_3797	0.07	0.10	0.09	0.05
T. cruzi-negative_3798	0.22	0.20	0.21	0.13
T. cruzi-negative_3799	0.18	0.21	0.34	0.24
T. cruzi-negative_3800	0.17	0.16	0.23	0.09
T. cruzi-negative_3801	0.07	0.15	0.13	0.06
T. cruzi-negative_3802	0.11	0.23	0.25	0.12
T. cruzi-negative_3803	0.18	0.38	0.28	0.19
T. cruzi-negative_3804	0.21	0.29	0.27	0.24
T. cruzi-negative_3805	0.18	0.21	0.18	0.12
T. cruzi-negative_3806	0.26	0.38	0.29	0.28
T. cruzi-negative_3807	0.15	0.16	0.27	0.10
T. cruzi-negative_3808	0.14	0.29	0.24	0.20
T. cruzi-negative_3809	0.10	0.24	0.19	0.15
T. cruzi-negative_3810	0.04	0.12	0.12	0.06
T. cruzi-negative_3811	0.11	0.22	0.20	0.12
T. cruzi-negative_3812	0.16	0.19	0.21	0.14
T. cruzi-negative_3813	0.22	0.24	0.28	0.14
T. cruzi-negative_3814	0.13	0.13	0.23	0.08
T. cruzi-negative_3815	0.13	0.17	0.35	0.14
T. cruzi-negative_3816	0.07	0.15	0.15	0.07
T. cruzi-negative_3817	0.16	0.37	0.17	0.16
T. cruzi-negative_3818	0.10	0.33	0.09	0.09
T. cruzi-negative_3819	0.12	0.23	0.07	0.10
T. cruzi-negative_3820	0.12	0.24	0.13	0.11
T. cruzi-negative_3821	0.29	0.21	0.16	0.17
T. cruzi-negative_3822	0.10	0.29	0.14	0.15
T. cruzi-negative_3823	0.12	0.28	0.19	0.12
T. cruzi-negative_3824	0.13	0.25	0.28	0.15
T. cruzi-negative_3825	0.14	0.25	0.15	0.14
T. cruzi-negative_3826	0.17	0.29	0.10	0.16
T. cruzi-negative_3827	0.09	0.19	0.18	0.07
T. cruzi-negative_3828	0.14	0.29	0.12	0.22
T. cruzi-negative_3829	0.07	0.19	0.16	0.07
T. cruzi-negative_3830	0.09	0.27	0.15	0.11
T. cruzi-negative_3831	0.06	0.14	0.11	0.06
T. cruzi-negative_3832	0.08	0.10	0.12	0.03
T. cruzi-negative_3833	0.07	0.14	0.20	0.08
T. cruzi-negative_3834	0.08	0.13	0.12	0.08
T. cruzi-negative_3835	0.13	0.24	0.11	0.15
T. cruzi-negative_3836	0.12	0.24	0.12	0.07
T. cruzi-negative_3837	0.04	0.15	0.15	0.06

T. cruzi-negative_3838	0.05	0.11	0.08	0.05
T. cruzi-negative_3839	0.16	0.31	0.13	0.15
T. cruzi-negative_3840	0.15	0.27	0.18	0.11
T. cruzi-negative_3841	0.07	0.11	0.10	0.03
T. cruzi-negative_3842	0.23	0.40	0.11	0.35
T. cruzi-negative_3843	0.12	0.25	0.12	0.09
T. cruzi-negative_3844	0.11	0.22	0.09	0.10
T. cruzi-negative_3845	0.07	0.19	0.11	0.10
T. cruzi-negative_3846	0.03	0.13	0.08	0.03
T. cruzi-negative_3847	0.12	0.26	0.18	0.13
T. cruzi-negative_3848	0.07	0.17	0.12	0.08
T. cruzi-negative_3849	0.12	0.26	0.13	0.11
T. cruzi-negative_3850	0.08	0.17	0.05	0.08
T. cruzi-negative_3851	0.09	0.20	0.10	0.18
T. cruzi-negative_3852	0.10	0.18	0.11	0.09
T. cruzi-negative_3853	0.09	0.26	0.14	0.10
T. cruzi-negative_3854	0.12	0.26	0.11	0.13
T. cruzi-negative_3855	0.09	0.22	0.11	0.07
T. cruzi-negative_3856	0.13	0.31	0.09	0.14
T. cruzi-negative_3857	0.10	0.24	0.11	0.16
T. cruzi-negative_3858	0.06	0.12	0.11	0.08
T. cruzi-negative_3859	0.11	0.20	0.11	0.09
T. cruzi-negative_3860	0.10	0.17	0.11	0.07
T. cruzi-negative_3861	0.08	0.25	0.10	0.11
T. cruzi-negative_3862	0.12	0.24	0.11	0.11
T. cruzi-negative_3863	0.07	0.22	0.12	0.11
T. cruzi-negative_3864	0.05	0.16	0.10	0.07
T. cruzi-negative_3865	0.11	0.26	0.09	0.10
T. cruzi-negative_3866	0.08	0.20	0.12	0.08
T. cruzi-negative_3867	0.10	0.20	0.07	0.14
T. cruzi-negative_3868	0.11	0.21	0.30	0.11
T. cruzi-negative_3869	0.08	0.24	0.18	0.14
T. cruzi-negative_3870	0.03	0.15	0.07	0.03
T. cruzi-negative_3871	0.01	0.08	0.10	0.02
T. cruzi-negative_3872	0.10	0.23	0.08	0.14
T. cruzi-negative_3873	0.04	0.12	0.12	0.04
T. cruzi-negative_3874	0.08	0.16	0.14	0.06
T. cruzi-negative_3875	0.07	0.13	0.12	0.09
T. cruzi-negative_3876	0.12	0.24	0.11	0.10
T. cruzi-negative_3877	0.08	0.20	0.11	0.08
T. cruzi-negative_3878	0.08	0.10	0.06	0.03
T. cruzi-negative_3879	0.05	0.13	0.25	0.05

T. cruzi-negative_3880	0.09	0.21	0.11	0.27
T. cruzi-negative_3881	0.06	0.15	0.10	0.05
T. cruzi-negative_3882	0.08	0.19	0.13	0.06
T. cruzi-negative_3883	0.04	0.08	0.16	0.01
T. cruzi-negative_3884	0.09	0.21	0.13	0.08
T. cruzi-negative_3885	0.10	0.26	0.18	0.12
T. cruzi-negative_3886	0.06	0.17	0.20	0.06
T. cruzi-negative_3887	0.12	0.30	0.09	0.14
T. cruzi-negative_3888	0.05	0.10	0.17	0.06
T. cruzi-negative_3889	0.04	0.15	0.05	0.04
T. cruzi-negative_3890	0.05	0.15	0.06	0.04
T. cruzi-negative_3891	0.12	0.16	0.08	0.09
T. cruzi-negative_3892	0.09	0.17	0.07	0.13
T. cruzi-negative_3893	0.08	0.16	0.31	0.08
T. cruzi-negative_3894	0.07	0.19	0.12	0.06
T. cruzi-negative_3895	0.16	0.24	0.15	0.08
T. cruzi-negative_3896	0.07	0.11	0.11	0.04
T. cruzi-negative_3897	0.10	0.24	0.13	0.08
T. cruzi-negative_3898	0.08	0.15	0.08	0.08
T. cruzi-negative_3899	0.06	0.13	0.08	0.06
T. cruzi-negative_3900	0.28	0.28	0.18	0.21
T. cruzi-negative_3901	0.26	0.21	0.14	0.14
T. cruzi-negative_3902	0.27	0.25	0.20	0.21
T. cruzi-negative_3903	0.27	0.27	0.15	0.19
T. cruzi-negative_3904	0.20	0.27	0.15	0.17
T. cruzi-negative_3905	0.33	0.45	0.17	0.22
T. cruzi-negative_3906	0.20	0.25	0.12	0.16
T. cruzi-negative_3907	0.13	0.27	0.09	0.08
T. cruzi-negative_3908	0.25	0.24	0.19	0.17
T. cruzi-negative_3909	0.30	0.52	0.19	0.23
T. cruzi-negative_3910	0.28	0.34	0.30	0.20
T. cruzi-negative_3911	0.16	0.13	0.12	0.13
T. cruzi-negative_3912	0.21	0.15	0.11	0.16
T. cruzi-negative_3913	0.23	0.28	0.16	0.17
T. cruzi-negative_3914	0.20	0.32	0.15	0.16
T. cruzi-negative_3915	0.21	0.35	0.24	0.20
T. cruzi-negative_3916	0.11	0.13	0.07	0.07
T. cruzi-negative_3917	0.21	0.19	0.13	0.14
T. cruzi-negative_3918	0.19	0.27	0.15	0.20
T. cruzi-negative_3919	0.11	0.27	0.11	0.11
T. cruzi-negative_3920	0.14	0.27	0.12	0.15
T. cruzi-negative_3921	0.09	0.14	0.08	0.10
T. cruzi-negative_3922	0.09	0.21	0.07	0.09

*T. cruz*i-negative_3923	0.11	0.37	0.14	0.12
*T. cruz*i-negative_3924	0.27	0.28	0.14	0.13
*T. cruz*i-negative_3925	0.14	0.21	0.16	0.15
*T. cruz*i-negative_3926	0.19	0.34	0.21	0.18
*T. cruz*i-negative_3927	0.14	0.18	0.12	0.11
*T. cruz*i-negative_3928	0.16	0.21	0.18	0.17
*T. cruz*i-negative_3929	0.20	0.27	0.14	0.16
*T. cruz*i-negative_3930	0.19	0.27	0.15	0.20
*T. cruz*i-negative_3931	0.36	0.48	0.21	0.32
*T. cruz*i-negative_3932	0.18	0.54	0.17	0.18
*T. cruz*i-negative_3933	0.14	0.19	0.11	0.12
*T. cruz*i-negative_3934	0.15	0.20	0.10	0.14
*T. cruz*i-negative_3935	0.19	0.21	0.16	0.21
*T. cruz*i-negative_3936	0.12	0.43	0.10	0.10
*T. cruz*i-negative_3937	0.14	0.17	0.08	0.10
*T. cruz*i-negative_3938	0.22	0.26	0.16	0.27
*T. cruz*i-negative_3939	0.23	0.60	0.18	0.22
*T. cruz*i-negative_3940	0.13	0.15	0.09	0.08
*T. cruz*i-negative_3941	0.16	0.59	0.13	0.12
*T. cruz*i-negative_3942	0.19	0.24	0.17	0.19
*T. cruz*i-negative_3943	0.15	0.21	0.15	0.17
*T. cruz*i-negative_3944	0.18	0.18	0.13	0.16
*T. cruz*i-negative_3945	0.20	0.19	0.09	0.08
*T. cruz*i-negative_3946	0.16	0.24	0.09	0.13
*T. cruz*i-negative_3947	0.33	0.30	0.20	0.28
*T. cruz*i-negative_3948	0.23	0.21	0.16	0.20
*T. cruz*i-negative_3949	0.13	0.45	0.10	0.13
*T. cruz*i-negative_3950	0.22	0.24	0.19	0.21
*T. cruz*i-negative_3951	0.17	0.22	0.18	0.22
*T. cruz*i-negative_3952	0.23	0.27	0.20	0.27
*T. cruz*i-negative_3953	0.22	0.22	0.19	0.20
*T. cruz*i-negative_3954	0.20	0.23	0.16	0.24
*T. cruz*i-negative_3955	0.15	0.17	0.13	0.16
*T. cruz*i-negative_3956	0.29	0.22	0.18	0.22
*T. cruz*i-negative_3957	0.21	0.22	0.14	0.15
*T. cruz*i-negative_3958	0.16	0.21	0.17	0.19
*T. cruz*i-negative_3959	0.09	0.16	0.12	0.14
*T. cruz*i-negative_3960	0.12	0.23	0.11	0.15
*T. cruz*i-negative_3961	0.15	0.41	0.16	0.16
*T. cruz*i-negative_3962	0.16	0.16	0.11	0.12
*T. cruz*i-negative_3963	0.27	0.36	0.24	0.27
*T. cruz*i-negative_3964	0.24	0.33	0.18	0.22

T. cruzi-negative_3965	0.23	0.20	0.17	0.17
T. cruzi-negative_3966	0.26	0.21	0.16	0.14
T. cruzi-negative_3967	0.14	0.39	0.13	0.14
T. cruzi-negative_3968	0.28	0.28	0.22	0.23
T. cruzi-negative_3969	0.23	0.27	0.19	0.23
T. cruzi-negative_3970	0.21	0.21	0.14	0.17
T. cruzi-negative_3971	0.22	0.26	0.12	0.13
T. cruzi-negative_3972	0.21	0.26	0.15	0.18
T. cruzi-negative_3973	0.25	0.38	0.19	0.23
T. cruzi-negative_3974	0.25	0.16	0.13	0.14
T. cruzi-negative_3975	0.52	0.10	0.27	0.22
T. cruzi-negative_3976	0.32	0.32	0.18	0.21
T. cruzi-negative_3977	0.33	0.34	0.23	0.28
T. cruzi-negative_3978	0.25	0.18	0.15	0.23
T. cruzi-negative_3979	0.16	0.19	0.10	0.14
T. cruzi-negative_3980	0.16	0.44	0.12	0.13
T. cruzi-negative_3981	0.23	0.31	0.21	0.24
T. cruzi-negative_3982	0.17	0.25	0.13	0.14
T. cruzi-negative_3983	0.29	0.11	0.13	0.16
T. cruzi-negative_3984	0.12	0.12	0.07	0.15
T. cruzi-negative_3985	0.23	0.20	0.13	0.24
T. cruzi-negative_3986	0.06	0.10	0.05	0.13
T. cruzi-negative_3987	0.06	0.15	0.12	0.13
T. cruzi-negative_3988	0.30	0.25	0.14	0.26
T. cruzi-negative_3989	0.20	0.15	0.11	0.19
T. cruzi-negative_3990	0.08	0.08	0.05	0.12
T. cruzi-negative_3991	0.21	0.16	0.11	0.21
T. cruzi-negative_3992	0.17	0.16	0.09	0.18
T. cruzi-negative_3993	0.18	0.15	0.10	0.17
T. cruzi-negative_3994	0.16	0.14	0.09	0.16
T. cruzi-negative_3995	0.21	0.18	0.13	0.22
T. cruzi-negative_3996	0.08	0.19	0.09	0.21
T. cruzi-negative_3997	0.14	0.14	0.10	0.16
T. cruzi-negative_3998	0.34	0.23	0.18	0.35
T. cruzi-negative_3999	0.11	0.09	0.12	0.17
T. cruzi-negative_4000	0.08	0.15	0.05	0.12
T. cruzi-negative_4001	0.06	0.12	0.06	0.15
T. cruzi-negative_4002	0.08	0.22	0.05	0.16
T. cruzi-negative_4003	0.10	0.16	0.09	0.17
T. cruzi-negative_4004	0.13	0.31	0.13	0.22
T. cruzi-negative_4005	0.15	0.20	0.12	0.23
T. cruzi-negative_4006	0.13	0.15	0.09	0.18
T. cruzi-negative_4007	0.12	0.12	0.08	0.18

*T. cruz*i-negative_4008	0.18	0.30	0.13	0.24
*T. cruz*i-negative_4009	0.12	0.25	0.10	0.20
*T. cruz*i-negative_4010	0.17	0.23	0.14	0.23
*T. cruz*i-negative_4011	0.08	0.14	0.05	0.15
*T. cruz*i-negative_4012	0.06	0.17	0.06	0.13
*T. cruz*i-negative_4013	0.12	0.14	0.13	0.32
*T. cruz*i-negative_4014	0.13	0.16	0.09	0.19
*T. cruz*i-negative_4015	0.07	0.25	0.07	0.16
*T. cruz*i-negative_4016	0.05	0.09	0.05	0.13
*T. cruz*i-negative_4017	0.07	0.20	0.06	0.16
*T. cruz*i-negative_4018	0.06	0.17	0.05	0.12
*T. cruz*i-negative_4019	0.05	0.11	0.05	0.12
*T. cruz*i-negative_4020	0.13	0.23	0.10	0.23
*T. cruz*i-negative_4021	0.08	0.19	0.11	0.19
*T. cruz*i-negative_4022	0.07	0.11	0.08	0.17
*T. cruz*i-negative_4023	0.03	0.27	0.07	0.12
*T. cruz*i-negative_4024	0.09	0.16	0.10	0.24
*T. cruz*i-negative_4025	0.04	0.14	0.03	0.11
*T. cruz*i-negative_4026	0.14	0.16	0.10	0.24
*T. cruz*i-negative_4027	0.04	0.28	0.07	0.16
*T. cruz*i-negative_4028	0.08	0.25	0.07	0.16
*T. cruz*i-negative_4029	0.06	0.17	0.08	0.16
*T. cruz*i-negative_4030	0.08	0.15	0.11	0.20
*T. cruz*i-negative_4031	0.14	0.27	0.15	0.28
*T. cruz*i-negative_4032	0.10	0.17	0.08	0.22
*T. cruz*i-negative_4033	0.07	0.26	0.07	0.16
*T. cruz*i-negative_4034	0.12	0.21	0.10	0.19
*T. cruz*i-negative_4035	0.07	0.23	0.09	0.18
*T. cruz*i-negative_4036	0.16	0.21	0.41	0.22
*T. cruz*i-negative_4037	0.12	0.23	0.21	0.23
*T. cruz*i-negative_4038	0.05	0.18	0.09	0.16
*T. cruz*i-negative_4039	0.01	0.12	0.06	0.11
*T. cruz*i-negative_4040	0.09	0.15	0.11	0.17
*T. cruz*i-negative_4041	0.07	0.21	0.07	0.17
*T. cruz*i-negative_4042	0.17	0.15	0.07	0.14
*T. cruz*i-negative_4043	0.05	0.14	0.07	0.13
*T. cruz*i-negative_4044	0.02	0.19	0.06	0.13
*T. cruz*i-negative_4045	0.15	0.25	0.16	0.24
*T. cruz*i-negative_4046	0.11	0.16	0.15	0.24
*T. cruz*i-negative_4047	0.01	0.05	0.11	0.10
*T. cruz*i-negative_4048	0.08	0.16	0.11	0.22
*T. cruz*i-negative_4049	0.11	0.24	0.13	0.23

*T. cruz*i-negative_4050	0.06	0.16	0.10	0.19
*T. cruz*i-negative_4051	0.07	0.20	0.08	0.16
*T. cruz*i-negative_4052	0.03	0.26	0.08	0.13
*T. cruz*i-negative_4053	0.09	0.15	0.13	0.17
*T. cruz*i-negative_4054	0.12	0.18	0.12	0.22
*T. cruz*i-negative_4055	0.12	0.20	0.14	0.26
*T. cruz*i-negative_4056	0.25	0.23	0.33	0.53
*T. cruz*i-negative_4057	0.04	0.13	0.03	0.12
*T. cruz*i-negative_4058	0.08	0.15	0.14	0.17
*T. cruz*i-negative_4059	0.03	0.30	0.07	0.12
*T. cruz*i-negative_4060	0.06	0.23	0.09	0.16
*T. cruz*i-negative_4061	0.08	0.17	0.09	0.19
*T. cruz*i-negative_4062	0.04	0.20	0.11	0.26
*T. cruz*i-negative_4063	0.03	0.12	0.07	0.13
*T. cruz*i-negative_4064	0.03	0.15	0.10	0.41
*T. cruz*i-negative_4065	0.02	0.12	0.07	0.14
*T. cruz*i-negative_4066	0.13	0.19	0.14	0.18
*T. cruz*i-negative_4067	0.05	0.18	0.07	0.14
*T. cruz*i-negative_4068	0.03	0.06	0.04	0.06
*T. cruz*i-negative_4069	0.02	0.04	0.05	0.07
*T. cruz*i-negative_4070	0.01	0.03	0.03	0.11
*T. cruz*i-negative_4071	0.08	0.14	0.08	0.10
*T. cruz*i-negative_4072	0.10	0.20	0.07	0.09
*T. cruz*i-negative_4073	0.11	0.15	0.08	0.11
*T. cruz*i-negative_4074	0.08	0.11	0.07	0.08
*T. cruz*i-negative_4075	0.15	0.18	0.13	0.14
*T. cruz*i-negative_4076	0.16	0.15	0.13	0.18
*T. cruz*i-negative_4077	0.07	0.11	0.07	0.08
*T. cruz*i-negative_4078	0.12	0.16	0.10	0.17
*T. cruz*i-negative_4079	0.11	0.09	0.08	0.10
*T. cruz*i-negative_4080	0.13	0.16	0.07	0.25
*T. cruz*i-negative_4081	0.10	0.13	0.14	0.09
*T. cruz*i-negative_4082	0.10	0.12	0.10	0.11
*T. cruz*i-negative_4083	0.03	0.05	0.06	0.06
*T. cruz*i-negative_4084	0.03	0.09	0.07	0.12
*T. cruz*i-negative_4085	0.11	0.10	0.13	0.14
*T. cruz*i-negative_4086	0.02	0.07	0.08	0.10
*T. cruz*i-negative_4087	0.08	0.30	0.08	0.14
*T. cruz*i-negative_4088	0.09	0.35	0.08	0.15
*T. cruz*i-negative_4089	0.05	0.13	0.09	0.09
*T. cruz*i-negative_4090	0.14	0.14	0.09	0.15
*T. cruz*i-negative_4091	0.12	0.17	0.11	0.16
*T. cruz*i-negative_4092	0.14	0.08	0.05	0.09

T. cruzi-negative_4093	0.03	0.05	0.06	0.09
T. cruzi-negative_4094	0.01	0.04	0.03	0.09
T. cruzi-negative_4095	0.01	0.04	0.12	0.07
T. cruzi-negative_4096	0.05	0.22	0.06	0.14
T. cruzi-negative_4097	0.04	0.09	0.06	0.11
T. cruzi-negative_4098	0.08	0.09	0.06	0.13
T. cruzi-negative_4099	0.08	0.15	0.07	0.11
T. cruzi-negative_4100	0.02	0.07	0.11	0.08
T. cruzi-negative_4101	0.07	0.09	0.08	0.12
T. cruzi-negative_4102	0.06	0.05	0.05	0.12
T. cruzi-negative_4103	0.06	0.17	0.04	0.19
T. cruzi-negative_4104	0.12	0.11	0.08	0.19
T. cruzi-negative_4105	0.01	0.07	0.07	0.08
T. cruzi-negative_4106	0.09	0.16	0.08	0.16
T. cruzi-negative_4107	0.06	0.13	0.05	0.15
T. cruzi-negative_4108	0.07	0.11	0.07	0.20
T. cruzi-negative_4109	0.14	0.11	0.12	0.15
T. cruzi-negative_4110	0.08	0.08	0.06	0.23
T. cruzi-negative_4111	0.25	0.22	0.19	0.30
T. cruzi-negative_4112	0.35	0.57	0.30	0.42
T. cruzi-negative_4113	0.13	0.19	0.12	0.11
T. cruzi-negative_4114	0.02	0.08	0.02	0.06
T. cruzi-negative_4115	0.08	0.11	0.06	0.14
T. cruzi-negative_4116	0.12	0.22	0.09	0.15
T. cruzi-negative_4117	0.07	0.07	0.06	0.10
T. cruzi-negative_4118	0.11	0.09	0.06	0.25
T. cruzi-negative_4119	0.13	0.16	0.08	0.25
T. cruzi-negative_4120	0.10	0.20	0.11	0.24
T. cruzi-negative_4121	0.11	0.19	0.12	0.13
T. cruzi-negative_4122	0.20	0.22	0.13	0.17
T. cruzi-negative_4123	0.07	0.10	0.08	0.13
T. cruzi-negative_4124	0.06	0.05	0.04	0.14
T. cruzi-negative_4125	0.07	0.05	0.06	0.08
T. cruzi-negative_4126	0.06	0.12	0.05	0.20
T. cruzi-negative_4127	0.14	0.14	0.09	0.22
T. cruzi-negative_4128	0.04	0.09	0.03	0.20
T. cruzi-negative_4129	0.07	0.10	0.07	0.09
T. cruzi-negative_4130	0.11	0.12	0.09	0.10
T. cruzi-negative_4131	0.06	0.07	0.05	0.08
T. cruzi-negative_4132	0.06	0.08	0.07	0.11
T. cruzi-negative_4133	0.12	0.10	0.07	0.13
T. cruzi-negative_4134	0.32	0.25	0.19	0.42

*T. cruz*i-negative_4135	0.17	0.08	0.06	0.17
*T. cruz*i-negative_4136	0.10	0.09	0.06	0.13
*T. cruz*i-negative_4137	0.10	0.12	0.11	0.08
*T. cruz*i-negative_4138	0.05	0.11	0.04	0.05
*T. cruz*i-negative_4139	0.11	0.08	0.06	0.07
*T. cruz*i-negative_4140	0.13	0.14	0.07	0.09
*T. cruz*i-negative_4141	0.07	0.06	0.05	0.06
*T. cruz*i-negative_4142	0.14	0.09	0.08	0.16
*T. cruz*i-negative_4143	0.21	0.08	0.06	0.11
*T. cruz*i-negative_4144	0.10	0.11	0.06	0.10
*T. cruz*i-negative_4145	0.17	0.18	0.13	0.09
*T. cruz*i-negative_4146	0.12	0.15	0.09	0.10
*T. cruz*i-negative_4147	0.09	0.09	0.04	0.04
*T. cruz*i-negative_4148	0.11	0.10	0.06	0.07
*T. cruz*i-negative_4149	0.11	0.07	0.07	0.06
*T. cruz*i-negative_4150	0.15	0.17	0.09	0.15
*T. cruz*i-negative_4151	0.37	0.08	0.17	0.08
*T. cruz*i-negative_4152	0.21	0.22	0.17	0.15
*T. cruz*i-negative_4153	0.15	0.16	0.09	0.09
*T. cruz*i-negative_4154	0.11	0.13	0.07	0.05
*T. cruz*i-negative_4155	0.10	0.14	0.13	0.05
*T. cruz*i-negative_4156	0.26	0.33	0.16	0.15
*T. cruz*i-negative_4157	0.21	0.27	0.14	0.11
*T. cruz*i-negative_4158	0.34	0.38	0.19	0.24
*T. cruz*i-negative_4159	0.14	0.19	0.28	0.14
*T. cruz*i-negative_4160	0.18	0.17	0.15	0.18
*T. cruz*i-negative_4161	0.15	0.19	0.10	0.19
*T. cruz*i-negative_4162	0.27	0.30	0.18	0.29
*T. cruz*i-negative_4163	0.19	0.21	0.12	0.19
*T. cruz*i-negative_4164	0.21	0.39	0.16	0.27
*T. cruz*i-negative_4165	0.17	0.23	0.15	0.18
*T. cruz*i-negative_4166	0.16	0.21	0.11	0.18
*T. cruz*i-negative_4167	0.20	0.36	0.12	0.15
*T. cruz*i-negative_4168	0.11	0.13	0.08	0.21
*T. cruz*i-negative_4169	0.24	0.21	0.20	0.21
*T. cruz*i-negative_4170	0.20	0.21	0.12	0.17
*T. cruz*i-negative_4171	0.22	0.20	0.11	0.27
*T. cruz*i-negative_4172	0.13	0.15	0.12	0.25
*T. cruz*i-negative_4173	0.15	0.23	0.15	0.17
*T. cruz*i-negative_4174	0.12	0.17	0.08	0.12
*T. cruz*i-negative_4175	0.19	0.20	0.13	0.21
*T. cruz*i-negative_4176	0.21	0.26	0.18	0.31
*T. cruz*i-negative_4177	0.17	0.17	0.10	0.21

*T. cruz*i-negative_4178	0.13	0.16	0.11	0.16
*T. cruz*i-negative_4179	0.21	0.24	0.12	0.29
*T. cruz*i-negative_4180	0.14	0.17	0.09	0.33
*T. cruz*i-negative_4181	0.07	0.18	0.11	0.12
*T. cruz*i-negative_4182	0.11	0.15	0.08	0.22
*T. cruz*i-negative_4183	0.15	0.16	0.18	0.15
*T. cruz*i-negative_4184	0.17	0.21	0.12	0.23
*T. cruz*i-negative_4185	0.22	0.26	0.13	0.23
*T. cruz*i-negative_4186	0.06	0.09	0.05	0.16
*T. cruz*i-negative_4187	0.10	0.21	0.10	0.27
*T. cruz*i-negative_4188	0.17	0.18	0.11	0.33
*T. cruz*i-negative_4189	0.18	0.29	0.14	0.24
*T. cruz*i-negative_4190	0.12	0.17	0.11	0.38
*T. cruz*i-negative_4191	0.15	0.19	0.11	0.22
*T. cruz*i-negative_4192	0.11	0.15	0.09	0.31
*T. cruz*i-negative_4193	0.15	0.20	0.11	0.26
*T. cruz*i-negative_4194	0.12	0.16	0.11	0.29
*T. cruz*i-negative_4195	0.10	0.15	0.11	0.36
*T. cruz*i-negative_4196	0.18	0.28	0.11	0.40
*T. cruz*i-negative_4197	0.08	0.15	0.10	0.30
*T. cruz*i-negative_4198	0.12	0.17	0.11	0.29
*T. cruz*i-negative_4199	0.17	0.23	0.12	0.34
*T. cruz*i-negative_4200	0.13	0.14	0.10	0.29
*T. cruz*i-negative_4201	0.16	0.17	0.09	0.32
*T. cruz*i-negative_4202	0.12	0.17	0.12	0.39
*T. cruz*i-negative_4203	0.08	0.10	0.07	0.34
*T. cruz*i-negative_4204	0.14	0.18	0.30	0.42
*T. cruz*i-negative_4205	0.18	0.29	0.18	0.37
*T. cruz*i-negative_4206	0.11	0.13	0.07	0.19
*T. cruz*i-negative_4207	0.15	0.15	0.10	0.23
*T. cruz*i-negative_4208	0.13	0.17	0.08	0.23
*T. cruz*i-negative_4209	0.17	0.22	0.12	0.24
*T. cruz*i-negative_4210	0.18	0.23	0.14	0.30
*T. cruz*i-negative_4211	0.11	0.15	0.12	0.33
*T. cruz*i-negative_4212	0.16	0.22	0.11	0.47
*T. cruz*i-negative_4213	0.15	0.30	0.11	0.34
*T. cruz*i-negative_4214	0.08	0.07	0.06	0.21
*T. cruz*i-negative_4215	0.16	0.20	0.25	0.27
*T. cruz*i-negative_4216	0.13	0.13	0.11	0.21
*T. cruz*i-negative_4217	0.15	0.16	0.10	0.18
*T. cruz*i-negative_4218	0.23	0.26	0.13	0.21
*T. cruz*i-negative_4219	0.26	0.30	0.16	0.34

*T. cruz*i-negative_4220	0.71	0.20	0.13	0.34
*T. cruz*i-negative_4221	0.12	0.19	0.18	0.17
*T. cruz*i-negative_4222	0.23	0.25	0.20	0.21
*T. cruz*i-negative_4223	0.14	0.20	0.09	0.14
*T. cruz*i-negative_4224	0.06	0.09	0.17	0.12
*T. cruz*i-negative_4225	0.07	0.10	0.05	0.14
*T. cruz*i-negative_4226	0.11	0.13	0.06	0.11
*T. cruz*i-negative_4227	0.10	0.15	0.08	0.17
*T. cruz*i-negative_4228	0.12	0.14	0.07	0.17
*T. cruz*i-negative_4229	0.24	0.39	0.31	0.25
*T. cruz*i-negative_4230	0.14	0.10	0.12	0.22
*T. cruz*i-negative_4231	0.20	0.29	0.15	0.21
*T. cruz*i-negative_4232	0.17	0.26	0.11	0.21
*T. cruz*i-negative_4233	0.21	0.25	0.13	0.23
*T. cruz*i-negative_4234	0.10	0.13	0.08	0.14
*T. cruz*i-negative_4235	0.10	0.15	0.08	0.16
*T. cruz*i-negative_4236	0.10	0.20	0.24	0.24
*T. cruz*i-negative_4237	0.07	0.07	0.09	0.12
*T. cruz*i-negative_4238	0.14	0.22	0.23	0.27
*T. cruz*i-negative_4239	0.07	0.08	0.10	0.14
*T. cruz*i-negative_4240	0.14	0.13	0.17	0.20
*T. cruz*i-negative_4241	0.14	0.23	0.22	0.26
*T. cruz*i-negative_4242	0.15	0.21	0.21	0.30
*T. cruz*i-negative_4243	0.17	0.15	0.17	0.24
*T. cruz*i-negative_4244	0.18	0.19	0.29	0.27
*T. cruz*i-negative_4245	0.20	0.30	0.25	0.31
*T. cruz*i-negative_4246	0.11	0.14	0.21	0.22
*T. cruz*i-negative_4247	0.12	0.14	0.18	0.18
*T. cruz*i-negative_4248	0.08	0.09	0.17	0.12
*T. cruz*i-negative_4249	0.14	0.16	0.19	0.23
*T. cruz*i-negative_4250	0.10	0.12	0.14	0.23
*T. cruz*i-negative_4251	0.10	0.08	0.14	0.22
*T. cruz*i-negative_4252	0.15	0.16	0.23	0.25
*T. cruz*i-negative_4253	0.15	0.18	0.25	0.32
*T. cruz*i-negative_4254	0.14	0.13	0.19	0.31
*T. cruz*i-negative_4255	0.11	0.08	0.14	0.17
*T. cruz*i-negative_4256	0.12	0.06	0.20	0.17
*T. cruz*i-negative_4257	0.12	0.17	0.15	0.22
*T. cruz*i-negative_4258	0.20	0.20	0.17	0.43
*T. cruz*i-negative_4259	0.10	0.10	0.08	0.20
*T. cruz*i-negative_4260	0.18	0.18	0.15	0.31
*T. cruz*i-negative_4261	0.13	0.09	0.10	0.22
*T. cruz*i-negative_4262	0.12	0.08	0.10	0.20

*T. cruz*i-negative_4263	0.19	0.13	0.16	0.32
*T. cruz*i-negative_4264	0.21	0.18	0.22	0.33
*T. cruz*i-negative_4265	0.23	0.27	0.26	0.40
*T. cruz*i-negative_4266	0.13	0.18	0.13	0.26
*T. cruz*i-negative_4267	0.15	0.18	0.15	0.28
*T. cruz*i-negative_4268	0.12	0.21	0.16	0.21
*T. cruz*i-negative_4269	0.15	0.20	0.15	0.24
*T. cruz*i-negative_4270	0.19	0.19	0.18	0.27
*T. cruz*i-negative_4271	0.15	0.19	0.14	0.25
*T. cruz*i-negative_4272	0.17	0.20	0.16	0.24
*T. cruz*i-negative_4273	0.19	0.22	0.18	0.39
*T. cruz*i-negative_4274	0.13	0.11	0.10	0.21
*T. cruz*i-negative_4275	0.20	0.17	0.15	0.35
*T. cruz*i-negative_4276	0.16	0.18	0.15	0.29
*T. cruz*i-negative_4277	0.30	0.20	0.27	0.40
*T. cruz*i-negative_4278	0.08	0.07	0.09	0.14
*T. cruz*i-negative_4279	0.16	0.16	0.15	0.27
*T. cruz*i-negative_4280	0.30	0.32	0.30	0.50
*T. cruz*i-negative_4281	0.29	0.15	0.19	0.26
*T. cruz*i-negative_4282	0.15	0.10	0.07	0.24
*T. cruz*i-negative_4283	0.14	0.13	0.12	0.25
*T. cruz*i-negative_4284	0.17	0.15	0.13	0.15
*T. cruz*i-negative_4285	0.28	0.24	0.18	0.35
*T. cruz*i-negative_4286	0.29	0.25	0.20	0.39
*T. cruz*i-negative_4287	0.13	0.12	0.16	0.14
*T. cruz*i-negative_4288	0.15	0.11	0.16	0.23
*T. cruz*i-negative_4289	0.25	0.33	0.20	0.39
*T. cruz*i-negative_4290	0.17	0.26	0.21	0.29
*T. cruz*i-negative_4291	0.17	0.21	0.19	0.30
*T. cruz*i-negative_4292	0.11	0.12	0.13	0.17
*T. cruz*i-negative_4293	0.11	0.14	0.15	0.16
*T. cruz*i-negative_4294	0.35	0.21	0.19	0.47
*T. cruz*i-negative_4295	0.11	0.10	0.11	0.17
*T. cruz*i-negative_4296	0.14	0.17	0.18	0.22
*T. cruz*i-negative_4297	0.14	0.19	0.15	0.22
*T. cruz*i-negative_4298	0.23	0.33	0.26	0.42
*T. cruz*i-negative_4299	0.17	0.19	0.37	0.28
*T. cruz*i-negative_4300	0.13	0.20	0.13	0.19
*T. cruz*i-negative_4301	0.23	0.24	0.21	0.38
*T. cruz*i-negative_4302	0.09	0.12	0.13	0.15
*T. cruz*i-negative_4303	0.17	0.14	0.15	0.20
*T. cruz*i-negative_4304	0.20	0.18	0.18	0.27

*T. cruz*i-negative_4305	0.24	0.24	0.17	0.26
*T. cruz*i-negative_4306	0.22	0.31	0.35	0.33
*T. cruz*i-negative_4307	0.18	0.22	0.15	0.22
*T. cruz*i-negative_4308	0.32	0.25	0.20	0.30
*T. cruz*i-negative_4309	0.16	0.22	0.13	0.17
*T. cruz*i-negative_4310	0.25	0.26	0.27	0.31
*T. cruz*i-negative_4311	0.16	0.12	0.13	0.16
*T. cruz*i-negative_4312	0.19	0.24	0.23	0.19
*T. cruz*i-negative_4313	0.13	0.21	0.14	0.17
*T. cruz*i-negative_4314	0.16	0.24	0.19	0.24
*T. cruz*i-negative_4315	0.15	0.20	0.14	0.29
*T. cruz*i-negative_4316	0.36	0.28	0.19	0.48
*T. cruz*i-negative_4317	0.33	0.39	0.24	0.61
*T. cruz*i-negative_4318	0.11	0.17	0.14	0.18
*T. cruz*i-negative_4319	0.12	0.17	0.15	0.13
*T. cruz*i-negative_4320	0.09	0.10	0.31	0.21
*T. cruz*i-negative_4321	0.28	0.25	0.21	0.31
*T. cruz*i-negative_4322	0.25	0.32	0.23	0.24
*T. cruz*i-negative_4323	0.08	0.14	0.23	0.10
*T. cruz*i-negative_4324	0.16	0.20	0.08	0.20
*T. cruz*i-negative_4325	0.13	0.13	0.11	0.30
*T. cruz*i-negative_4326	0.12	0.66	0.09	0.14
*T. cruz*i-negative_4327	0.09	0.09	0.14	0.11
*T. cruz*i-negative_4328	0.23	0.17	0.14	0.19
*T. cruz*i-negative_4329	0.18	0.35	0.20	0.18
*T. cruz*i-negative_4330	0.15	0.10	0.13	0.04
*T. cruz*i-negative_4331	0.10	0.12	0.07	0.13
*T. cruz*i-negative_4332	0.18	0.16	0.17	0.16
*T. cruz*i-negative_4333	0.32	0.27	0.36	0.20
*T. cruz*i-negative_4334	0.19	0.17	0.26	0.16
*T. cruz*i-negative_4335	0.17	0.16	0.16	0.17
*T. cruz*i-negative_4336	0.12	0.11	0.17	0.12
*T. cruz*i-negative_4337	0.26	0.12	0.08	0.11
*T. cruz*i-negative_4338	0.15	0.15	0.08	0.09
*T. cruz*i-negative_4339	0.11	0.11	0.14	0.09
*T. cruz*i-negative_4340	0.16	0.19	0.09	0.15
*T. cruz*i-negative_4341	0.23	0.23	0.13	0.21
*T. cruz*i-negative_4342	0.08	0.09	0.18	0.08
*T. cruz*i-negative_4343	0.11	0.09	0.07	0.09
*T. cruz*i-negative_4344	0.26	0.20	0.07	0.28
*T. cruz*i-negative_4345	0.20	0.26	0.32	0.22
*T. cruz*i-negative_4346	0.11	0.14	0.17	0.08
*T. cruz*i-negative_4347	0.13	0.09	0.10	0.08

*T. cruz*i-negative_4348	0.35	0.32	0.07	0.35
*T. cruz*i-negative_4349	0.26	0.19	0.26	0.22
*T. cruz*i-negative_4350	0.09	0.14	0.17	0.11
*T. cruz*i-negative_4351	0.09	0.08	0.08	0.07
*T. cruz*i-negative_4352	0.17	0.14	0.13	0.14
*T. cruz*i-negative_4353	0.12	0.11	0.12	0.10
*T. cruz*i-negative_4354	0.12	0.12	0.16	0.13
*T. cruz*i-negative_4355	0.15	0.12	0.14	0.13
*T. cruz*i-negative_4356	0.12	0.13	0.11	0.13
*T. cruz*i-negative_4357	0.21	0.22	0.09	0.19
*T. cruz*i-negative_4358	0.12	0.09	0.15	0.08
*T. cruz*i-negative_4359	0.18	0.19	0.07	0.18
*T. cruz*i-negative_4360	0.11	0.12	0.11	0.10
*T. cruz*i-negative_4361	0.18	0.19	0.08	0.13
*T. cruz*i-negative_4362	0.39	0.42	0.15	0.31
*T. cruz*i-negative_4363	0.19	0.17	0.31	0.21
*T. cruz*i-negative_4364	0.14	0.13	0.17	0.13
*T. cruz*i-negative_4365	0.12	0.76	0.13	0.11
*T. cruz*i-negative_4366	0.11	0.12	0.15	0.10
*T. cruz*i-negative_4367	0.12	0.12	0.09	0.11
*T. cruz*i-negative_4368	0.06	0.14	0.11	0.07
*T. cruz*i-negative_4369	0.26	0.24	0.07	0.22
*T. cruz*i-negative_4370	0.13	0.15	0.17	0.36
*T. cruz*i-negative_4371	0.12	0.16	0.17	0.26
*T. cruz*i-negative_4372	0.23	0.20	0.14	0.23
*T. cruz*i-negative_4373	0.17	0.13	0.16	0.13
*T. cruz*i-negative_4374	0.07	0.09	0.14	0.07
*T. cruz*i-negative_4375	0.20	0.12	0.29	0.17
*T. cruz*i-negative_4376	0.10	0.10	0.11	0.14
*T. cruz*i-negative_4377	0.08	0.08	0.12	0.08
*T. cruz*i-negative_4378	0.10	0.12	0.07	0.12
*T. cruz*i-negative_4379	0.10	0.15	0.07	0.09
*T. cruz*i-negative_4380	0.12	0.14	0.11	0.14
*T. cruz*i-negative_4381	0.15	0.11	0.10	0.11
*T. cruz*i-negative_4382	0.07	0.09	0.11	0.06
*T. cruz*i-negative_4383	0.15	0.12	0.14	0.18
*T. cruz*i-negative_4384	0.14	0.13	0.25	0.18
*T. cruz*i-negative_4385	0.08	0.08	0.27	0.11
*T. cruz*i-negative_4386	0.14	0.11	0.10	0.18
*T. cruz*i-negative_4387	0.26	0.17	0.17	0.21
*T. cruz*i-negative_4388	0.10	0.10	0.14	0.20
*T. cruz*i-negative_4389	0.10	0.10	0.09	0.10

*T. cruz*i-negative_4390	0.12	0.14	0.19	0.12
*T. cruz*i-negative_4391	0.11	0.11	0.18	0.29
*T. cruz*i-negative_4392	0.25	0.18	0.23	0.19
*T. cruz*i-negative_4393	0.12	0.15	0.18	0.16
*T. cruz*i-negative_4394	0.09	0.09	0.13	0.06
*T. cruz*i-negative_4395	0.13	0.13	0.05	0.10
*T. cruz*i-negative_4396	0.33	0.17	0.06	0.19
*T. cruz*i-negative_4397	0.18	0.16	0.13	0.18
*T. cruz*i-negative_4398	0.22	0.21	0.15	0.19
*T. cruz*i-negative_4399	0.20	0.16	0.18	0.18
*T. cruz*i-negative_4400	0.56	0.42	0.13	0.72
*T. cruz*i-negative_4401	0.11	0.10	0.39	0.11
*T. cruz*i-negative_4402	0.20	0.16	0.11	0.19
*T. cruz*i-negative_4403	0.09	0.08	0.13	0.13
*T. cruz*i-negative_4404	0.16	0.10	0.14	0.19
*T. cruz*i-negative_4405	0.10	0.07	0.09	0.11
*T. cruz*i-negative_4406	0.11	0.06	0.10	0.12
*T. cruz*i-negative_4407	0.20	0.13	0.15	0.19
*T. cruz*i-negative_4408	0.08	0.05	0.17	0.10
*T. cruz*i-negative_4409	0.15	0.21	0.14	0.18
*T. cruz*i-negative_4410	0.18	0.11	0.15	0.17
*T. cruz*i-negative_4411	0.13	0.16	0.11	0.13
*T. cruz*i-negative_4412	0.14	0.13	0.12	0.18
*T. cruz*i-negative_4413	0.18	0.21	0.27	0.19
*T. cruz*i-negative_4414	0.20	0.14	0.22	0.19
*T. cruz*i-negative_4415	0.19	0.22	0.18	0.25
*T. cruz*i-negative_4416	0.13	0.15	0.14	0.18
*T. cruz*i-negative_4417	0.23	0.15	0.24	0.27
*T. cruz*i-negative_4418	0.21	0.14	0.18	0.21
*T. cruz*i-negative_4419	0.14	0.08	0.12	0.14
*T. cruz*i-negative_4420	0.06	0.06	0.06	0.12
*T. cruz*i-negative_4421	0.09	0.06	0.11	0.10
*T. cruz*i-negative_4422	0.13	0.08	0.18	0.14
*T. cruz*i-negative_4423	0.15	0.10	0.35	0.19
*T. cruz*i-negative_4424	0.29	0.12	0.18	0.24
*T. cruz*i-negative_4425	0.20	0.14	0.21	0.26
*T. cruz*i-negative_4426	0.12	0.10	0.17	0.13
*T. cruz*i-negative_4427	0.15	0.11	0.13	0.15
*T. cruz*i-negative_4428	0.17	0.21	0.17	0.20
*T. cruz*i-negative_4429	0.14	0.28	0.12	0.15
*T. cruz*i-negative_4430	0.09	0.07	0.08	0.11
*T. cruz*i-negative_4431	0.14	0.09	0.12	0.16
*T. cruz*i-negative_4432	0.13	0.10	0.12	0.15

*T. cruz*i-negative_4433	0.18	0.15	0.17	0.21
*T. cruz*i-negative_4434	0.11	0.10	0.11	0.13
*T. cruz*i-negative_4435	0.15	0.22	0.11	0.13
*T. cruz*i-negative_4436	0.14	0.10	0.11	0.11
*T. cruz*i-negative_4437	0.10	0.08	0.10	0.10
*T. cruz*i-negative_4438	0.06	0.07	0.07	0.12
*T. cruz*i-negative_4439	0.07	0.06	0.15	0.10
*T. cruz*i-negative_4440	0.13	0.09	0.12	0.14
*T. cruz*i-negative_4441	0.11	0.10	0.16	0.17
*T. cruz*i-negative_4442	0.12	0.10	0.10	0.14
*T. cruz*i-negative_4443	0.17	0.13	0.15	0.21
*T. cruz*i-negative_4444	0.09	0.09	0.07	0.10
*T. cruz*i-negative_4445	0.11	0.08	0.09	0.12
*T. cruz*i-negative_4446	0.10	0.09	0.09	0.07
*T. cruz*i-negative_4447	0.14	0.18	0.10	0.15
*T. cruz*i-negative_4448	0.28	0.21	0.24	0.35
*T. cruz*i-negative_4449	0.26	0.17	0.18	0.23
*T. cruz*i-negative_4450	0.18	0.17	0.17	0.16
*T. cruz*i-negative_4451	0.09	0.44	0.08	0.10
*T. cruz*i-negative_4452	0.15	0.18	0.61	0.14
*T. cruz*i-negative_4453	0.07	0.05	0.09	0.10
*T. cruz*i-negative_4454	0.12	0.08	0.08	0.13
*T. cruz*i-negative_4455	0.15	0.10	0.14	0.22
*T. cruz*i-negative_4456	0.13	0.10	0.13	0.19
*T. cruz*i-negative_4457	0.18	0.13	0.16	0.21
*T. cruz*i-negative_4458	0.16	0.12	0.15	0.15
*T. cruz*i-negative_4459	0.11	0.09	0.12	0.13
*T. cruz*i-negative_4460	0.11	0.30	0.15	0.13
*T. cruz*i-negative_4461	0.06	0.08	0.05	0.08
*T. cruz*i-negative_4462	0.06	0.04	0.10	0.07
*T. cruz*i-negative_4463	0.25	0.19	0.25	0.37
*T. cruz*i-negative_4464	0.13	0.09	0.14	0.16
*T. cruz*i-negative_4465	0.13	0.14	0.12	0.14
*T. cruz*i-negative_4466	0.10	0.09	0.12	0.12
*T. cruz*i-negative_4467	0.09	0.14	0.19	0.13
*T. cruz*i-negative_4468	0.19	0.14	0.16	0.20
*T. cruz*i-negative_4469	0.13	0.13	0.11	0.13
*T. cruz*i-negative_4470	0.11	0.10	0.09	0.11
*T. cruz*i-negative_4471	0.09	0.09	0.08	0.10
*T. cruz*i-negative_4472	0.11	0.05	0.56	0.13
*T. cruz*i-negative_4473	0.09	0.08	0.09	0.12
*T. cruz*i-negative_4474	0.13	0.10	0.13	0.14

T. cruzi-negative_4475	0.12	0.12	0.12	0.13
T. cruzi-negative_4476	0.13	0.11	0.11	0.15
T. cruzi-negative_4477	0.07	0.08	0.06	0.09
T. cruzi-negative_4478	0.09	0.11	0.14	0.16
T. cruzi-negative_4479	0.15	0.12	0.13	0.17
T. cruzi-negative_4480	0.20	0.15	0.20	0.25
T. cruzi-negative_4481	0.12	0.18	0.15	0.14
T. cruzi-negative_4482	0.16	0.13	0.17	0.15
T. cruzi-negative_4483	0.08	0.07	0.08	0.09
T. cruzi-negative_4484	0.16	0.16	0.12	0.17
T. cruzi-negative_4485	0.07	0.08	0.07	0.08
T. cruzi-negative_4486	0.13	0.12	0.15	0.13
T. cruzi-negative_4487	0.08	0.08	0.25	0.13
T. cruzi-negative_4488	0.18	0.09	0.11	0.14
T. cruzi-negative_4489	0.12	0.06	0.06	0.11
T. cruzi-negative_4490	0.27	0.16	0.19	0.29
T. cruzi-negative_4491	0.19	0.12	0.12	0.18
T. cruzi-negative_4492	0.23	0.14	0.14	0.20
T. cruzi-negative_4493	0.25	0.17	0.53	0.23
T. cruzi-negative_4494	0.35	0.16	0.20	0.30
T. cruzi-negative_4495	0.16	0.09	0.10	0.19
T. cruzi-negative_4496	0.22	0.16	0.12	0.19
T. cruzi-negative_4497	0.21	0.25	0.10	0.16
T. cruzi-negative_4498	0.16	0.07	0.26	0.31
T. cruzi-negative_4499	0.15	0.14	0.08	0.11
T. cruzi-negative_4500	0.23	0.21	0.15	0.26
T. cruzi-negative_4501	0.17	0.21	0.09	0.16
T. cruzi-negative_4502	0.13	0.08	0.09	0.13
T. cruzi-negative_4503	0.21	0.12	0.13	0.19
T. cruzi-negative_4504	0.20	0.11	0.10	0.16
T. cruzi-negative_4505	0.13	0.09	0.07	0.15
T. cruzi-negative_4506	0.16	0.09	0.08	0.17
T. cruzi-negative_4507	0.16	0.08	0.10	0.12
T. cruzi-negative_4508	0.21	0.20	0.15	0.23
T. cruzi-negative_4509	0.29	0.42	0.16	0.26
T. cruzi-negative_4510	0.21	0.14	0.13	0.25
T. cruzi-negative_4511	0.14	0.10	0.08	0.12
T. cruzi-negative_4512	0.11	0.07	0.05	0.11
T. cruzi-negative_4513	0.17	0.12	0.10	0.14
T. cruzi-negative_4514	0.17	0.09	0.11	0.15
T. cruzi-negative_4515	0.14	0.06	0.06	0.13
T. cruzi-negative_4516	0.17	0.11	0.16	0.17
T. cruzi-negative_4517	0.16	0.12	0.10	0.19

T. cruzi-negative_4518	0.17	0.11	0.12	0.15
T. cruzi-negative_4519	0.15	0.12	0.25	0.18
T. cruzi-negative_4520	0.17	0.08	0.09	0.17
T. cruzi-negative_4521	0.16	0.06	0.06	0.14
T. cruzi-negative_4522	0.18	0.15	0.14	0.17
T. cruzi-negative_4523	0.33	0.25	0.23	0.31
T. cruzi-negative_4524	0.20	0.12	0.12	0.22
T. cruzi-negative_4525	0.18	0.11	0.11	0.16
T. cruzi-negative_4526	0.22	0.18	0.18	0.25
T. cruzi-negative_4527	0.21	0.12	0.11	0.20
T. cruzi-negative_4528	0.17	0.16	0.09	0.16
T. cruzi-negative_4529	0.14	0.08	0.08	0.13
T. cruzi-negative_4530	0.22	0.18	0.13	0.18
T. cruzi-negative_4531	0.21	0.13	0.23	0.36
T. cruzi-negative_4532	0.10	0.06	0.09	0.11
T. cruzi-negative_4533	0.27	0.16	0.16	0.31
T. cruzi-negative_4534	0.16	0.20	0.11	0.15
T. cruzi-negative_4535	0.14	0.07	0.08	0.14
T. cruzi-negative_4536	0.11	0.09	0.06	0.12
T. cruzi-negative_4537	0.14	0.08	0.06	0.14
T. cruzi-negative_4538	0.31	0.29	0.21	0.31
T. cruzi-negative_4539	0.21	0.22	0.10	0.19
T. cruzi-negative_4540	0.20	0.10	0.15	0.18
T. cruzi-negative_4541	0.20	0.16	0.10	0.17
T. cruzi-negative_4542	0.14	0.08	0.10	0.10
T. cruzi-negative_4543	0.19	0.11	0.10	0.18
T. cruzi-negative_4544	0.21	0.13	0.09	0.18
T. cruzi-negative_4545	0.28	0.11	0.10	0.16
T. cruzi-negative_4546	0.18	0.18	0.11	0.17
T. cruzi-negative_4547	0.20	0.14	0.12	0.23
T. cruzi-negative_4548	0.23	0.16	0.14	0.21
T. cruzi-negative_4549	0.15	0.11	0.10	0.16
T. cruzi-negative_4550	0.15	0.09	0.12	0.14
T. cruzi-negative_4551	0.09	0.09	0.05	0.10
T. cruzi-negative_4552	0.17	0.25	0.18	0.20
T. cruzi-negative_4553	0.15	0.11	0.07	0.18
T. cruzi-negative_4554	0.13	0.11	0.13	0.18
T. cruzi-negative_4555	0.19	0.10	0.06	0.10
T. cruzi-negative_4556	0.20	0.14	0.11	0.19
T. cruzi-negative_4557	0.19	0.17	0.12	0.17
T. cruzi-negative_4558	0.15	0.08	0.29	0.12
T. cruzi-negative_4559	0.16	0.06	0.07	0.12

T. cruzi-negative_4560	0.10	0.05	0.04	0.08
T. cruzi-negative_4561	0.08	0.05	0.05	0.11
T. cruzi-negative_4562	0.24	0.18	0.13	0.20
T. cruzi-negative_4563	0.15	0.09	0.07	0.12
T. cruzi-negative_4564	0.17	0.14	0.09	0.18
T. cruzi-negative_4565	0.14	0.12	0.10	0.13
T. cruzi-negative_4566	0.19	0.16	0.21	0.19
T. cruzi-negative_4567	0.10	0.21	0.07	0.13
T. cruzi-negative_4568	0.11	0.08	0.06	0.10
T. cruzi-negative_4569	0.14	0.11	0.06	0.20
T. cruzi-negative_4570	0.14	0.09	0.06	0.11
T. cruzi-negative_4571	0.16	0.13	0.09	0.18
T. cruzi-negative_4572	0.26	0.18	0.25	0.23
T. cruzi-negative_4573	0.18	0.12	0.17	0.22
T. cruzi-negative_4574	0.19	0.20	0.13	0.16
T. cruzi-negative_4575	0.15	0.11	0.09	0.13
T. cruzi-negative_4576	0.22	0.13	0.15	0.19
T. cruzi-negative_4577	0.17	0.14	0.17	0.26
T. cruzi-negative_4578	0.40	0.14	0.17	0.26
T. cruzi-negative_4579	0.21	0.10	0.17	0.14
T. cruzi-negative_4580	0.18	0.11	0.14	0.16
T. cruzi-negative_4581	0.21	0.16	0.14	0.21
T. cruzi-negative_4582	0.26	0.17	0.17	0.28
T. cruzi-negative_4583	0.16	0.08	0.10	0.16
T. cruzi-negative_4584	0.17	0.12	0.12	0.15
T. cruzi-negative_4585	0.23	0.14	0.17	0.28
T. cruzi-negative_4586	0.24	0.12	0.19	0.25
T. cruzi-negative_4587	0.16	0.17	0.15	0.17
T. cruzi-negative_4588	0.15	0.11	0.10	0.12
T. cruzi-negative_4589	0.17	0.09	0.10	0.14
T. cruzi-negative_4590	0.09	0.06	0.13	0.10
T. cruzi-negative_4591	0.24	0.16	0.12	0.22
T. cruzi-negative_4592	0.18	0.13	0.12	0.19
T. cruzi-negative_4593	0.16	0.11	0.06	0.13
T. cruzi-negative_4594	0.21	0.67	0.13	0.29
T. cruzi-negative_4595	0.14	0.09	0.09	0.12
T. cruzi-negative_4596	0.17	0.12	0.20	0.19
T. cruzi-negative_4597	0.19	0.11	0.09	0.16
T. cruzi-negative_4598	0.18	0.09	0.17	0.19
T. cruzi-negative_4599	0.24	0.17	0.16	0.22
T. cruzi-negative_4600	0.15	0.13	0.10	0.15
T. cruzi-negative_4601	0.14	0.10	0.07	0.15
T. cruzi-negative_4602	0.17	0.07	0.09	0.13

*T. cruz*i-negative_4603	0.17	0.14	0.13	0.16
*T. cruz*i-negative_4604	0.17	0.11	0.18	0.15
*T. cruz*i-negative_4605	0.11	0.07	0.22	0.15
*T. cruz*i-negative_4606	0.19	0.13	0.18	0.21
*T. cruz*i-negative_4607	0.25	0.15	0.17	0.25
*T. cruz*i-negative_4608	0.28	0.20	0.21	0.32
*T. cruz*i-negative_4609	0.16	0.12	0.20	0.17
*T. cruz*i-negative_4610	0.15	0.11	0.09	0.17
*T. cruz*i-negative_4611	0.24	0.17	0.15	0.22
*T. cruz*i-negative_4612	0.21	0.14	0.15	0.14
*T. cruz*i-negative_4613	0.24	0.13	0.17	0.25
*T. cruz*i-negative_4614	0.30	0.16	0.34	0.31
*T. cruz*i-negative_4615	0.18	0.17	0.12	0.19
*T. cruz*i-negative_4616	0.20	0.17	0.24	0.23
*T. cruz*i-negative_4617	0.24	0.12	0.16	0.23
*T. cruz*i-negative_4618	0.23	0.18	0.15	0.25
*T. cruz*i-negative_4619	0.13	0.07	0.11	0.12
*T. cruz*i-negative_4620	0.22	0.14	0.17	0.18
*T. cruz*i-negative_4621	0.22	0.12	0.09	0.20
*T. cruz*i-negative_4622	0.21	0.18	0.18	0.22
*T. cruz*i-negative_4623	0.16	0.10	0.12	0.18
*T. cruz*i-negative_4624	0.18	0.13	0.12	0.18
*T. cruz*i-negative_4625	0.26	0.17	0.13	0.22
*T. cruz*i-negative_4626	0.22	0.14	0.16	0.20
*T. cruz*i-negative_4627	0.22	0.15	0.19	0.22
*T. cruz*i-negative_4628	0.32	0.27	0.22	0.31
*T. cruz*i-negative_4629	0.15	0.14	0.12	0.17
*T. cruz*i-negative_4630	0.20	0.15	0.15	0.18
*T. cruz*i-negative_4631	0.25	0.14	0.18	0.22
*T. cruz*i-negative_4632	0.15	0.12	0.10	0.16
*T. cruz*i-negative_4633	0.30	0.17	0.18	0.26
*T. cruz*i-negative_4634	0.14	0.11	0.10	0.16
*T. cruz*i-negative_4635	0.15	0.11	0.13	0.14
*T. cruz*i-negative_4636	0.12	0.07	0.16	0.08
*T. cruz*i-negative_4637	0.18	0.10	0.13	0.17
*T. cruz*i-negative_4638	0.26	0.16	0.16	0.26
*T. cruz*i-negative_4639	0.30	0.15	0.13	0.12
*T. cruz*i-negative_4640	0.24	0.33	0.19	0.23
*T. cruz*i-negative_4641	0.22	0.17	0.13	0.26
*T. cruz*i-negative_4642	0.19	0.12	0.13	0.22
*T. cruz*i-negative_4643	0.24	0.16	0.19	0.33
*T. cruz*i-negative_4644	0.21	0.16	0.18	0.24

T. cruzi-negative_4645	0.23	0.24	0.17	0.26
T. cruzi-negative_4646	0.18	0.14	0.07	0.18
T. cruzi-negative_4647	0.16	0.11	0.11	0.13
T. cruzi-negative_4648	0.22	0.15	0.13	0.24
T. cruzi-negative_4649	0.14	0.08	0.11	0.13
T. cruzi-negative_4650	0.32	0.22	0.20	0.28
T. cruzi-negative_4651	0.18	0.13	0.13	0.20
T. cruzi-negative_4652	0.21	0.13	0.12	0.17
T. cruzi-negative_4653	0.15	0.07	0.10	0.13
T. cruzi-negative_4654	0.26	0.14	0.12	0.22
T. cruzi-negative_4655	0.19	0.16	0.22	0.20
T. cruzi-negative_4656	0.16	0.08	0.07	0.08
T. cruzi-negative_4657	0.17	0.09	0.08	0.10
T. cruzi-negative_4658	0.20	0.12	0.15	0.12
T. cruzi-negative_4659	0.18	0.08	0.08	0.08
T. cruzi-negative_4660	0.12	0.05	0.08	0.06
T. cruzi-negative_4661	0.53	0.15	0.15	0.14
T. cruzi-negative_4662	0.11	0.09	0.06	0.08
T. cruzi-negative_4663	0.29	0.16	0.20	0.23
T. cruzi-negative_4664	0.27	0.16	0.15	0.17
T. cruzi-negative_4665	0.18	0.10	0.13	0.08
T. cruzi-negative_4666	0.14	0.09	0.08	0.07
T. cruzi-negative_4667	0.14	0.09	0.17	0.07
T. cruzi-negative_4668	0.18	0.09	0.12	0.13
T. cruzi-negative_4669	0.19	0.13	0.14	0.15
T. cruzi-negative_4670	0.11	0.03	0.05	0.06
T. cruzi-negative_4671	0.22	0.17	0.15	0.21
T. cruzi-negative_4672	0.13	0.05	0.05	0.10
T. cruzi-negative_4673	0.13	0.07	0.09	0.06
T. cruzi-negative_4674	0.20	0.15	0.18	0.17
T. cruzi-negative_4675	0.10	0.04	0.06	0.05
T. cruzi-negative_4676	0.18	0.10	0.17	0.14
T. cruzi-negative_4677	0.29	0.19	0.16	0.18
T. cruzi-negative_4678	0.26	0.15	0.16	0.22
T. cruzi-negative_4679	0.20	0.11	0.13	0.13
T. cruzi-negative_4680	0.14	0.09	0.08	0.10
T. cruzi-negative_4681	0.18	0.11	0.09	0.11
T. cruzi-negative_4682	0.20	0.13	0.14	0.12
T. cruzi-negative_4683	0.18	0.14	0.13	0.13
T. cruzi-negative_4684	0.72	0.65	0.70	0.81
T. cruzi-negative_4685	0.27	0.15	0.16	0.19
T. cruzi-negative_4686	0.15	0.09	0.18	0.08
T. cruzi-negative_4687	0.22	0.30	0.11	0.09

T. cruzi-negative_4688	0.16	0.09	0.09	0.11
T. cruzi-negative_4689	0.17	0.09	0.17	0.09
T. cruzi-negative_4690	0.14	0.05	0.08	0.05
T. cruzi-negative_4691	0.12	0.13	0.08	0.05
T. cruzi-negative_4692	0.17	0.09	0.16	0.11
T. cruzi-negative_4693	0.14	0.06	0.06	0.07
T. cruzi-negative_4694	0.24	0.15	0.24	0.14
T. cruzi-negative_4695	0.16	0.12	0.08	0.09
T. cruzi-negative_4696	0.24	0.13	0.20	0.14
T. cruzi-negative_4697	0.11	0.10	0.22	0.11
T. cruzi-negative_4698	0.14	0.03	0.11	0.10
T. cruzi-negative_4699	0.26	0.15	0.27	0.22
T. cruzi-negative_4700	0.12	0.07	0.79	0.15
T. cruzi-negative_4701	0.27	0.17	0.16	0.19
T. cruzi-negative_4702	0.20	0.09	0.15	0.15
T. cruzi-negative_4703	0.13	0.06	0.09	0.05
T. cruzi-negative_4704	0.18	0.13	0.11	0.09
T. cruzi-negative_4705	0.22	0.14	0.15	0.11
T. cruzi-negative_4706	0.31	0.05	0.19	0.09
T. cruzi-negative_4707	0.26	0.13	0.16	0.14
T. cruzi-negative_4708	0.18	0.11	0.12	0.09
T. cruzi-negative_4709	0.14	0.07	0.07	0.03
T. cruzi-negative_4710	0.08	0.04	0.02	0.02
T. cruzi-negative_4711	0.13	0.07	0.06	0.04
T. cruzi-negative_4712	0.12	0.11	0.06	0.05
T. cruzi-negative_4713	0.14	0.09	0.07	0.10
T. cruzi-negative_4714	0.16	0.11	0.12	0.08
T. cruzi-negative_4715	0.17	0.18	0.14	0.14
T. cruzi-negative_4716	0.12	0.07	0.04	0.08
T. cruzi-negative_4717	0.13	0.08	0.26	0.06
T. cruzi-negative_4718	0.16	0.10	0.10	0.07
T. cruzi-negative_4719	0.13	0.08	0.15	0.05
T. cruzi-negative_4720	0.17	0.11	0.07	0.08
T. cruzi-negative_4721	0.24	0.17	0.15	0.21
T. cruzi-negative_4722	0.09	0.10	0.05	0.07
T. cruzi-negative_4723	0.18	0.09	0.25	0.08
T. cruzi-negative_4724	0.14	0.13	0.34	0.12
T. cruzi-negative_4725	0.13	0.23	0.03	0.04
T. cruzi-negative_4726	0.16	0.10	0.08	0.11
T. cruzi-negative_4727	0.55	0.70	0.53	0.95
T. cruzi-negative_4728	0.17	0.13	0.09	0.15
T. cruzi-negative_4729	0.33	0.23	0.20	0.31

*T. cruz*i-negative_4730	0.19	0.19	0.12	0.20
*T. cruz*i-negative_4731	0.18	0.13	0.33	0.11
*T. cruz*i-negative_4732	0.12	0.05	0.03	0.04
*T. cruz*i-negative_4733	0.17	0.08	0.07	0.07
*T. cruz*i-negative_4734	0.15	0.12	0.10	0.08
*T. cruz*i-negative_4735	0.32	0.22	0.20	0.27
*T. cruz*i-negative_4736	0.08	0.21	0.13	0.21
*T. cruz*i-negative_4737	0.04	0.16	0.15	0.15
*T. cruz*i-negative_4738	0.03	0.10	0.10	0.08
*T. cruz*i-negative_4739	0.12	0.10	0.09	0.10
*T. cruz*i-negative_4740	0.07	0.11	0.08	0.08
*T. cruz*i-negative_4741	0.04	0.08	0.13	0.08
*T. cruz*i-negative_4742	0.05	0.12	0.10	0.13
*T. cruz*i-negative_4743	0.06	0.21	0.20	0.22
*T. cruz*i-negative_4744	0.02	0.08	0.06	0.05
*T. cruz*i-negative_4745	0.02	0.13	0.45	0.10
*T. cruz*i-negative_4746	0.07	0.19	0.11	0.18
*T. cruz*i-negative_4747	0.04	0.09	0.08	0.04
*T. cruz*i-negative_4748	0.08	0.15	0.16	0.15
*T. cruz*i-negative_4749	0.07	0.14	0.25	0.11
*T. cruz*i-negative_4750	0.03	0.10	0.09	0.08
*T. cruz*i-negative_4751	0.05	0.14	0.63	0.09
*T. cruz*i-negative_4752	0.02	0.12	0.12	0.12
*T. cruz*i-negative_4753	0.02	0.11	0.24	0.08
*T. cruz*i-negative_4754	0.14	0.21	0.24	0.17
*T. cruz*i-negative_4755	0.14	0.18	0.18	0.17
*T. cruz*i-negative_4756	0.06	0.14	0.10	0.10
*T. cruz*i-negative_4757	0.02	0.33	0.12	0.06
*T. cruz*i-negative_4758	0.07	0.19	0.14	0.15
*T. cruz*i-negative_4759	0.02	0.10	0.05	0.05
*T. cruz*i-negative_4760	0.01	0.05	0.03	0.02
*T. cruz*i-negative_4761	0.06	0.14	0.15	0.19
*T. cruz*i-negative_4762	0.32	0.34	0.23	0.37
*T. cruz*i-negative_4763	0.07	0.12	0.10	0.05
*T. cruz*i-negative_4764	0.16	0.28	0.19	0.27
*T. cruz*i-negative_4765	0.08	0.21	0.13	0.13
*T. cruz*i-negative_4766	0.01	0.06	0.06	0.02
*T. cruz*i-negative_4767	0.12	0.31	0.23	0.33
*T. cruz*i-negative_4768	0.10	0.15	0.09	0.16
*T. cruz*i-negative_4769	0.15	0.35	0.27	0.43
*T. cruz*i-negative_4770	0.27	0.26	0.24	0.26
*T. cruz*i-negative_4771	0.07	0.05	0.06	0.05
*T. cruz*i-negative_4772	0.06	0.12	0.09	0.10

T. cruzi-negative_4773	0.07	0.18	0.12	0.15
T. cruzi-negative_4774	0.01	0.08	0.06	0.04
T. cruzi-negative_4775	0.02	0.09	0.04	0.08
T. cruzi-negative_4776	0.04	0.16	0.10	0.12
T. cruzi-negative_4777	0.04	0.14	0.10	0.10
T. cruzi-negative_4778	0.04	0.06	0.04	0.04
T. cruzi-negative_4779	0.22	0.32	0.26	0.53
T. cruzi-negative_4780	0.09	0.20	0.19	0.19
T. cruzi-negative_4781	0.06	0.13	0.12	0.11
T. cruzi-negative_4782	0.11	0.30	0.31	0.32
T. cruzi-negative_4783	0.04	0.14	0.10	0.13
T. cruzi-negative_4784	0.02	0.15	0.07	0.10
T. cruzi-negative_4785	0.02	0.13	0.12	0.10
T. cruzi-negative_4786	0.07	0.12	0.08	0.08
T. cruzi-negative_4787	0.05	0.08	0.10	0.16
T. cruzi-negative_4788	0.10	0.22	0.12	0.15
T. cruzi-negative_4789	0.06	0.18	0.18	0.14
T. cruzi-negative_4790	0.10	0.25	0.18	0.25
T. cruzi-negative_4791	0.36	0.57	0.38	0.78
T. cruzi-negative_4792	0.05	0.09	0.06	0.05
T. cruzi-negative_4793	0.04	0.12	0.06	0.06
T. cruzi-negative_4794	0.10	0.17	0.11	0.13
T. cruzi-negative_4795	0.13	0.24	0.12	0.16
T. cruzi-negative_4796	0.04	0.06	0.04	0.03
T. cruzi-negative_4797	0.05	0.14	0.22	0.16
T. cruzi-negative_4798	0.15	0.35	0.26	0.35
T. cruzi-negative_4799	0.06	0.12	0.12	0.10
T. cruzi-negative_4800	0.08	0.15	0.09	0.10
T. cruzi-negative_4801	0.12	0.24	0.23	0.20
T. cruzi-negative_4802	0.09	0.08	0.06	0.08
T. cruzi-negative_4803	0.72	0.56	0.18	0.12
T. cruzi-negative_4804	0.12	0.18	0.11	0.14
T. cruzi-negative_4805	0.11	0.22	0.21	0.23
T. cruzi-negative_4806	0.10	0.29	0.26	0.36
T. cruzi-negative_4807	0.10	0.20	0.14	0.29
T. cruzi-negative_4808	0.08	0.19	0.15	0.20
T. cruzi-negative_4809	0.08	0.14	0.16	0.07
T. cruzi-negative_4810	0.26	0.24	0.21	0.37
T. cruzi-negative_4811	0.04	0.09	0.06	0.09
T. cruzi-negative_4812	0.09	0.21	0.22	0.18
T. cruzi-negative_4813	0.08	0.21	0.15	0.10
T. cruzi-negative_4814	0.14	0.24	0.34	0.29

*T. cruz*i-negative_4815	0.03	0.13	0.10	0.04
*T. cruz*i-negative_4816	0.09	0.22	0.18	0.13
*T. cruz*i-negative_4817	0.12	0.11	0.10	0.08
*T. cruz*i-negative_4818	0.14	0.29	0.14	0.41
*T. cruz*i-negative_4819	0.24	0.56	0.20	0.62
*T. cruz*i-negative_4820	0.03	0.09	0.10	0.17
*T. cruz*i-negative_4821	0.09	0.14	0.14	0.26
*T. cruz*i-negative_4822	0.34	0.49	0.45	0.72
*T. cruz*i-negative_4823	0.16	0.20	0.17	0.33
*T. cruz*i-negative_4824	0.14	0.18	0.12	0.22
*T. cruz*i-negative_4825	0.05	0.09	0.08	0.15
*T. cruz*i-negative_4826	0.07	0.12	0.14	0.17
*T. cruz*i-negative_4827	0.17	0.28	0.30	0.31
*T. cruz*i-negative_4828	0.13	0.18	0.11	0.27
*T. cruz*i-negative_4829	0.09	0.15	0.14	0.21
*T. cruz*i-negative_4830	0.05	0.13	0.06	0.16
*T. cruz*i-negative_4831	0.10	0.13	0.11	0.20
*T. cruz*i-negative_4832	0.06	0.14	0.08	0.12
*T. cruz*i-negative_4833	0.53	0.23	0.10	0.22
*T. cruz*i-negative_4834	0.03	0.06	0.07	0.09
*T. cruz*i-negative_4835	0.35	0.24	0.32	0.61
*T. cruz*i-negative_4836	0.08	0.11	0.14	0.17
*T. cruz*i-negative_4837	0.06	0.17	0.05	0.06
*T. cruz*i-negative_4838	0.11	0.19	0.12	0.16
*T. cruz*i-negative_4839	0.07	0.15	0.07	0.11
*T. cruz*i-negative_4840	0.07	0.10	0.08	0.10
*T. cruz*i-negative_4841	0.09	0.12	0.09	0.15
*T. cruz*i-negative_4842	0.11	0.13	0.22	0.17
*T. cruz*i-negative_4843	0.07	0.14	0.10	0.19
*T. cruz*i-negative_4844	0.08	0.12	0.11	0.26
*T. cruz*i-negative_4845	0.04	0.10	0.08	0.16
*T. cruz*i-negative_4846	0.07	0.12	0.07	0.17
*T. cruz*i-negative_4847	0.06	0.15	0.08	0.08
*T. cruz*i-negative_4848	0.10	0.20	0.16	0.21
*T. cruz*i-negative_4849	0.13	0.17	0.13	0.21
*T. cruz*i-negative_4850	0.13	0.21	0.33	0.33
*T. cruz*i-negative_4851	0.06	0.13	0.10	0.17
*T. cruz*i-negative_4852	0.14	0.18	0.18	0.32
*T. cruz*i-negative_4853	0.06	0.13	0.10	0.23
*T. cruz*i-negative_4854	0.06	0.12	0.11	0.14
*T. cruz*i-negative_4855	0.15	0.19	0.16	0.32
*T. cruz*i-negative_4856	0.08	0.16	0.12	0.17
*T. cruz*i-negative_4857	0.16	0.17	0.25	0.30

T. cruzi-negative_4858	0.06	0.11	0.13	0.18
T. cruzi-negative_4859	0.03	0.10	0.05	0.10
T. cruzi-negative_4860	0.10	0.15	0.08	0.13
T. cruzi-negative_4861	0.10	0.17	0.16	0.22
T. cruzi-negative_4862	0.03	0.15	0.07	0.07
T. cruzi-negative_4863	0.10	0.12	0.09	0.16
T. cruzi-negative_4864	0.08	0.15	0.12	0.17
T. cruzi-negative_4865	0.05	0.21	0.06	0.11
T. cruzi-negative_4866	0.08	0.16	0.12	0.19
T. cruzi-negative_4867	0.07	0.12	0.09	0.20
T. cruzi-negative_4868	0.10	0.15	0.15	0.19
T. cruzi-negative_4869	0.11	0.17	0.13	0.32
T. cruzi-negative_4870	0.12	0.27	0.10	0.26
T. cruzi-negative_4871	0.12	0.17	0.16	0.41
T. cruzi-negative_4872	0.07	0.13	0.15	0.17
T. cruzi-negative_4873	0.11	0.12	0.09	0.13
T. cruzi-negative_4874	0.11	0.10	0.15	0.16
T. cruzi-negative_4875	0.11	0.16	0.14	0.23
T. cruzi-negative_4876	0.02	0.07	0.05	0.05
T. cruzi-negative_4877	0.20	0.32	0.20	0.48
T. cruzi-negative_4878	0.10	0.20	0.09	0.24
T. cruzi-negative_4879	0.08	0.11	0.16	0.17
T. cruzi-negative_4880	0.12	0.20	0.14	0.35
T. cruzi-negative_4881	0.10	0.17	0.10	0.23
T. cruzi-negative_4882	0.15	0.21	0.16	0.31
T. cruzi-negative_4883	0.12	0.20	0.16	0.24
T. cruzi-negative_4884	0.05	0.09	0.07	0.09
T. cruzi-negative_4885	0.06	0.12	0.86	0.09
T. cruzi-negative_4886	0.12	0.15	0.10	0.17
T. cruzi-negative_4887	0.07	0.47	0.09	0.09
T. cruzi-negative_4888	0.18	0.22	0.25	0.38
T. cruzi-negative_4889	0.28	0.15	0.16	0.16
T. cruzi-negative_4890	0.08	0.13	0.15	0.20
T. cruzi-negative_4891	0.03	0.09	0.06	0.05
T. cruzi-negative_4892	0.19	0.28	0.18	0.35
T. cruzi-negative_4893	0.20	0.26	0.17	0.29
T. cruzi-negative_4894	0.12	0.15	0.10	0.17
T. cruzi-negative_4895	0.12	0.16	0.10	0.16
T. cruzi-negative_4896	0.12	0.16	0.21	0.19
T. cruzi-negative_4897	0.14	0.19	0.11	0.22
T. cruzi-negative_4898	0.31	0.34	0.33	0.56
T. cruzi-negative_4899	0.36	0.19	0.10	0.18

*T. cruz*i-negative_4900	0.10	0.17	0.11	0.22
*T. cruz*i-negative_4901	0.13	0.20	0.14	0.22
*T. cruz*i-negative_4902	0.19	0.14	0.15	0.04
*T. cruz*i-negative_4903	0.10	0.12	0.09	0.09
*T. cruz*i-negative_4904	0.15	0.26	0.15	0.06
*T. cruz*i-negative_4905	0.12	0.16	0.12	0.21
*T. cruz*i-negative_4906	0.14	0.12	0.97	0.13
*T. cruz*i-negative_4907	0.15	0.18	0.39	0.08
*T. cruz*i-negative_4908	0.15	0.15	0.19	0.14
*T. cruz*i-negative_4909	0.10	0.14	0.10	0.15
*T. cruz*i-negative_4910	0.14	0.15	0.15	0.10
*T. cruz*i-negative_4911	0.28	0.26	0.41	0.11
*T. cruz*i-negative_4912	0.20	0.13	0.12	0.08
*T. cruz*i-negative_4913	0.16	0.20	0.17	0.13
*T. cruz*i-negative_4914	0.09	0.07	0.08	0.14
*T. cruz*i-negative_4915	0.11	0.18	0.10	0.06
*T. cruz*i-negative_4916	0.15	0.13	0.09	0.13
*T. cruz*i-negative_4917	0.16	0.16	0.16	0.21
*T. cruz*i-negative_4918	0.14	0.27	0.11	0.25
*T. cruz*i-negative_4919	0.18	0.16	0.25	0.21
*T. cruz*i-negative_4920	0.14	0.19	0.15	0.23
*T. cruz*i-negative_4921	0.37	0.18	0.33	0.09
*T. cruz*i-negative_4922	0.18	0.20	0.13	0.08
*T. cruz*i-negative_4923	0.19	0.23	0.13	0.10
*T. cruz*i-negative_4924	0.18	0.17	0.16	0.09
*T. cruz*i-negative_4925	0.08	0.08	0.09	0.04
*T. cruz*i-negative_4926	0.12	0.13	0.13	0.22
*T. cruz*i-negative_4927	0.14	0.17	0.13	0.06
*T. cruz*i-negative_4928	0.24	0.31	0.23	0.08
*T. cruz*i-negative_4929	0.36	0.59	0.39	0.13
*T. cruz*i-negative_4930	0.14	0.10	0.10	0.29
*T. cruz*i-negative_4931	0.13	0.15	0.16	0.07
*T. cruz*i-negative_4932	0.16	0.15	0.18	0.16
*T. cruz*i-negative_4933	0.14	0.38	0.18	0.07
*T. cruz*i-negative_4934	0.08	0.10	0.09	0.10
*T. cruz*i-negative_4935	0.19	0.17	0.57	0.07
*T. cruz*i-negative_4936	0.49	0.31	0.34	0.09
*T. cruz*i-negative_4937	0.14	0.17	0.18	0.05
*T. cruz*i-negative_4938	0.09	0.13	0.39	0.18
*T. cruz*i-negative_4939	0.11	0.47	0.09	0.27
*T. cruz*i-negative_4940	0.10	0.11	0.07	0.19
*T. cruz*i-negative_4941	0.20	0.18	0.21	0.06
*T. cruz*i-negative_4942	0.10	0.10	0.15	0.16

T. cruzi-negative_4943	0.04	0.07	0.30	0.15
T. cruzi-negative_4944	0.07	0.11	0.07	0.14
T. cruzi-negative_4945	0.14	0.15	0.57	0.08
T. cruzi-negative_4946	0.17	0.25	0.23	0.36
T. cruzi-negative_4947	0.11	0.13	0.11	0.21
T. cruzi-negative_4948	0.08	0.08	0.09	0.12
T. cruzi-negative_4949	0.22	0.17	0.18	0.09
T. cruzi-negative_4950	0.10	0.08	0.10	0.07
T. cruzi-negative_4951	0.13	0.07	0.25	0.11
T. cruzi-negative_4952	0.11	0.14	0.13	0.14
T. cruzi-negative_4953	0.13	0.18	0.09	0.12
T. cruzi-negative_4954	0.12	0.10	0.12	0.29
T. cruzi-negative_4955	0.24	0.26	0.16	0.13
T. cruzi-negative_4956	0.16	0.16	0.31	0.20
T. cruzi-negative_4957	0.24	0.28	0.50	0.09
T. cruzi-negative_4958	0.27	0.28	0.26	0.12
T. cruzi-negative_4959	0.13	0.09	0.10	0.09
T. cruzi-negative_4960	0.08	0.14	0.31	0.08
T. cruzi-negative_4961	0.21	0.21	0.16	0.04
T. cruzi-negative_4962	0.23	0.20	0.18	0.09
T. cruzi-negative_4963	0.07	0.11	0.17	0.09
T. cruzi-negative_4964	0.13	0.12	0.14	0.46
T. cruzi-negative_4965	0.09	0.11	0.13	0.11
T. cruzi-negative_4966	0.11	0.11	0.19	0.08
T. cruzi-negative_4967	0.19	0.17	0.12	0.11
T. cruzi-negative_4968	0.12	0.14	0.13	0.13
T. cruzi-negative_4969	0.13	0.23	0.28	0.29
T. cruzi-negative_4970	0.29	0.21	0.24	0.19
T. cruzi-negative_4971	0.12	0.10	0.14	0.14
T. cruzi-negative_4972	0.16	0.15	0.11	0.09
T. cruzi-negative_4973	0.11	0.14	0.16	0.23
T. cruzi-negative_4974	0.09	0.13	0.08	0.13
T. cruzi-negative_4975	0.16	0.09	0.16	0.14
T. cruzi-negative_4976	0.15	0.31	0.21	0.27
T. cruzi-negative_4977	0.19	0.13	0.18	0.43
T. cruzi-negative_4978	0.35	0.30	0.23	0.37
T. cruzi-negative_4979	0.16	0.19	0.19	0.30
T. cruzi-negative_4980	0.10	0.23	0.16	0.16
T. cruzi-negative_4981	0.16	0.17	0.15	0.33
T. cruzi-negative_4982	0.16	0.17	0.16	0.29
T. cruzi-negative_4983	0.19	0.25	0.15	0.33
T. cruzi-negative_4984	0.17	0.21	0.14	0.18

*T. cruz*i-negative_4985	0.20	0.22	0.16	0.21
*T. cruz*i-negative_4986	0.20	0.22	0.18	0.23
*T. cruz*i-negative_4987	0.23	0.24	0.18	0.23
*T. cruz*i-negative_4988	0.20	0.19	0.11	0.19
*T. cruz*i-negative_4989	0.29	0.25	0.20	0.29
*T. cruz*i-negative_4990	0.11	0.14	0.11	0.15
*T. cruz*i-negative_4991	0.20	0.22	0.19	0.31
*T. cruz*i-negative_4992	0.29	0.29	0.23	0.29
*T. cruz*i-negative_4993	0.30	0.26	0.22	0.32
