# Supplementary material for: Performance of Chimeric Trypanosoma cruzi Antigens in Serological Screening for Chagas Disease in Blood Banks
Source: Front Med (Lausanne). 2022 Mar 7;9:852864. doi: 10.3389/fmed.2022.852864 (PMC8940225; doi:10.3389/fmed.2022.852864)
Supplement: Supplementary file 2 [file Table_2.pdf]

Supplementary Material

Table S2. Reactivity Index for cross-reactivity assessment.

Samples	REACTIVITY INDEX			
	IBMP-8.1	IBMP-8.2	IBMP-8.3	IBMP-8.4
Anti-HBc_1	0.43	0.40	0.19	0.17
Anti-HBc_2	0.25	0.38	0.17	0.15
Anti-HBc_3	0.23	0.24	0.26	0.32
Anti-HBc_4	0.25	0.34	0.20	0.23
Anti-HBc_5	0.20	0.34	0.17	0.18
Anti-HBc_6	0.18	0.22	0.38	0.28
Anti-HBc_7	0.26	0.42	0.27	0.32
Anti-HBc_8	0.14	0.29	0.10	0.16
Anti-HBc_9	0.26	0.17	0.19	0.23
Anti-HBc_10	0.15	0.17	0.14	0.17
Anti-HBc_11	0.22	0.20	0.17	0.15
Anti-HBc_12	0.64	0.64	0.49	0.54
Anti-HBc_13	0.21	0.28	0.18	0.26
Anti-HBc_14	0.22	0.36	0.20	0.23
Anti-HBc_15	0.27	0.61	0.23	0.46
Anti-HBc_16	0.13	0.21	0.10	0.17
Anti-HBc_17	0.27	0.37	0.17	0.32
Anti-HBc_18	0.25	0.40	0.21	0.36
Anti-HBc_19	0.26	0.55	0.33	0.73
Anti-HBc_20	0.27	0.19	0.18	0.28
Anti-HBc_21	0.19	0.43	0.23	0.18
Anti-HBc_22	0.31	0.59	0.24	0.59
Anti-HBc_23	0.12	0.18	0.11	0.13
Anti-HBc_24	0.18	0.21	0.12	0.21
Anti-HBc_25	0.20	0.32	0.12	0.22
Anti-HBc_26	0.06	0.15	0.11	0.16
Anti-HBc_27	0.14	0.21	0.13	0.25
Anti-HBc_28	0.08	0.15	0.09	0.16
Anti-HBc_29	0.09	0.10	0.06	0.09
Anti-HBc_30	0.15	0.22	0.13	0.25
Anti-HBc_31	0.09	0.17	0.10	0.12
Anti-HBc_32	0.23	0.35	0.17	0.21
Anti-HBc_33	0.68	0.92	0.74	0.74

Anti-HBc_34	0.27	0.13	0.08	0.08
Anti-HBc_35	0.05	0.32	0.20	0.24
Anti-HBc_36	0.25	0.23	0.17	0.22
Anti-HBc_37	0.02	0.21	0.14	0.17
Anti-HBc_38	0.41	0.49	0.31	0.54
Anti-HBc_39	0.03	0.22	0.13	0.19
Anti-HBc_40	0.07	0.24	0.18	0.24
Anti-HBc_41	0.08	0.18	0.10	0.17
Anti-HBc_42	0.16	0.02	0.14	0.35
Anti-HBc_43	0.01	0.14	0.20	0.13
Anti-HBc_44	0.14	0.37	0.15	0.21
Anti-HBc_45	0.15	0.30	0.17	0.29
Anti-HBc_46	0.18	0.33	0.15	0.36
Anti-HBc_47	0.07	0.22	0.09	0.18
Anti-HBc_48	0.13	0.28	0.15	0.19
Anti-HBc_49	0.13	0.25	0.13	0.19
Anti-HBc_50	0.09	0.20	0.10	0.18
Anti-HBc_51	0.12	0.33	0.18	0.27
Anti-HBc_52	0.07	0.18	0.15	0.14
Anti-HBc_53	0.25	0.72	0.34	0.52
Anti-HBc_54	0.15	0.14	0.15	0.08
Anti-HBc_55	0.14	0.20	0.17	0.09
Anti-HBc_56	0.08	0.10	0.11	0.01
Anti-HBc_57	0.16	0.35	0.25	0.17
Anti-HBc_58	0.07	0.10	0.09	0.05
Anti-HBc_59	0.22	0.20	0.21	0.13
Anti-HBc_60	0.18	0.21	0.34	0.24
Anti-HBc_61	0.17	0.16	0.23	0.09
Anti-HBc_62	0.26	0.38	0.29	0.28
Anti-HBc_63	0.15	0.16	0.27	0.10
Anti-HBc_64	0.14	0.29	0.24	0.20
Anti-HBc_65	0.10	0.24	0.19	0.15
Anti-HBc_66	0.04	0.12	0.12	0.06
Anti-HBc_67	0.11	0.22	0.20	0.12
Anti-HBc_68	0.16	0.19	0.21	0.14
Anti-HBc_69	0.12	0.24	0.13	0.11
Anti-HBc_70	0.09	0.19	0.18	0.07
Anti-HBc_71	0.14	0.29	0.12	0.22
Anti-HBc_72	0.07	0.19	0.16	0.07
Anti-HBc_73	0.09	0.27	0.15	0.11
Anti-HBc_74	0.32	0.32	0.18	0.21
Anti-HBc_75	0.24	0.13	0.20	0.14

Anti-HBc_76	0.14	0.03	0.11	0.10
Anti-HBc_77	0.26	0.15	0.27	0.22
Anti-HBc_78	0.13	0.06	0.09	0.05
Anti-HBc_79	0.18	0.13	0.11	0.09
Anti-HBc_80	0.14	0.07	0.07	0.03
Anti-HBc_81	0.13	0.07	0.06	0.04
Anti-HBc_82	0.12	0.07	0.04	0.08
Anti-HBc_83	0.14	0.13	0.34	0.12
Anti-HBc_84	0.16	0.10	0.08	0.11
Anti-HBc_85	0.55	0.70	0.53	0.95
Anti-HBc_86	0.17	0.13	0.09	0.15
Anti-HBc_87	0.33	0.23	0.20	0.31
Anti-HBc_88	0.12	0.05	0.03	0.04
Anti-HBc_89	0.08	0.21	0.13	0.21
Anti-HBc_90	0.06	0.21	0.20	0.22
Anti-HBc_91	0.02	0.13	0.45	0.10
Anti-HBc_92	0.04	0.09	0.08	0.04
Anti-HBc_93	0.07	0.14	0.25	0.11
Anti-HBc_94	0.03	0.10	0.09	0.08
Anti-HBc_95	0.05	0.14	0.63	0.09
Anti-HBc_96	0.02	0.12	0.12	0.12
Anti-HBc_97	0.02	0.33	0.12	0.06
Anti-HBc_98	0.07	0.19	0.14	0.15
Anti-HBc_99	0.32	0.34	0.23	0.37
Anti-HBc_100	0.08	0.21	0.13	0.13
Anti-HBc_101	0.01	0.06	0.06	0.02
Anti-HBc_102	0.12	0.31	0.23	0.33
Anti-HBc_103	0.10	0.15	0.09	0.16
Anti-HBc_104	0.15	0.35	0.27	0.43
Anti-HBc_105	0.07	0.05	0.06	0.05
Anti-HBc_106	0.07	0.18	0.12	0.15
Anti-HBc_107	0.01	0.08	0.06	0.04
Anti-HBc_108	0.09	0.20	0.19	0.19
Anti-HBc_109	0.06	0.13	0.12	0.11
Anti-HBc_110	0.05	0.08	0.10	0.16
Anti-HBc_111	0.10	0.17	0.11	0.13
Anti-HBc_112	0.05	0.14	0.22	0.16
Anti-HBc_113	0.09	0.08	0.06	0.08
Anti-HBc_114	0.11	0.22	0.21	0.23
Anti-HBc_115	0.10	0.20	0.14	0.29
Anti-HBc_116	0.08	0.19	0.15	0.20
Anti-HBc_117	0.09	0.21	0.22	0.18
Anti-HBc_118	0.08	0.21	0.15	0.10

Anti-HBc_119	0.03	0.13	0.10	0.04
Anti-HBc_120	0.12	0.11	0.10	0.08
Anti-HBc_121	0.14	0.29	0.14	0.41
Anti-HBc_122	0.24	0.56	0.20	0.62
Anti-HBc_123	0.16	0.20	0.17	0.33
Anti-HBc_124	0.14	0.18	0.12	0.22
Anti-HBc_125	0.07	0.12	0.14	0.17
Anti-HBc_126	0.17	0.28	0.30	0.31
Anti-HBc_127	0.13	0.18	0.11	0.27
Anti-HBc_128	0.09	0.15	0.14	0.21
Anti-HBc_129	0.05	0.13	0.06	0.16
Anti-HBc_130	0.06	0.14	0.08	0.12
Anti-HBc_131	0.53	0.23	0.10	0.22
Anti-HBc_132	0.03	0.06	0.07	0.09
Anti-HBc_133	0.35	0.24	0.32	0.61
Anti-HBc_134	0.08	0.11	0.14	0.17
Anti-HBc_135	0.06	0.17	0.05	0.06
Anti-HBc_136	0.07	0.15	0.07	0.11
Anti-HBc_137	0.07	0.10	0.08	0.10
Anti-HBc_138	0.09	0.12	0.09	0.15
Anti-HBc_139	0.07	0.14	0.10	0.19
Anti-HBc_140	0.08	0.12	0.11	0.26
Anti-HBc_141	0.04	0.10	0.08	0.16
Anti-HBc_142	0.06	0.15	0.08	0.08
Anti-HBc_143	0.10	0.20	0.16	0.21
Anti-HBc_144	0.13	0.17	0.13	0.21
Anti-HBc_145	0.13	0.21	0.33	0.33
Anti-HBc_146	0.06	0.13	0.10	0.17
Anti-HBc_147	0.14	0.18	0.18	0.32
Anti-HBc_148	0.06	0.12	0.11	0.14
Anti-HBc_149	0.15	0.19	0.16	0.32
Anti-HBc_150	0.08	0.16	0.12	0.17
Anti-HBc_151	0.16	0.17	0.25	0.30
Anti-HBc_152	0.06	0.11	0.13	0.18
Anti-HBc_153	0.03	0.10	0.05	0.10
Anti-HBc_154	0.10	0.17	0.16	0.22
Anti-HBc_155	0.08	0.15	0.12	0.17
Anti-HBc_156	0.08	0.16	0.12	0.19
Anti-HBc_157	0.10	0.15	0.15	0.19
Anti-HBc_158	0.12	0.27	0.10	0.26
Anti-HBc_159	0.12	0.17	0.16	0.41
Anti-HBc_160	0.07	0.13	0.15	0.17

Anti-HBc_161	0.11	0.12	0.09	0.13
Anti-HBc_162	0.11	0.16	0.14	0.23
Anti-HBc_163	0.02	0.07	0.05	0.05
Anti-HBc_164	0.08	0.11	0.16	0.17
Anti-HBc_165	0.10	0.17	0.10	0.23
Anti-HBc_166	0.15	0.21	0.16	0.31
Anti-HBc_167	0.12	0.20	0.16	0.24
Anti-HBc_168	0.12	0.15	0.10	0.17
Anti-HBc_169	0.07	0.47	0.09	0.09
Anti-HBc_170	0.28	0.15	0.16	0.16
Anti-HBc_171	0.08	0.13	0.15	0.20
Anti-HBc_172	0.03	0.09	0.06	0.05
Anti-HBc_173	0.19	0.28	0.18	0.35
Anti-HBc_174	0.20	0.26	0.17	0.29
Anti-HBc_175	0.12	0.15	0.10	0.17
Anti-HBc_176	0.12	0.16	0.21	0.19
Anti-HBc_177	0.14	0.19	0.11	0.22
Anti-HBc_178	0.31	0.34	0.33	0.56
Anti-HBc_179	0.36	0.19	0.10	0.18
Anti-HBc_180	0.19	0.14	0.15	0.04
Anti-HBc_181	0.10	0.12	0.09	0.09
Anti-HBc_182	0.15	0.26	0.15	0.06
Anti-HBc_183	0.12	0.16	0.12	0.21
Anti-HBc_184	0.28	0.26	0.41	0.11
Anti-HBc_185	0.09	0.07	0.08	0.14
Anti-HBc_186	0.16	0.16	0.16	0.21
Anti-HBc_187	0.14	0.27	0.11	0.25
Anti-HBc_188	0.18	0.16	0.25	0.21
Anti-HBc_189	0.14	0.19	0.15	0.23
Anti-HBc_190	0.18	0.20	0.13	0.08
Anti-HBc_191	0.18	0.17	0.16	0.09
Anti-HBc_192	0.08	0.08	0.09	0.04
Anti-HBc_193	0.24	0.31	0.23	0.08
Anti-HBc_194	0.36	0.59	0.39	0.13
Anti-HBc_195	0.14	0.10	0.10	0.29
Anti-HBc_196	0.13	0.15	0.16	0.07
Anti-HBc_197	0.14	0.38	0.18	0.07
Anti-HBc_198	0.14	0.17	0.18	0.05
Anti-HBc_199	0.09	0.13	0.39	0.18
Anti-HBc_200	0.11	0.47	0.09	0.27
Anti-HBc_201	0.10	0.11	0.07	0.19
Anti-HBc_202	0.20	0.18	0.21	0.06
Anti-HBc_203	0.04	0.07	0.30	0.15

Anti-HBc_204	0.07	0.11	0.07	0.14
Anti-HBc_205	0.14	0.15	0.57	0.08
Anti-HBc_206	0.17	0.25	0.23	0.36
Anti-HBc_207	0.11	0.13	0.11	0.21
Anti-HBc_208	0.13	0.07	0.25	0.11
Anti-HBc_209	0.11	0.14	0.13	0.14
Anti-HBc_210	0.13	0.18	0.09	0.12
Anti-HBc_211	0.24	0.26	0.16	0.13
Anti-HBc_212	0.16	0.16	0.31	0.20
Anti-HBc_213	0.24	0.28	0.50	0.09
Anti-HBc_214	0.13	0.09	0.10	0.09
Anti-HBc_215	0.08	0.14	0.31	0.08
Anti-HBc_216	0.21	0.21	0.16	0.04
Anti-HBc_217	0.23	0.20	0.18	0.09
Anti-HBc_218	0.07	0.11	0.17	0.09
Anti-HBc_219	0.13	0.12	0.14	0.46
Anti-HBc_220	0.11	0.11	0.19	0.08
Anti-HBc_221	0.19	0.17	0.12	0.11
Anti-HBc_222	0.12	0.14	0.13	0.13
Anti-HBc_223	0.29	0.21	0.24	0.19
Anti-HBc_224	0.12	0.10	0.14	0.14
Anti-HBc_225	0.09	0.13	0.08	0.13
Anti-HBc_226	0.16	0.17	0.16	0.29
Anti-HBc_227	0.19	0.25	0.15	0.33
Anti-HBc_228	0.20	0.22	0.16	0.21
Anti-HBc_229	0.20	0.22	0.18	0.23
Anti-HBc_230	0.23	0.24	0.18	0.23
Anti-HBc_231	0.20	0.19	0.11	0.19
Anti-HBc_232	0.20	0.22	0.19	0.31
Anti-HBc_233	0.29	0.29	0.23	0.29
Syphilis_1	0.14	0.36	0.20	0.19
Syphilis_2	0.19	0.22	0.39	0.19
Syphilis_3	0.19	0.19	0.14	0.15
Syphilis_4	0.20	0.19	0.15	0.15
Syphilis_5	0.18	0.20	0.21	0.21
Syphilis_6	0.11	0.17	0.09	0.11
Syphilis_7	0.24	0.30	0.19	0.20
Syphilis_8	0.21	0.24	0.16	0.14
Syphilis_9	0.17	0.20	0.09	0.22
Syphilis_10	0.19	0.23	0.11	0.14
Syphilis_11	0.20	0.14	0.28	0.13
Syphilis_12	0.23	0.31	0.22	0.24

Syphilis_13	0.16	0.19	0.11	0.13
Syphilis_14	0.23	0.31	0.20	0.26
Syphilis_15	0.12	0.14	0.21	0.16
Syphilis_16	0.23	0.25	0.17	0.16
Syphilis_17	0.30	0.48	0.15	0.40
Syphilis_18	0.39	0.39	0.14	0.42
Syphilis_19	0.16	0.37	0.19	0.32
Syphilis_20	0.07	0.08	0.02	0.17
Syphilis_21	0.14	0.28	0.15	0.28
Syphilis_22	0.07	0.09	0.10	0.22
Syphilis_23	0.05	0.10	0.10	0.01
Syphilis_24	0.03	0.45	0.27	0.61
Syphilis_25	0.25	0.47	0.25	0.72
Syphilis_26	0.21	0.25	0.17	0.24
Syphilis_27	0.01	0.16	0.07	0.12
Syphilis_28	0.11	0.19	0.10	0.13
Syphilis_29	0.15	0.23	0.15	0.19
Syphilis_30	0.01	0.10	0.03	0.11
Syphilis_31	0.04	0.08	0.09	0.14
Syphilis_32	0.07	0.18	0.11	0.15
Syphilis_33	0.05	0.13	0.13	0.11
Syphilis_34	0.07	0.10	0.05	0.08
Syphilis_35	0.05	0.16	0.11	0.15
Syphilis_36	0.18	0.30	0.15	0.20
Syphilis_37	0.01	0.18	0.07	0.11
Syphilis_38	0.05	0.20	0.10	0.15
Syphilis_39	0.13	0.43	0.11	0.14
Syphilis_40	0.10	0.16	0.07	0.17
Syphilis_41	0.05	0.34	0.12	0.16
Syphilis_42	0.17	0.34	0.17	0.26
Syphilis_43	0.06	0.24	0.12	0.19
Syphilis_44	0.10	0.18	0.16	0.24
Syphilis_45	0.03	0.18	0.08	0.12
Syphilis_46	0.14	0.03	0.11	0.19
Syphilis_47	0.18	0.32	0.24	0.32
Syphilis_48	0.06	0.25	0.11	0.20
Syphilis_49	0.07	0.20	0.17	0.37
Syphilis_50	0.10	0.24	0.18	0.11
Syphilis_51	0.05	0.16	0.16	0.06
Syphilis_52	0.11	0.23	0.25	0.12
Syphilis_53	0.18	0.38	0.28	0.19
Syphilis_54	0.21	0.29	0.27	0.24
Syphilis_55	0.18	0.21	0.18	0.12

Syphilis_56	0.07	0.15	0.15	0.07
Syphilis_57	0.16	0.37	0.17	0.16
Syphilis_58	0.10	0.33	0.09	0.09
Syphilis_59	0.12	0.28	0.19	0.12
Syphilis_60	0.13	0.25	0.28	0.15
Syphilis_61	0.14	0.25	0.15	0.14
Syphilis_62	0.17	0.29	0.10	0.16
Syphilis_63	0.13	0.24	0.11	0.15
Syphilis_64	0.12	0.24	0.12	0.07
Syphilis_65	0.04	0.15	0.15	0.06
Syphilis_66	0.05	0.11	0.08	0.05
Syphilis_67	0.16	0.31	0.13	0.15
Syphilis_68	0.15	0.27	0.18	0.11
Syphilis_69	0.07	0.11	0.10	0.03
Syphilis_70	0.18	0.54	0.17	0.18
Syphilis_71	0.12	0.43	0.10	0.10
Syphilis_72	0.20	0.26	0.32	0.22
Syphilis_73	0.12	0.13	0.08	0.05
Syphilis_74	0.14	0.06	0.06	0.07
Syphilis_75	0.24	0.15	0.24	0.14
Syphilis_76	0.16	0.12	0.08	0.09
Syphilis_77	0.11	0.10	0.22	0.11
Syphilis_78	0.27	0.17	0.16	0.19
Syphilis_79	0.20	0.09	0.15	0.15
Syphilis_80	0.22	0.14	0.15	0.11
Syphilis_81	0.26	0.13	0.16	0.14
Syphilis_82	0.18	0.11	0.12	0.09
Syphilis_83	0.12	0.11	0.06	0.05
Syphilis_84	0.16	0.11	0.12	0.08
Syphilis_85	0.13	0.08	0.26	0.06
Syphilis_86	0.16	0.10	0.10	0.07
Syphilis_87	0.17	0.11	0.07	0.08
Syphilis_88	0.24	0.17	0.15	0.21
Syphilis_89	0.09	0.10	0.05	0.07
Syphilis_90	0.18	0.09	0.25	0.08
Syphilis_91	0.13	0.23	0.03	0.04
Syphilis_92	0.19	0.19	0.12	0.20
Syphilis_93	0.18	0.13	0.33	0.11
Syphilis_94	0.17	0.08	0.07	0.07
Syphilis_95	0.15	0.12	0.10	0.08
Syphilis_96	0.12	0.10	0.09	0.10
Syphilis_97	0.04	0.08	0.13	0.08

Syphilis_98	0.02	0.08	0.06	0.05
Syphilis_99	0.02	0.11	0.24	0.08
Syphilis_100	0.14	0.21	0.24	0.17
Syphilis_101	0.14	0.18	0.18	0.17
Syphilis_102	0.02	0.10	0.05	0.05
Syphilis_103	0.01	0.05	0.03	0.02
Syphilis_104	0.06	0.14	0.15	0.19
Syphilis_105	0.07	0.12	0.10	0.05
Syphilis_106	0.04	0.14	0.10	0.10
Syphilis_107	0.02	0.13	0.12	0.10
Syphilis_108	0.07	0.12	0.08	0.08
Syphilis_109	0.05	0.09	0.06	0.05
Syphilis_110	0.13	0.24	0.12	0.16
Syphilis_111	0.06	0.12	0.12	0.10
Syphilis_112	0.08	0.15	0.09	0.10
Syphilis_113	0.12	0.24	0.23	0.20
Syphilis_114	0.72	0.56	0.18	0.12
Syphilis_115	0.08	0.14	0.16	0.07
Syphilis_116	0.03	0.09	0.10	0.17
Syphilis_117	0.09	0.14	0.14	0.26
Syphilis_118	0.05	0.09	0.08	0.15
Syphilis_119	0.10	0.13	0.11	0.20
Syphilis_120	0.11	0.19	0.12	0.16
Syphilis_121	0.10	0.15	0.08	0.13
Syphilis_122	0.03	0.15	0.07	0.07
Syphilis_123	0.10	0.12	0.09	0.16
Syphilis_124	0.05	0.21	0.06	0.11
Syphilis_125	0.07	0.12	0.09	0.20
Syphilis_126	0.11	0.10	0.15	0.16
Syphilis_127	0.20	0.32	0.20	0.48
Syphilis_128	0.12	0.20	0.14	0.35
Syphilis_129	0.18	0.22	0.25	0.38
Syphilis_130	0.12	0.16	0.10	0.16
Syphilis_131	0.13	0.20	0.14	0.22
Syphilis_132	0.15	0.15	0.19	0.14
Syphilis_133	0.10	0.14	0.10	0.15
Syphilis_134	0.14	0.15	0.15	0.10
Syphilis_135	0.20	0.13	0.12	0.08
Syphilis_136	0.11	0.18	0.10	0.06
Syphilis_137	0.19	0.23	0.13	0.10
Syphilis_138	0.12	0.13	0.13	0.22
Syphilis_139	0.14	0.17	0.13	0.06
Syphilis_140	0.16	0.15	0.18	0.16

Syphilis_141	0.08	0.10	0.09	0.10
Syphilis_142	0.22	0.17	0.18	0.09
Syphilis_143	0.10	0.08	0.10	0.07
Syphilis_144	0.27	0.28	0.26	0.12
Syphilis_145	0.16	0.15	0.11	0.09
Syphilis_146	0.16	0.09	0.16	0.14
Syphilis_147	0.16	0.19	0.19	0.30
Syphilis_148	0.16	0.17	0.15	0.33
Syphilis_149	0.17	0.21	0.14	0.18
Syphilis_150	0.29	0.25	0.20	0.29
HTLV_1	0.83	0.59	0.80	0.76
HTLV_2	0.16	0.30	0.17	0.12
HTLV_3	0.08	0.10	0.14	0.11
HTLV_4	0.10	0.17	0.11	0.08
HTLV_5	0.20	0.24	0.21	0.17
HTLV_6	0.29	0.30	0.26	0.25
HTLV_7	0.10	0.25	0.08	0.25
HTLV_8	0.12	0.13	0.06	0.08
HTLV_9	0.16	0.38	0.23	0.33
HTLV_10	0.15	0.27	0.44	0.51
HTLV_11	0.08	0.16	0.08	0.03
HTLV_12	0.11	0.06	0.09	0.06
HTLV_13	0.13	0.13	0.23	0.08
HTLV_14	0.12	0.07	0.79	0.15
HTLV_15	0.08	0.04	0.02	0.02
HTLV_16	0.17	0.18	0.14	0.14
HTLV_17	0.32	0.22	0.20	0.27
HTLV_18	0.04	0.16	0.15	0.15
HTLV_19	0.07	0.11	0.08	0.08
HTLV_20	0.05	0.12	0.10	0.13
HTLV_21	0.02	0.09	0.04	0.08
HTLV_22	0.04	0.14	0.10	0.13
HTLV_23	0.02	0.15	0.07	0.10
HTLV_24	0.10	0.22	0.12	0.15
HTLV_25	0.06	0.18	0.18	0.14
HTLV_26	0.04	0.12	0.06	0.06
HTLV_27	0.12	0.18	0.11	0.14
HTLV_28	0.10	0.29	0.26	0.36
HTLV_29	0.04	0.09	0.06	0.09
HTLV_30	0.11	0.13	0.22	0.17
HTLV_31	0.07	0.12	0.07	0.17
HTLV_32	0.10	0.17	0.11	0.22

HTLV_33	0.15	0.18	0.39	0.08
HTLV_34	0.08	0.08	0.09	0.12
HTLV_35	0.19	0.13	0.18	0.43
HTLV_36	0.10	0.23	0.16	0.16
HTLV_37	0.11	0.14	0.11	0.15
HIV_1	0.25	0.28	0.17	0.15
HIV_2	0.35	0.38	0.23	0.45
HIV_3	0.28	0.21	0.11	0.15
HIV_4	0.58	0.16	0.10	0.17
HIV_5	0.20	0.41	0.25	0.42
HIV_6	0.22	0.24	0.28	0.14
HIV_7	0.08	0.10	0.12	0.03
HIV_8	0.14	0.17	0.08	0.10
HIV_9	0.23	0.60	0.18	0.22
HIV_10	0.17	0.09	0.16	0.11
HIV_11	0.14	0.09	0.07	0.10
HIV_12	0.22	0.32	0.26	0.53
HIV_13	0.26	0.24	0.21	0.37
HIV_14	0.14	0.24	0.34	0.29
HIV_15	0.09	0.22	0.18	0.13
HIV_16	0.34	0.49	0.45	0.72
HIV_17	0.06	0.13	0.10	0.23
HIV_18	0.10	0.20	0.09	0.24
HIV_19	0.15	0.13	0.09	0.13
HIV_20	0.13	0.23	0.28	0.29
HCV_1	0.23	0.24	0.19	0.21
HCV_2	0.11	0.21	0.16	0.20
HCV_3	0.23	0.42	0.18	0.22
HCV_4	0.33	0.38	0.15	0.33
HCV_5	0.31	0.49	0.21	0.34
HCV_6	0.33	0.27	0.24	0.37
HCV_7	0.12	0.22	0.12	0.16
HCV_8	0.06	0.14	0.11	0.06
HCV_9	0.13	0.08	0.15	0.05
HCV_10	0.11	0.30	0.31	0.32
HCV_11	0.05	0.09	0.07	0.09
HCV_12	0.16	0.20	0.17	0.13
HCV_13	0.49	0.31	0.34	0.09
HCV_14	0.12	0.10	0.12	0.29
HCV_15	0.11	0.14	0.16	0.23
HBsAG_1	0.14	0.21	0.12	0.12
HBsAG_2	0.06	0.24	0.10	0.17
HBsAG_3	0.14	0.24	0.18	0.31

HBsAG_4	0.18	0.38	0.24	0.19
HBsAG_5	0.06	0.27	0.16	0.22
HBsAG_6	0.01	0.12	0.04	0.10
HBsAG_7	0.13	0.35	0.18	0.29
HBsAG_8	0.10	0.29	0.14	0.15
HBsAG_9	0.07	0.14	0.20	0.08
HBsAG_10	0.08	0.13	0.12	0.08
HBsAG_11	0.13	0.15	0.09	0.08
HBsAG_12	0.36	0.57	0.38	0.78
Mixed_1	0.08	0.51	0.21	0.38
Mixed_2	0.11	0.19	0.11	0.18
Mixed_3	0.13	0.14	0.10	0.10
Mixed_4	0.19	0.10	0.10	0.08
Mixed_5	0.14	0.12	0.09	0.10
Mixed_6	0.13	0.25	0.18	0.25
Mixed_7	0.29	0.35	0.26	0.35
Mixed_8	0.33	0.06	0.04	0.03
Mixed_9	0.21	0.18	0.18	0.10
Mixed_10	0.23	0.23	0.07	0.10
Mixed_11	0.23	0.36	0.19	0.25
Mixed_12	0.19	0.57	0.38	0.78
Mixed_13	0.31	0.16	0.10	0.12
Mixed_14	0.29	0.26	0.24	0.26
